# On the Stability of Periodic Multi-Solitons of the KdV Equation

**DOI:** 10.1007/s00220-021-04089-9

**Published:** 2021-05-11

**Authors:** Thomas Kappeler, Riccardo Montalto

**Affiliations:** 1 Institut für Mathematik, Universität Zürich, Winterthurerstrasse 190, 8057 Zürich, Switzerland; 2 University of Milan, Via Saldini 50, 20133 Milan, Italy

## Abstract

In this paper we obtain the following stability result for periodic multi-solitons of the KdV equation: We prove that under any given semilinear Hamiltonian perturbation of small size $\varepsilon > 0$, a large class of periodic multi-solitons of the KdV equation, including ones of large amplitude, are orbitally stable for a time interval of length at least $O(\varepsilon ^{-2})$. To the best of our knowledge, this is the first stability result of such type for periodic multi-solitons of large size of an integrable PDE.

## Introduction

The Korteweg-de Vries (KdV) equation

1.1 $$\begin{aligned} \partial _t u = - \partial _x^3 u + 6 u \partial _x u \end{aligned}$$

is one of the most important model equations for describing dispersive phenomena. It is named after the two Dutch mathematician Korteweg and de Vries [29] (cf. also Boussinesq [14], Raleigh [40]) and originally was proposed as a model equation in one space dimension for long surface waves of water in a narrow and shallow channel. Today it is used in many branches of physics as well as in the engineering sciences. The seminal discovery in the late sixties that (1.1) admits infinitely many conservation laws ([34, 38]), and the development of the inverse scattering transform method ( [24]) led to the modern theory of integrable systems of finite and infinite dimension (see e.g. [20, 22], and references therein). More recently, as one of the most prominent examples among dispersive equations, (1.1) played a major role in the development of the theory of dispersive PDEs to which many of the leading analysts of our times contributed. In particular, the (globally in time) well-posedness theory of (1.1) has been established in various setups in great detail – see [19].

A distinguished feature of Eq. (1.1) is the existence of sharply localized traveling wave solutions of arbitrarily large amplitude and particle like properties. Kruskal and Zabusky, who studied them in numerical experiments in the early sixties (cf. [30]), coined the name ’soliton’ for them. More generally, they found solutions, which are localized near finitely many points in space, referred to as multi-solitons. In the periodic setup, these solutions often are referred to as *periodic multi-solitons* or *finite gap solutions*. Due to their importance in applications, various stability aspects have been considered such as the long time asymptotics of solutions with initial data near (periodic) multi-solitons (orbital stability, soliton resolution conjecture). Two major questions arise in connection with the *structural stability* of (1.1). One of them concerns the persistence of the (periodic) multi-solitons under perturbations of (1.1), and the other one concerns the long time asymptotics of solutions of perturbations of (1.1) with initial data close to a (periodic) multi-soliton. In the periodic setup, the first question has been studied quite extensively by developing KAM methods, pioneered by Kolmogorov, Arnold, and Moser to treat perturbations of finite dimensional integrable system, for PDEs (cf. [1, 8, 12, 15, 28, 31–33, 35, 39, 41], and references therein), whereas the second one turned out to be quite challenging and little is known so far. Our goal is to address this longstanding open problem.

The aim of this paper is to study in the periodic setup the long time asymptotics of the solutions of Hamiltonian perturbations of (1.1) with initial data close to a periodic multi-soliton of arbitrary large amplitude. To describe the class of perturbations considered, recall that (1.1) with the space periodic variable $x \in {\mathbb {T}}_1:= {\mathbb {R}}/ {\mathbb {Z}}$ can be written in Hamiltonian form

1.2 $$\begin{aligned} \partial _t u = \partial _x \nabla H^{kdv}(u)\ , \qquad H^{kdv}(u) := \int _0^1 \big ( \frac{1}{2} (\partial _x u)^2 + u^3 \big ) d x \ , \end{aligned}$$

where $\nabla H^{kdv}(u)$ denotes the $L^2-$gradient of $H^{kdv}$ and where $\partial _x$ is the Poisson structure, corresponding to the Poisson bracket defined for functionals *F*, *G* by

1.3 $$\begin{aligned} \{ F, G \} (u) = \int _0^1 \nabla F \partial _x \nabla G d x. \end{aligned}$$

We consider semilinear Hamiltonian perturbations of (1.1) of the form

1.4 $$\begin{aligned} \partial _t u = - \partial _x^3 u + 6 u \partial _x u + \varepsilon F(u) \end{aligned}$$

where $0< \varepsilon <1$ is a small parameter and *F* is a semilinear Hamiltonian vector field

1.5 $$\begin{aligned} F(u) = \partial _x \nabla {P_f}(u). \end{aligned}$$

Here $P_f$ is a Hamiltonian of the form

1.6 $$\begin{aligned} { P_f}(u) := \int _0^1 f(x, u(x))\, d x \end{aligned}$$

and *f* a $C^{\infty }-$smooth density

1.7 $$\begin{aligned} f : {\mathbb {T}}_1 \times {\mathbb {R}}\rightarrow {\mathbb {R}}, \quad (x, \zeta ) \mapsto f(x, \zeta ) , \end{aligned}$$

so that with $f'(x, \zeta ) := \partial _\zeta f(x, \zeta )$ and $f''(x, \zeta ) := \partial ^2_\zeta f(x, \zeta )$,

$$\begin{aligned} F(u)(x) = \partial _x \nabla P_f(u)(x) = \partial _x f'(x, u(x)) + f''(x, u(x))\partial _x u(x). \end{aligned}$$

To state our main results, we first need to introduce some more notations. Since $u \mapsto \langle u \rangle _x := \int _0^1 u \, d x$ is a Casimir for the Poisson bracket (1.3) and hence a prime integral of (1.4), we restrict our attention to spaces of functions with zero mean (cf. [28], Section 13) and choose as phase spaces of (1.4) the scale of Sobolev spaces $H^s_0({\mathbb {T}}_1)$, $s \in {\mathbb {Z}}_{\ge 0}$,

$$\begin{aligned} H^s_0({\mathbb {T}}_1) := \{ q \in H^s({\mathbb {T}}_1) \ : \ \int _0^1 q(x) d x = 0 \} , \qquad L^2_0 ({\mathbb {T}}_1) \equiv H^0_0({\mathbb {T}}_1) , \end{aligned}$$

where

1.8 $$\begin{aligned} H^s({\mathbb {T}}_1) \equiv H^s({\mathbb {T}}_1, {\mathbb {R}}) := \big \{ q = \sum _{n \in {\mathbb {Z}}} q_n e^{2 \pi \mathrm{i} n x} \ : \, q_n \in {\mathbb {C}}, \ q_{-n} = {\overline{q}}_n \ \forall n \in {\mathbb {Z}}, \ \Vert q \Vert _s < \infty \big \},\nonumber \\ \end{aligned}$$

and

$$\begin{aligned} \Vert q \Vert _s = \big ( \sum _{n \in {\mathbb {Z}}} \langle n \rangle ^{2 s} |q_n|^2 \big )^{\frac{1}{2}} \ , \qquad \langle n \rangle := \mathrm{max}\{1, |n| \} \ , \quad \ \forall \ n \in {\mathbb {Z}}\ . \end{aligned}$$

On $L^2_0({\mathbb {T}}_1)$, the Poisson structure $\partial _x$ is nondegenerate and the corresponding symplectic form is given by

1.9 $$\begin{aligned} {{\mathcal {W}}}_{L^2_0} (u, v) := \int _0^1 (\partial _x^{- 1} u ) v\, d x \, , \qquad \partial _x^{- 1} u = \sum _{n \ne 0} \frac{1}{\mathrm{i} n} u_n e^{\mathrm{i} 2 \pi n x}\, , \qquad \forall u, v \in L^2_0({\mathbb {T}}_1).\nonumber \\ \end{aligned}$$

Note that the Hamiltonian vector field $X_H (u) = \partial _x \nabla H (u)$, associated with the Hamiltonian *H*, satisfies $d H (u)[ \cdot ] = {{\mathcal {W}}}_{L^2_0} ( X_H , \cdot )$.

Our results can informally be stated as follows: for any $f \in C^{\infty }({\mathbb {T}}_1 \times {\mathbb {R}})$, *s* sufficiently large, $\varepsilon > 0$ sufficiently small, and for most of the finite gap solutions $q: t \mapsto q(t, \cdot )$ of (1.1), the following holds: for any initial data $u_0 \in H^s_0({\mathbb {T}}_1)$, which is $\varepsilon$-close in $H^s_0({\mathbb {T}}_1)$ to the orbit ${\mathcal {O}}_q := \{ q(t, \cdot ) : \, t \in {\mathbb {R}}\}$ of *q*, the perturbed equation (1.4) admits a unique solution $t \mapsto u(t, \cdot )$ in $H^s_0({\mathbb {T}}_1)$ with initial data $u(0, \cdot ) = u_0$ and life span at least $[- T, T]$, $T = O(\varepsilon ^{-2})$. The solution $u(t, \cdot )$ stays $\varepsilon$-close in $H^s_0({\mathbb {T}}_1)$ to the orbit ${\mathcal {O}}_q$.

To state our results in precise terms, we need to define the notion of finite gap solution and the invariant tori, on which they evolve, and explain for which of these solutions the above stability results hold. Since these finite gap solutions are not small, we need to introduce coordinates to describe them. Most conveniently, this can be done in terms of a Euclidean version of action angle coordinates, referred to as Birkhoff coordinates. Let us now explain this in detail.

According to [28], the KdV equation (1.2) on ${\mathbb {T}}_1$ is an integrable PDE in the strongest possible sense, meaning that it admits globally defined canonical coordinates on $L^2_0({\mathbb {T}}_1)$ so that when expressed in these coordinates, (1.2) can be solved by quadrature.

To describe these coordinates in more detail, we introduce for any $s \in {\mathbb {Z}}_{\ge 0}$ the weighted $\ell ^2-$sequence spaces

$$\begin{aligned} h_0^s := \big \{ (w_n)_{n \ne 0} \in h^s_{0, c} \ : \ w_{- n} = \overline{w}_n \,\, \forall n \ge 1 \big \} , \qquad \ell ^2_0 \equiv h^0_0, \end{aligned}$$

where $h^s_{0, c} \equiv h^s({\mathbb {Z}}{\setminus } \{ 0 \}, {\mathbb {C}})$ is given by

$$\begin{aligned} h^s_{0,c} : = \big \{ w = (w_n)_{n \ne 0} : w_n \in {\mathbb {C}}\,\, \forall n \ne 0, \ \Vert w \Vert _s < \infty \big \} , \quad \Vert w \Vert _s {:=} \big ( \sum _{n \ne 0} |n|^{2 s} |w_n|^2 \big )^{\frac{1}{2}} . \end{aligned}$$

By [28] there exists a real analytic diffeomorphism, referred to as (complex) Birkhoff map,

$$\begin{aligned} \Phi ^{kdv} : L^2_0({\mathbb {T}}_1) \rightarrow \ell ^2_0, \quad q \mapsto w(q) := (w_n(q))_{n \ne 0}\,, \end{aligned}$$

which is canonical in the sense that

1.10 $$\begin{aligned} \{ w_n, w_{- n} \} = \int _0^1 \nabla w_n \partial _x \nabla w_{- n}\, d x = 2 \pi \mathrm{i} n, \quad \forall n \ne 0\,, \end{aligned}$$

whereas the brackets between all other coordinate functions vanish, and which has the property that for any $s \in {\mathbb {N}}$, the restriction of $\Phi ^{kdv}$ to $H^s_0({\mathbb {T}}_1)$ is a real analytic diffeomorphism with range $h^s_0$, $\Phi ^{kdv} : H^s_0({\mathbb {T}}_1) \rightarrow h^s_0$, so that the KdV Hamiltonian, when expressed in the coordinates $w_n,$ $n \ne 0,$ is in normal form. More precisely,

$$\begin{aligned} {H}^{kdv} \circ \Psi ^{kdv} : h^1_0 \rightarrow {\mathbb {R}}\,, \qquad \Psi ^{kdv} := (\Phi ^{kdv})^{- 1}\,, \end{aligned}$$

is a real analytic function ${{{\mathcal {H}}}}^{kdv }$ of the actions $I(w)= (I_n(w))_{n \ge 1}$ alone,

$$\begin{aligned} {{\mathcal {H}}}^{kdv } :\ell ^{1, 3}_+ \rightarrow {\mathbb {R}}, \ I \mapsto {{\mathcal {H}}}^{kdv }(I), \qquad I_n(w) := 2 \pi n w_n w_{- n}, \ \forall n \ge 1, \end{aligned}$$

where $\ell ^{1,3}_+$ denotes the positive quadrant of the weighted $\ell ^1-$sequence space,

$$\begin{aligned} \ell ^{1,3} \equiv \ell ^{1,3}({\mathbb {N}}, {\mathbb {R}}) := \{ I=(I_n)_{n \ge 1} \subset {\mathbb {R}}\, : \, \sum _{n =1}^\infty n^3 |I_n| < \infty \} \, , \qquad {\mathbb {N}}:= {\mathbb {Z}}_{\ge 1}\, . \end{aligned}$$

Equation (1.2), when expressed in the coordinates $w_n$, $n \ne 0$, then takes the form

1.11 $$\begin{aligned} \dot{w}_n = \mathrm{i} \omega _n^{kdv}(I) w_n\,, \qquad \forall n \ne 0, \end{aligned}$$

where $\omega _n^{kdv}(I)$, $n \ne 0$, denote the KdV frequencies

1.12 $$\begin{aligned} \omega _n^{kdv}(I) := \partial _{I_n} {{\mathcal {H}}}^{kdv}(I) \ , \quad \omega _{-n}^{kdv}(I) := - \omega _n^{kdv}(I) , \qquad \forall n \ge 1. \end{aligned}$$

Since by (1.10) the action variables Poisson commute, $\{ I_n, I_m \}$, $\forall n, m \ge 1$, it follows that they are prime integrals of (1.2) and so are the frequencies $\omega _n^{kdv}(I)$, $n \ne 0$. As a consequence, (1.11) can be solved by quadrature. Finally, the differential $d_0 \Phi ^{kdv} : L^2_0({\mathbb {T}}_1) \rightarrow \ell ^2_0$ of $\Phi ^{kdv}$ at $q = 0$ is the Fourier transform (cf. [28], Theorem 9.8)

$$\begin{aligned} {{\mathcal {F}}} : L^2_0({\mathbb {T}}_1) \rightarrow \ell ^2_0, \quad q \mapsto (q_n)_{n \ne 0}, \quad q_n := \int _0^1 q(x) e^{- 2 \pi \mathrm{i} n x}\, d x , \end{aligned}$$

and hence $d_0 \Psi ^{kdv}$ is given by the inverse Fourier transform ${{\mathcal {F}}}^{- 1}$. We remark that the coordinates $w_{\pm n} \equiv w_{\pm n}(q)$, referred to as (complex) Birkhoff coordinates, are related to the (real) Birkhoff coordinates $x_n,$ $y_n$, $n \ge 1$, introduced in [28], by

$$\begin{aligned} x_n = \frac{w_n + w_{-n}}{2 \sqrt{n \pi }} , \quad y_n = \mathrm{i} \frac{w_n - w_{-n}}{2 \sqrt{n \pi }} , \qquad \forall \ n \ge 1\,, \end{aligned}$$

where $\sqrt{\cdot }$ denotes the principal branch of the square root, $\sqrt{\cdot } \equiv \root + \of {\cdot }\,$.

The Birkhoff coordinates are well suited to describe the finite gap solutions of (1.2). For any *finite* subset $S_+ \subseteq {\mathbb {N}}$, let

$$\begin{aligned} S := S_+ \cup (- S_+)\, , \qquad S^\bot := {\mathbb {Z}}{\setminus } (S \cup \{ 0 \})\,. \end{aligned}$$

We denote by $M_S$ the submanifold of $L^2_0({\mathbb {T}}_1)$, given by

$$\begin{aligned} M_S := \big \{ q = \Psi ^{kdv}(w) \ : \ w_n(q) = 0 \ \ \forall \, n \in S^\bot \big \}, \quad \end{aligned}$$

whose elements are referred to as *S*-gap potentials, and by $M_S^o$ the open subset of $M_S$, consisting of the so called proper *S*-gap potentials,

$$\begin{aligned} M_S^o := \{ q \in M_S \ : \, w_n(q) \ne 0 \ \ \forall \, n \in S \}\,. \end{aligned}$$

Note that $M_S$ is contained in $\cap _{s \ge 0} H^s_0({\mathbb {T}}_1)$ and hence consists of $C^\infty$-smooth potentials and that $M_S^o$ can be parametrized by the action-angle coordinates $\theta = (\theta _k)_{k \in S_+} \in {\mathbb {T}}^{S_+},$ and $I = (I_k)_{k \in S_+} \in {\mathbb {R}}^{S_+}_{> 0}$,

$$\begin{aligned} \Psi _{S_+} : {{\mathcal {M}}}_S^o := {\mathbb {T}}^{S_+} \times {\mathbb {R}}^{S_+}_{> 0} \rightarrow M_S^o, \,\, (\theta , I) \mapsto \Psi _{S_+}(\theta , I) := \Psi ^{kdv}(w(\theta , I)) \end{aligned}$$

where ${\mathbb {T}}:= {\mathbb {R}}/ 2 \pi {\mathbb {Z}}$ and $w(\theta , I) = (w_n(\theta , I))_{n \ne 0}$ is defined by

1.13 $$\begin{aligned} w_{\pm n} := \sqrt{ I_n / (2 \pi n)} e^{\mp \mathrm{i} \theta _n}, \quad \forall n \in S_+ , \qquad \quad w_n : = 0, \quad \forall n \in S^\bot \,. \end{aligned}$$

Introduce

$$\begin{aligned} h^s_\bot := \big \{ w \in h^s_{\bot c} : w_{- n} = \overline{w}_n \,\, \forall n \in S^\bot \big \}, \qquad h^s_{\bot c} := h^s(S^\bot , {\mathbb {C}})\,. \end{aligned}$$

For notational convenience, we view ${{\mathcal {M}}}_S^o \times h^s_\bot$ as a subset of $h^s_0$. Its elements are denoted by

$$\begin{aligned} \quad \theta = (\theta _n)_{n \in S_+}, \,\,\, I = (I_n)_{n \in S_+}, \,\,\, w = (w_n)_{n \in S^\bot } \end{aligned}$$

and it is endowed with the canonical Poisson bracket, given by

$$\begin{aligned} \{ I_n, \theta _n \} = 1, \quad \forall n \in S_+, \qquad \{ w_n, w_{- n} \} = \mathrm{i} 2 \pi n, \quad \forall n \in S^\bot _+ := S^\bot \cap {\mathbb {N}}\, , \end{aligned}$$

whereas the brackets between all other coordinate functions vanish.

It is convenient to introduce the frequency vector $\omega (I)$ (cf. (1.12)),

1.14 $$\begin{aligned} \omega (I):= (\omega ^{kdv}_n(I , 0))_{n \in S_+}\,. \end{aligned}$$

By [11], the action to frequency map $\omega : {\mathbb {R}}^{S_+}_{> 0} \rightarrow {\mathbb {R}}^{S_+},$ $I \mapsto \omega (I)$, is a local diffeomorphism. Throughout the paper, we denote by $\Xi \subset {\mathbb {R}}^{S_+}_{> 0}$ the closure of a bounded, open, nonempty set so that the restriction of $\omega$ to $\Xi$ is a diffeomorphism onto its image $\Pi := \omega (\Xi )$ and so that for some $\delta > 0,$

$$\begin{aligned} \Xi + B_{S_+}(\delta ) \subset {\mathbb {R}}^{S_+}_{> 0} , \end{aligned}$$

where $$B_{S_+}(\delta )$$ is the ball in $${\mathbb {R}}^{S_+}$$ of radius $$\delta > 0$$, centered at the origin. We remark that for any $$I \in \Xi + B_{S_+}(\delta )$$, the nth action $$I_n = I_n(w)$$, $$n \in S_+$$, is of the form $$ I_n(w) = I_n^{(0)} +y$$ where $$I_n^{(0)}:= 2 \pi n w^{(0)}_n w^{(0)}_{- n} \in \Xi $$ and

1.15 $$\begin{aligned} y_n = (w_n - w_n^{(0)})w_{-n}^{(0)} + w_n^{(0)} (w_{-n} - w_{-n}^{(0)}) + (w_n - w_n^{(0)})(w_{-n} - w_{-n}^{(0)}) \, . \end{aligned}$$

The inverse of $\omega : \Xi \rightarrow \Pi$ is denoted by $\mu$,

$$\begin{aligned} \mu : \Pi \rightarrow \Xi , \quad \omega \mapsto \mu (\omega )\,. \end{aligned}$$

In what follows, we will consider the frequency vector $\omega$ as a parameter. For any $\omega \in \Pi ,$ a $S-$gap solution of (1.2) is defined as a solution of the form

1.16 $$\begin{aligned} q(t, x; \omega ) = \Psi _{S_+}(\theta ^{(0)} + \omega t, \mu (\omega ))(x) \ , \qquad \theta ^{(0)} \in {\mathbb {T}}^{S_+} , \end{aligned}$$

whereas a finite gap solution of (1.2) is a solution of the form (1.16) for some $S = S_+ \cup (- S_+)$ with $S_+ \subset {\mathbb {N}}$ finite. The $S-$gap solution $t \mapsto q(t, x; \omega )$ is a curve on the $|S_+|-$dimensional torus

$$\begin{aligned} {\mathfrak {T}}_{\mu (\omega )} := \Psi _{S_+}\big ({\mathbb {T}}^{S_+} \times \{\mu (\omega )\} \big ). \end{aligned}$$

We note that ${\mathfrak {T}}_{\mu (\omega )}$ is invariant under (1.2) and Lyapunov stable in $H^s_0({\mathbb {T}}_1)$ for any $s \ge 0$. More precisely, for any $\varepsilon > 0$ there exists $\delta > 0$, depending on *s*, so that for any initial data $u_0 \in H^s_0({\mathbb {T}}_1)$ with

1.17 $$\begin{aligned} \mathrm{dist}_{H^s} \big (u_0, {\mathfrak {T}}_{\mu (\omega )} \big ) \le \delta \ , \qquad \mathrm{dist}_{H^s} \big (u_0, {\mathfrak {T}}_{\mu (\omega )} \big ) := \inf _{q \in {\mathfrak {T}}_{\mu (\omega )} } \Vert u_0 - q\Vert _s \ , \end{aligned}$$

the solution $u(t, \cdot )$ of (1.2) with $u(0, \cdot ) = u_0$ satisfies

$$\begin{aligned} \mathrm{dist}_{H^s} \big (u(t, \cdot ), {\mathfrak {T}}_{\mu (\omega )} \big ) \le \varepsilon \ , \qquad \forall \ t \in {\mathbb {R}}. \end{aligned}$$

Finally, we introduce the so called normal frequencies,

1.18 $$\begin{aligned} \Omega _j(\omega ) := \omega ^{kdv}_j(\mu (\omega ), 0), \quad j \in S^\bot , \ \omega \in \Pi \, , \end{aligned}$$

and for any given $\tau > |S_+|$, the subsets $\Pi _\gamma$ of $\Pi$,

1.19 $$\begin{aligned} \Pi _\gamma := \cap _{i = 0}^3 \Pi _\gamma ^{(i)} , \qquad 0< \gamma <1 \ , \end{aligned}$$

where $\Pi _\gamma ^{(i)}$, $0 \le i \le 3$, are given by

1.20 $$\begin{aligned} \begin{aligned} \Pi _\gamma ^{(0)}&:= \big \{ \omega \in \Pi \ : \ |\omega \cdot \ell | \ge \frac{\gamma }{\langle \ell \rangle ^\tau } \ \ \forall \ell \in {\mathbb {Z}}^{S_+} {\setminus } \{0\}\big \}\,, \\ \Pi ^{(1)}_\gamma&:= \big \{ \omega \in \Pi \ : \ |\omega \cdot \ell + \Omega _j(\omega )| \ge \frac{\gamma }{\langle \ell \rangle ^\tau } \ \ \forall (\ell , j) \in {\mathbb {Z}}^{S_+} \times S^\bot \big \}\,, \\ \Pi _\gamma ^{(2)}&:= \big \{ \omega \in \Pi \ : \ |\omega \cdot \ell + \Omega _{j_1}(\omega ) + \Omega _{j_2}(\omega )| \ge \frac{\gamma }{\langle \ell \rangle ^\tau } \\&\ \ \forall (\ell , j_1, j_2) \in {\mathbb {Z}}^{S_+} \times S^\bot \times S^\bot \ \text {with} \ (\ell , j_1, j_2) \ne (0, j_1, - j_1) \big \}\,, \\ \Pi ^{(3)}_\gamma&:= \big \{ \omega \in \Pi \ : \ |\omega \cdot \ell + \Omega _{j_1}(\omega ) + \Omega _{j_2}(\omega ) + \Omega _{j_3}(\omega )| \ge \frac{\gamma }{\langle \ell \rangle ^\tau \langle j_1 \rangle ^2 \langle j_2 \rangle ^2 \langle j_3 \rangle ^2} \\&\ \ \forall (\ell , j_1, j_2, j_3) \in {\mathbb {Z}}^{S_+} \times S^\bot \times S^\bot \times S^\bot \ \text {with} \ j_k + j_m \ne 0 \ \ \forall k, m \in \{1,2,3\} \big \}\,. \end{aligned} \end{aligned}$$

Here we used the standard notation for vectors *y* in ${\mathbb {R}}^n$,

1.21 $$\begin{aligned} \langle y \rangle := \max \{1, | y | \} , \quad | y | := (\sum _{j=1}^n |y_j|^2)^{1/2} , \qquad \forall \, y \in {\mathbb {R}}^n \, . \end{aligned}$$

We refer to $\Pi ^{(j)}_\gamma$, $0 \le j \le 3$, as the *jth Melnikov conditions* and note that the third Melnikov conditions allow for ’a loss of derivatives in space’—see item (*ii*) in *Comments on Theorem* 1.1 below.

The goal of this paper is to prove a long time stability result of finite gap solutions (1.16) of the Korteweg-de Vries equation on ${\mathbb {T}}_1$. To state it, we denote for any Banach space *X* with norm $\Vert \cdot \Vert _X$, integer $m \ge 0$, and interval $J \subset {\mathbb {R}}$, by $C^m(J, X)$ the Banach space of functions $f: J \rightarrow X$, which are *m* times continuously differentiable, endowed with the supremum norm, $\Vert f\Vert _{C^m_t} := \max _{0 \le j \le m} \sup \{ \Vert \partial _t^j f(t) \Vert _X \, : \, t \in J; 0 \le j \le m\}$.

### Theorem 1.1

Let *f* be a function in $C^{\infty }({\mathbb {T}}_1 \times {\mathbb {R}})$ (cf. (1.6)), $S_+$ be a finite subset of ${\mathbb {N}}$, and $\tau$ be a number with $\tau > |S_+|$ (cf. (1.20)). Then for any integer *s* sufficiently large and any $\omega \in \Pi _\gamma$, $0< \gamma < 1$, there exists $0< \varepsilon _0 \equiv \varepsilon _0(s, \gamma ) < 1$ with the following properties: for any $0 < \varepsilon \le \varepsilon _0$ and any initial data $u_0 \in H^s_0({\mathbb {T}}_1)$, satisfying

1.22 $$\begin{aligned} \mathrm{dist}_{H^s} \big (u_0, {\mathfrak {T}}_{\mu (\omega )} \big ) \le \varepsilon \ , \end{aligned}$$

equation (1.4) admits a unique solution

$$\begin{aligned} t \mapsto u(t, \cdot ) in C^0([-T, T], H^s_0({\mathbb {T}}_1)) \cap C^1([-T, T], H^{s-3}_0({\mathbb {T}}_1)) \end{aligned}$$

with initial data $u(0, x) = u_0(x)$ and $T \equiv T_{\varepsilon , s, \gamma } = O(\varepsilon ^{- 2})$. Moreover, *u* satisfies the estimate

$$\begin{aligned} \mathrm{dist}_{H^s} \big (u(t, \cdot ), {\mathfrak {T}}_{\mu (\omega )} \big ) \lesssim _{s, \gamma } \ \varepsilon \, , \qquad \forall \ - T \le t \le T \ , \end{aligned}$$

where the distance function $\mathrm{dist}_{H^s}$ is defined in (1.17). Furthermore, there exists $0< \mathtt {a} < 1$ so that for any $0< \gamma < 1,$ the Lebesgue measure $|\Pi {\setminus } \Pi _\gamma |$ of $\Pi {\setminus } \Pi _\gamma$ satisfies

1.23 $$\begin{aligned} |\Pi {\setminus } \Pi _\gamma | \lesssim \gamma ^{\mathtt {a}} \ , \quad \mathrm{implying \ that } \quad \lim _{\gamma \rightarrow 0}|\Pi _\gamma | = |\Pi | \ . \end{aligned}$$

Here and in the sequel, the notation $h \lesssim _{\alpha , \ldots } g$ means that the real valued function *h*, depending on various variables, satisfies an estimate of the form $h \le C g$ where *g* is also a real valued function, typically small, and the constant $C>0$ only depends on the parameters $\alpha , \ldots$. For notational convenience, the dependence of the constant *C* on *f*, $S_+$, and $\tau$ is not indicated.

**Comments on Theorem** 1.1

(*i*): *Initial data.* Note that the size of the distance of the initial value $u_0$ to the considered $S-$gap solution of the KdV equation (cf. (1.22)) is assumed to be of the same order of magnitude as the size of the perturbation $\varepsilon F(u)$ in (1.4).
(*ii*): *Measure estimate* (1.23). The proof of the measure estimates (1.23) requires that the third Melnikov conditions $\Pi ^{(3)}_\gamma$ in (1.20) allow for a loss of derivatives in space. Furthermore, a key ingredient into the proof of (1.23) is the case $n=3$ of Fermat’s Last Theorem, proved by Euler [21] (cf. Lemma 8.3).
(*iii*): *Assumptions in Theorem*  1.1. The results of Theorem 1.1 hold for any density $f(x, \zeta )$ of class ${\mathcal {C}}^{\sigma }$ with $\sigma$ sufficiently large. Furthermore, corresponding results hold for (invariant tori of) finite gap solutions of the KdV equation in the affine spaces $c + H^s_0({\mathbb {T}}_1)$, $c \in {\mathbb {R}}$. We assume in this paper that *f* is ${\mathcal {C}}^\infty -$smooth and that $c=0$ merely to simplify the exposition. In order to limit the size of the paper, we assume the perturbation $\varepsilon F(u)$ to be semilinear (cf. (1.5)), leaving the case of a quasilinear one for future work. Most likely, the elaborate method designed in [23] will allow to transform quasilinear perturbations into normal form while preserving the Hamiltonian structure of the equation.
(*iv*): *Time of stability.* It seems unlikely that the stability results of Theorem 1.1 in the generality stated are valid for time intervals of size larger than $O(\varepsilon ^{-2})$ since the conditions, required to hold for the frequencies $\Omega _j$, $j \in S^\bot$, so that the normal form procedure could be implemented, are too strong. See Remark 8.1 at the end of Sect. 8. Actually, it might be possible that the (almost) resonances of the KdV frequencies of degree four can be used to prove instability results for solutions of the perturbed equation (1.4)—see [16, 25] and references therein for related results for Schrödinger equations in two space dimension.
(*v*): *Conservation of momentum.* If the density *f* of the perturbation ${P_f}(u) = \int _0^1 f(x, u(x))\, d x$ does not explicitely depend on *x*, then the momentum $M(u) := \frac{1}{2} \int _{{\mathbb {T}}_1} u^2\, d x$ is a prime integral of Eq. (1.4). We plan to prove in future work that the stability time can be improved in such a case.
(*v*): *Integrable PDEs.* The method of proof of Theorem 1.1 is quite general. We expect that for any integrable PDE, admitting coordinates of the type constructed in [27], a corresponding version of Theorem 1.1 holds, up to the measure estimates related to the nonresonance conditions for the frequencies of the integrable PDE considered. These estimates might require specific arithmetic properties of the frequencies—see item (*ii*) above.

To explain the main ideas of the proof, we first need to introduce some terminology and additional notations. They will be used throughout the paper.

*Notations and terminology.* For any finite subset $S_+ \subset {\mathbb {N}}$, $L^2_\bot ({\mathbb {T}}_1)$ is the subspace, given by

1.24 $$\begin{aligned} L^2_\bot ({\mathbb {T}}_1) := \big \{ w = \sum _{n \in S^\bot } w_n e^{\mathrm{i} 2\pi n x} \in L^2_0 ({\mathbb {T}}_1) \big \}\, , \qquad S^\bot = {\mathbb {Z}}{\setminus } \big ( S_+ \cup (- S_+) \cup \{ 0 \} \big ) \, , \end{aligned}$$

and $\Pi _\bot$ denotes the $L^2-$orthogonal projector onto the subspace $L^2_\bot ({\mathbb {T}}_1)$. For any $s > 0$, we set

1.25 $$\begin{aligned} H^s_\bot ({\mathbb {T}}_1) := H^s({\mathbb {T}}_1) \cap L^2_\bot ({\mathbb {T}}_1), \qquad H_\bot ^0({\mathbb {T}}_1) := L^2_\bot ( {\mathbb {T}}_1)\, . \end{aligned}$$

By ${{\mathcal {E}}}_s$ we denote the phase space and by $E_s$ the corresponding tangent space, given by

1.26 $$\begin{aligned} {{\mathcal {E}}}_s := {\mathbb {T}}^{S_+} \times {\mathbb {R}}^{S_+} \times H^s_\bot ({\mathbb {T}}_1)\,, \quad {{\mathcal {E}}} \equiv {{\mathcal {E}}}_0 \, , \quad E_s := {\mathbb {R}}^{S_+} \times {\mathbb {R}}^{S_+} \times H^s_\bot ({\mathbb {T}}_1)\,, \quad E \equiv E_0\, ,\nonumber \\ \end{aligned}$$

where $${\mathbb {T}}_1 = {\mathbb {R}}/ {\mathbb {Z}}$$ and $${\mathbb {T}}= {\mathbb {R}}/ 2\pi {\mathbb {Z}}$$. Elements of $${{\mathcal {E}}}$$ are denoted by $${\mathfrak {x}} = (\theta , y , w)$$ and the ones of its tangent space *E* by $$ \widehat{ {\mathfrak {x}}} = ({{\widehat{\theta }}}, {\widehat{y}},{\widehat{w}})$$. For $$s > 0$$, $$ H^{s}_\bot ({\mathbb {T}}_1)^*$$ denotes the dual space of $$ H^{s}_\bot ({\mathbb {T}}_1)$$, which is canonically identified with the Sobolev space $$ H^{-s}_\bot ({\mathbb {T}}_1)$$ of distributions. The spaces $$ {{\mathcal {E}}}_{-s} $$ and $$ E_{-s}$$ are then defined as in (1.26). On *E*, we denote by $$ \langle \cdot , \cdot \rangle _E$$ the inner product defined by

1.27 $$\begin{aligned} \big \langle ({\widehat{\theta }}_1, {\widehat{y}}_1, {\widehat{w}}_1), ({\widehat{\theta }}_2, {\widehat{y}}_2, {\widehat{w}}_2) \big \rangle _E := {\widehat{\theta }}_1 \cdot {\widehat{\theta }}_2 + {\widehat{y}}_1 \cdot {\widehat{y}}_2 + \big \langle {\widehat{w}}_1, {\widehat{w}}_2 \big \rangle \, \end{aligned}$$

where $\langle \cdot , \cdot \rangle$ is the standard real scalar product on $L^2_\bot$. For notational convenience, $\Pi _\bot$ also denotes the projector of $E_s$ onto its third component,

$$\begin{aligned} \Pi _\bot : E_s \rightarrow H^s_\bot ({\mathbb {T}}_1) \, , \, \quad ({\widehat{\theta }}, {\widehat{y}}, {\widehat{w}}) \mapsto {\widehat{w}}\, . \end{aligned}$$

For any $0< \delta < 1$, we denote by $B_{S_+}(\delta )$ the open ball in ${\mathbb {R}}^{S_+}$ of radius $\delta$ centered at 0 and by $B_\bot ^s(\delta )$, $s \ge 0$, the corresponding one in $H^s_\bot ({\mathbb {T}}_1)$. For $s=0$, we also write $B_\bot (\delta )$ instead of $B^0_\bot (\delta )$. These balls are used to define the following open neighborhoods in ${\mathcal {E}}_s$, $s \ge 0$,

1.28 $$\begin{aligned} {{\mathcal {V}}}^s(\delta ) := {\mathbb {T}}^{S_+}_1 \times B_{S_+}(\delta ) \times B_\bot ^s(\delta ) \,, \qquad {{\mathcal {V}}}(\delta ) \equiv {{\mathcal {V}}}^0(\delta ) \, , \qquad 0< \delta < 1\, . \end{aligned}$$

For notational convenience, often without stating it explicitly, $\delta > 0$ will take on different values in the course of our arguments. In particular, $\delta > 0$ typically will depend on *s*. (Note that by (1.15), the coordinates $y= (y_n)_{n \in S_+}$ are of the same order as the coordinates $w= (w_n)_{n \in S^\bot }$.)

For any $k \ge 1,$ $\partial _x^{-k} : L^2({\mathbb {T}}_1) \rightarrow L^2_0({\mathbb {T}}_1)$ is the linear operator, defined by

$$\begin{aligned} \partial _x^{-k}[e^{2\pi \mathrm{i} nx}] = \frac{1}{(2\pi \mathrm{i} n)^k} e^{2\pi \mathrm{i} nx}\, , \quad \forall n \ne 0\,, \qquad \text{ and } \qquad \partial _x^{-k}[1] = 0\,. \end{aligned}$$

The space ${{\mathcal {V}}}^s(\delta )$ is endowed with the symplectic form

1.29 $$\begin{aligned} {{\mathcal {W}}} := \big ( {\mathop \sum }_{j \in S_+} d y_j \wedge d \theta _j \big ) \oplus {{\mathcal {W}}}_\bot \end{aligned}$$

where ${{\mathcal {W}}}_\bot$ is the restriction to $L^2_\bot ({\mathbb {T}}_1)$ of the symplectic form ${{\mathcal {W}}}_{L^2_0}$ defined in (1.9). Throughout the paper, the Hamiltonians considered depend on the small parameter $\varepsilon \in [0, \varepsilon _0]$, $0< \varepsilon _0 < 1$, and are $C^\infty$-smooth maps, ${{\mathcal {V}}}^s(\delta ) \times [0, \varepsilon _0] \rightarrow {\mathbb {R}}$. Given such a Hamiltonian *H*, we often do not indicate the dependence of *H* on the parameter $\varepsilon$. The Hamiltonian vector field of *H* is denoted by $X_H$. It is given by

1.30 $$\begin{aligned} X_H ({\mathfrak {x}}) = {{\mathcal {J}}} \nabla H ({\mathfrak {x}}) = \big ( - \nabla _y H({\mathfrak {x}}), \, \nabla _\theta H({\mathfrak {x}}), \, \partial _x \nabla _\bot H({\mathfrak {x}}) \big ) \end{aligned}$$

where ${\mathcal {J}}$ is the Poisson structure, associated to the symplectic form ${\mathcal {W}}$,

1.31 $$\begin{aligned} {\mathcal {J}} : E_{s } \rightarrow E_{s - 1} \, , \quad ({\widehat{\theta }}, {\widehat{y}}, {\widehat{w}}) \mapsto (- {\widehat{y}}, {\widehat{\theta }}, \partial _x {\widehat{w}}) \, \end{aligned}$$

and where $\nabla _\bot H({\mathfrak {x}}) \equiv \nabla _w H({\mathfrak {x}})$ denotes the $L^2-$gradient of *H* with respect to the variable *w*. For notational convenience, we denote by $\{ F, G \}$ the Poisson bracket corresponding to ${\mathcal {J}},$

1.32 $$\begin{aligned} \{ F, G \}= & {} {{\mathcal {W}}}(X_F, X_G) = \big \langle \nabla F \,,\, {{\mathcal {J}}} \nabla G \big \rangle _E \nonumber \\= & {} - \nabla _\theta F \cdot \nabla _y G + \nabla _y F \cdot \nabla _\theta G + \big \langle \nabla _\bot F\,,\, \partial _x \nabla _\bot G \big \rangle \,. \end{aligned}$$

Given a Hamiltonian vector field $X_F : {{\mathcal {V}}}^s(\delta ) \times [0, \varepsilon _0] \rightarrow E_s$ with Hamiltonian *F*, we denote by $\Phi _F(\tau , \cdot )$ or $\Phi _{X_F}(\tau , \cdot )$ the flow generated by $X_F$. For the vector fields $X_F$ considered in this paper, there exists $0< \delta ' < \delta$ so that for any $\tau \in [- 1, 1]$, the flow map ${\mathcal {V}}^s(\delta ') \rightarrow {\mathcal {V}}^s(\delta )$, ${\mathfrak {x}} \mapsto \Phi _F(\tau , {\mathfrak {x}})$ is well defined. The Taylor expansion of $\tau \mapsto H \circ \Phi _F(\tau , {\mathfrak {x}})$ at $\tau = 0$ can be computed as

1.33 $$\begin{aligned} H \circ \Phi _F(\tau , {\mathfrak {x}}) = H({\mathfrak {x}}) + \tau \{ H, F \}({\mathfrak {x}}) + \tau ^2 \int _0^1 (1 - t) \{ \{H, F \}, F \} \circ \Phi _F(t\tau , {\mathfrak {x}})\, d t\,. \end{aligned}$$

We will also need to consider $C^\infty$-smooth vector fields, which are not necessarily Hamiltonian,

$$\begin{aligned} X = (X^{(\theta )}, \, X^{(y)}, \, X^\bot ) : {{\mathcal {V}}}^s(\delta ) \times [0, \varepsilon _0] \rightarrow E_s\, , \end{aligned}$$

where $X^{(\theta )}$, $X^{(y)}$, and $X^\bot$ are the components of *X*,

$$\begin{aligned} X^{(\theta )}, \ X^{(y)} : {{\mathcal {V}}}^s(\delta ) \times [0, \varepsilon _0] \rightarrow {\mathbb {R}}^{S_+} \, , \qquad X^\bot : {{\mathcal {V}}}^s(\delta ) \times [0, \varepsilon _0] \rightarrow H^s_\bot ({\mathbb {T}}_1)\,. \end{aligned}$$

The corresponding flow is denoted by $\Phi _X(\tau , \cdot )$. Again we will only consider vector fields *X* with the property that there exists $0< \delta ' < \delta$ so that for any $\tau \in [- 1, 1]$, $\Phi _X(\tau , \cdot )$ is well defined on ${{\mathcal {V}}}^s(\delta ')$. Given two $C^\infty$-smooth vector fields $X, Y : {{\mathcal {V}}}^s(\delta ) \times [0, \varepsilon _0] \rightarrow E_s$, the commutator [*X*, *Y*] is defined as

1.34 $$\begin{aligned} {[}X, Y]({\mathfrak {x}}) := d X({\mathfrak {x}})[Y({\mathfrak {x}})] - d Y({\mathfrak {x}})[X({\mathfrak {x}})]\,. \end{aligned}$$

The pull-back of a vector field $X : {{\mathcal {V}}}^s(\delta ) \rightarrow E_s$ by a $C^\infty$-smooth diffeomorphism $\Phi : {{\mathcal {V}}}^s(\delta ') \rightarrow {{\mathcal {V}}}^s(\delta )$ is defined as,

1.35 $$\begin{aligned} \Phi ^* X({\mathfrak {x}}) := d \Phi ({\mathfrak {x}})^{ - 1} X(\Phi ({\mathfrak {x}}))\, , \qquad \forall {\mathfrak {x}} \in {{\mathcal {V}}}^s(\delta ')\, . \end{aligned}$$

If $\Phi _\tau (\cdot ) \equiv \Phi _Y(\tau , \cdot )$ is the flow of a vector field *Y*, then the Taylor expansion of $\tau \mapsto \Phi _\tau ^* X ({\mathfrak {x}})$ at $\tau = 0$ reads

1.36 $$\begin{aligned} \Phi _\tau ^* X ({\mathfrak {x}})&= X({\mathfrak {x}}) + \tau \int _0^1 (d \Phi (t\tau , {\mathfrak {x}}))^{- 1}[X, Y] (\Phi (t \tau , {\mathfrak {x}}))\, d t \nonumber \\&= X({\mathfrak {x}}) + \tau [X, Y]({\mathfrak {x}}) + \tau ^2 \int _0^1 (1 - t) (d \Phi (t\tau , {\mathfrak {x}}))^{- 1}[[X, Y], Y] (\Phi (t \tau , {\mathfrak {x}}))\, d t \,. \end{aligned}$$

In the case $\tau =1$, we will often write $\Phi _Y^*X$ instead of $\Phi _1^* X$. Clearly if $X = X_H$, $Y = Y_F$ are Hamiltonian vector fields, then

$$\begin{aligned} {[}X, Y] = X_{\{ H, F\}}, \quad (\Phi _Y(\tau , \cdot ))^* X = X_{H \circ \Phi _Y(\tau , \cdot )}\,. \end{aligned}$$

Given two linear operators *A*, *B*, acting on $L^2({\mathbb {T}}_1)$ (or $L^2_\bot ({\mathbb {T}}_1)$), their commutator is conveniently denoted by $[A, B]_{lin}$,

1.37 $$\begin{aligned} {[}A, B]_{lin} = A B - B A\, . \end{aligned}$$

Moreover, given a densely defined linear operator $A : L^2_\bot (T_1) \rightarrow L^2_\bot ({\mathbb {T}}_1)$, whose domain contains the elements of the Fourier basis $e^{\mathrm{i} 2 \pi j x}$, $j \in S^\bot$, we denote by $A_j^{j'}$ or $[A]_j^{j'}$ the (Fourier) matrix coefficients of *A*,

$$\begin{aligned} A_j^{j'} := \int _0^1 A[e^{\mathrm{i} 2 \pi j' x}] e^{- \mathrm{i} 2 \pi j x}\, dx , \qquad j, j' \in S^\bot \,. \end{aligned}$$

Given a Banach space $(X, \Vert \cdot \Vert _X)$, we denote by $C^\infty _b ({{\mathcal {V}}}^s(\delta ) \times [0, \varepsilon _0], X)$ the space of $C^\infty$ functions ${{\mathcal {V}}}^s(\delta ) \times [0, \varepsilon _0] \rightarrow X$ with all derivatives bounded.

In our normal form procedure, we need to take into account the order of vanishing with respect to the variables *y*, *w* and the small parameter $\varepsilon$. The following definition turns out to be convenient.

### Definition 1.1

Let $(B, \Vert \cdot \Vert _B)$ be a Banach space and $p \in {\mathbb {Z}}_{\ge 0}$. A $C^\infty$-smooth map

$$\begin{aligned} g : {{\mathcal {V}}}^{s}(\delta ) \times [0, \varepsilon _0] \rightarrow B, \ ({\mathfrak {x}}, \varepsilon ) \mapsto g({\mathfrak {x}}, \varepsilon ) \end{aligned}$$

is said to be small of order *p* if for any $\beta \in {\mathbb {Z}}_{\ge 0}^{S_+}$ and $k_1, k_2 \in {\mathbb {Z}}_{\ge 0}$ with $|\beta | + k_1 + k_2 \le p - 1$

1.38 $$\begin{aligned} d^{k_2}_\bot \partial _y^\beta \partial _\varepsilon ^{k_1} g(\theta , 0, 0, 0)= 0\, , \qquad \forall \, \theta \in {\mathbb {T}}^{S_+}\, . \end{aligned}$$

Note that if *g* is small of order *p*, then

$$\begin{aligned} \Vert g ( {\mathfrak {x}}, \varepsilon ) \Vert _B \lesssim _{g} ( |y| + \Vert w \Vert _s + \varepsilon )^p\, , \qquad \forall \, {\mathfrak {x}} = (\theta , y, w) \in {{\mathcal {V}}}^s(\delta ), \ \forall \, \varepsilon \in [0, \varepsilon _0]\, , \end{aligned}$$

and for any $\alpha \in {\mathbb {Z}}_{\ge 0}^{S_+}$, $\partial _\theta ^\alpha g$ is small of order *p* as well.

Given two Banach spaces $(X, \Vert \cdot \Vert _X)$, $(Y, \Vert \cdot \Vert _Y)$, we denote by ${{\mathcal {B}}}(X, Y)$ the space of bounded linear operators $X \rightarrow Y$. If $X = Y$, we write ${{\mathcal {B}}}(X)$ instead of ${{\mathcal {B}}}(X, X)$. Moreover for any integer $p \ge 2$, we denote by ${{\mathcal {B}}}_p(X, Y)$, the space of bounded, *p*-multilinear maps $M : X^p \rightarrow Y$, equipped with the standard norm,

1.39 $$\begin{aligned} \Vert M\Vert _{{{\mathcal {B}}}_p(X, Y)} := \sup _{\Vert u_1 \Vert _{X}, \ldots , \Vert u_p \Vert _X \le 1} \Vert M[u_1, \ldots , u_p] \Vert _Y\,, \quad M \in {{\mathcal {B}}}_p(X, Y)\,. \end{aligned}$$

If $X = Y$, we write ${{\mathcal {B}}}_p(X)$ instead of ${{\mathcal {B}}}_p(X, X)$. Furthermore, given open sets $U \subset X$ and $V \subset Y$, we denote by $C^\infty _b \big (U, V \big )$ the space of maps $f: U \rightarrow V$ which are $C^\infty$-smooth and together with each of its derivatives, bounded.

*Overview of the proof of Theorem*  1.1. We prove Theorem 1.1 by the means of a normal form procedure. A key ingredient are canonical coordinates near a torus ${\mathfrak {T}}_{\mu (\omega )}$ of arbitrary size, constructed in [27]. They are obtained by first linearizing the Birkhoff map $\Phi ^{kdv}$ at ${\mathfrak {T}}_{\mu (\omega )}$ and then constructing a symplectic corrector. The new coordinates yield a family of canonical transformations $\Phi ^{kdv}_{\mu }$, parametrized by $\mu \equiv \mu (\omega )$, $\omega \in \Pi$. One of the main features of these transformations is that they admit expansions in terms of pseudo-differential operators up to a remainder of arbitrary negative order. To prove Theorem  1.1 we then follow a strategy developed in [7] in the context of water waves.

In a first step, referred to as Step 1, we write the perturbed Hamiltonian $H^{kdv} + \varepsilon P_f$ in the new coordinates (cf. Theorem 4.1). More precisely, in Theorem 4.1, we rephrase [27, Theorem 1.1] in a form taylored to our needs and in Corollary 4.1, we compute for any given $\mu \equiv \mu (\omega )$, $\omega \in \Pi$, and ${\mathfrak {x}} = (\theta , y, w) \in {\mathcal {V}}^1(\delta )$ the Taylor expansion of ${{\mathcal {H}}}_{\varepsilon , \mu } := (H^{kdv} + \varepsilon P_f) \circ \Phi ^{kdv}_\mu$ at $(\theta , 0, 0)$ up to order three in the variables *y*, *w*, and $\varepsilon$,

1.40 $$\begin{aligned}&{{\mathcal {H}}}_{\varepsilon , \mu }(\theta , y, w) = {{\mathcal {N}}}_\mu (y, w) + {{\mathcal {P}}}_{\varepsilon , \mu }(\theta , y, w) \,, \end{aligned}$$

1.41 $$\begin{aligned}&{{\mathcal {N}}}_\mu ( y, w) := \omega \cdot y + \frac{1}{2} \Omega _{S_+}(\omega ) [y] \cdot y + \frac{1}{2} \big \langle D_\bot ^{- 1} \Omega _\bot (\omega ) w\,,\, w \big \rangle \, , \end{aligned}$$

where $\Omega _{S_+}(\omega )$ is given by the $S_+ \times S_+$ matrix $(\partial _{I_j} \omega _i^{kdv}(\mu , 0))_{i, j \in S_+}$ and where $D^{- 1}_\bot : L^2_\bot ({\mathbb {T}}_1) \rightarrow L^2_\bot ({\mathbb {T}}_1)$ and $\Omega _\bot (\omega ) \equiv \Omega _{S^\bot }(\omega ): \, L^2_\bot ({\mathbb {T}}_1) \rightarrow L^2_\bot ({\mathbb {T}}_1)$ are Fourier multipliers in diagonal form,

1.42 $$\begin{aligned} D^{- 1}_\bot [w] : = \sum _{n \in S^\bot } \frac{1}{2 \pi n} w_n e^{\mathrm{i} 2\pi n x} \, , \qquad \Omega _\bot (\omega )[w] := \sum _{n \in S^\bot } \Omega _n(\omega ) w_n e^{\mathrm{i} 2\pi n x},\qquad \end{aligned}$$

with $\Omega _n(\omega )$ given by (1.18). In order to simplify notation, in the sequel, we often will not indicate the dependence of quantities such as ${\mathcal {H}}_{\varepsilon , \mu }$, ${\mathcal {P}}_{\varepsilon , \mu }$, $\Omega _\bot (\omega )$, $\ldots$ on $\varepsilon$, $\mu \equiv \mu (\omega )$, and $\omega$.

We note that $\Omega _\bot$ is an unbounded operator. For any ${\mathfrak {x}} = (\theta , y, w)$, ${{\mathcal {P}}}({\mathfrak {x}})$ can be expanded as

1.43 $$\begin{aligned} {{\mathcal {P}}}({\mathfrak {x}}) = \varepsilon \big ( {{\mathcal {P}}}_{00}(\theta ) + {{\mathcal {P}}}_{1 0}(\theta ) \cdot y + \langle {{\mathcal {P}}}_{0 1}(\theta ), w \rangle \big ) + {{\mathcal {P}}}_{e}({\mathfrak {x}}) \, , \end{aligned}$$

where ${{\mathcal {P}}}_{e}({\mathfrak {x}})$ is small of order three (cf. Definition (1.1)). The Hamiltonian vector field $X_{{\mathcal {H}}}$, associated to ${{\mathcal {H}}}$, is given at any point ${\mathfrak {x}} = (\theta , y, w)$ by

1.44 $$\begin{aligned} X_{{\mathcal {H}}} ({\mathfrak {x}})= \begin{pmatrix} - \nabla _y {{\mathcal {H}}} ({\mathfrak {x}})\\ \nabla _\theta {{\mathcal {H}}}({\mathfrak {x}}) \\ \partial _x \nabla _\bot {{\mathcal {H}}}({\mathfrak {x}}) \end{pmatrix} = \begin{pmatrix} - \omega - \Omega _{S_+ }[ y] - \varepsilon {{\mathcal {P}}}_{1 0}(\theta ) - \nabla _y {{\mathcal {P}}}_{e}({\mathfrak {x}}) \\ \varepsilon \nabla _\theta \big ( {{\mathcal {P}}}_{00}(\theta ) + {{\mathcal {P}}}_{1 0}(\theta ) \cdot y + \langle {{\mathcal {P}}}_{0 1}(\theta ), w \rangle \big ) + \nabla _\theta {{\mathcal {P}}}_{e}({\mathfrak {x}}) \\ \mathrm{i} \Omega _\bot w + \varepsilon \partial _x {{\mathcal {P}}}_{0 1}(\theta ) + \partial _x \nabla _\bot {{\mathcal {P}}}_{e} ({\mathfrak {x}}) \end{pmatrix}.\nonumber \\ \end{aligned}$$

We also show that the normal component $\partial _x \nabla _\bot {{\mathcal {P}}}_{e}$ of the Hamiltonian vector field $X_{{\mathcal {P}}_{e}}$ is the sum of a para-differential vector field of order one (cf. Definition 3.1 in Sect. 3) and a smoothing vector field (cf. Definition 3.3 in Sect. 3), i.e., for ${\mathfrak {x}} = (\theta , y, w)$,

1.45 $$\begin{aligned} \partial _x \nabla _\bot {{\mathcal {P}}}_{e}({\mathfrak {x}}) = \Pi _\bot \sum _{k = 0}^{N+1} T_{a_{1 - k}({\mathfrak {x}})} \partial _x^{1 - k} w + {{\mathcal {R}}}^\bot _N({\mathfrak {x}}) \, , \end{aligned}$$

where for any $0 \le k \le N + 1$, $T_{a_{1 - k}({\mathfrak {x}})}$ is the operator of para-multiplication with $a_{1 - k}({\mathfrak {x}}) \in H^s({\mathbb {T}}_1)$ (cf. (2.1) in Sect. 2), which is small of order one, and where ${{\mathcal {R}}}^\bot _{N}({\mathfrak {x}})$ is a regularizing vector field, which is small of order two.

In Step 2, we apply a regularization procedure, which conjugates the vector field (1.44) to another one, which is a smoothing perturbation of a vector field in diagonal form. Since the torus ${\mathfrak {T}}_{\mu (\omega )}$ in the coordinates $(\theta , y, w)$ is described by $\{ y = 0, w = 0 \}$, the variables *y*, *w* can be used to measure the distance of a solution of the equation

1.46 $$\begin{aligned} {\left\{ \begin{array}{ll} \partial _t \theta = - \nabla _y {{\mathcal {H}}} \\ \partial _t y = \nabla _\theta {{\mathcal {H}}} \\ \partial _t w = \partial _x \nabla _\bot {{\mathcal {H}}} \end{array}\right. } \end{aligned}$$

from ${\mathfrak {T}}_{\mu (\omega )}$. Theorem 1.1 follows from Theorem 4.2 in Sect. 4, which states that for $\mu$ in a large subset of $\Xi$ and for any initial data ${\mathfrak {x}}_0 = (\theta _0, y_0, w_0)$, satisfying $|y_0|, \Vert w_0 \Vert _s \le \varepsilon$ with $s > 0$ large enough, the solution $t \mapsto {\mathfrak {x}}(t) = (\theta (t), y(t), w(t))$ of (1.46) exists on a time interval of the form $[- T, T]$ with $T \equiv T_{\varepsilon , s, \gamma } = O(\varepsilon ^{- 2})$ and

$$\begin{aligned} |y(t)|, \, \Vert w(t) \Vert _s \lesssim _{s, \gamma } \varepsilon , \quad \forall t \in [- T, T]\,. \end{aligned}$$

We deduce Theorem 4.2 from Theorem 4.3 and a local existence Theorem (cf. Appendix C), using energy estimates (cf. Sect. 7). Theorem 4.3 provides coordinates having the property that the vector field in (1.46), when expressed in these coordinates, is a vector field $X = (X^{(\theta )}, X^{(y)}, X^{\bot })$ with the following two features: (F1) The *y*-component $X^{(y)}$ of *X* is small of order three. (F2) The normal component $X^\bot ({\mathfrak {x}})$ of $X({\mathfrak {x}})$ at ${\mathfrak {x}} = (\theta , y, w)$ reads

1.47 $$\begin{aligned} X^\bot ({\mathfrak {x}}) = \mathrm{i} \Omega _\bot w + {\mathtt {D}}^\bot ({\mathfrak {x}})[w] + \Pi _\bot T_{a({\mathfrak {x}})} \partial _x w + {{\mathcal {R}}}^\bot ({\mathfrak {x}})\, , \end{aligned}$$

where ${\mathtt {D}}^\bot ({\mathfrak {x}})$ is a skew-adjoint Fourier multiplier of order one (depending nonlinearly on ${\mathfrak {x}}$), $a({\mathfrak {x}}) \in H^s({\mathbb {T}}_1)$ is small of order two, and the remainder ${{\mathcal {R}}}^\bot ({\mathfrak {x}})$ is small of order three. In broad terms, our normal form procedure *diagonalizes* the normal component $X^\bot$ of the vector field *X* up to a term, which is small of order three and which can be controlled by energy estimates. The procedure consists in *eliminating*/*normalizing* the terms of the Taylor expansion (1.40)–(1.43) of $X_{{\mathcal {H}}}$, which are *p*-homogeneous in *y*, *w*, $\varepsilon$ with $0 \le p \le 2$ (cf. Definition 1.1).

Based on the normal form procedure, developed in Sects. 5 and 6, Theorem 4.3 is proved in Sect. 7. In Sect. 8 we show that the Lebesgue measure $|\Pi {\setminus } \Pi _\gamma |$ of $\Pi {\setminus } \Pi _\gamma$ (cf. (1.20)) satisfies $|\Pi {\setminus } \Pi _\gamma | \lesssim \gamma ^{\mathtt {a}}$ for some $0< \mathtt {a} < 1$. As already mentioned in item (ii) of *Comments on Theorem* 1.1, a key ingredient of the proof is the case $n=3$ of Fermat’s Last Theorem, proved by Euler [21] (cf. Lemma 8.3). Sections 2 and 3 are prelimimary where para-differential calculus and para-differential vector fields are discussed to the extent needed in the paper.

We finish our overview of the proof of Theorem  1.1 by describing in some more detail the normal form procedure, developed in Sects. 5–6, to prove Theorem 4.3. In order to setup such a procedure in an effective way, we introduce, in the spirit of [7, 18, 23], various classes of para-differential and smoothing vector fields, which possibly depend in a nonlinear fashion on ${\mathfrak {x}} = (\theta , y, w)$, and develop a symbolic calculus for them—see Sect. 3. The order of homogeneity in our symbol classes is computed with respect to *y*, *w*, $\varepsilon$ where we recall that *y*, *w* (together with $\theta$) are phase space variables and $\varepsilon$ is the perturbation parameter appearing in (1.4) and (1.22). Our normal form procedure is split into two steps which we now describe.

In a first step, presented in Sect. 5, we normalize the terms in the Taylor expansion of the Hamiltonian ${{\mathcal {H}}}$, which are linear with respect to the normal variable *w* and homogeneous of order at most three in $(y, w, \varepsilon )$. Equivalently, this means that we normalize the terms in the Taylor expansion of the Hamiltonian vector field $X_{{{\mathcal {H}}}}$ which do not contain *w* and are homogeneous of order at most two. This is achieved by a *standard normal form procedure* which consists in constructing a canonical transformation, given by the time one flow map $\Phi _{\mathcal {F}}$ of a Hamiltonian vector field $X_{{\mathcal {F}}}$ with a Hamiltonian ${\mathcal {F}}$ of the form

1.48 $$\begin{aligned} {{\mathcal {F}}}(\theta , y, w) := {{\mathcal {F}}}_{0}(\theta , y) + \big \langle {{\mathcal {F}}}_{1}(\theta , y), w \big \rangle \, , \end{aligned}$$

with the property that $X_{{\mathcal {F}} }$ is a smoothing Hamiltonian vector field (cf. Lemma 3.19). Hence its flow is a smoothing perturbation of the identity, implying that the Hamiltonian vector field of the Hamiltonian ${{\mathcal {H}}} \circ \Phi _{{\mathcal {F}}}$ has a normal component, which is again of the form (1.45) (cf. Lemma 3.17). To construct ${\mathcal {F}}$, we only need to impose zeroth and first Melnikov conditions on $\omega$, i.e., $\omega \in \Pi _\gamma ^{(0)} \cap \Pi _\gamma ^{(1)}$ (cf. (1.20)). For notational convenience, the Hamiltonian vector field obtained in this way is again denoted by $X = (X^{(\theta )}, X^{(y)}, X^\bot )$. The $y-$component $X^{(y)}$ is small of order three and the normal component $X^\bot$ of *X* at ${\mathfrak {x}} = (\theta , y, w)$ has the form

1.49 $$\begin{aligned} X^\bot ({\mathfrak {x}}) = \mathrm{i} \Omega _\bot [w] + X^\bot _1(\theta , y)[w] + X^\bot _2(\theta )[w, w] + \text {term small of order three} \end{aligned}$$

where

1.50 $$\begin{aligned} \begin{aligned}&X^\bot _1(\theta , y)[w] = \Pi _\bot \sum _{k = 0}^{N+1} T_{a_{1 - k}(\theta , y)} \partial _x^{1 - k}w + {{\mathcal {R}}}^\bot _{N, 1}(\theta , y)[w]\,, \\&X^\bot _2(\theta )[w, w] = \Pi _\bot \sum _{k = 0}^{N+1} T_{A_{1 - k}(\theta )[w]} \partial _x^{1 - k} w + {{\mathcal {R}}}^\bot _{N, 2}(\theta )[w, w] \, , \end{aligned} \end{aligned}$$

and for any $0 \le k \le N + 1$, $a_{1 - k} (\theta , y)$ is small of order one, $w \mapsto A_{1 - k}(\theta )[w]$ is a linear operator, whereas $w \mapsto {{\mathcal {R}}}^\bot _{N, 1}(\theta , y)[w]$ is a linear smoothing operator (smoothing of order $N+1$), and $w \mapsto {{\mathcal {R}}}^\bot _{N, 2}(\theta )[w,w]$ is a quadratic smoothing operator (smoothing of order $N+1$). The term in (1.49), which is small of order three, is the sum of a para-differential vector field of order one and a smoothing vector field.

The second step of our normal form procedure is developed in Sect. 6. Since $\Pi _\gamma ^{(3)}$ (cf. (1.20)) allows for a *loss of derivatives in space*, we first need to reduce the terms in the Taylor expansion of the normal component $X^\bot$ of *X*, which are linear and quadratic in *w*, to constant coefficients up to smoothing terms—see Sect. 6.1. This regularization procedure is achieved by constructing a transformation which is *not canonical*, but nevertheless preserves the following important property, needed for the energy estimates: the linearization of $X^\bot$ at $w = 0$ equals $X^\bot _1(\theta , y)$ and hence *is Hamiltonian*. In particular, the diagonal elements of the Fourier matrix representation of the linear operator $X_1^\bot (\theta , y)$ are purely imaginary,

1.51 $$\begin{aligned}{}[X^\bot _1(\theta , y)]_j^j \in \mathrm{i} {\mathbb {R}}, \qquad \forall j \in S^\bot \,. \end{aligned}$$

We remark that in the spirit of [23], one could construct a canonical transformation, but the construction of the one in Sect. 6.1 is technically simpler and due to (1.51) suffices for our purposes.

We now describe the second step of our normal form procedure in more detail. We begin by normalizing the operator

$$\begin{aligned} \Pi _\bot T_{a_1(\theta , y)} \partial _x + \Pi _\bot T_{ A_1(\theta )[w]} \partial _x = \Pi _\bot T_{a_1(\theta , y) + A_1(\theta )[w]} \partial _x \end{aligned}$$

in the expansion of the vector field $X^\bot _1(\theta , y)[w] + X^\bot _2(\theta )[w, w]$ (cf. (1.49), (1.50)). We transform the vector field in (1.49) by the means of the time one flow map $\Phi _{Y}$ of the vector field

$$\begin{aligned} Y(\theta , y, w) = \big (0, \, 0, \, \Pi _\bot T_{b(\theta , y) + B(\theta )[w]} \partial _x^{- 1} w \big ) \end{aligned}$$

with *b* and *B* given by

1.52 $$\begin{aligned} b(\theta , y):= & {} \frac{1}{3} \partial _x^{- 1}\big (\langle a_{1}(\theta , y) \rangle _x - a_{1}(\theta , y) \big ), \nonumber \\ B(\theta )[w]:= & {} \frac{1}{3} \partial _x^{- 1}\big (\langle A_{1}(\theta )[w] \rangle _x - A_{1}(\theta )[w] \big ). \end{aligned}$$

(Recall that for $a \in L^2({\mathbb {T}}_1)$, $\langle a \rangle _x = \int _0^1 a\, d x$.) Note that *b* and *B* satisfy

1.53 $$\begin{aligned} 3 \partial _x b(\theta , y) + a_{1 }(\theta , y) = \langle a_{1 }(\theta , y) \rangle _x, \quad 3 \partial _x B(\theta )[w] + A_{1 }(\theta )[w] = \langle A_{1 }(\theta )[w] \rangle _x. \end{aligned}$$

For notational convenience, we denote the transformed vector field also by $X_{1} = (X_{1}^{(\theta )}, \, X_{1}^{(y)}, \, X_{1}^\bot )$. We show that $X_{1}^{(y)}$ is small of order three and that $X_{1}^\bot (\theta , y, w)$ has the form

1.54 $$\begin{aligned}&\mathrm{i} \Omega _\bot w + {{\mathcal {D}}}^{\bot }_{1, 1}(\theta , y)[w] + {{\mathcal {D}}}^{\bot }_{1, 2}(\theta , w)[w] + X_{1, 1}^{\bot }(\theta , y)[w] \nonumber \\&\quad + X_{1, 2}^{\bot }(\theta )[w, w] + \text {term small of order three} \end{aligned}$$

with

$$\begin{aligned}&{{\mathcal {D}}}^{\bot }_{1, 1}(\theta , y) := \langle a_1(\theta , y) \rangle _x \partial _x , \qquad {{\mathcal {D}}}^{\bot }_{1, 2}(\theta , w) := \langle A_1(\theta )[w] \rangle _x \partial _x\, , \quad \\&X_{1, 1}^{\bot }(\theta , y)[w] : = \Pi _\bot \sum _{k = 1}^{N+1} T_{a_{1,1 - k}(\theta , y)} \partial _x^{ 1- k} w + {{\mathcal {R}}}_{N, 1}^{\bot }(\theta , y)[w]\, , \qquad \\&X_{1, 2}^{\bot }(\theta )[w, w] := \Pi _\bot \sum _{k = 1}^{N + 1} T_{A_{1, 1 - k}(\theta )[w]} \partial _x^{1 - k} w + {{\mathcal {R}}}_{N, 2}^{\bot }(\theta )[w, w]\, , \qquad \end{aligned}$$

where for any $1 \le k \le N+1$, $a_{1, 1 - k}(\theta , y)$ is small of order one and $w \mapsto A_{1, 1 - k}(\theta )[w]$ is a linear operator. Furthermore, ${{\mathcal {R}}}_{N, 1}^{\bot }(\theta , y)$ is a smoothing linear operator and ${{\mathcal {R}}}_{N, 2}^{\bot }(\theta )$ is a smoothing bilinear operator. The term in (1.54), which is small of order three, is the sum of a para-differential vector field of order one and a smoothing vector field. We also show that the linear vector field $X_{1, 1}^{\bot }(\theta , y)[w]$ in (1.54) satisfies the property (1.51), i.e., $[X_{1, 1}^{\bot }(\theta , y)]_j^j \in \mathrm{i} {\mathbb {R}}$ for any $j \in S^\bot$, and that the Fourier multiplier ${{\mathcal {D}}}^{\bot }_{1, 1}(\theta , y)$ is skew-adjoint. By iterating this procedure $N + 2$ times, one gets a vector field, which we denote by $X_{4} = (X_{4}^{(\theta )}, X_{4}^{(y)}, X_{4}^\bot )$ (cf. Proposition 6.1), with the following properties: $X_{4}^{(y)}$ is small of order three and $X_{4}^\bot (\theta , y, w)$ has the form

1.55 $$\begin{aligned} \begin{aligned}&\mathrm{i} \Omega _\bot w + {{\mathcal {D}}}_{4, 1}^{\bot }(\theta , y)[w] + {{\mathcal {D}}}_{4, 2}^{\bot }(\theta , w)[w] + {{\mathcal {R}}}_{N, 1}^{\bot }(\theta , y)[w] \\&\quad + {{\mathcal {R}}}_{N, 2}^{\bot }(\theta )[w, w] + \text {term small of order three} \, . \end{aligned} \end{aligned}$$

Here ${{\mathcal {D}}}_{4, 1}^{\bot }(\theta , y)$ and ${{\mathcal {D}}}_{ 4, 2}^{\bot }(\theta , w)$ are Fourier multipliers of the form

1.56 $$\begin{aligned} \begin{aligned} {{\mathcal {D}}}_{4, 1}^{\bot }(\theta , y) = \sum _{k = 0}^{N+1} \lambda _{1 - k}(\theta , y) \partial _x^{1 - k} \, , \quad {{\mathcal {D}}}_{4, 2}^{\bot }(\theta , w) := \sum _{k = 0}^{N+1} \Lambda ^\bot _{1 - k}(\theta ) [w] \partial _x^{1 - k},\qquad \end{aligned} \end{aligned}$$

where for any $0 \le k \le N +1$, $\lambda _{1 - k}(\theta , y) \in {\mathbb {R}}$ is small of order one and $w \mapsto \Lambda ^\bot _{1 - k}(\theta )[w] \in {\mathbb {R}}$ is a linear operator. The remainder ${{\mathcal {R}}}_{N, 1}^{\bot }(\theta , y)$ is a smoothing linear operator and ${{\mathcal {R}}}_{N, 2}^{\bot }(\theta )$ is a smoothing bilinear operator. In addition, the Fourier multiplier ${{\mathcal {D}}}_{4, 1}^{\bot }(\theta , y)$ is skew-adjoint. Moreover we show that

1.57 $$\begin{aligned} {[}{{\mathcal {R}}}_{N, 1}^{\bot }(\theta , y)]_j^j \in \mathrm{i} {\mathbb {R}}, \quad \forall j \in S^\bot . \end{aligned}$$

Since the transformation $\Phi _Y$ and the subsequent transformations constructed in the interative procedure are not canonical, the linear operator ${{\mathcal {D}}}_{4, 2}^{\bot }(\theta , w)$ is not necessarily skew-adjoint. However the leading order term $\Lambda ^\bot _1(\theta )[w] \partial _x$ of ${{\mathcal {D}}}_{4, 2}^{\bot }(\theta , w)$ is skew-adjoint since $\Lambda ^\bot _1(\theta )[w]\in {\mathbb {R}}$.

In Sect. 6.2 we design a normal form procedure to remove

1.58 $$\begin{aligned} \sum _{k = 1}^{N+1} \Lambda ^\bot _{1 - k}(\theta ) [w] \partial _x^{1 - k} \end{aligned}$$

from ${{\mathcal {D}}}_{4, 2}^{\bot }(\theta , w)$ which requires to impose first Melnikov conditions on $\omega$ (cf. definition (1.20) of $\Pi _\gamma ^{(1)}$). We transform the vector field $X_{4}$ (cf. (1.55)) by the means of the time one flow map of a vector field, which in view of (1.58) is chosen to be of the form

1.59 $$\begin{aligned} \big ( 0, \, 0, \, \sum _{k = 1}^{N+1} \Xi ^\bot _{1 - k}(\theta )[w] \partial _x^{1 - k}w \big ) \, \end{aligned}$$

where for any $1 \le k \le N +1$, the linear functional $w \mapsto \Xi ^\bot _{1 - k}(\theta )[w]$ is a solution of

1.60 $$\begin{aligned} \omega \cdot \partial _\theta \, \Xi ^\bot _{1 - k}(\theta )[w] - \Xi ^\bot _{1 - k}(\theta )[ \mathrm{i} \Omega _\bot w] + \Lambda ^\bot _{1 - k}(\theta )[w] = 0 \, . \end{aligned}$$

The latter equation can be solved if $\omega \in \Pi _\gamma ^{(1)}$ (first Melnikov conditions). The transformed vector field is denoted by $X_{5} = (X_{5}^{(\theta )}, X_{5}^{(y)}, X_{5}^\bot )$. We show that $X_{5}^{(y)}$ is small of order three and that $X_{5}^\bot (\theta , y, w)$ has the form

1.61 $$\begin{aligned} \begin{aligned}&\mathrm{i} \Omega _\bot w + {{\mathcal {D}}}^\bot _{5}(\theta , y, w)[w] + {{\mathcal {R}}}^\bot _{N, 1}(\theta , y)[w] + {{\mathcal {R}}}_{N, 2}^\bot (\theta )[w, w]\\&\quad + \text { term small of order three} , \end{aligned} \end{aligned}$$

where

1.62 $$\begin{aligned} {{\mathcal {D}}}^\bot _{5}({\mathfrak {x}}) := {{\mathcal {D}}}^\bot _{4, 1}(\theta , y) + \Lambda ^\bot _1(\theta )[w] \partial _x \end{aligned}$$

and ${{\mathcal {R}}}_{N, 1}^\bot$, ${{\mathcal {R}}}_{N, 2}^\bot$ are as in (1.55). Clearly, the Fourier multiplier ${{\mathcal {D}}}^\bot _{5}({\mathfrak {x}})$ is skew-adjoint.

Finally in Sect. 6.3 we normalize the term in the Taylor expansion of the $\theta$-component $X_{5}^{(\theta )}$ of $X_{5}$, which is quadratic in *w*, and normalize the smoothing vector fields ${{\mathcal {R}}}_{N, 1}^\bot$ and ${{\mathcal {R}}}_{N, 2}^\bot$ in $X^\bot _5$. Let us explain in more detail how to achieve the latter. We transform the vector field $X_{5}$ by the time one flow map generated by the vector field

1.63 $$\begin{aligned} \big ( 0, \, 0, \, \, {{\mathcal {S}}}^\bot _1(\theta , y)[w] + {{\mathcal {S}}}^\bot _2(\theta )[w, w] \big ) \end{aligned}$$

where ${{\mathcal {S}}}^\bot _1(\theta , y)$ is a smoothing linear operator and ${{\mathcal {S}}}^\bot _2(\theta )$ is a smoothing bilinear operator. They are chosen to be solutions of

1.64 $$\begin{aligned} - \omega \cdot \partial _\theta \, {{\mathcal {S}}}^\bot _1(\theta , y) + [\mathrm{i} \Omega _\bot , \, {{\mathcal {S}}}^\bot _1(\theta , y)]_{lin} + {{\mathcal {R}}}^\bot _{N, 1}(\theta , y) = {{\mathcal {Z}}}^\bot (y) \, \qquad \end{aligned}$$

and, respectively,

1.65 $$\begin{aligned}&- \omega \cdot \partial _\theta \, {{\mathcal {S}}}^\bot _2(\theta )[w, w] +\mathrm{i} \Omega _\bot {{\mathcal {S}}}^\bot _2(\theta )[w, w] - {{\mathcal {S}}}^\bot _2(\theta )\big ( [\mathrm{i} \Omega _\bot w, w] + [w, \mathrm{i} \Omega _\bot w] \big )\nonumber \\&\quad + {{\mathcal {R}}}^\bot _{N, 2}(\theta )[ w, w] = 0 \, , \end{aligned}$$

where

1.66 $$\begin{aligned} {{\mathcal {Z}}}^\bot (y) := \mathrm{diag}_{j \in S^\bot } [\widehat{{\mathcal {R}}}^\bot _{N, 1}(0, y)]_j^{j}, \quad [\widehat{{\mathcal {R}}}^\bot _{N, 1}(0, y)]_j^j := \frac{1}{(2 \pi )^{S_+}} \int _{{\mathbb {T}}^{S_+}} [{{\mathcal {R}}}^\bot _{N, 1}(\theta , y)]_j^j\, d \theta .\nonumber \\ \end{aligned}$$

Equation (1.64) can be solved by imposing the *second* Melnikov conditions on $\omega$, i.e., $\omega \in \Pi _\gamma ^{(2)}$, and Eq. (1.65) by imposing the *third* Melnikov conditions, $\omega \in \Pi _\gamma ^{(3)}$ - see Lemma 6.1. Note that in Eq. (1.65), the right hand side vanishes, meaning that the left hand side does not contain any resonant terms. Finally we get a vector field $X_{6} = (X_{6}^{(\theta )}, X_{6}^{(y)}, X_{6}^\bot )$ where $X_{6}^{(y)}$ is small of order three and $X_{6}^\bot ({\mathfrak {x}})$ has the form

1.67 $$\begin{aligned} \begin{aligned} X_{6}^\bot ({\mathfrak {x}}) = \mathrm{i} \Omega _\bot w + {{\mathcal {D}}}^\bot _{5}({\mathfrak {x}})[w] + {{\mathcal {Z}}}^\bot (y)[w] + \text {term small of order three}\,. \end{aligned} \end{aligned}$$

By the property (1.57) and the definition (1.66) of ${{\mathcal {Z}}}^\bot (y)$, it follows that ${{\mathcal {Z}}}^\bot (y)$ and hence ${{\mathcal {D}}}^\bot _{5}({\mathfrak {x}}) + {{\mathcal {Z}}}^\bot (y)$ are skew-adjoint Fourier multiplier. Finally one shows that $X_{6}^\bot$ in (1.67) has the form stated in (1.47).

*Related work.* Prior to our work, no results have been obtained on the long time asymptotics of the solutions of Hamiltonian perturbations of integrable PDEs such as the KdV or the nonlinear Schrödinger equation on ${\mathbb {T}}_1$ with initial data close to a *periodic multi-soliton of possibly large amplitude*. For Hamiltonian perturbations of linear integrable PDEs on ${\mathbb {T}}_1$, which satisfy nonresonance conditions, a by now standard normal form method has been developed allowing to prove the stability of the *equilibrium solution* $u\equiv 0$ of (Hamiltonian) perturbations for time intervals of large size—see e.g. [2–4, 7, 13, 17, 18, 23, 37] and references therein. More recently, these techniques have been refined so that in specific cases, such results can also be proved for Hamiltonian perturbations of resonant linear integrable PDEs by approximating the perturbed equation by nonlinear integrable systems, satisfying nonresonance conditions—see [5, 13] for Hamiltonian perturbations of the linear Schrödinger equation and [6] for such perturbations of the Airy equation as well as the linearized Benjamin-Ono equation. We remark that for the Airy equation, the Hamiltonian perturbations considered in [6] are of the form $\partial _x \nabla P_f$ (cf. (1.6)–(1.7)) with the density *f*(*u*(*x*)) not explicitly depending on *x* and *f*(*z*) being analytic in a neighborhood of $z=0$ in ${\mathbb {C}}$.

Finally, we mention the recent paper [8] where it is proved by KAM methods that many periodic multi-solitons persist under quasi-linear perturbations of the KdV equation. As in this paper, a key ingredient are the normal form coordinates, constructed in [27].

## Para-Differential Calculus

In this section we review some standard notions and results of the para-differential calculus, needed throughout the paper. For details we refer to [37].

We begin with reviewing the notion of para-product. To this end we need the following

### Definition 2.1

A function $\psi \in C^\infty ({\mathbb {R}}\times {\mathbb {R}})$ is said to be an admissible cut-off function, if there exist $0< \varepsilon '< \varepsilon <1$ so that

$$\begin{aligned}&\mathrm{supp}(\psi ) \subseteq \{ (\eta , \xi ) \in {\mathbb {R}}\times {\mathbb {R}}: |\eta | \le \varepsilon \langle \xi \rangle \}\,, \qquad \quad \\&\quad \quad \psi (\eta , \xi ) = 1, \forall (\eta , \xi ) \in {\mathbb {R}}\times {\mathbb {R}}\ \text {with} \ |\eta | \le \varepsilon ' \langle \xi \rangle , \end{aligned}$$

and

$$\begin{aligned} |\partial _\eta ^{\alpha } \partial _\xi ^\beta \psi (\eta , \xi )| \lesssim _{\alpha , \beta } \langle \xi \rangle ^{- \alpha - \beta }\,, \qquad \forall (\alpha , \beta ) \in {\mathbb {Z}}_{\ge 0} \times {\mathbb {Z}}_{\ge 0} \end{aligned}$$

where by (1.21) $$\langle \xi \rangle = \max \{1, |\xi | \}$$.

Given a cut-off function $\psi$ as in Definition 2.1, the para-product $T_a u$ of a function $a\in H^{1}({\mathbb {T}}_1)$ with a function $u \in H^s({\mathbb {T}}_1)$, $s \ge 1$, is defined as

2.1 $$\begin{aligned} T_a u(x):= & {} \sigma _a(x, D)u(x) = \sum _{\xi \in {\mathbb {Z}}} \sigma _a(x, \xi ) {\widehat{u}}(\xi ) e^{\mathrm{i} 2 \pi \xi x}\,,\nonumber \\ \sigma _a(x, \xi ):= & {} \sum _{\eta \in {\mathbb {Z}}} \psi (\eta , \xi ) {\widehat{a}}(\eta ) e^{\mathrm{i} 2 \pi \eta x }\, , \end{aligned}$$

where ${\widehat{a}}(\eta )$, also denoted by $a_\eta$, is the $\eta$th Fourier coefficient of *a*,

$$\begin{aligned} {\widehat{a}}(\eta ) = \int _0^1 a(x) e^{- \mathrm{i} 2\pi \eta x} d x \, . \end{aligned}$$

### Lemma 2.1

For any $a \in H^{1}({\mathbb {T}}_1)$ and $s \ge 1$, $T_a$ is in ${{\mathcal {B}}}(H^s({\mathbb {T}}_1), H^s({\mathbb {T}}_1))$ and

2.2 $$\begin{aligned} \Vert T_a \Vert _{{{\mathcal {B}}}(H^s, H^s)} \lesssim _s \Vert a \Vert _{1}\,. \end{aligned}$$

Furthermore, for any $s \ge 1$, the map $H^1({\mathbb {T}}_1) \rightarrow {{\mathcal {B}}}(H^s({\mathbb {T}}_1), H^s({\mathbb {T}}_1)), \, a \mapsto T_a$, is linear.

Given two functions $a, u \in H^s({\mathbb {T}}_1)$ with $s \ge 1$, their product can be split as

2.3 $$\begin{aligned} a u = T_a u + T_u a + {{\mathcal {R}}}^{(B)}(a, u)\,, \end{aligned}$$

where the remainder ${{\mathcal {R}}}^{(B)}(a, u)$ is given by

2.4 $$\begin{aligned} {{\mathcal {R}}}^{(B)}(a, u) (x)= \sum _{\eta , \xi \in {\mathbb {Z}}} \omega (\eta , \xi ){\widehat{a}}(\eta ) {\widehat{u}}(\xi ) e^{\mathrm{i} 2 \pi (\eta + \xi ) x}\,, \quad \omega (\eta , \xi ) := 1- \psi (\eta , \xi )- \psi (\xi , \eta )\,.\nonumber \\ \end{aligned}$$

Note that the support $\mathrm{supp}(\omega )$ of $\omega : {\mathbb {Z}}\times {\mathbb {Z}}\rightarrow {\mathbb {R}}$ satisfies

2.5 $$\begin{aligned}&\big \{ (\eta , \xi ) \in {\mathbb {Z}}^{2 } : \varepsilon \langle \xi \rangle< |\eta |< \frac{\langle \xi \rangle }{\varepsilon } \big \} \cup \{ (0,0) \} \subseteq \mathrm{supp}(\omega ) \nonumber \\&\quad \subseteq \big \{ (\eta , \xi ) \in {\mathbb {Z}}^{2 } : \varepsilon ' \langle \xi \rangle< |\eta | < \frac{ \langle \xi \rangle }{\varepsilon '} \big \} \cup \{ (0,0) \} \,. \end{aligned}$$

The main feature of ${{\mathcal {R}}}^{(B)}(a, u)$ is that it is a regularizing bilinear operator in the following sense.

### Lemma 2.2

For any $$s_1, s_2 \ge 0$$,

$$\begin{aligned} {\mathcal {R}}^{(B)} : H^{s_1 + 1}({\mathbb {T}}_1) \times H^{s_2}({\mathbb {T}}_1) \rightarrow H^{s_1 + s_2}({\mathbb {T}}_1), \, (a, u) \mapsto {{\mathcal {R}}}^{(B)}(a, u) \end{aligned}$$

is a bilinear map, satisfying

2.6 $$\begin{aligned} \Vert {{\mathcal {R}}}^{(B)}(a, u) \Vert _{s_1 + s_2} \lesssim _{s_1, s_2} \Vert a \Vert _{s_1 + 1} \Vert u \Vert _{s_2}\, \qquad \forall \, a \in H^{s_1 + 1}({\mathbb {T}}_1), \, u \in H^{s_2}({\mathbb {T}}_1)\, . \end{aligned}$$

Next, we discuss the standard symbolic calculus for para-differential operators to the extent needed in this paper. It suffices to consider operators of the form

2.7 $$\begin{aligned} T_a \partial _x^m, \qquad a \in H^1({\mathbb {T}}_1), \ m \in {\mathbb {Z}}\,, \end{aligned}$$

where we recall that for any $m \in {\mathbb {Z}}$, the Fourier multiplier $\partial _x^m$ is defined by

$$\begin{aligned} \partial _x^m [e^{\mathrm{i} 2 \pi j x}] := ( \mathrm{i} 2 \pi j)^m e^{\mathrm{i} 2 \pi j x}\,, \ \ \forall \, j \ne 0\,, \qquad \partial _x^m[1] := 0\,. \end{aligned}$$

Alternatively, $\partial _x^m$ can be written as the pseudo-differential operator $\mathrm{Op}( ( \mathrm{i} 2 \pi \xi )^m \chi (\xi ))$ with symbol $( \mathrm{i} 2 \pi \xi )^m \chi (\xi )$ where $\chi : {\mathbb {R}}\rightarrow {\mathbb {R}}$ is a $C^\infty -$smooth cut-off function, satisfying

2.8 $$\begin{aligned} \chi (\xi ) = 1\,, \ \ \forall \, |\xi | \ge \frac{2}{3}\,, \qquad \chi (\xi ) = 0\,, \ \ \forall \, |\xi | \le \frac{1}{3}\,. \end{aligned}$$

The symbol of an operator of the form (2.7) is given by

$$\begin{aligned} \sigma _{a}(x, \xi ) = \sum _{\eta \in {\mathbb {Z}}} \psi (\eta , \xi ) {\widehat{a}}(\eta ) ( \mathrm{i} 2 \pi \xi )^m e^{\mathrm{i} 2 \pi \eta x}\,. \end{aligned}$$

### Lemma 2.3

Let $a, b \in H^{N+3}({\mathbb {T}}_1)$ with $N \in {\mathbb {N}}$. Then

$$\begin{aligned} T_a \circ T_b = T_{ab} + {{\mathcal {R}}}_N(a, b) \end{aligned}$$

where for any $s \ge 0$,

$$\begin{aligned} {\mathcal {R}}_N : H^{N+3}({\mathbb {T}}_1) \times H^{N+3} ({\mathbb {T}}_1) \rightarrow {{\mathcal {B}}}\big ( H^s({\mathbb {T}}_1), H^{s + N+1}({\mathbb {T}}_1)\big ), \, (a, b) \mapsto {{\mathcal {R}}}_N(a, b)\, , \end{aligned}$$

is a bilinear map, satisfying

$$\begin{aligned} \Vert {{\mathcal {R}}}_N(a, b)\Vert _{{{\mathcal {B}}}(H^s, H^{s + N+1})} \lesssim _{s, N} \Vert a \Vert _{N+3} \Vert b \Vert _{N+3} \, , \qquad \forall \, a, b \in H^{N+3}({\mathbb {T}}_1). \end{aligned}$$

### Lemma 2.4

Let $m \in {\mathbb {Z}}$, $N \in {\mathbb {N}}$. Then there exist an integer $\sigma _N > N + m$ and combinatorial constants $(K_{n, m})_{1 \le n \le N + m}$, with $K_{1, m} = m$ so that for any $a \in H^{\sigma _N}({\mathbb {T}}_1)$

$$\begin{aligned} \partial _x^m \circ T_a = T_{ a} \partial _x^{m } + \sum _{n = 1}^{N+m} K_{n, m} T_{\partial _x^n a} \partial _x^{m - n} + {{\mathcal {R}}}_{N, m}(a)\, , \end{aligned}$$

where for any $s \ge 0$, the map

$$\begin{aligned} {{\mathcal {R}}}_{N, m} : H^{\sigma _N}({\mathbb {T}}_1) \rightarrow {{\mathcal {B}}}(H^s({\mathbb {T}}_1), H^{s + N + 1}({\mathbb {T}}_1)), \, a \mapsto {{\mathcal {R}}}_{N, m}(a) \end{aligned}$$

is linear and satisfies the estimate

$$\begin{aligned} \Vert {{\mathcal {R}}}_{N, m}(a)\Vert _{ {{\mathcal {B}}}(H^s, H^{s + N + 1})} \lesssim _{s, m, N} \Vert a \Vert _{\sigma _N}\,, \quad \forall a \in H^{\sigma _N}({\mathbb {T}}_1) , \end{aligned}$$

and where we use the customary convention that the sum $\sum _{n = 1}^{N+m}$ equals 0 if $N+m < 1$.

Combining Lemmas 2.3 and 2.4 yields the following

### Lemma 2.5

Let $m, m' \in {\mathbb {Z}}$, $N \in {\mathbb {N}}$. Then there exists an integer $\sigma _N > N + m$ so that for any $a, b \in H^{\sigma _N}({\mathbb {T}}_1)$,

2.9 $$\begin{aligned} T_a \partial _x^m \circ T_b \partial _x^{m'} = T_{a b} \partial _x^{m + m' } + \sum _{n = 1}^{N+m+m'} K_{n, m} T_{a \partial _x^n b} \partial _x^{m + m' - n} + {{\mathcal {R}}}_{N, m, m'}(a, b) \, , \end{aligned}$$

where $K_{n, m}$ are the combinatorial constants of Lemma 2.4 and where for any $s \ge 0$, the map

$$\begin{aligned} {{\mathcal {R}}}_{N, m, m'} : H^{\sigma _N}({\mathbb {T}}_1) \times H^{\sigma _N}({\mathbb {T}}_1) \rightarrow {{\mathcal {B}}}(H^s({\mathbb {T}}_1), \, H^{s + N + 1}({\mathbb {T}}_1)), \, (a, b) \mapsto {{\mathcal {R}}}_{N, m, m'}(a, b) \end{aligned}$$

is bilinear and satisfies the estimate

$$\begin{aligned} \Vert {{\mathcal {R}}}_{N, m, m'}(a, b)\Vert _{{{\mathcal {B}}}(H^s, H^{s + N + 1})} \lesssim _{s, m, N} \Vert a \Vert _{\sigma _N} \Vert b \Vert _{\sigma _N} \, , \qquad \forall \, a, b \in H^{\sigma _N}({\mathbb {T}}_1). \end{aligned}$$

According to Lemma 2.3, in the case $m=0$, a possible choice is $\sigma _N= N+ 3$, $K_{n, 0} = 0$ for $1 \le n \le N+m'$.

Using that $K_{1,m} = m$, one infers from Lemma 2.5 an expansion of the commutator $[T_a \partial _x^m \,,\, T_b \partial _x^{m'} ]_{lin}$.

### Corollary 2.1

(Commutator expansion). Let $m, m' \in {\mathbb {Z}}$, $N \in {\mathbb {N}}$. Then there exists $\sigma _N > N +m +m'$ so that for any $a, b \in H^{\sigma _N}({\mathbb {T}}_1)$, $[T_a \partial _x^m \,,\, T_b \partial _x^{m'} ]_{lin}$ has an expansion of the form

2.10 $$\begin{aligned} T_{m a \partial _x b - m' b \partial _x a } \partial _x^{m + m' - 1} + \sum _{n = 2}^{N+m+m'} \big ( K_{n, m} T_{a \partial _x^n b} - K_{n,m'} T_{b \partial _x^n a } \big ) \partial _x^{m + m' - n} + {{\mathcal {R}}}^{{\mathcal {C}}}_{N, m, m'}(a, b)\, \end{aligned}$$

where for any $s \ge 0$, the map

$$\begin{aligned} {{\mathcal {R}}}^{{\mathcal {C}}}_{N, m, m'} : H^{\sigma _N}({\mathbb {T}}_1) \times H^{\sigma _N}({\mathbb {T}}_1) \rightarrow {{\mathcal {B}}}\big ( H^s({\mathbb {T}}_1), H^{s + N + 1}({\mathbb {T}}_1) \big ), \, (a, b) \mapsto {{\mathcal {R}}}^{\mathcal {C}}_{N, m, m'}(a, b) \end{aligned}$$

is bilinear and satisfies

$$\begin{aligned} \Vert {{\mathcal {R}}}^{\mathcal {C}}_{N, m, m'}(a, b)\Vert _{{{\mathcal {B}}}(H^s, H^{s + N + 1})} \lesssim _{s, m, m', N} \Vert a \Vert _{\sigma _N} \Vert b \Vert _{\sigma _N}\, , \qquad \forall \, a, b \in H^{\sigma _N}({\mathbb {T}}_1) \,. \end{aligned}$$

According to Lemma 2.3, in the case $m=0, m'=0$, $[T_a \, ,\, T_b ]_{lin} = {{\mathcal {R}}}_N(a, b) - {{\mathcal {R}}}_N(b, a)$. Hence a possible choice is $\sigma _N= N+ 3$, $K_{n, 0} = 0$ for $1 \le n \le N$.

Finally, we discuss the adjoint $T_a^\top$ of $T_a$ with respect to the standard $L^2-$inner product.

### Lemma 2.6

Let $a \in H^{N+1}({\mathbb {T}}_1)$ with $N \in {\mathbb {N}}$. Then $T_a^\top = T_{ a} + {{\mathcal {R}}}_\top ( a)$ where for any $s \ge 0$, the map

$$\begin{aligned} {\mathcal {R}}_\top : H^{N+1}({\mathbb {T}}_1) \rightarrow {{\mathcal {B}}}\big ( H^s({\mathbb {T}}_1), H^{s + N + 1}({\mathbb {T}}_1) \big ), \, a \mapsto {{\mathcal {R}}}_\top (a) \, , \end{aligned}$$

is linear and for any $a \in H^{N+1}({\mathbb {T}}_1)$ satisfies $\Vert {{\mathcal {R}}}_\top (a)\Vert _{{{\mathcal {B}}}(H^s, H^{s + N+1})} \lesssim _{s, N} \Vert a \Vert _{N+1}$.

Combining Lemmas 2.4 and 2.6 yields the following

### Corollary 2.2

Let $m \in {\mathbb {Z}}$, $N \in {\mathbb {N}}$. Then there exists an integer $\sigma _N > N + m$ so that for any $a \in H^{\sigma _N}({\mathbb {T}}_1)$, $(T_a \partial _x^m)^\top$ admits the expansion

$$\begin{aligned} (T_a \partial _x^m)^\top = (- 1)^m T_{ a} \partial _x^{m } + (- 1)^m\sum _{n = 1}^{N+m} K_{n, m}T_{\partial _x^n a} \partial _x^{m - n} + {{\mathcal {R}}}_{\top , N, m}(a) , \end{aligned}$$

where $K_{n, m}$ are the combinatorial constants of Lemma 2.4, and where for any $s \ge 0$, the map

$$\begin{aligned} {{\mathcal {R}}}_{\top , N, m} : H^{\sigma _N}({\mathbb {T}}_1) \rightarrow {{\mathcal {B}}}(H^s({\mathbb {T}}_1), H^{s + N + 1}({\mathbb {T}}_1)), \, a \mapsto {{\mathcal {R}}}_{\top , N, m}(a), \end{aligned}$$

is linear and for any $a \in H^{\sigma _N}({\mathbb {T}}_1)$ satisfies $\Vert {{\mathcal {R}}}_{\top , N, m}(a)\Vert _{{{\mathcal {B}}}(H^s, H^{s + N + 1})} \lesssim _{s, N} \Vert a \Vert _{\sigma _N}$.

## Para-Differential Vector Fields

In this section we introduce several classes of vector fields, compute the commutators between vector fields from these classes and study their flows. As part of the proof of Theorem 1.1 , these vector fields are used to transform equation (1.4) into normal form.

### Definitions

#### Definition 3.1

(*Para-differential vector fields*). Let *N*, $p \in {\mathbb {N}}$ and $m \in {\mathbb {Z}}$. A vector field $X^\bot$ in normal direction, defined on a subset of ${\mathcal {E}}$ and depending on the parameters $\varepsilon$ and $\mu$, is said to be of class ${{\mathcal {O}} B}(m, N)$, $X^\bot \in {{\mathcal {O}} B}(m, N)$, if it is of the form

3.1 $$\begin{aligned} X^\bot ({\mathfrak {x}}) =\Pi _\bot \sum _{k = 0}^{N + m} T_{a_{m - k}({\mathfrak {x}})} \partial _x^{m - k} w \end{aligned}$$

and has the following property: there are integers $\sigma _N, s_N \ge 0$ so that for any $s \ge s_N$ there exist $0< \delta \equiv \delta (s, N) < 1$ and $0< \varepsilon _0 \equiv \varepsilon _0(s, N) < 1$ so that for any $0 \le k \le N + m$

$$\begin{aligned} a_{m - k} : {{\mathcal {V}}}^{s + \sigma _N}(\delta ) \times [0, \varepsilon _0] \rightarrow H^s({\mathbb {T}}_1), \, ({\mathfrak {x}}, \varepsilon ) \mapsto a_{m - k}({\mathfrak {x}}) \equiv a_{m - k}({\mathfrak {x}}, \varepsilon ) \end{aligned}$$

is $C^\infty -$smooth and together with each of its derivatives bounded. $X^{\bot }$ is said to be of class ${\mathcal {OB}}^p(m, N)$ if it is in ${\mathcal {OB}}(m, N)$ and in addition, the functions $a_{m -k}$ are small of order $p - 1$.

#### Remark 3.1

(i) If $N+ m < 0$ in (3.1), the sum is defined to be the zero vector field. As a consequence, ${\mathcal {OB}}(m, N) = \{ 0 \}$ if $N + m < 0$. Throughout the paper, the same convention holds for any sum of terms, indexed by an empty set, and for any of the used classes of vector fields.
(ii) We point out that the bounds are uniform in the parameter $\mu$, but no regularity assumptions with respect to $\mu$ are required. Throughout the paper, the same convention holds.

#### Definition 3.2

(*Fourier multiplier vector fields*). Let *N*, $p \in {\mathbb {N}}$ and $m \in {\mathbb {Z}}$. A vector field ${\mathcal {M}}^\bot$ in normal direction, defined on a subset of ${\mathcal {E}}$ and depending on the parameters $\varepsilon$ and $\mu$, is said to be of class ${{\mathcal {O}} F}(m, N)$, ${\mathcal {M}}^\bot \in {{\mathcal {O}} F}(m, N)$, if it is of the form

3.2 $$\begin{aligned} {\mathcal {M}}^\bot ({\mathfrak {x}}) = \sum _{k = 0}^{N + m} \lambda _{m - k}({\mathfrak {x}}) \partial _x^{m - k} w \end{aligned}$$

and has the following property: there exist an integer $\sigma _N \ge 0$, $0< \delta \equiv \delta (N) <1$, and $0< \varepsilon _0 \equiv \varepsilon _0(N) < 1$ so that for any $0 \le k \le N + m$,

$$\begin{aligned} \lambda _{m - k} : {{\mathcal {V}}}^{\sigma _N}(\delta ) \times [0, \varepsilon _0] \rightarrow {\mathbb {R}}, \, ({\mathfrak {x}}, \varepsilon ) \mapsto \lambda _{m - k}({\mathfrak {x}}) \equiv \lambda _{m - k}({\mathfrak {x}}, \varepsilon ) \end{aligned}$$

is $C^\infty$-smooth and together with each of its derivatives bounded. ${\mathcal {M}}^{\bot }$ is said to be of class ${\mathcal {OF}}^p(m, N)$ if it is in ${\mathcal {OF}}(m, N)$ and in addition, the functions $\lambda _{m -k}$ are small of order $p - 1$.

#### Definition 3.3

(*Smoothing vector fields*). Let *N*, $p \in {\mathbb {N}}$. A vector field ${\mathcal {R}}$, defined on a subset of ${\mathcal {E}}$ and depending on the parameters $\varepsilon$ and $\mu$, is said to be of class ${\mathcal {OS}}( N)$, ${\mathcal {R}} \in {\mathcal {OS}}(N)$, if there exist $s_N \ge 0$ so that for any $s \ge s_N$, there exist $0< \delta \equiv \delta (s, N) <1$ and $0< \varepsilon _0 \equiv \varepsilon _0(s, N) < 1$ with the property that

$$\begin{aligned} {{\mathcal {R}}} : {{\mathcal {V}}}^s(\delta ) \times [0, \varepsilon _0] \rightarrow E_{s + N + 1}, \, ({\mathfrak {x}}, \varepsilon ) \mapsto {{\mathcal {R}}}({\mathfrak {x}}) \equiv {{\mathcal {R}}}({\mathfrak {x}}, \varepsilon ) \end{aligned}$$

is $C^\infty$-smooth and together with each of its dervatives bounded. ${{\mathcal {R}}}$ is said to be of class ${\mathcal {OS}}^p(N)$ if it is in ${\mathcal {OS}}(N)$ and in addition is small of order *p*.

#### Remark 3.2

For notational convenience, in the sequel, we refer to a function, which is $C^\infty$-smooth and together with each of its derivatives bounded, as a function which is $C^\infty$-smooth and bounded.

Next we introduce special classes of vector fields which are small of order 2 with respect to *y*, *w*, $\varepsilon$.

#### Definition 3.4

Let $$N \in {\mathbb {N}}$$ and $$m \in {\mathbb {Z}}$$.

(i) Assume that $X^\bot ({\mathfrak {x}}) = \Pi _\bot \sum _{k = 0}^{m + N} T_{a_{m - k}({\mathfrak {x}})} \partial _x^{m - k} w$ is of class ${\mathcal {OB}}^2(m, N)$.
(i1) $X^\bot$ is said to be of class ${\mathcal {OB}}^2_{w}(m , N)$ if it is linear with respect to *w*. As a consequence, for any $0 \le k \le m + N$, the coefficient $a_{m - k}$ is small of order one and independent of *w*. More precisely, there is an integer $s_N \ge 0$ so that for any $s \ge s_N$, there exist $0< \delta \equiv \delta (s, N) < 1$ and $\varepsilon _0 \equiv \varepsilon _0(s, N) > 0$ with the property that
  $$\begin{aligned} a_{m - k} : {\mathbb {T}}^{S_+} {\times } B_{S_+}(\delta ) \times [0, \varepsilon _0] {\rightarrow } H^s({\mathbb {T}}_1), \, (\theta , y, \varepsilon ) \mapsto a_{m - k}(\theta , y) \equiv a_{m - k}(\theta , y, \varepsilon ) \end{aligned}$$
  is $C^\infty$-smooth and bounded (cf. Remark 3.2). In this case, we often write $X^\bot (\theta , y)[w]$ instead of $X^\bot ({\mathfrak {x}})$ where
  $$\begin{aligned} X^\bot (\theta , y) := \Pi _\bot \sum _{k = 0}^{N+m} T_{a_{m - k}(\theta , y)} \partial _x^{m - k}\, . \end{aligned}$$
(i2) $X^\bot$ is said be of class ${\mathcal {OB}}^2_{ww}(m, N)$ if it is quadratic with respect to *w* and independent of *y*. As a consequence, for any $0 \le k \le m + N$, the coefficient $a_{m - k}$ is linear with respect to *w* and independent of *y*. More precisely, there are integers $s_N \ge 0$, $\sigma _N \ge 0$ so that for any $s \ge s_N$ there exist $0< \delta \equiv \delta (s, N) < 1$ and $0< \varepsilon _0 \equiv \varepsilon _0(s, N) < 1$ with the property that
  $$\begin{aligned} a_{m - k} : {\mathbb {T}}^{S_+} {\times } H_\bot ^{s + \sigma _N} \times [0, \varepsilon _0] \rightarrow H^s({\mathbb {T}}_1), \, (\theta , w, \varepsilon ) \mapsto a_{m - k}(\theta , w) \equiv A_{m - k}(\theta )[w] \, , \end{aligned}$$
  with
  $$\begin{aligned} A_{m - k}: {\mathbb {T}}^{S_+} \times [0, \varepsilon _0] \rightarrow {{\mathcal {B}}}(H^{s + \sigma _N}_\bot ({\mathbb {T}}_1), H^s({\mathbb {T}}_1)), \, (\theta , \varepsilon ) \mapsto A_{m - k}(\theta ) \equiv A_{m - k}(\theta , \varepsilon ) \ \end{aligned}$$
  being $C^\infty$-smooth and bounded. In this case we often write $X^\bot (\theta , w)[w]$ instead of $X^\bot ({\mathfrak {x}})$ where
  $$\begin{aligned} X^\bot (\theta , w) = \Pi _\bot \sum _{k = 0}^{N+m} T_{A_{m - k}(\theta )[w]} \partial _x^{m - k} \, . \end{aligned}$$
(ii) Assume that ${\mathcal {M}}^\bot ({\mathfrak {x}}) = \sum _{k = 0}^{N + m} \lambda _{m - k}({\mathfrak {x}}) \partial _x^{m - k} w$ is of class ${\mathcal {OF}}^2(m, N)$.
(ii1) ${\mathcal {M}}^\bot$ is said to be of class ${\mathcal {OF}}^2_w(m, N)$ if it is linear with respect to *w*. More precisely, there exist $0< \delta \equiv \delta (N) < 1$ and $0< \varepsilon _0 \equiv \varepsilon _0(N) < 1$ with the property that for any $0 \le k \le m + N$,
  $$\begin{aligned} \lambda _{m - k} : {\mathbb {T}}^{S_+} \times B_{S_+}(\delta ) \times [0, \varepsilon _0] \rightarrow {\mathbb {R}}, \, (\theta , y, \varepsilon ) \mapsto \lambda _{m-k}(\theta , y) \equiv \lambda _{m-k}(\theta , y, \varepsilon ) \end{aligned}$$
  is $C^\infty$-smooth and bounded.
(ii2) ${\mathcal {M}}^\bot$ is said to be of class ${\mathcal {OF}}^2_{ww}(m, N)$ if it is quadratic with respect to *w* and independent of *y*. More precisely, there exist an integer $\sigma _N \ge 0$, $0< \varepsilon _0 \equiv \varepsilon _0(N) < 1$, and for any $0 \le k \le m + N$ a $C^\infty -$smooth map
  $$\begin{aligned} \Lambda _{m -k} : {\mathbb {T}}^{S_+} \times [0, \varepsilon _0] \rightarrow {{\mathcal {B}}}(H^{\sigma _N}_\bot ({\mathbb {T}}_1), {\mathbb {R}}), \, \theta \mapsto \Lambda _{m -k} (\theta ) \equiv \Lambda _{m -k} (\theta , \varepsilon ), \end{aligned}$$
  so that $$\lambda _{m - k}({\mathfrak {x}}) = \Lambda _{m - k}(\theta )[w]$$.
(iii) Assume that ${{\mathcal {R}}}$ is a smoothing vector field of class ${\mathcal {OS}}^2(N)$.
(iii1) ${{\mathcal {R}}}$ is said to be of class ${\mathcal {OS}}_{w}^2(N)$ if ${{\mathcal {R}}}({\mathfrak {x}})$ of the form ${{\mathfrak {R}}}(\theta , y)[w]$ with ${\mathfrak {R}}$ having the following property: there is an integer $s_N \ge 0$ so that for any $s \ge s_N$, there exist $0< \delta \equiv \delta (s, N) < 1$ and $0< \varepsilon _0 \equiv \varepsilon _0(s, N) < 1$ with the property that
  $$\begin{aligned}&{{\mathfrak {R}}} : {\mathbb {T}}^{S_+} \times B_{S_+}(\delta ) \times [0, \varepsilon _0] \rightarrow {{\mathcal {B}}}(H^s({\mathbb {T}}_1), H^{s + N + 1}({\mathbb {T}}_1)), (\theta , y, \varepsilon )\\&\quad \mapsto {{\mathfrak {R}}}(\theta , y) \equiv {{\mathfrak {R}}}(\theta , y; \varepsilon ) \end{aligned}$$
  is $C^\infty$-smooth, bounded, and small of order one. In the sequel, we will also write ${{\mathcal {R}}}(\theta , y)[w]$ for ${{\mathfrak {R}}}(\theta , y)[w]$.
(iii2) ${{\mathcal {R}}}$ is said to be of class ${\mathcal {OS}}^2_{ww}(N)$ if ${{\mathcal {R}}}$ is quadratic with respect to *w* and independent of *y*. More precisely, ${{\mathcal {R}}}({\mathfrak {x}})$ is of the form ${{\mathfrak {R}}}(\theta )[w, w]$ with ${\mathfrak {R}}$ having the following property: there is an integer $s_N \ge 0$ so that for any $s \ge s_N$ there exists $0< \varepsilon _0 \equiv \varepsilon _0(s, N) < 1$ with the property that
  $$\begin{aligned} {{\mathfrak {R}}} : {\mathbb {T}}^{S_+} \times [0, \varepsilon _0] \rightarrow {{\mathcal {B}}}_2\big (H^s_\bot ({\mathbb {T}}_1) , H^{s + N + 1}_\bot ({\mathbb {T}}_1)\big ), \, (\theta , \varepsilon ) \mapsto {{\mathfrak {R}}}(\theta ) \equiv {{\mathfrak {R}}}(\theta , \varepsilon ) \end{aligned}$$
  is $C^\infty$-smooth and bounded. In the sequel, we will often write ${{\mathcal {R}}}(\theta )[w, w]$ instead of ${{\mathfrak {R}}}(\theta )[w, w]$.

#### Remark 3.3

For any $N \in {\mathbb {N}}$ and $m \in {\mathbb {Z}}$, the following inclusions between the classes of vector fields introduced above hold:

$$\begin{aligned} \begin{aligned}&{\mathcal {OF}}(m, N) \subseteq {\mathcal {OB}}(m, N), \qquad \ {\mathcal {OF}}^p(m, N) \subseteq {\mathcal {OB}}^p(m, N)\,, \\&{{\mathcal {OF}}}^2_w(m, N) \subseteq {{\mathcal {OB}}}^2_{w}(m, N), \quad {{\mathcal {OF}}}^2_{ww}(m, N) \subseteq {{\mathcal {OB}}}^2_{ww}(m, N)\,. \quad \end{aligned} \end{aligned}$$

These inclusions hold since by (2.1) the operator $T_\lambda$ of para-multiplication with any constant $\lambda \in {\mathbb {R}}$ satisfies $\Pi _\bot T_\lambda = \lambda \Pi _\bot$.

For notational convenience, we will often not distinguish between a vector field *X* of the form $(0, 0, X^\bot )$ and its normal component $X^\bot$. Given two vector fields *X* and *Y*, defined on a subset of ${\mathcal {E}}$ and depending on the parameters $\varepsilon$ and $\mu$, we write

$$\begin{aligned} X = Y + {{\mathcal {O}}}_1 + \cdots + {{\mathcal {O}}}_n \end{aligned}$$

if for any $1 \le j \le n,$ there exists a vector field $X_j \in {{\mathcal {O}}}_j$ so that $X = Y + X_1 + \cdots + X_n$. Here ${{\mathcal {O}}}_j$ denotes any of the classes of vector fields introduced above.

### Commutators

#### Lemma 3.1

(Commutators I). Let *N*, *p*, and *q* be in ${\mathbb {N}}$.

(i) For any smoothing vector fields ${{\mathcal {R}}}$, ${{\mathcal {Q}}} \in {{\mathcal {OS}}}(N)$, the commutator $[{{\mathcal {R}}}, {{\mathcal {Q}}}]$ is also in ${{\mathcal {OS}}}(N)$.
(ii) For any vector fields ${{\mathcal {R}}} {\in } {{\mathcal {OS}}}^p(N)$ and ${{\mathcal {Q}}} \in {{\mathcal {OS}}}^q(N)$, one has $[{{\mathcal {R}}}, {{\mathcal {Q}}}] \in {{\mathcal {OS}}}^{p + q - 1}(N)$

#### Proof

The two items follow from Definition 3.3 (smoothing vector fields) and the definition (1.34) of the commutator. $\square$

#### Lemma 3.2

(Commutators II). Let *N*, *p*, $q \in {\mathbb {N}}$ and $m \in {\mathbb {Z}}$.

If $X= (0, 0, X^\bot )$ with $X^\bot \in {{\mathcal {O}} B}(m, N)$ and ${{\mathcal {R}}} = ({\mathcal {R}}^{(\theta )}, {\mathcal {R}}^{(y)}, {\mathcal {R}}^\bot ) \in {{\mathcal {OS}}}(N)$, then

3.3 $$\begin{aligned}&{[}(0, 0, X^\bot ), \, {{\mathcal {R}}}] = (0,0, \, {\mathcal {C}}^\bot _{[X, {\mathcal {R}}]}) + {{\mathcal {R}}}_{[X, {\mathcal {R}}]}\, , \qquad {\mathcal {C}}^\bot _{[X, {\mathcal {R}}]} \in {{\mathcal {OB}}}(m, N)\, , \nonumber \\&\quad {{\mathcal {R}}}_{[X, {\mathcal {R}}]} \in {{\mathcal {OS}}}(N - m). \end{aligned}$$

If $X^\bot \in {{\mathcal {O}} B}^p(m, N)$ and ${{\mathcal {R}}} \in {{\mathcal {OS}}}^q(N)$, then ${\mathcal {C}}^\bot _{[X, {\mathcal {R}}]} \in {{\mathcal {OB}}}^{p + q -1}(m, N)$ and ${{\mathcal {R}}}_{[X, {\mathcal {R}}]} \in {{\mathcal {OS}}}^{p + q - 1}(N - m)$.

#### Proof

By (3.1), *X* can be written as $X({\mathfrak {x}}) := \sum _{k = 0}^{N + m} X_k({\mathfrak {x}})$ where

$$\begin{aligned} X_k({\mathfrak {x}}) = \big (0,0, \, \Pi _\bot T_{a_{m -k}({\mathfrak {x}})} \partial _x^{m - k} w \big ), \qquad \forall \, 0 \le k \le N+m\, . \end{aligned}$$

For any $0 \le k \le N+m$, the commutator $[X_k, {{\mathcal {R}}}] ({\mathfrak {x}})= d X_k({\mathfrak {x}})[{{\mathcal {R}}}({\mathfrak {x}})] - d {{\mathcal {R}}}({\mathfrak {x}}) [X_k({\mathfrak {x}})]$ can be computed as

$$\begin{aligned} {[}X_k, {{\mathcal {R}}}]({\mathfrak {x}}) = (0 ,0, \Pi _\bot T_{a_{m - k}({\mathfrak {x}})} \partial _x^{m - k} {{\mathcal {R}}}^\bot ({\mathfrak {x}})) {+} (0,0, \Pi _\bot T_{d a_{m - k}({\mathfrak {x}})[{{\mathcal {R}}}({\mathfrak {x}})]} \partial _x^{m - k} w) - d {{\mathcal {R}}}({\mathfrak {x}}) [X_k({\mathfrak {x}})] , \end{aligned}$$

where ${\mathcal {R}} = ({\mathcal {R}}^{(\theta )}, {\mathcal {R}}^{(y)}, {\mathcal {R}}^\bot )$. Note that $d {{\mathcal {R}}}({\mathfrak {x}}) [X_k({\mathfrak {x}})] \in {{\mathcal {OS}}}(N - m)$ and that for any $0 \le k \le N+m$,

$$\begin{aligned} \big (0 ,0, \, \Pi _\bot T_{a_{m - x}({\mathfrak {x}})} \partial _x^{m - k} {{\mathcal {R}}}^\bot ({\mathfrak {x}}) \big ) \in {{\mathcal {OS}}}(N - (m - k)) \subseteq {{\mathcal {OS}}}(N - m). \end{aligned}$$

Formula (3.3) then follows by setting ${\mathcal {C}}^\bot _{[X, {\mathcal {R}}]}({\mathfrak {x}}) := \Pi _\bot \sum _{k = 0}^{m + N} T_{d a_{m - k}({\mathfrak {x}})[{{\mathcal {R}}}({\mathfrak {x}})]} \partial _x^{m - k} w$, and

$$\begin{aligned} {{\mathcal {R}}}_{[X, {\mathcal {R}}]}({\mathfrak {x}}) := \sum _{k = 0}^{m + N}\big (0, 0, \, \Pi _\bot T_{a_{m - k}({\mathfrak {x}})} \partial _x^{m - k} {{\mathcal {R}}}^\bot ({\mathfrak {x}}) \big ) - d {{\mathcal {R}}}({\mathfrak {x}}) [X_k({\mathfrak {x}})]\,. \end{aligned}$$

The remaining part of the lemma is proved by using similar arguments. $\square$

#### Lemma 3.3

(Commutators III). Let *N*, *p*, $q \in {\mathbb {N}}$, *m*, $m' \in {\mathbb {Z}}$, and let $m_* := \max \{ m + m' - 1, \, m, \, m' \}$. For any $X^\bot \in {{\mathcal {OB}}}(m, N)$ and $Y^\bot \in {{\mathcal {OB}}}(m', N)$, one has

$$\begin{aligned} {[}X^\bot , Y^\bot ] = {\mathcal {C}}^\bot _{[X^\bot , Y^\bot ]}+ {{\mathcal {R}}}^\bot _{[X^\bot , Y^\bot ]} \, , \qquad {{\mathcal {C}}}^\bot _{[X^\bot , Y^\bot ]} \in {{\mathcal {OB}}}(m_*, N), \quad {{\mathcal {R}}}^\bot _{[X^\bot , Y^\bot ]} \in {{\mathcal {OS}}}(N). \end{aligned}$$

If in fact $X^\bot \in {{\mathcal {OB}}}^p(m, N)$ and $Y^\bot \in {{\mathcal {OB}}}^q(m', N)$, then

$$\begin{aligned} {{\mathcal {C}}}^\bot _{[X^\bot , Y^\bot ]} \in {{\mathcal {OB}}}^{p + q - 1}(m_*, N), \qquad {{\mathcal {R}}}^\bot _{[X^\bot , Y^\bot ]} \in {{\mathcal {OS}}}^{p + q - 1}(N). \end{aligned}$$

#### Proof

By formula (3.1), $X^\bot \in {{\mathcal {OB}}}(m, N)$ and $Y^\bot \in {{\mathcal {OB}}}(m', N)$ are of the form

$$\begin{aligned} X^\bot ({\mathfrak {x}}) =\Pi _\bot \sum _{k = 0}^{N + m} T_{a_{m - k}({\mathfrak {x}})} \partial _x^{m - k} w\, , \qquad Y^\bot ({\mathfrak {x}}) =\Pi _\bot \sum _{k = 0}^{N + m'} T_{b_{m' - k}({\mathfrak {x}})} \partial _x^{m' - k} w\, . \end{aligned}$$

With $X^\bot = \sum _{k = 0}^{N + m} X^\bot _k$ and $Y^\bot = \sum _{j = 0}^{N + m'} Y^\bot _j$ one gets

$$\begin{aligned}{}[X^\bot , Y^\bot ] = \sum _{k = 0}^{N + m}\sum _{j = 0}^{N + m'} [X^\bot _k, Y^\bot _j] \end{aligned}$$

where

$$\begin{aligned}&\quad X^\bot _k({\mathfrak {x}}) = \Pi _\bot T_{a_{m - k}({\mathfrak {x}})} \partial _x^{m - k} w, \ \ \forall \, 0 \le k \le N+m\, , \\&\qquad Y_j^\bot ({\mathfrak {x}}) := \Pi _\bot T_{b_{m' - j}({\mathfrak {x}})} \partial _x^{m' - j} w, \ \ \forall \, 0 \le j \le N+m'. \end{aligned}$$

To compute $[X^\bot _k, Y^\bot _j]$ for *k*, *j* in the corresponding ranges, for notational convenience we let

$$\begin{aligned} X^\bot _* := X^\bot _k, \quad Y^\bot _* := Y^\bot _j, \quad a({\mathfrak {x}}) := a_{m - k}({\mathfrak {x}}), \quad b({\mathfrak {x}}) := b_{m' - j}({\mathfrak {x}}), \quad n := m - k, \quad n' := m' - j. \end{aligned}$$

One computes

$$\begin{aligned} {[} X^\bot _*, Y^\bot _* ] = [\Pi _\bot T_{a} \partial _x^{n}\,,\, \Pi _\bot T_{b} \partial _x^{n'}]_{lin} w + \Pi _\bot T_{d_\bot a({\mathfrak {x}})[Y_*^\bot ({\mathfrak {x}})]} \partial _x^{n} w - \Pi _\bot T_{d_\bot b({\mathfrak {x}})[X_*^\bot ({\mathfrak {x}})]} \partial _x^{n'} w . \end{aligned}$$

Using the formula

$$\begin{aligned} \Pi _\bot T_{a} \partial _x^{n} \circ \Pi _\bot T_{b} \partial _x^{n'} = \Pi _\bot \circ \big ( T_{a} \partial _x^{n} \circ T_{b} \partial _x^{n'} + T_{a} \partial _x^{n} \circ ( \Pi _\bot - \text {Id}) T_{b} \partial _x^{n'} \big ), \end{aligned}$$

and the corresponding one for $\Pi _\bot T_{b} \partial _x^{n'} \circ \Pi _\bot T_{a} \partial _x^{n}$, one obtains $[X^\bot _*, \, Y^\bot _*] = {{\mathcal {C}}}^\bot _1 + {{\mathcal {R}}}^\bot _1$ where

$$\begin{aligned} {{\mathcal {C}}}^\bot _1 ({\mathfrak {x}}) := \Pi _\bot [T_{a} \partial _x^{n}, \, T_b \partial _x^{n'}]_{lin} w + \Pi _\bot T_{d_\bot a({\mathfrak {x}})[Y_*^\bot ({\mathfrak {x}})]} \partial _x^{n} w - \Pi _\bot T_{d_\bot b({\mathfrak {x}}) [X_*^\bot ({\mathfrak {x}})]} \partial _x^{n'} w \end{aligned}$$

and

$$\begin{aligned} {{\mathcal {R}}}^\bot _1({\mathfrak {x}}) := \Pi _\bot T_{a({\mathfrak {x}})} \partial _x^n \circ (\Pi _\bot - \mathrm{Id}) T_{b({\mathfrak {x}})} \partial _x^{n'} w \, - \, \Pi _\bot T_{b({\mathfrak {x}})} \partial _x^{n'} \circ (\Pi _\bot - \mathrm{Id}) T_{a({\mathfrak {x}})} \partial _x^{n} w\,. \end{aligned}$$

Since by assumption, there exist integers $s_N \ge 0$, $\sigma _N \ge 0$, so that for any $s \ge s_N$ there is $0< \delta \equiv \delta (s, N) < 1$ and $0< \varepsilon _0 \equiv \varepsilon _0(s, N) < 1$ with the property that $a, b: {{\mathcal {V}}}^{s + \sigma _N}(\delta ) \times [0, \varepsilon _0] \rightarrow H^s({\mathbb {T}}_1)$ are $C^\infty$-smooth and bounded, it then follows that

$$\begin{aligned} \Pi _\bot T_{d_\bot a({\mathfrak {x}})[ Y_* ({\mathfrak {x}})]} \partial _x^n w \in {{\mathcal {OB}}}(n, N), \qquad \Pi _\bot T_{d_\bot b({\mathfrak {x}})[ X_* ({\mathfrak {x}})]} \partial _x^{n'} w \in {{\mathcal {OB}}}(n', N), \end{aligned}$$

and, in view of Corollary 2.1, that

$$\begin{aligned} \Pi _\bot [T_a \partial _x^n, \, T_b \partial _x^{n'}]_{lin} w = {{\mathcal {OB}}}(n + n' - 1, N) + {{\mathcal {OS}}}(N) \, . \end{aligned}$$

Furthermore, since $\Pi _\bot - \mathrm{Id}$ is a smoothing operator, one concludes that ${{\mathcal {R}}}^\bot _1 \in {{\mathcal {OS}}}(N)$. Altogether, we have proved that $[X^\bot _*, \, Y^\bot _*]$ is of the form ${{\mathcal {C}}}^\bot _{[X^\bot _*, \, Y^\bot _*]} + {{\mathcal {R}}}^\bot _{[X^\bot _*, \, Y^\bot _*]}$ where

$$\begin{aligned} {{\mathcal {C}}}^\bot _{[X^\bot _*, \, Y^\bot _*]} \in {{\mathcal {OB}}}(n_*, N) , \quad n_* = \max \{n + n' - 1, n , n' \} \le m_*\, , \qquad {{\mathcal {R}}}^\bot _{[X^\bot _*, \, Y^\bot _*]} \in {{\mathcal {OS}}}(N). \end{aligned}$$

If in fact $X_*^\bot \in {{\mathcal {OB}}}^p(m, N)$ and $Y_*^\bot \in {{\mathcal {OB}}}^q(m', N)$, then *a* is small of order $p - 1$, *b* is small of order $q - 1$ and it follows that

$$\begin{aligned} \Pi _\bot T_{d_\bot a({\mathfrak {x}})[ Y_*^\bot ({\mathfrak {x}})]} \partial _x^n w \in {{\mathcal {OB}}}^{p + q - 1}(n, N), \qquad \Pi _\bot T_{d_\bot b({\mathfrak {x}})[ X_*^\bot ({\mathfrak {x}})]} \partial _x^{n'} w \in {{\mathcal {OB}}}^{p + q - 1}(n', N)\,, \\ \Pi _\bot [T_a \partial _x^n, \, T_b \partial _x^{n'}]_{lin} w = {{\mathcal {OB}}}^{p + q - 1}(n + n'- 1, N) + {{\mathcal {OS}}}^{p + q - 1}(N) \,, \qquad {{\mathcal {R}}}^\bot _1 \in {{\mathcal {OS}}}^{p + q - 1}(N)\,. \end{aligned}$$

One then infers that ${{\mathcal {C}}}^\bot _{[X^\bot _*, \, Y^\bot _*]} \in {{\mathcal {OB}}}^{p + q - 1}(n_*, N)$ and ${{\mathcal {R}}}^\bot _{[X^\bot _*, \, Y^\bot _*]} \in {{\mathcal {OS}}}^{p + q - 1}(N)$. $\square$

#### Lemma 3.4

(Commutators IV). Let *N*, *p*, $q \in {\mathbb {N}}$, *m*, $m' \in {\mathbb {Z}}$, and let $m_* := \max \{ m + m' - 1, \, m, \, m' \}$.

(i) For any ${\mathcal {M}}^\bot \in {{\mathcal {OF}}}(m, N)$ and ${\mathcal {M}}'^\bot \in {{\mathcal {OF}}}(m', N)$
  $$\begin{aligned} {[}{\mathcal {M}}^\bot , \, {\mathcal {M}}'^\bot ] \in {{\mathcal {OF}}}\big (m\vee m' , N \big ). \end{aligned}$$
  If in fact ${\mathcal {M}}^\bot \in {{\mathcal {OF}}}^p(m, N)$ and ${\mathcal {M}}'^\bot \in {{\mathcal {OF}}}^q(m', N)$, then $[{\mathcal {M}}^\bot , {\mathcal {M}}'^\bot ] \in {{\mathcal {OF}}}^{p + q - 1}(m \vee m' , N )$.
(ii) For any $X^\bot \in {{\mathcal {OB}}}(m, N)$ and ${\mathcal {M}}^\bot \in {{\mathcal {OF}}}(m', N)$,
  $$\begin{aligned} {[}X^\bot , {\mathcal {M}}^\bot ] {=} {{\mathcal {C}}}^\bot _{[X^\bot , {\mathcal {M}}^\bot ]} + {{\mathcal {R}}}^\bot _{[X^\bot , {\mathcal {M}}^\bot ]}, \quad {{\mathcal {C}}}^\bot _{[X^\bot , {\mathcal {M}}^\bot ]} \in {{\mathcal {OB}}}(m_* ,N), \qquad {{\mathcal {R}}}^\bot _{[X^\bot , {\mathcal {M}}^\bot ]} \in {{\mathcal {OS}}}(N). \end{aligned}$$
  If $X^\bot \in {{\mathcal {OB}}}^p(m, N)$ and ${\mathcal {M}}^\bot \in {{\mathcal {OF}}}^q(m', N)$, then
  $$\begin{aligned} {{\mathcal {C}}}^\bot _{[X^\bot , {\mathcal {M}}^\bot ]} \in {{\mathcal {OB}}}^{p + q - 1}(m_*, N),\qquad {{\mathcal {R}}}^\bot _{[X^\bot , {\mathcal {M}}^\bot ]} \in {{\mathcal {OS}}}^{p + q - 1}(N). \end{aligned}$$
(iii) For any ${\mathcal {M}}= (0, 0, {\mathcal {M}}^\bot )$ with ${\mathcal {M}}^\bot \in {{\mathcal {OF}}}(m, N)$ and ${\mathcal {R}} = \big ( {\mathcal {R}}^{(\theta )}, {\mathcal {R}}^{(y)}, {\mathcal {R}}^\bot \big ) \in {{\mathcal {OS}}}(N)$
  $$\begin{aligned} {[}{\mathcal {M}}, {\mathcal {R}}] = (0,0, {{\mathcal {C}}}^\bot _{[{\mathcal {M}}, {\mathcal {R}}^]}) + {{\mathcal {R}}}_{[{\mathcal {M}}, {\mathcal {R}}]}, \qquad {{\mathcal {C}}}^\bot _{[{\mathcal {M}}, {\mathcal {R}}]} \in {{\mathcal {OF}}}(m, N), \qquad {{\mathcal {R}}}_{[{\mathcal {M}}, {\mathcal {R}}]} \in {{\mathcal {OS}}}(N - m) \end{aligned}$$
  If ${\mathcal {M}}^\bot \in {{\mathcal {OF}}}^p(m, N)$ and ${\mathcal {R}} \in {{\mathcal {OS}}}^q(N)$, then ${{\mathcal {C}}}^\bot _{[{\mathcal {M}}, {\mathcal {R}}]} \in {{\mathcal {OF}}}^{p + q - 1}(m, N)$ and ${{\mathcal {R}}}_{[M, {\mathcal {R}}]} \in {{\mathcal {OS}}}^{p + q - 1}(N)$.

#### Proof

Since the claims of the lemma follow by arguing as in the proofs of Lemma 3.2 and Lemma 3.3, the details of the proofs are omitted. $\square$

### Flows of para-differential vector fields

In this subsection we study the flow of para-differential vector fields of the form $Y = (0, 0, \, Y^\bot )$ with

3.4 $$\begin{aligned} \begin{aligned} Y^\bot ({\mathfrak {x}}) = \Pi _\bot T_{a_{m}({\mathfrak {x}})} \partial _x^{ m} w \in {{\mathcal {OB}}}^p(m, N), \qquad N, \, p \ge 1\,, \ m \le 0\, . \end{aligned} \end{aligned}$$

By Definition  3.1, there are integers $s_N \ge 0$, $\sigma _N\ge 0$ so that for any $s \ge s_N$ there exist $0< \delta \equiv \delta (s, N) < 1$ and $0< \varepsilon _0 \equiv \varepsilon _0(s, N) < 1$ with the property that

$$\begin{aligned} a_{m} : {{\mathcal {V}}}^{s + \sigma _N}(\delta ) \times [0, \varepsilon _0] \rightarrow H^s({\mathbb {T}}_1), \, ({\mathfrak {x}}, \varepsilon ) \mapsto a_{m}({\mathfrak {x}}) \equiv a_{m}({\mathfrak {x}}, \varepsilon ) \end{aligned}$$

is $C^\infty -$smooth and bounded. In the sequel, we will often *tacitly* increase $s_N$, $\sigma _N$ and decrease $\delta \equiv \delta (s, N)$, $\varepsilon _0\equiv \varepsilon _0(s, N)$, whenever needed.

Denote by $\Phi _Y (\tau , \cdot )$ the flow associated with *Y*. By the standard ODE theorem in Banach spaces, for any $s \ge s_N$, there exist $0< \delta \equiv \delta (s, N) < 1$, and $0 < \varepsilon _0 \equiv \varepsilon _0(s, N) \ll \delta$, so that for any $-1 \le \tau \le 1$,

3.5 $$\begin{aligned} \Phi _Y(\tau , \cdot ) \in C^\infty _b \big ({{\mathcal {V}}}^s(\delta ) \times [0, \varepsilon _0], \, {{\mathcal {V}}}^s(2 \delta ) \big )\, . \end{aligned}$$

It then follows that for $-1 \le \tau \le 1$ and ${\mathfrak {x}} \in {\mathcal {V}}^s(\delta ),$ one has $\Phi _Y(- \tau , \Phi _Y(\tau , {\mathfrak {x}})) = {\mathfrak {x}}$.

#### Remark 3.4

For notational convenience, $\Phi _Y(- \tau , \cdot )$ is referred to as the inverse of $\Phi _Y(\tau , \cdot )$ and we write $\Phi _Y(\tau , \cdot )^{-1} = \Phi _Y(- \tau , \cdot )$. In particular, $\Phi _Y(1, \cdot )^{-1} = \Phi _Y(- 1, \cdot )$. Using our convention of tacitly decreasing $\delta$ and $\varepsilon _0$, if needed, $\Phi _Y(\tau , \cdot )^{-1}$ is defined for $({\mathfrak {x}}, \varepsilon ) \in {{\mathcal {V}}}^s(\delta ) \times [0, \varepsilon _0]$. More generally, a similar convention is used for diffeomorphisms between neighborhoods of ${\mathbb {T}}^{S_+} \times 0 \times 0$ in ${\mathcal {E}}_s$ throughout the paper.

The following lemma provides a para-differential expansion of the flow $\Phi _Y (\tau , \cdot )$.

#### Lemma 3.5

Let *N*, $p \in {\mathbb {N}}$ and assume that the normal component $Y^\bot$ of $Y = (0, 0, \, Y^\bot )$ satisfies (3.4). Then for any $-1 \le \tau \le 1$, $\Phi _Y(\tau , {\mathfrak {x}})$ admits an expansion of the form

$$\begin{aligned} \Phi _Y(\tau , {\mathfrak {x}}) = {\mathfrak {x}} + \big (0, 0, \, {\Upsilon }^\bot (\tau , {\mathfrak {x}}) + {{\mathcal {R}}}^\bot _{N}(\tau , {\mathfrak {x}}) \big ) \end{aligned}$$

where

$$\begin{aligned} {\Upsilon }^\bot (\tau , {\mathfrak {x}}) = \Pi _\bot \sum _{k = 0 }^{N +m} T_{b_{m - k}(\tau , {\mathfrak {x}})}\partial _x^{m - k} w \in {{\mathcal {OB}}}^p(m, N)\, , \qquad {{\mathcal {R}}}^\bot _{N}(\tau , {\mathfrak {x}}) \in {{\mathcal {OS}}}^{2 p - 1}(N) \, . \end{aligned}$$

#### Proof

The normal component $\Phi _Y^\bot (\tau , {\mathfrak {x}})$ of the flow $\Phi _Y(\tau , {\mathfrak {x}})$ satisfies the integral equation

3.6 $$\begin{aligned} \Phi _{Y}^\bot (\tau , {\mathfrak {x}}) = w + \int _0^\tau Y^\bot (\Phi _Y(t, {\mathfrak {x}}))\, d t\, , \qquad \forall \, -1 \le \tau \le 1\,. \end{aligned}$$

To solve it, we make the ansatz that $\Phi _Y^\bot (\tau , {\mathfrak {x}})$ admits an expansion of the form

3.7 $$\begin{aligned} \Phi _Y^\bot (\tau , {\mathfrak {x}}) = w + {\Upsilon }^\bot (\tau , {\mathfrak {x}}) + {{\mathcal {R}}}^\bot _{N}(\tau , {\mathfrak {x}})\, , \quad {\Upsilon }^\bot (\tau , {\mathfrak {x}}) = \Pi _\bot \sum _{k = 0 }^{N +m} T_{b_{m - k}(\tau , {\mathfrak {x}})} \partial _x^{m - k} w \, ,\qquad \end{aligned}$$

with the property that there exist $s_N \ge 0$, $\sigma _N \ge 0$ so that the following holds: for any $s \ge s_N$, there exist $0< \delta \equiv \delta (s, N) < 1$ and $0< \varepsilon _0 \equiv \varepsilon _0(s, N) < 1$ so that for any $-1 \le \tau \le 1$ and $0 \le k \le N+m$,

3.8 $$\begin{aligned}&b_{m-k}(\tau , \cdot ) \in C^\infty _b \big ( {{\mathcal {V}}}^{s + \sigma _N}(\delta ) \times [0, \varepsilon _0] , \, H^s({\mathbb {T}}_1) \big ), \ \ b_{m - k} \ \text {small of order } p - 1\,,\nonumber \\&\quad \qquad {{\mathcal {R}}}^\bot _{N}(\tau , \cdot ) \in {{\mathcal {OS}}}^p(N)\,. \end{aligned}$$

To determine $\big ( b_{m-k}\big )_{0 \le k \le N+m}$ and ${{\mathcal {R}}}^\bot _{N}$, in terms of the coefficient $a_{m}$ of $Y^\bot$ in (3.4), we compute the expansion of the right hand side of the Eq. (3.6) by substituting the ansatz (3.7) into the integrand $Y^\bot (\Phi _{Y}(t, {\mathfrak {x}}))$. In view of definition (3.4) of $Y^\bot$, one gets for any $-1 \le t \le 1$,

3.9 $$\begin{aligned} Y^\bot (\Phi _Y(t, {\mathfrak {x}}))= & {} \Pi _\bot T_{a_{m}(\Phi _Y(t, {\mathfrak {x}}))} \partial _x^{ m} \Phi _Y^\bot (t, {\mathfrak {x}}) \nonumber \\= & {} \Pi _\bot T_{a_{m}(\Phi _Y(t, {\mathfrak {x}}))} \partial _x^{m} \Big (w + \Pi _\bot \sum _{k = 0 }^{N +m} T_{b_{m-k}(t, {\mathfrak {x}})} \partial _x^{m-k} w + {{\mathcal {R}}}^\bot _{N}(t, {\mathfrak {x}}) \Big ) \,.\nonumber \\ \end{aligned}$$

Using that $\Pi _\bot - \mathrm{Id}$ is a smoothing operator and that

$$\begin{aligned} \Phi _Y(\tau , \cdot ) \in C^\infty _b\big ( {{\mathcal {V}}}^s(\delta )\times [0, \varepsilon _0] , \, {{\mathcal {V}}}^s(2 \delta ) \big ) \end{aligned}$$

one gets

3.10 $$\begin{aligned} \begin{aligned}&\Pi _\bot T_{a_{m}(\Phi _Y(t, {\mathfrak {x}}))} \partial _x^{m} (\Pi _\bot - \mathrm{Id}) \sum _{k = 0}^{N +m} T_{b_{m-k}(t, {\mathfrak {x}})} \partial _x^{m-k} w \, \in {{\mathcal {OS}}}^{2 p - 1}(N) {\mathop {\subseteq }\limits ^{p \ge 1}} {{\mathcal {OS}}}^p(N)\,, \\&\Pi _\bot \sum _{k = N+1+2m}^{N +m} T_{a_{m}(\Phi _Y(t, {\mathfrak {x}}))} \partial _x^{m} T_{b_{m-k}(t, {\mathfrak {x}})} \partial _x^{m-k} w \in {{\mathcal {OS}}}^{2 p - 1}(N) {\mathop {\subseteq }\limits ^{p \ge 1}} {{\mathcal {OS}}}^p(N) \end{aligned} \end{aligned}$$

where we recall that $m \le 0$ and that by our convention, a sum of terms over an empty index set equals 0. Moreover, by increasing $s_N, \sigma _N$ if needed, it follows that for any $s \ge s_N$ and $-1 \le t \le 1,$ the map $A(t, {\mathfrak {x}}) := \Pi _\bot T_{a_{m}(\Phi _Y(t, {\mathfrak {x}}))} \partial _x^{m}$ satisfies (after decreasing $\delta$ and $\varepsilon _0$ if necessary)

$$\begin{aligned} A(t, \cdot ) \in C^\infty _b\big ( {{\mathcal {V}}}^{s+ \sigma _N}(\delta ) \times [0, \varepsilon _0], \ {{\mathcal {B}}}(H_\bot ^{s + N + 1}({\mathbb {T}}_1)) \big ) \end{aligned}$$

and hence in view of (3.8),

3.11 $$\begin{aligned} A(t, \cdot )[{{\mathcal {R}}}^\bot _{N}(t, \cdot )] \in {{\mathcal {OS}}}^{2 p - 1}(N) {\mathop {\subseteq }\limits ^{p \ge 1}} {{\mathcal {OS}}}^p(N) \, . \end{aligned}$$

In view of (3.10)–(3.11), we rewrite (3.9) as

3.12 $$\begin{aligned} Y^\bot (\Phi _Y(t, {\mathfrak {x}}))&=\Pi _\bot T_{a_{m}(\Phi _Y(t, {\mathfrak {x}}))} \partial _x^{m}w + \Pi _\bot \sum _{ k= 0}^{N + 2m} T_{a_{m}(\Phi _Y(t, {\mathfrak {x}}))} \partial _x^{m} T_{b_{m-k}(t, {\mathfrak {x}})} \partial _x^{m-k} w + {{\mathcal {OS}}}^p(N).\nonumber \\ \end{aligned}$$

Since $a_{m}$ and $b_{m-k}$ are small of order $p - 1$ (cf. (3.8)), it follows from Lemma 2.5 that for any $0 \le k \le N + 2m$, the term $T_{a_{m}(\Phi _Y(t, {\mathfrak {x}}))} \partial _x^{m} T_{b_{m-k}(t, {\mathfrak {x}})} \partial _x^{m-k} w$ has an expansion of the form

3.13 $$\begin{aligned} T_{a_{m}(\Phi _{Y}(t, {\mathfrak {x}})) b_{m-k}(t, {\mathfrak {x}})} \partial _x^{ 2m - k } w + \sum _{j = 1}^{N + 2m - k} K(j, m) T_{a_{m}(\Phi _{Y}(t, {\mathfrak {x}})) \partial _x^j b_{m-k}(t, {\mathfrak {x}})} \partial _x^{ 2m - k - j} w + {{\mathcal {OS}}}^{2p -1}(N)\nonumber \\ \end{aligned}$$

with the constants *K*(*j*, *m*) given as in Lemma 2.5, implying that

3.14 $$\begin{aligned}&\Pi _\bot \sum _{ k=0}^{N + 2m} T_{a_{m}(\Phi _Y(t, {\mathfrak {x}}))} \partial _x^{m} T_{b_{m - k}(t, {\mathfrak {x}})} \partial _x^{m-k} w = \Pi _\bot \sum _{k=0}^{N +2m} T_{a_{m}(\Phi _{Y}(t, {\mathfrak {x}})) b_{m-k}(t, {\mathfrak {x}})} \partial _x^{ 2m - k} w \nonumber \\&\qquad + \Pi _\bot \sum _{k=0}^{N +2m} \sum _{j=1}^{N+2m - k} K(j, m) T_{a_{m}(\Phi _{Y}(t, {\mathfrak {x}})) \partial _x^j b_{m - k}(t, {\mathfrak {x}})} \partial _x^{ 2m - k - j} w + {{\mathcal {OS}}}^{2 p - 1}(N) \nonumber \\&\quad = \Pi _\bot \sum _{i = 0}^{N +2m} T_{g_{2m - i}(t, {\mathfrak {x}})} \partial _x^{2m - i} w + {{\mathcal {OS}}}^{2 p - 1}(N)\, , \end{aligned}$$

where $g_{2m}(t, {\mathfrak {x}}) = a_{m}(\Phi _{Y}(t, {\mathfrak {x}})) b_{m}(t, {\mathfrak {x}})$ and for any $1 \le i \le N + 2m$,

3.15 $$\begin{aligned} g_{2m-i}(t, {\mathfrak {x}}) = a_{m}(\Phi _{Y}(t, {\mathfrak {x}})) b_{m-i}(t, {\mathfrak {x}}) + \sum _{k= 1}^{i-1} K(i-k, m) a_{m}(\Phi _{Y}(t, {\mathfrak {x}})) \partial _x^{i-k} b_{m - k}(t, {\mathfrak {x}}) \,.\nonumber \\ \end{aligned}$$

Combining (3.6)–(3.15) then yields the following identity,

$$\begin{aligned} \begin{aligned} \Pi _\bot \sum _{k = 0 }^{N +m} T_{b_{m - k}(\tau , {\mathfrak {x}})} \partial _x^{m - k} w&= \Pi _\bot \big ( \int _{0}^\tau T_{a_{m}(\Phi _Y(t, {\mathfrak {x}}))} d t \big ) \, \partial _x^{m}w + \Pi _\bot \big ( \int _{0}^\tau T_{a_{m}(\Phi _Y(t, {\mathfrak {x}})) b_m(t, {\mathfrak {x}})} d t \big ) \, \partial _x^{2m}w \\&+ \Pi _\bot \sum _{i = 1}^{N +2m} \big (\int _0^\tau T_{g_{2m - i}(t, {\mathfrak {x}})} d t\big ) \, \partial _x^{2m - i} w + {{\mathcal {OS}}}^{2 p - 1}(N)\, . \end{aligned} \end{aligned}$$

Let us first consider the case where $m \le -1$. We then require that the coefficients $b_{m-k}$, $0 \le k \le N+m$, satisfy the following system of equations,

3.16 $$\begin{aligned} \begin{aligned} b_{m}(\tau , {\mathfrak {x}})&= \int _0^\tau a_{m}(\Phi _{Y}(t, {\mathfrak {x}}))\, d t, \qquad \qquad \ \ \ b_{m - k}(\tau , {\mathfrak {x}}) = 0, \quad \forall \, 1 \le k \le |m| -1, \\ b_{2m}(\tau , {\mathfrak {x}})&= \int _0^\tau a_{m}(\Phi _Y(t, {\mathfrak {x}})) b_m(t, {\mathfrak {x}}) \, dt, \\ b_{m - k}(\tau , {\mathfrak {x}})&= \int _0^\tau g_{m - k}(t, {\mathfrak {x}})\, d t, \ \ \forall \, |m| + 1 \le k \le N + 2m . \end{aligned} \end{aligned}$$

Since for any $|m| + 1 \le k \le N + 2m$, $g_{m-k}$ only depends on $b_{m-k'}$ with $k' \le k + m \le k -1$ (cf. (3.15)), the coefficients $b_{m-k}$ are determined inductively in terms of $a_{m}$. One then verifies that the properties of the coefficients $b_{m-k}$, stated in ansatz (3.8), are satisfied. The remainder ${{\mathcal {R}}}^\bot _{N}$ then satisfies the following integral equation

3.17 $$\begin{aligned} {{\mathcal {R}}}^\bot _{N}(\tau , {\mathfrak {x}}) = {{\mathcal {Q}}}^\bot _N(\tau , {\mathfrak {x}}) + \int _0^\tau A(t, {\mathfrak {x}})[{{\mathcal {R}}}^\bot _{N}(t, {\mathfrak {x}})] d t\, , \end{aligned}$$

where ${{\mathcal {Q}}}^\bot _{N}(\tau , \cdot ) \in {{\mathcal {OS}}}^{2 p- 1}(N)$ is given by the sum of the two terms in (3.10) and the operator $A(t, {\mathfrak {x}})$ is defined in (3.11). By increasing $s_N$ if needed, it follows that for any $s \ge s_N$,

$$\begin{aligned} \Vert {{\mathcal {R}}}^\bot _N(\tau , {\mathfrak {x}}) \Vert _{s + N + 1} \le \sup _{\tau \in [- 1, 1]} \Vert {{\mathcal {Q}}}^\bot _N(\tau , {\mathfrak {x}}) \Vert _{s + N +1} + \int _0^\tau \Vert A(t, {\mathfrak {x}}) \Vert _{{{\mathcal {B}}}(H_\bot ^{s + N + 1}({\mathbb {T}}_1))} \Vert {{\mathcal {R}}}^\bot _N(t, {\mathfrak {x}})\Vert _{s + N +1}\, d t \end{aligned}$$

and hence by the Gronwall Lemma, one infers that ${{\mathcal {R}}}^\bot _N$ satisfies

$$\begin{aligned} \Vert {{\mathcal {R}}}^\bot _N(\tau , {\mathfrak {x}}) \Vert _{s + N +1} \lesssim _{s, N} \mathrm{exp}\big ( \int _{-1}^1 \Vert A(t, {\mathfrak {x}}) \Vert _{{{\mathcal {B}}}(H_\bot ^{s + N + 1}({\mathbb {T}}_1))}\, d t \big )\sup _{t \in [- 1, 1]} \Vert {{\mathcal {Q}}}^\bot _N(t, {\mathfrak {x}}) \Vert _{s + N +1}\, , \end{aligned}$$

implying that $\Vert {{\mathcal {R}}}^\bot _N(\tau , {\mathfrak {x}}) \Vert _{s + N + 1} \lesssim _{s, N} (\varepsilon + \Vert y \Vert + \Vert w \Vert _s)^{2 p - 1}$. Similar estimates hold for the derivatives of ${{\mathcal {R}}}^\bot _N$. Altogether we have shown that ${{\mathcal {R}}}^\bot _N \in {{\mathcal {OS}}}^{2 p - 1}(N)$.

Finally let us consider case $m=0$. We then require that the coefficients $b_{-k}$, $0 \le k \le N$, satisfy the following system of equations,

$$\begin{aligned} \begin{aligned} b_{0}(\tau , {\mathfrak {x}})&= \int _0^\tau a_{0}(\Phi _{Y}(t, {\mathfrak {x}}))\, d t + \int _0^\tau a_{0}(\Phi _Y(t, {\mathfrak {x}})) b_0(t, {\mathfrak {x}}) \, dt, \\&\qquad b_{ - k}(\tau , {\mathfrak {x}}) = \int _0^\tau g_{ - k}(t, {\mathfrak {x}})\, d t, \ \forall \, 1 \le k \le N . \end{aligned} \end{aligned}$$

The solution $b_0$ then reads $b_{0}(\tau , {\mathfrak {x}}) = e^{\int _0^\tau a_{0}(\Phi _Y(t, {\mathfrak {x}})) \, dt} -1$. The remaining part of the proof then follows as in the case $m \le -1$. $\square$

#### Lemma 3.6

Let *N*, $p \in {\mathbb {N}}$ and let $\Phi _Y(\tau , {\mathfrak {x}})$ denote the flow map considered in Lemma 3.5, corresponding to the vector field $Y = (0, 0, \, Y^\bot )$, with $Y^\bot ({\mathfrak {x}}) = \Pi _\bot T_{a_{m}({\mathfrak {x}})} \partial _x^{ m} w$ and $m \le 0$, satisfying (3.4). Then for any $-1\le \tau \le 1,$ $d \Phi _Y( \tau , {\mathfrak {x}})^{- 1}[\widehat{{\mathfrak {x}}}]$ admits an expansion of the form

3.18 $$\begin{aligned}&d \Phi _Y( \tau , {\mathfrak {x}})^{- 1}[\widehat{{\mathfrak {x}}}] = \widehat{{\mathfrak {x}}} + \big (0, 0, \, {\Upsilon }^\bot (\tau , {\mathfrak {x}})[\widehat{{\mathfrak {x}}} ] + {{\mathcal {R}}}^\bot _{N}(\tau , {\mathfrak {x}})[\widehat{{\mathfrak {x}}} ] \big ) \, , \nonumber \\&{\Upsilon }^\bot (\tau , {\mathfrak {x}}) [\widehat{{\mathfrak {x}}} ] := \Pi _\bot \sum _{k = 0 }^{N +m} T_{b_{m - k}(\tau , {\mathfrak {x}})}\partial _x^{m - k} [{\widehat{w}}] + \Pi _\bot \sum _{k = 0}^{N +m} T_{B_{m - k}(\tau , {\mathfrak {x}})[\widehat{{\mathfrak {x}}}]} \partial _x^{m - k} w \end{aligned}$$

with the following properties: there exist $s_N$, $\sigma _N \ge N$ so that for any $s \ge s_N$, there exist $\delta \equiv \delta (s, N) > 0$ and $0< \varepsilon _0 \equiv \varepsilon _0(s, N) < 1$ so that the following holds: for any $0 \le k \le N + m$ and $-1 \le \tau \le 1$,

$$\begin{aligned} \begin{aligned}&b_{m - k}(\tau , \cdot ) \in C^\infty _b( {{\mathcal {V}}}^{s + \sigma _N}(\delta ) \times [0, \varepsilon _0] , \, H^s({\mathbb {T}}_1)),\\&B_{m - k}(\tau , \cdot ) \in C^\infty _b\big ( {{\mathcal {V}}}^{s + \sigma _N}(\delta ) \times [0, \varepsilon _0], \, {{\mathcal {B}}}(E_{s + \sigma _N}, H^s({\mathbb {T}}_1))\big )\,, \\&{{\mathcal {R}}}^\bot _N(\tau , \cdot ) \in C^\infty _b \big ( {{\mathcal {V}}}^s(\delta ) \times [0, \varepsilon _0], \, {{\mathcal {B}}}(H^s({\mathbb {T}}_1), H^{s + N +1}_\bot ({\mathbb {T}}_1)) \big ) \end{aligned} \end{aligned}$$

with $b_{m- k}(\tau , \cdot )$, $B_{m- k}(\tau , \cdot )$, and ${{\mathcal {R}}}^\bot _N(\tau , \cdot )$ being small of order $p - 1$, and the expansion above holds for any ${\mathfrak {x}} \in {\mathcal {V}}^{s+\sigma _N}(\delta )$ and $\widehat{{\mathfrak {x}}} \in E_{s+\sigma _N}$.

#### Proof

First we note that for any $-1 \le \tau \le 1$, $d \Phi _Y(\tau , {\mathfrak {x}})^{- 1} = d \Phi _Y(- \tau , \Phi _Y(\tau , {\mathfrak {x}}))$ and that by Lemma 3.5,

$$\begin{aligned} \Phi _Y(\tau , {\mathfrak {x}}) = {\mathfrak {x}} + \Big ( 0, 0, \, \Pi _\bot \sum _{k = 0}^{N +m} T_{b_{m - k}(\tau , {\mathfrak {x}}; \Phi _Y)} \partial _x^{m - k} w + {{\mathcal {R}}}^\bot _N(\tau , {\mathfrak {x}}; \Phi _Y) \Big ) \end{aligned}$$

with $b_{m-k}(\tau , \cdot ; \Phi _Y) \in C^\infty _b\big ( {{\mathcal {V}}}^{s + \sigma _N}(\delta ) \times [0, \varepsilon _0], \, H^s({\mathbb {T}}_1) \big )$ being small of order $p - 1$ and ${{\mathcal {R}}}^\bot _N(\tau , \cdot ; \Phi _Y) \in {{\mathcal {OS}}}^p(N)$. To simplify notation, let ${\widetilde{b}}_{m-k}(\tau , {\mathfrak {x}}):= b_{m-k}(\tau , {\mathfrak {x}}; \Phi _Y)$ and $\widetilde{{\mathcal {R}}}^\bot _N(\tau , {\mathfrak {x}}) := {{\mathcal {R}}}^\bot _N(\tau , {\mathfrak {x}}; \Phi _Y)$. Then the normal component of $d \Phi _Y( \tau , {\mathfrak {x}})^{- 1}[\widehat{{\mathfrak {x}}}] - \widehat{{\mathfrak {x}}}$ can be computed as follows

$$\begin{aligned}&\Pi _\bot \sum _{k = 0}^{N +m} T_{{\widetilde{b}}_{m-k}(- \tau , \Phi _Y(\tau , {\mathfrak {x}}))} \partial _x^{m-k} {\widehat{w}} + \Pi _\bot \sum _{k = 0}^{N +m} T_{d {\widetilde{b}}_{m-k}(- \tau , \Phi _Y(\tau , {\mathfrak {x}}))[\widehat{{\mathfrak {x}}}]} \partial _x^{m-k} \Phi _Y^\bot (\tau , {\mathfrak {x}})\\&\quad + d \widetilde{{{\mathcal {R}}}}^\bot _N(- \tau , \Phi _Y(\tau , {\mathfrak {x}})) [\widehat{{\mathfrak {x}}}] \, . \end{aligned}$$

By expanding the terms $T_{d {\widetilde{b}}_{m-k}(- \tau , \Phi _Y(\tau , {\mathfrak {x}}))[\widehat{{\mathfrak {x}}}]} \partial _x^{m-k} \Phi _Y^\bot (\tau , {\mathfrak {x}})$ with the help of Lemma 2.5, one is led to define $b_{m-k}(\tau , {\mathfrak {x}})$, $B_{m-k}(\tau , {\mathfrak {x}})$, and ${{\mathcal {R}}}^\bot _N(\tau , {\mathfrak {x}})$ with the claimed properties. $\square$

Combining Lemmas 3.5 and 3.6, one obtains an expansion of the pullback of various types of vector fields by the time one flow map $\Phi _Y(1, \cdot )$:

#### Lemma 3.7

Let *N*, *p*, $q \in {\mathbb {N}}$ and let $\Phi _Y(1, {\mathfrak {x}})$ denote the time one flow map, corresponding to the vector field $Y = (0, 0, \, Y^\bot )$, with $Y^\bot ({\mathfrak {x}}) = \Pi _\bot T_{a_{m}({\mathfrak {x}})} \partial _x^{ m} w$ and $m \le 0$, satisfying (3.4) (cf. Lemma 3.5). Then the following holds:

(i) For any $X := (0,0, X^\bot )$ with $X^\bot \in {{\mathcal {OB}}}^q(n, N)$ and $n \ge 0$, the pullback $\Phi _Y^* X({\mathfrak {x}}) = d\Phi _Y(1, {\mathfrak {x}})^{-1} X(\Phi _Y(1, {\mathfrak {x}}))$ of *X* by $\Phi _Y(1, \cdot )$ admits an expansion of the form
  $$\begin{aligned} \Phi _Y^* X ({\mathfrak {x}})= \big (0, 0, \, X^\bot ({\mathfrak {x}}) + \Upsilon ^\bot ({\mathfrak {x}}) + {{\mathcal {R}}}_N^\bot ({\mathfrak {x}}) \big ) \end{aligned}$$
  where $$\Upsilon ^\bot \in {{\mathcal {OB}}}^{p + q - 1}(n, N)$$ and $${{\mathcal {R}}}_N^\bot \in {{\mathcal {OS}}}^{p + q - 1}(N)$$.
(ii) For any *X* in ${{\mathcal {OS}}}^q(N)$, the pullback $\Phi _Y^* X$ of *X* by $\Phi _Y(1, \cdot )$ admits an expansion of the form
  $$\begin{aligned} \Phi _Y^* X({\mathfrak {x}}) = X({\mathfrak {x}}) + \big (0, 0, \, \Upsilon ^\bot ({\mathfrak {x}}) \big ) + {{\mathcal {R}}}_N({\mathfrak {x}}) \end{aligned}$$
  where $$ \Upsilon ^\bot \in {{\mathcal {OB}}}^{p + q - 1}(m, N)$$ and $${{\mathcal {R}}}_N \in {{\mathcal {OS}}}^{p + q - 1}(N)$$.

#### Proof

We only prove item (*i*) since item (*ii*) can be proved by similar arguments. Since by (1.36) with $\tau =1$

$$\begin{aligned} \Phi _Y^* X( {\mathfrak {x}}) = X({\mathfrak {x}}) + \int _0^1 (d\Phi _Y(t, {\mathfrak {x}}))^{- 1}[X, Y](\Phi _Y(t, {\mathfrak {x}}))\, d t, \end{aligned}$$

we analyze for any $t \in [0, 1]$ the vector field

3.19 $$\begin{aligned} Z(t, {\mathfrak {x}}) := (d\Phi _Y(t, {\mathfrak {x}}) )^{- 1}[X, Y](\Phi _Y(t, {\mathfrak {x}})). \end{aligned}$$

Recall that $Y^\bot ({\mathfrak {x}}) = \Pi _\bot T_{a_{m}({\mathfrak {x}})} \partial _x^{m} w \in {{\mathcal {OB}}}^p(m, N)$. Taking into account that $m_* = \max \{n+m-1, m, n\} = n$ (since $n \ge 0 \ge m$), it follows from Lemma 3.3 that $[X, Y] = \big (0, 0, [X^\bot , Y^\bot ]\big )$ satisfies

$$\begin{aligned} {[}X^\bot , Y^\bot ] = {\mathcal {C}}^\bot _{[X^\bot , Y^\bot ]}+ {{\mathcal {R}}}^\bot _{[X^\bot , Y^\bot ]} , \quad {\mathcal {C}}^\bot _{[X^\bot , Y^\bot ]} \in {{\mathcal {OB}}}^{p + q - 1}(n, N), \quad {{\mathcal {R}}}^\bot _{[X^\bot , Y^\bot ]} \in {{\mathcal {OS}}}^{p + q - 1}(N). \end{aligned}$$

By Definitions 3.1–3.3, and Lemma 3.5, Lemma 3.6, as well as Lemma 2.5, one obtains

3.20 $$\begin{aligned} \int _0^1 Z(t, {\mathfrak {x}})\, d t = \big (0, 0, \, \Upsilon ^\bot ({\mathfrak {x}}) + {{\mathcal {R}}}_N^\bot ({\mathfrak {x}}) \big ) \end{aligned}$$

with $$\Upsilon ^\bot ({\mathfrak {x}}) \in {{\mathcal {OB}}}^{p + q - 1}(n, N)$$ and $${{\mathcal {R}}}_N^\bot ({\mathfrak {x}}) \in {{\mathcal {OS}}}^{p + q - 1}(N)$$. $$\square $$

Next we analyze the pullback $\Phi _Y^* X_{{\mathcal {N}}}$ of the Hamiltonian vector field $X_{{{\mathcal {N}}}}({\mathfrak {x}})$ with ${\mathcal {N}}$ being the following Hamiltonian in normal form (cf. (4.15)),

3.21 $$\begin{aligned} {{\mathcal {N}}}({\mathfrak {x}}) := (\omega + \varepsilon {\widehat{\omega }}) \cdot y + Q(y) + \frac{1}{2} \big \langle D^{- 1}_\bot \Omega _\bot w, w \big \rangle \,, \qquad \omega \in \Pi \, , \ \ {\widehat{\omega }} \in {\mathbb {R}}^{S_+}\, , \end{aligned}$$

where the Fourier multipliers $D^{- 1}_\bot$ and $\Omega _\bot \equiv \Omega _\bot (\omega )$ are given by (1.42) and *Q* is assumed to be a map in $C^\infty _b(B_{S_+}(\delta ) \times [0, \varepsilon _0], \, {\mathbb {R}})$ with $Q(0) = 0$ and $\nabla _y Q(0) = 0$. Since $\partial _x D_\bot ^{- 1} \Omega _\bot = \mathrm{i} \Omega _\bot$, the vector field $X_{{{\mathcal {N}}}}({\mathfrak {x}})$ then reads

3.22 $$\begin{aligned} X_{{\mathcal {N}}}({\mathfrak {x}}) = \begin{pmatrix} - \nabla _y {{\mathcal {N}}}({\mathfrak {x}}) \\ \nabla _\theta {{\mathcal {N}}}({\mathfrak {x}}) \\ \partial _x \nabla _\bot {{\mathcal {N}}}({\mathfrak {x}}) \end{pmatrix} = \begin{pmatrix} - \omega - \varepsilon {\widehat{\omega }} - \nabla _y Q(y) \\ 0 \\ \mathrm{i} \Omega _\bot w \end{pmatrix} \end{aligned}$$

and its differential is given by

3.23 $$\begin{aligned} d X_{{{\mathcal {N}}}}({\mathfrak {x}}) = \begin{pmatrix} 0 &{} - d_y \nabla _y Q(y) &{} 0 \\ 0 &{} 0 &{} 0 \\ 0 &{} 0 &{} \mathrm{i} \Omega _\bot \end{pmatrix} \, . \end{aligned}$$

Note that ${{\mathcal {N}}}({\mathfrak {x}})$ does not depend on $\theta$, but only on *y*, *w*, and $\varepsilon$. For notational convenience, we will often write ${{\mathcal {N}}}(y, w)$ instead of ${{\mathcal {N}}}({\mathfrak {x}})$. The following result on the expansion of $\mathrm{i} \Omega _\bot$ can be found in [27].

#### Lemma 3.8

([27, Lemma C.7]). For any $N \in {\mathbb {N}}$, the Fourier multiplier $\mathrm{i} \Omega _\bot$ has an expansion of the form

$$\begin{aligned} \mathrm{i} \Omega _\bot = - \partial _x^3 + \sum _{k = 1}^N c_{- k} \partial _x^{- k} + {{\mathcal {R}}}^\bot _N\, , \end{aligned}$$

where $c_{- k}\equiv c_{-k}(\omega )$ are real constants, depending only on the parameter $\omega \in \Pi$, and ${{\mathcal {R}}}^\bot _N \equiv {\mathcal {R}}_N^\bot (\omega )$ is in ${{\mathcal {B}}}(H^s_\bot ({\mathbb {T}}_1), H^{s + N + 1}_\bot ({\mathbb {T}}_1))$ for any $s \in {\mathbb {R}}$.

#### Lemma 3.9

Let $X_{{\mathcal {N}}}$ be the vector field given by (3.22) and $Y = (0, 0, \, Y^\bot )$ be the vector field with $Y^\bot ({\mathfrak {x}}) = \Pi _\bot T_{a_{m}({\mathfrak {x}})} \partial _x^{ m} w$ and $m \le 0$, satisfying (3.4) with $p, N \in {\mathbb {N}}$. Furthermore let $\Phi _Y(1, {\mathfrak {x}})$ be the time one flow map corresponding to the vector field *Y* (cf. Lemma 3.5). Then the following holds:

(i) If in addition $Y^\bot ({\mathfrak {x}}) = \Pi _\bot T_{a_{m}({\mathfrak {x}})} \partial _x^{ m} w$ is in ${{\mathcal {OB}}}^2_{w}(m, N)$, hence $a_m({\mathfrak {x}}) \equiv a_m(\theta , y)$ independent of *w*, and if $\langle a_{m}({\mathfrak {x}}) \rangle _x = 0$, then $[X_{{\mathcal {N}}}, Y]$ is of the form $\big ( 0, 0 , \, [X_{{\mathcal {N}}}, Y]^\bot \big )$ with $[X_{{\mathcal {N}}}, Y]^\bot \in {{\mathcal {OB}}}^2(2 + m, N)$ and admits an expansion of the form
  $$\begin{aligned} {[}X_{{\mathcal {N}}}, Y]^\bot ({\mathfrak {x}}) = \Pi _\bot T_{-3\partial _x a_{m}({\mathfrak {x}})} \partial _x^{2 + m} w + {\mathcal {C}}^\bot ( {\mathfrak {x}}) + {{\mathcal {R}}}^\bot _{N}( {\mathfrak {x}}) + {{\mathcal {OB}}}^3(m, N), \end{aligned}$$
  where ${\mathcal {C}}^\bot ( {\mathfrak {x}}) \in {{\mathcal {OB}}}^2_{w}(1 + m, N)$ and ${{\mathcal {R}}}^\bot _{N}( {\mathfrak {x}}) \in {{\mathcal {OS}}}_{w}^2(N)$. Moreover ${\mathcal {C}}^\bot ( {\mathfrak {x}})$ and ${{\mathcal {R}}}^\bot _{N}( {\mathfrak {x}})$ are of the form ${\mathcal {C}}^\bot ( {\mathfrak {x}}) = {\mathcal {C}}^\bot ( \theta , y) [w]$ and, respectively, ${{\mathcal {R}}}^\bot _{N}( {\mathfrak {x}}) = {{\mathcal {R}}}^\bot _{N}(\theta , y)[w]$, and the diagonal matrix elements of ${\mathcal {C}}^\bot ( \theta , y)$ and ${{\mathcal {R}}}^\bot _{N}(\theta , y)$ vanish,
  $$\begin{aligned} {[}{\mathcal {C}}^\bot ( \theta , y)]_j^j = 0, \qquad [{\mathcal {R}}^\bot _N( \theta , y)]_j^j = 0, \ \ \qquad \forall j \in S^\bot \,. \end{aligned}$$
(ii) If in addition $Y^\bot ({\mathfrak {x}})$ is in ${{\mathcal {OB}}}^2_{ww}(m, N)$, hence $a_m({\mathfrak {x}})$ of the form $A_{m}(\theta )[w]$, then $[X_{{\mathcal {N}}}, Y]({\mathfrak {x}})$ is of the form $(0, 0, [X_{{\mathcal {N}}}^\bot , Y^\bot ]({\mathfrak {x}}))$ with $[X_{{\mathcal {N}}}, Y]^\bot \in {{\mathcal {OB}}}^2(2 + m, N)$ and admits an expansion of the form
  $$\begin{aligned} {[}X_{{\mathcal {N}}}, Y]^\bot ({\mathfrak {x}})= \Pi _\bot T_{- 3\partial _x A_{m}(\theta )[w]} \partial _x^{2 + m} w +{{\mathcal {C}}}^\bot ({\mathfrak {x}}) + {{\mathcal {R}}}^\bot _{N}( {\mathfrak {x}}) + {{\mathcal {OB}}}^3(m, N) \, , \end{aligned}$$
  where $${\mathcal {C}}^\bot ( {\mathfrak {x}}) \in {{\mathcal {OB}}}^2_{ww}(1 + m, N)$$ and $${{\mathcal {R}}}^\bot _{N}( {\mathfrak {x}}) \in {{\mathcal {OS}}}_{ww}^2(N)$$.

#### Proof

(*i*): Since $Y^\bot ({\mathfrak {x}}) = \Pi _\bot T_{a_{m}({\mathfrak {x}})} \partial _x^{ m} w$ is in ${{\mathcal {OB}}}^2_{w}(m, N)$, $a_m$ is independent of *w* and for any $s \ge s_N$,
  3.24 $$\begin{aligned} a_{m} \in C^\infty _b \big ( {{\mathcal {V}}}^{s + \sigma _N}(\delta ) \times [0, \varepsilon _0], \, H^s({\mathbb {T}}_1) \big ) \ \ \text {small of order one.} \end{aligned}$$
  For notational convenience, we write $Y^\bot (\theta , y)[w]$ instead of $Y^\bot ({\mathfrak {x}})$ (similarly as we write $a_m(\theta , y)$ instead of $a_m({\mathfrak {x}})$). Then
  $$\begin{aligned}{}[X_{{\mathcal {N}}}, Y]({\mathfrak {x}}) = d X_{{\mathcal {N}}}(y, w)[Y({\mathfrak {x}})] - d Y({\mathfrak {x}})[X_{{\mathcal {N}}}(y, w)] \end{aligned}$$
  can be computed as
  3.25 $$\begin{aligned}&{[}X_{{\mathcal {N}}}, Y]({\mathfrak {x}}) {\mathop {=}\limits ^{(3.22),(3.23)}} \begin{pmatrix} 0 &{} - d_y (\nabla _y Q(y)) &{} 0 \\ 0 &{} 0 &{} 0 \\ 0 &{} 0 &{} \mathrm{i} \Omega _\bot \end{pmatrix} \begin{pmatrix} 0 \\ 0 \\ Y^\bot ({\mathfrak {x}}) \end{pmatrix} \nonumber \\&\quad - \begin{pmatrix} 0 &{} 0 &{} 0 \\ 0 &{} 0 &{} 0 \\ \partial _\theta Y^\bot ({\mathfrak {x}})&{} \partial _y Y^\bot ({\mathfrak {x}}) &{} Y^\bot (\theta , y) \end{pmatrix} \begin{pmatrix} - \omega - \varepsilon {\widehat{\omega }} - \nabla _y Q(y) \\ 0 \\ \mathrm{i} \Omega _\bot w \end{pmatrix} = \begin{pmatrix} 0 \\ 0 \\ [X_{{\mathcal {N}}}, Y]^\bot ({\mathfrak {x}}) \end{pmatrix}\nonumber \\ \end{aligned}$$
  where
  $$\begin{aligned} {[}X_{{\mathcal {N}}}, Y]^\bot ({\mathfrak {x}}) :=\Big ( [\mathrm{i} \Omega _\bot , \, Y^\bot (\theta , y)]_{lin} + (\omega + \varepsilon {\widehat{\omega }}) \cdot \partial _\theta \, Y^\bot (\theta , y) + \nabla _y Q(y) \cdot \partial _\theta \, Y^\bot (\theta , y)\Big )[w] \end{aligned}$$
  By (3.21), $\nabla _y Q(y)$ is small of order one and hence
  $$\begin{aligned} \begin{aligned}&\omega \cdot \partial _\theta \, Y^\bot (\theta , y)[w] = \Pi _\bot T_{\omega \cdot \partial _\theta a_{m}(\theta , y)} \partial _x^{m} w \in {{\mathcal {OB}}}^2_{w}(m, N)\,, \\&\varepsilon {\widehat{\omega }} \cdot \partial _\theta \, Y^\bot (\theta , y)[w] = \varepsilon \Pi _\bot T_{{\widehat{\omega }} \cdot \partial _\theta a_{m}(\theta , y)} \partial _x^{ m} w \in {{\mathcal {OB}}}^3(m, N)\,, \\&\nabla _y Q(y) \cdot \partial _\theta \, Y^\bot (\theta , y)[w] = \Pi _\bot T_{\nabla _y Q(y) \cdot \partial _\theta \, a_{m}(\theta , y)} \partial _x^{m} w \in {{\mathcal {OB}}}^3( m, N)\, . \end{aligned} \end{aligned}$$
  Furthermore by (3.24), Corollary 2.1, and Lemma 3.8, one sees that
  3.26 $$\begin{aligned}&\big [ \mathrm{i} \Omega _\bot , Y^\bot (\theta , y) \big ]_{lin} w = \Pi _\bot T_{- 3\partial _x a_{m}(\theta , y)} \partial _x^{2 + m} w + {{\mathcal {C}}}^{(1)}(\theta , y)[w] + {{\mathcal {R}}}^\bot _N(\theta , y)[w]\,,\nonumber \\&{{\mathcal {C}}}^{(1)}(\theta , y)[w] \in {{\mathcal {OB}}}^2_{w}(1 + m, N), \quad {{\mathcal {R}}}^\bot _N(\theta , y)[w] \in {{\mathcal {OS}}}_{w}^2(N)\,. \end{aligned}$$
  Altogether we have shown that
  $$\begin{aligned} \begin{aligned}&[X_{{\mathcal {N}}}, Y]^\bot ({\mathfrak {x}}) = \Pi _\bot T_{-3\partial _x a_{m}(\theta , y)} \partial _x^{2 + m} w + {{\mathcal {C}}}^\bot (\theta , y)[w] + {{\mathcal {R}}}^\bot _N(\theta , y)[w] + {{\mathcal {OB}}}^3( m, N) \,, \\&{\mathcal {C}}^\bot ( \theta , y) [w] := {{\mathcal {C}}}^{(1)}(\theta , y)[w] + \omega \cdot \partial _\theta Y^\bot (\theta , y)[w] \in {{\mathcal {OB}}}^2_{w}(1 + m, N)\, . \end{aligned} \end{aligned}$$
  For any $j \in S^\bot$, the diagonal matrix element $[\omega \cdot \partial _\theta \, Y^\bot (\theta , y)]_j^j$ vanishes,
  $$\begin{aligned} {[}\omega \cdot \partial _\theta \, Y^\bot (\theta , y)]_j^j = \omega \cdot \partial _\theta \langle a_{m}(\theta , y) \big \rangle _x (\mathrm{i} 2 \pi j)^{m} = 0, \end{aligned}$$
  since by assumption $\langle a_{m}(\theta , y) \rangle _x = 0$, and so does the diagonal matrix element $\big [ [\mathrm{i} \Omega _\bot \,,\, Y^\bot (\theta , y)]_{lin} \big ]_j^j$, implying together with (3.26)
  $$\begin{aligned} {[} {\mathcal {C}}^\bot ( \theta , y)]_j^j = 0, \qquad [{{\mathcal {R}}}^\bot _{N}( \theta , y)]_j^j = 0\, , \qquad \ \ \forall j \in S^\bot \, . \end{aligned}$$
(*ii*): Since $Y^\bot ({\mathfrak {x}}) = \Pi _\bot T_{a_{m}({\mathfrak {x}})} \partial _x^{ m} w$ is in ${{\mathcal {OB}}}^2_{ww}(m, N)$, it follows from Definition 3.4 that $a_m({\mathfrak {x}})$ is of the form $a_m({\mathfrak {x}}) = A_{m}(\theta )[w]$ and that for any $s \ge s_N$,
  3.27 $$\begin{aligned} A_{m} \in C^\infty _b\big ({\mathbb {T}}^{S_+}, \, {{\mathcal {B}}}(H^{s + \sigma _N}_\bot ({\mathbb {T}}_1), H^s({\mathbb {T}}_1) \big ) \, . \end{aligned}$$
  For notational convenience, we write $a_m(\theta , w)$ instead of $a_m({\mathfrak {x}})$. Arguing as in (3.25), one sees that ${[}X_{{\mathcal {N}}}, Y]({\mathfrak {x}}) = d X_{{\mathcal {N}}}(y, w)[Y({\mathfrak {x}})] - d Y({\mathfrak {x}})[X_{{\mathcal {N}}}(y, w)]$ can be computed as
  3.28 $$\begin{aligned}&{[}X_{{\mathcal {N}}}, Y]({\mathfrak {x}}) = \begin{pmatrix} 0 &{} - d_y( \nabla _y Q(y)) &{} 0 \\ 0 &{} 0 &{} 0 \\ 0 &{} 0 &{} \mathrm{i} \Omega _\bot \end{pmatrix} \begin{pmatrix} 0 \\ 0 \\ Y^\bot ({\mathfrak {x}}) \end{pmatrix} \nonumber \\&\quad - \begin{pmatrix} 0 &{} 0 &{} 0 \\ 0 &{} 0 &{} 0 \\ d_\theta Y^\bot ({\mathfrak {x}})&{} 0&{} d_\bot Y^\bot ({\mathfrak {x}}) \end{pmatrix} \begin{pmatrix} - \omega - \varepsilon {\widehat{\omega }} - \nabla _y Q(y) \\ 0 \\ \mathrm{i} \Omega _\bot w \end{pmatrix} = \begin{pmatrix} 0 \\ 0 \\ {[}X_{{\mathcal {N}}}, Y]^\bot ({\mathfrak {x}}) \end{pmatrix}\nonumber \\ \end{aligned}$$
  where
  $$\begin{aligned} \begin{aligned}&{[}X_{{\mathcal {N}}}, Y]^\bot ({\mathfrak {x}}) = \mathrm{i} \Omega _\bot [Y^\bot ({\mathfrak {x}})] - d_\bot Y^\bot ({\mathfrak {x}})[\mathrm{i} \Omega _\bot w] + ( \omega + \varepsilon {\widehat{\omega }}) \cdot \partial _\theta \, Y^\bot ({\mathfrak {x}}) + \nabla _y Q(y) \cdot \partial _\theta \, Y^\bot ({\mathfrak {x}}) \, . \end{aligned} \end{aligned}$$
  Since by (3.21), $\nabla _y Q(y)$ is small of order one, one infers that
  3.29 $$\begin{aligned} \begin{aligned}&\omega \cdot \partial _\theta \, Y^\bot ({\mathfrak {x}}) = \Pi _\bot T_{\omega \cdot \partial _\theta \, A_{m}(\theta )[w]} \partial _x^{m} w \in {{\mathcal {OB}}}^2_{ww}(m, N)\,, \\&\varepsilon {\widehat{\omega }} \cdot \partial _\theta \, Y^\bot ({\mathfrak {x}}) = \varepsilon \Pi _\bot T_{{\widehat{\omega }} \cdot \partial _\theta \, A_{m}(\theta )[w]} \partial _x^{m} w \in {{\mathcal {OB}}}^3(m, N)\,, \\&\nabla _y Q(y) \cdot \partial _\theta \, Y^\bot ({\mathfrak {x}}) = \Pi _\bot T_{\nabla _y Q(y) \cdot \partial _\theta \, A_{m}(\theta )[w]} \partial _x^{m} w \in {{\mathcal {OB}}}^3(m, N)\,. \end{aligned} \end{aligned}$$
  Furthermore, $\mathrm{i} \Omega _\bot [Y^\bot ({\mathfrak {x}})] - d_\bot Y({\mathfrak {x}})[\mathrm{i} \Omega _\bot w]$ can be computed as
  3.30 $$\begin{aligned} \begin{aligned}&\mathrm{i} \Omega _\bot \Pi _\bot T_{A_{m}(\theta )[w]} \partial _x^{m} w - \Pi _\bot T_{A_{m}(\theta )[w]} \partial _x^{m} \mathrm{i} \Omega _\bot w - \Pi _\bot T_{A_{m}(\theta )[\mathrm{i} \Omega _\bot w]} \partial _x^{m} w \\&= \Pi _\bot \big [\mathrm{i} \Omega _\bot \,,\, T_{A_{m}(\theta )[w]} \partial _x^{m} \big ]_{lin} w - \Pi _\bot T_{A_{m}(\theta )[\mathrm{i} \Omega _\bot w]} \partial _x^{ m} w\,. \end{aligned} \end{aligned}$$
  By (3.27), Corollary 2.1, and Lemma 3.8 one has
  3.31 $$\begin{aligned}&\Pi _\bot T_{A_{m}(\theta )[\mathrm{i} \Omega _\bot w]} \partial _x^{ m} w \in {{\mathcal {OB}}}_{ww}^2( m, N)\, ,\nonumber \\&\Pi _\bot \big [\mathrm{i} \Omega _\bot \,,\, T_{A_{m}(\theta )[w]} \partial _x^{m} \big ]_{lin} w = \Pi _\bot T_{- 3\partial _x A_{m}(\theta )[w]} \partial _x^{2 + m}w + {{\mathcal {C}}}^{(1)}({\mathfrak {x}}) + {{\mathcal {R}}}^\bot _N({\mathfrak {x}}) + {{\mathcal {OB}}}^3( m, N) \,, \nonumber \\&{\mathcal {C}}^{(1)}( {\mathfrak {x}}) \in {{\mathcal {OB}}}_{ww}^2( 1 + m, N)\, , \qquad {{\mathcal {R}}}^\bot _N({\mathfrak {x}}) \in {{\mathcal {OS}}}_{ww}^2(N)\, . \end{aligned}$$
  Altogether, the identities (3.29)–(3.31) yield
  $$\begin{aligned} \begin{aligned}&{[}X_{{\mathcal {N}}}, Y]^\bot ({\mathfrak {x}}) = \Pi _\bot T_{-3\partial _x A_{m}(\theta )[w]} \partial _x^{2 + m} w + {\mathcal {C}}^\bot ( {\mathfrak {x}}) + {{\mathcal {R}}}^\bot _N({\mathfrak {x}}) + {{\mathcal {OB}}}^3( m, N) \,, \\&{\mathcal {C}}^\bot ( {\mathfrak {x}}) := {{\mathcal {C}}}^{(1)}({\mathfrak {x}}) + \Pi _\bot T_{\omega \cdot \partial _\theta \, A_{m}(\theta )[w]} \partial _x^{m} w - \Pi _\bot T_{A_{m}(\theta )[\mathrm{i} \Omega _\bot w]} \partial _x^{ m} w \in {{\mathcal {OB}}}^2_{ww}(1 + m, N) \end{aligned} \end{aligned}$$
  and hence item (*ii*) is proved. $\square$

#### Lemma 3.10

Let $X_{{\mathcal {N}}}$ be the vector field given by (3.22) and let $Y({\mathfrak {x}}) = (0, 0, \, Y^\bot ({\mathfrak {x}}))$ where $Y^\bot ({\mathfrak {x}})= \big (0, 0, Y^\bot _{0}({\mathfrak {x}}) + Y^\bot _{1}({\mathfrak {x}}) \big )$ and

3.32 $$\begin{aligned}&Y^\bot _{0}({\mathfrak {x}}) \equiv Y^\bot _{0}(\theta , y)[w] = \Pi _\bot T_{a_{m}(\theta , y)} \partial _x^{m} w \in {{\mathcal {OB}}}^2_{w}(m, N),\nonumber \\ \ \&\quad Y^\bot _{1}({\mathfrak {x}})= \Pi _\bot T_{A_{ m}(\theta )[w]} \partial _x^{m} w \in {{\mathcal {OB}}}_{ww}^2(m, N), \end{aligned}$$

with $N \in {\mathbb {N}}$ and $m \le 0$. If in addition $\langle a_{m} (\theta , y) \rangle _x = 0$, then the pullback $X_{{\mathcal {N}}, \Phi } \equiv \Phi _Y^* X_{{\mathcal {N}}}$ of the vector field $X_{{\mathcal {N}}}$ by be the time one flow map $\Phi _Y(1, \cdot )$ corresponding to *Y* has an expansion of the form

$$\begin{aligned} X_{{\mathcal {N}}, \Phi }({\mathfrak {x}}) = \big ( - \omega - \varepsilon {\widehat{\omega }} - \nabla _y Q(y), \ 0, \ X_{{\mathcal {N}}, \Phi }^\bot ({\mathfrak {x}}) \big ) \end{aligned}$$

where

$$\begin{aligned} \begin{aligned} X_{{\mathcal {N}}, \Phi }^\bot ({\mathfrak {x}})&= \mathrm{i} \Omega _\bot w + \Pi _\bot T_{- 3\partial _x (a_{ m}(\theta , y) + A_{m}(\theta )[w] )} \partial _x^{2 + m} w + {{\mathcal {C}}}_0^\bot (\theta , y)[w] + {{\mathcal {C}}}_1^\bot ({\mathfrak {x}}) \\&\qquad + {{\mathcal {R}}}^\bot _{N, 0}(\theta , y )[w] + {{\mathcal {R}}}^\bot _{N, 1}({\mathfrak {x}}) + {{\mathcal {OB}}}^3(2 + m, N) + {{\mathcal {OS}}}^3(N) \end{aligned} \end{aligned}$$

and ${{\mathcal {C}}}_0^\bot (\theta , y)$, ${{\mathcal {R}}}^\bot _{N, 0}(\theta , y)$, and ${{\mathcal {C}}}_1^\bot ({\mathfrak {x}})$, ${{\mathcal {R}}}^\bot _{N, 1}({\mathfrak {x}})$ are given by Lemma 3.9. Hence these terms satisfy

$$\begin{aligned} \begin{aligned}&{{\mathcal {C}}}^\bot _0(\theta , y)[w] \in {{\mathcal {OB}}}^2_{w}(1 + m, N), \qquad {{\mathcal {R}}}^\bot _{N, 0}(\theta , y)[w] \in {{\mathcal {OS}}}_{w}^2(N)\,, \\&{{\mathcal {C}}}_1^\bot ({\mathfrak {x}}) \in {{\mathcal {OB}}}^2_{ww}(1 + m, N), \ \qquad \quad \ {{\mathcal {R}}}^\bot _{N, 1}({\mathfrak {x}}) \in {{\mathcal {OS}}}^2_{ww}(N)\, , \end{aligned} \end{aligned}$$

and the diagonal matrix elements of ${{\mathcal {C}}}_0^\bot (\theta , y)$ and ${{\mathcal {R}}}^\bot _{N, 0}(\theta , y)$ vanish,

$$\begin{aligned} {[}{{\mathcal {C}}}_0^\bot (\theta , y)]_j^j = 0, \qquad [{{\mathcal {R}}}^\bot _{N, 0}(\theta , y)]_j^j = 0, \quad \qquad \forall j \in S^\bot \,. \end{aligned}$$

#### Proof

By (1.36), $X_{{\mathcal {N}}, \Phi }$ can be expanded as

$$\begin{aligned}&X_{{\mathcal {N}}, \Phi } = \Phi _Y^* X_{{\mathcal {N}}} = X_{{\mathcal {N}}} + [X_{{\mathcal {N}}}, Y] + Z, \\&\qquad Z({\mathfrak {x}}) := \int _0^1 (1 - t) (d \Phi _Y(t, {\mathfrak {x}}))^{- 1}[[X_{{\mathcal {N}}}, Y], Y](\Phi _Y(t, {\mathfrak {x}}))\, d t . \end{aligned}$$

By Lemma 3.9, one has $[X_{{\mathcal {N}}}, Y] = \big ( 0, \, 0, \, [X_{{\mathcal {N}}}, Y]^\bot \big )$ with $[X_{{\mathcal {N}}}, Y]^\bot \in {{\mathcal {OB}}}^2(2 + m, N)$ given by

3.33 $$\begin{aligned}&\Pi _\bot T_{-3\partial _x ( a_{m}(\theta , y) + A_{m}(\theta )[w] )} \partial _x^{2 + m} w + {{\mathcal {C}}}_0^\bot (\theta , y)[w] \nonumber \\&\quad + {{\mathcal {R}}}^\bot _{N, 0}(\theta , y)[w] + {{\mathcal {C}}}_1^\bot ({\mathfrak {x}})+ {{\mathcal {R}}}^\bot _{N, 1}({\mathfrak {x}}) + {{\mathcal {OB}}}^3(m, N) \, , \end{aligned}$$

where ${{\mathcal {C}}}_0^\bot (\theta , y)$, ${{\mathcal {R}}}^\bot _{N, 0}(\theta , y)$, and ${{\mathcal {C}}}_1^\bot ({\mathfrak {x}})$, ${{\mathcal {R}}}^\bot _{N, 1}({\mathfrak {x}})$ are given as in Lemma 3.9. In particular, the diagonal matrix elements of ${{\mathcal {C}}}_0^\bot (\theta , y)$ and ${{\mathcal {R}}}^\bot _{N, 0}(\theta , y)$ vanish. Furthermore, by Lemmata 3.2, 3.3, one infers that

3.34 $$\begin{aligned} {[}{[}X_{{\mathcal {N}}}, Y], Y] ({\mathfrak {x}})= \big ( 0, \, 0, \, {{\mathcal {C}}}_2^\bot ({\mathfrak {x}}) + {{\mathcal {R}}}^\bot _{N, 2}({\mathfrak {x}}) \big ) , \quad {{\mathcal {C}}}_2^\bot \in {{\mathcal {OB}}}^{2}(2 + m, N), \quad {{\mathcal {R}}}^\bot _{N, 2} \in {{\mathcal {OS}}}^{3}(N ),\nonumber \\ \end{aligned}$$

and hence concludes by Lemma 3.7 that

3.35 $$\begin{aligned} Z({\mathfrak {x}}) = \big ( 0, \, 0, \, {{\mathcal {C}}}^\bot _3({\mathfrak {x}}) + {{\mathcal {R}}}^\bot _{N, 3}({\mathfrak {x}}) \big ), \qquad {{\mathcal {C}}}^\bot _3 \in {{\mathcal {OB}}}^{3}(2 + m, N), \quad {{\mathcal {R}}}^\bot _{N, 3} \in {{\mathcal {OS}}}^{3}(N)\,. \end{aligned}$$

The claimed statement then follows by (3.33)–(3.35). $\square$

### Flows of Fourier multiplier vector fields and smoothing vector fields

In this subsection we discuss additional properties of Fourier multiplier vector fields and smooth vector fields and their flows, needed in Sect. 6.2.

We begin by considering the flows corresponding to Fourier multiplier vector fields. Let ${{\mathcal {M}}}$ be a vector field of the form $(0, 0, {{\mathcal {M}}}^\bot )$ with ${{\mathcal {M}}}^\bot \in {{\mathcal {OF}}}^p(0, N)$ and $N, p \in {\mathbb {N}}$ (cf. Definition 3.2). Then ${\mathcal {M}}^\bot ({\mathfrak {x}})$ has an expansion of the form ${\mathcal {M}}^\bot ({\mathfrak {x}}) {=} \sum _{k = 0}^{N} \lambda _{ - k}({\mathfrak {x}}) \partial _x^{ - k} w$ with the property that there exist $\sigma _N \ge 0$, $0< \delta \equiv \delta (N) <1$, and $0< \varepsilon _0 \equiv \varepsilon _0(N) < 1$, so that for any $0 \le k \le N$,

$$\begin{aligned} \lambda _{- k} : {{\mathcal {V}}}^{\sigma _N}(\delta ) \times [0, \varepsilon _0] \rightarrow {\mathbb {R}}, \, ({\mathfrak {x}}, \varepsilon ) \mapsto \lambda _{m - k}({\mathfrak {x}}) \equiv \lambda _{- k}({\mathfrak {x}}, \varepsilon ) \end{aligned}$$

is $C^\infty$-smooth and bounded. We denote by $\Phi _{{\mathcal {M}}} (\tau , \cdot )$ the flow corresponding to the vector field ${{\mathcal {M}}}$. By the standard ODE theorem in Banach spaces, there exist $s_N \ge 0$ so that for any $s \ge s_N$, there exist $0< \delta \equiv \delta (s, N) < 1$, and $0 < \varepsilon _0 \equiv \varepsilon _ 0(s, N) \ll \delta$, so that

$$\begin{aligned} \Phi _{\mathcal {M}}(\tau , \cdot ) \in C^\infty _b \big ({{\mathcal {V}}}^s(\delta ) \times [0, \varepsilon _0], \, {{\mathcal {V}}}^s(2 \delta ) \big )\, , \qquad \forall \, -1 \le \tau \le 1 \, . \end{aligned}$$

The following lemma can be proved arguing as in the proof of Lemma 3.5 (actually, the proof is simpler).

#### Lemma 3.11

For any $\tau \in [- 1, 1]$, the flow map $\Phi _{{\mathcal {M}}}(\tau , \cdot )$ admits an expansion of the form

$$\begin{aligned} \Phi _{{\mathcal {M}}}(\tau , {\mathfrak {x}}) = {\mathfrak {x}} + (0, 0, \Upsilon ^\bot (\tau , {\mathfrak {x}}) + {{\mathcal {R}}}^\bot _N(\tau , {\mathfrak {x}})) \end{aligned}$$

where $$\Upsilon ^\bot (\tau , \cdot ) \in {{\mathcal {OF}}}^p(0, N)$$ and $${{\mathcal {R}}}^\bot _N \in {{\mathcal {OS}}}^{2 p - 1}(N)$$.

The following lemma can be proved arguing as in the proof of Lemma 3.6.

#### Lemma 3.12

Let $\Phi _{\mathcal {M}}(\tau , {\mathfrak {x}})$ denote the flow map considered in Lemma 3.11, corresponding to the vector field ${\mathcal {M}} = (0, 0, \, {\mathcal {M}}^\bot )$ with ${{\mathcal {M}}}^\bot \in {{\mathcal {OF}}}^p(0, N)$ and $N, p \in {\mathbb {N}}$. Then $d \Phi _{\mathcal {M}}( \tau , {\mathfrak {x}})^{- 1}[\widehat{{\mathfrak {x}}}]$ admits an expansion of the form

3.36 $$\begin{aligned}&d \Phi _{\mathcal {M}}( \tau , {\mathfrak {x}})^{- 1}[\widehat{{\mathfrak {x}}}] = \widehat{{\mathfrak {x}}} + \big (0, 0, \, {\Upsilon }^\bot (\tau , {\mathfrak {x}})[\widehat{{\mathfrak {x}}} ] + {{\mathcal {R}}}^\bot _{N}(\tau , {\mathfrak {x}})[\widehat{{\mathfrak {x}}} ] \big ) \, , \\&{\Upsilon }^\bot (\tau , {\mathfrak {x}}) [\widehat{{\mathfrak {x}}} ] := \sum _{k = 0}^{N } \lambda _{- k}(\tau , {\mathfrak {x}}) \partial _x^{- k} {\widehat{w}} + \sum _{k = 0}^{N } \eta _{- k}(\tau , {\mathfrak {x}})[\widehat{{\mathfrak {x}}}] \partial _x^{- k } w \, ,\nonumber \end{aligned}$$

with the following properties: there exist $s_N$, $\sigma _N \ge N$ so that for any $s \ge s_N$, there exist $0< \delta \equiv \delta (s, N) < 1$ and $0< \varepsilon _0 \equiv \varepsilon _0(s, N) < 1$ so that the following holds: for any $0 \le k \le N$ and $-1 \le \tau \le 1$,

$$\begin{aligned} \begin{aligned}&\lambda _{- k} \in C^\infty _b({{\mathcal {V}}}^{ \sigma _N}(\delta ) \times [0, \varepsilon _0], \, {\mathbb {R}}), \qquad \eta _{- k} \in C^\infty _b\big ( {{\mathcal {V}}}^{ \sigma _N}(\delta ) \times [0, \varepsilon _0], \, {{\mathcal {B}}}(E_{ \sigma _N}, {\mathbb {R}})\big )\,, \\&{{\mathcal {R}}}^\bot _N \in C^\infty _b \big ( {{\mathcal {V}}}^s(\delta ) \times [0, \varepsilon _0], \, {{\mathcal {B}}}(H^s_\bot ({\mathbb {T}}_1), H^{s + N +1}_\bot ({\mathbb {T}}_1)) \big ), \end{aligned} \end{aligned}$$

and $\lambda _{- k}(\tau , \cdot )$, $\eta _{- k}(\tau , \cdot )$, and ${{\mathcal {R}}}^\bot _N(\tau , \cdot )$ are small of order $p - 1$.

The following lemma can be proved arguing as in the proof of Lemma 3.7.

#### Lemma 3.13

Let $\Phi _{\mathcal {M}}(1, {\mathfrak {x}})$ denote the time one flow map considered in Lemma 3.11, corresponding to the vector field ${\mathcal {M}} = (0, 0, \, {\mathcal {M}}^\bot )$, with ${{\mathcal {M}}}^\bot \in {{\mathcal {OF}}}^p(0, N)$ and *N*, $p \in {\mathbb {N}}$. Then the following holds:

(i) For any $X := (0,0, X^\bot )$ with $X^\bot \in {{\mathcal {OB}}}^q(n, N)$ and $q \ge 1$, $n \ge 0$, the pullback $\Phi _{{\mathcal {M}}}^* X$ of *X* by $\Phi _{\mathcal {M}}(1, \cdot )$ admits an expansion of the form
  $$\begin{aligned}&\Phi _{{\mathcal {M}}}^* X({\mathfrak {x}}) = \big (0, 0, X^\bot ({\mathfrak {x}}) + \Upsilon ^\bot ({\mathfrak {x}}) + {\mathcal {R}}^\bot _N({\mathfrak {x}}) \big ) \, ,\\&\qquad \Upsilon ^\bot \in {{\mathcal {OB}}}^{p + q - 1}(n, N), \quad {\mathcal {R}}^\bot _N \in {{\mathcal {OS}}}^{p + q - 1}(N). \end{aligned}$$
(ii) For any ${\mathcal {M}}_1 = \big ( 0, 0, {{\mathcal {M}}}_1^\bot \big )$ with ${{\mathcal {M}}}_1^\bot \in {{\mathcal {OF}}}^q(n, N)$ and $q \ge 1$, $n \ge 0$, the pullback $\Phi _{{\mathcal {M}}}^* {\mathcal {M}}_1$ of ${\mathcal {M}}_1$ by $\Phi _{\mathcal {M}}(1, \cdot )$ admits an expansion of the form
  $$\begin{aligned}&\Phi _{{{\mathcal {M}}}}^* {\mathcal {M}}_1({\mathfrak {x}}) = \big (0, 0, {\mathcal {M}}^\bot _1({\mathfrak {x}}) + \Upsilon ^\bot ({\mathfrak {x}}) + {{\mathcal {R}}}^\bot _N({\mathfrak {x}}) \big ), \\&\qquad \Upsilon ^\bot \in {{\mathcal {OF}}}^{p + q - 1}(n, N), \quad {{\mathcal {R}}}^\bot _N \in {{\mathcal {OS}}}^{p + q - 1}(N). \end{aligned}$$
(iii) For any $X \in {{\mathcal {OS}}}^q(N)$, the pullback $\Phi _{{\mathcal {M}}}^* X$ of *X* by $\Phi _{\mathcal {M}}(1, \cdot )$ admits an expansion of the form
  $$\begin{aligned} \Phi _{{\mathcal {M}}}^* X ({\mathfrak {x}})= X({\mathfrak {x}}) + \big (0,0, \, \Upsilon ^\bot ({\mathfrak {x}}) \big ) + {\mathcal {R}}_N({\mathfrak {x}}) \end{aligned}$$
  where $$ \Upsilon ^\bot \in {{\mathcal {OF}}}^{p + q - 1}(0, N)$$ and $${\mathcal {R}}_N \in {{\mathcal {OS}}}^{p + q - 1}(N)$$.

Next we consider ${{\mathcal {M}}} := (0, 0, {{\mathcal {M}}}^\bot )$ with ${{\mathcal {M}}}^\bot \in {{\mathcal {OF}}}^2_{ww}(0, N)$ and $N \in {\mathbb {N}}$ (cf. Definition 3.4-(*ii*2)), i.e., ${{\mathcal {M}}}^\bot ({\mathfrak {x}}) = {{\mathcal {M}}}^\bot (\theta , w)[w]$ with ${{\mathcal {M}}}^\bot (\theta , w) = \sum _{k=0}^N \Lambda _{- k}(\theta )[w] \partial _x^{-k}$ where, for some integer $\sigma _N \ge 0$ and some $0< \varepsilon _0 \equiv \varepsilon _0(N) < 1$,

3.37 $$\begin{aligned} \Lambda _{ -k} : {\mathbb {T}}^{S_+} \times [0, \varepsilon _0] \rightarrow {{\mathcal {B}}}(H^{\sigma _N}_\bot ({\mathbb {T}}_1), {\mathbb {R}}), \, \theta \mapsto \Lambda _{-k} (\theta ) \equiv \Lambda _{-k} (\theta , \varepsilon ), \quad \, 0 \le k \le N,\nonumber \\ \end{aligned}$$

are $C^\infty -$smooth. To obtain an expansion of the pullback $\Phi _{{\mathcal {M}}}^* X_{\mathcal {N}}$ of the vector field $X_{\mathcal {N}}$, defined in (3.22), by $\Phi _{\mathcal {M}}(1, \cdot )$, we first need to compute the one of the commutator $[X_{{\mathcal {N}}}, {\mathcal {M}}]$.

#### Lemma 3.14

The commutator $[X_{{\mathcal {N}}}, {\mathcal {M}}]({\mathfrak {x}})$ admits an expansion of the form

$$\begin{aligned} {[}X_{{\mathcal {N}}}, {{\mathcal {M}}}]({\mathfrak {x}}) = \big ( 0, 0, \ \omega \cdot \partial _\theta ( {{\mathcal {M}}}^\bot (\theta , w)[w]) - {{\mathcal {M}}}^\bot (\theta , \mathrm{i} \Omega _\bot w)[w] + {{\mathcal {OF}}}^3(0, N) \big ) \, . \end{aligned}$$

#### Proof

By (3.37) the differential of ${\mathcal {M}}$ can be computed as

$$\begin{aligned} d {{\mathcal {M}}}({\mathfrak {x}})[\widehat{{\mathfrak {x}}}] = \big ( 0, \, 0, \ {{\mathcal {M}}}^\bot (\theta , w)[{\widehat{w}}] + {{\mathcal {M}}}^\bot (\theta , {\widehat{w}})[w] + d_\theta \big ( {{\mathcal {M}}}(\theta , w)[w] \big )[{\widehat{\theta }}] \big )\,. \end{aligned}$$

By (3.22), (3.23), the commutator

$$\begin{aligned}{}[X_{{\mathcal {N}}}, {\mathcal {M}}]({\mathfrak {x}}) = d X_{{\mathcal {N}}}(y, w)[{\mathcal {M}}({\mathfrak {x}})] - d {\mathcal {M}}({\mathfrak {x}})[X_{{\mathcal {N}}}(y, w)] \end{aligned}$$

is given by

$$\begin{aligned} \begin{aligned}&{[}X_{{\mathcal {N}}}, {{\mathcal {M}}}]({\mathfrak {x}}) = \big ( 0, 0, \, \mathrm{i} \Omega _\bot {{\mathcal {M}}}^\bot (\theta , w)[w] \big )\\&\qquad - \big ( 0, 0, \, {{\mathcal {M}}}^\bot (\theta , w)[\mathrm{i} \Omega _\bot w] + {{\mathcal {M}}}^\bot (\theta , \mathrm{i} \Omega _\bot w)[w] - d_\theta \big ( {{\mathcal {M}}}^\bot (\theta , w)[w] \big )[\omega + \varepsilon {\widehat{\omega }} + \nabla _y Q(y)] \big ) \\&\quad = \big ( 0, 0, \, [\mathrm{i} \Omega _\bot , {{\mathcal {M}}}^\bot (\theta , w)]_{lin} w - {{\mathcal {M}}}^\bot (\theta , \mathrm{i} \Omega _\bot w)[w]\\&\qquad + (\omega + \varepsilon {\widehat{\omega }} + \nabla _y Q(y)) \cdot \partial _\theta \big ( {{\mathcal {M}}}^\bot (\theta , w)[w] \big ) \big ) \, . \end{aligned} \end{aligned}$$

Since ${{\mathcal {M}}}^\bot (\theta , w)$ and $\mathrm{i} \Omega _\bot$ are both Fourier multipliers, the linear commutator $[\mathrm{i} \Omega _\bot , {{\mathcal {M}}}^\bot (\theta , w)]_{lin}$ vanishes. The lemma then follows in view of the fact that

$$\begin{aligned}&(\varepsilon {\widehat{\omega }} + \nabla _y Q(y)) \cdot \partial _\theta \big ( {{\mathcal {M}}}^\bot (\theta , w)[w] \big ) \\&\quad = \sum _{k=0}^N (\varepsilon {\widehat{\omega }} + \nabla _y Q(y)) \cdot \partial _\theta )( \Lambda _{- k}(\theta )[w]) \partial _x^{-k} [w]\\&\qquad \qquad \in {{\mathcal {OF}}}^3(0, N). \end{aligned}$$

$$\square $$

#### Lemma 3.15

The pullback $\Phi _{{\mathcal {M}}}^* X_{\mathcal {N}}$ of the vector field $X_{\mathcal {N}}$ by $\Phi _{\mathcal {M}}(1, \cdot )$ with ${{\mathcal {M}}}$ given by (3.37) admits an expansion of the form

$$\begin{aligned} \Phi _{{\mathcal {M}}}^* X_{{\mathcal {N}}} ({\mathfrak {x}})= \begin{pmatrix} - \omega - \varepsilon {\widehat{\omega }} - \nabla _y Q(y) \\ 0 \\ \mathrm{i} \Omega _\bot w + \omega \cdot \partial _\theta ( {{\mathcal {M}}}^\bot (\theta , w)[w]) - {{\mathcal {M}}}^\bot (\theta , \mathrm{i} \Omega _\bot w)[w] + {{\mathcal {OF}}}^3(0, N) + {{\mathcal {OS}}}^3(N) \end{pmatrix} . \end{aligned}$$

#### Proof

We argue as in the proof of Lemma 3.10. By (1.36), $\Phi _{{\mathcal {M}}}^* X_{{\mathcal {N}}}$ can be expanded as

$$\begin{aligned} \begin{aligned}&\Phi _{{\mathcal {M}}}^* X_{{\mathcal {N}}} = X_{{\mathcal {N}}} + [X_{{\mathcal {N}}}, {{\mathcal {M}}}] + Z, \\&\qquad Z({\mathfrak {x}}) := \int _0^1 (1 - \tau ) [d \Phi _{{\mathcal {M}}}(\tau , {\mathfrak {x}})]^{- 1}[[X_{{\mathcal {N}}}, {{\mathcal {M}}}], {{\mathcal {M}}}](\Phi _{{\mathcal {M}}}(\tau , {\mathfrak {x}}))\, d \tau \,. \end{aligned} \end{aligned}$$

The claimed statement then follows by applying Lemmata 3.4, 3.11, 3.12, 3.14. $\quad \square$

Finally, we consider smoothing vector fields. Given a smoothing vector field ${{\mathcal {Q}}} \in {{\mathcal {OS}}}^p(N)$ with *N*, $p \in {\mathbb {N}}$ (cf. Definition 3.3), we denote by $\Phi _{{\mathcal {Q}}} (\tau , \cdot )$ the flow corresponding to the vector field ${{\mathcal {Q}}}$. By the standard ODE theorem in Banach spaces, there exists $s_N \ge 0$ so that for $s \ge s_N$, there exist $0< \delta \equiv \delta (s, N) < 1$ and $0 < \varepsilon _0 \equiv \varepsilon _0(s, N) \ll \delta$, so that

3.38 $$\begin{aligned}&\Phi _{\mathcal {Q}}(\tau , \cdot ) \in C^\infty _b \big ({{\mathcal {V}}}^s(\delta ) \times [0, \varepsilon _0], \, {{\mathcal {V}}}^s(2 \delta ) \big )\, , \nonumber \\&\quad \Phi _{\mathcal {Q}}(\tau , \cdot ) - \text {Id} \quad \text {small of order } p, \quad \forall \, -1 \le \tau \le 1 \, . \end{aligned}$$

#### Lemma 3.16

Let ${{\mathcal {Q}}} \in {{\mathcal {OS}}}^p(N)$ with *N*, $p \in {\mathbb {N}}$. For any $-1 \le \tau \le 1$, the following holds.

(i) The flow map $\Phi _{\mathcal {Q}}(\tau , \cdot )$ admits an expansion of the form
  $$\begin{aligned} \Phi _{\mathcal {Q}}(\tau , {\mathfrak {x}}) = {\mathfrak {x}} + {{\mathcal {R}}}_N(\tau , {\mathfrak {x}}), \qquad {{\mathcal {R}}}_N(\tau , \cdot ) \in {{\mathcal {OS}}}^p(N). \end{aligned}$$
(ii) The map $d \Phi _{\mathcal {S}}(\tau , {\mathfrak {x}})^{- 1}$ admits an expansion of the form
  $$\begin{aligned} d \Phi _{\mathcal {Q}}(\tau , {\mathfrak {x}})^{- 1}[\widehat{{\mathfrak {x}}}] = \widehat{{\mathfrak {x}}} + {{\mathcal {R}}}_N(\tau , {\mathfrak {x}})[\widehat{{\mathfrak {x}}}] \end{aligned}$$
  where there exists $s_N \ge 0$ so that for any $s \ge s_N$ there are $0< \delta \equiv \delta (s, N) < 1$ and $0< \varepsilon _0 \equiv \varepsilon _0(s, N) < 1$ such that
  $$\begin{aligned} {{\mathcal {R}}}_N(\tau , \cdot ) \in C^\infty _b \big ( {{\mathcal {V}}}^s(\delta )\times [0, \varepsilon _0], \, {{\mathcal {B}}}(E_s, E_{s + N +1}) \big )\,, \qquad \forall \, -1 \le \tau \le 1. \end{aligned}$$

#### Proof

To prove item (*i*) one uses the Volterra integral equation (cf. (3.6)) and (3.38) (cf. proof of Lemma 3.5). To prove item (*ii*), one argues as in the proof of Lemma 3.6, using the identity $d \Phi _{\mathcal {Q}}(\tau , {\mathfrak {x}})^{- 1} = d \Phi _{\mathcal {Q}}(- \tau , \Phi _{\mathcal {Q}}(\tau , {\mathfrak {x}}))$, $-1 \le \tau \le 1$ (cf. Remark 3.4).

$$\square $$

#### Lemma 3.17

For any ${{\mathcal {Q}}} \in {{\mathcal {OS}}}^p(N)$ with *N*, $p \in {\mathbb {N}}$, the following holds:

(i) For any $X := (0, 0, X^\bot )$ with $X^\bot \in {{\mathcal {OB}}}^q(m, N)$ and $m \in {\mathbb {Z}}$, $q \in {\mathbb {N}}$, the pullback $\Phi _{{\mathcal {Q}}}^* X$ of *X* by $\Phi _{{\mathcal {Q}}}(1, \cdot )$ admits an expansion of the form
  $$\begin{aligned} \Phi _{{\mathcal {Q}}}^* X({\mathfrak {x}}) = \big (0,0, \, X^\bot ({\mathfrak {x}}) + \Upsilon ^\bot ({\mathfrak {x}}) + {{\mathcal {R}}}^\bot _N \big ), \quad \Upsilon ^\bot \in {{\mathcal {OB}}}^{p + q - 1}(m, N), \quad {{\mathcal {R}}}^\bot _N \in {{\mathcal {OS}}}^{p + q - 1}(N). \end{aligned}$$
(ii) For any ${\mathcal {M}} := (0, 0, {{\mathcal {M}}}^\bot )$ with ${{\mathcal {M}}}^\bot \in {{\mathcal {OF}}}^q(m, N)$ and $m \in {\mathbb {Z}}$, $q \in {\mathbb {N}}$, the pullback $\Phi _{{\mathcal {Q}}}^* {\mathcal {M}}$ of ${\mathcal {M}}$ by $\Phi _{{\mathcal {Q}}}(1, \cdot )$ admits an expansion of the form
  $$\begin{aligned}&\Phi _{{\mathcal {Q}}}^* {\mathcal {M}}({\mathfrak {x}}) = \big (0, 0, {\mathcal {M}}^\bot ({\mathfrak {x}}) + \Upsilon ^\bot ({\mathfrak {x}}) + {{\mathcal {R}}}^\bot _N({\mathfrak {x}}) \big )\, , \\&\qquad \Upsilon ^\bot \in {{\mathcal {OF}}}^{p + q - 1}(m, N), \quad {{\mathcal {R}}}^\bot _N \in {{\mathcal {OS}}}^{p + q - 1}(N) \, . \end{aligned}$$
(iii) For any ${{\mathcal {Q}}}_1 \in {{\mathcal {OS}}}^q(N)$ with $q \in {\mathbb {N}}$, the pullback $\Phi _{{\mathcal {Q}}}^* {{\mathcal {Q}}}_1$ of ${\mathcal {Q}}_1$ by $\Phi _{{\mathcal {Q}}}(1, \cdot )$ admits an expansion of the form $\Phi _{{\mathcal {Q}}}^* {{\mathcal {Q}}}_1 = {{\mathcal {Q}}}_1 + {{\mathcal {OS}}}^{p + q - 1}(N)$.

#### Proof

(*i*) By (1.36), $\Phi _{{\mathcal {Q}}}^* X({\mathfrak {x}})$ can be expanded as

$$\begin{aligned} \Phi _{{\mathcal {Q}}}^* X({\mathfrak {x}}) = X({\mathfrak {x}}) + Z, \quad Z := \int _0^1 d \Phi _{{\mathcal {Q}}}(t, {\mathfrak {x}})^{- 1}[X, {{\mathcal {Q}}}](\Phi _{\mathcal {Q}}(t, {\mathfrak {x}}))\, d t\,. \end{aligned}$$

By applying Lemma 3.2, one gets that

$$\begin{aligned} {[}X, {{\mathcal {Q}}}] = \big (0, 0, \, \Upsilon ^\bot + {{\mathcal {R}}}^\bot _{[X, {{\mathcal {Q}}}] } \big ), \quad \Upsilon ^\bot \in {{\mathcal {OB}}}^{p + q - 1}(m, N), \quad {{\mathcal {R}}}^\bot _{[X, {{\mathcal {Q}}}] } \in {{\mathcal {OS}}}^{p + q - 1}(N + m)\,. \end{aligned}$$

Item (*i*) then follows by the definition of *Z*, the property (3.38), and Lemma 3.16. Items (*ii*) and (*iii*) can be proved similarly, using in addition Lemma 3.1 and Lemma 3.4. $\square$

We now consider a smoothing vector field ${{\mathcal {Q}}} \in {{\mathcal {OS}}}(N)$, $N \in {\mathbb {N}}$, of the form ${\mathcal {Q}} := {\mathcal {Q}}_0 + {\mathcal {Q}}_1$ where

3.39 $$\begin{aligned} \begin{aligned}&{\mathcal {Q}}_0 : = (0, 0, {{\mathcal {Q}}}^\bot _0), \qquad \ \ {{\mathcal {Q}}}^\bot _0({\mathfrak {x}}) \equiv {{\mathcal {Q}}}^\bot _0(\theta , y)[w] \in {{\mathcal {OS}}}_{w}^2(N), \\&{\mathcal {Q}}_1 := ({{\mathcal {F}}}_1, 0, {{\mathcal {Q}}}^\bot _1)\,, \qquad {{\mathcal {Q}}}^\bot _1({\mathfrak {x}}) \equiv {{\mathcal {Q}}}^\bot _1(\theta )[w, w] \in {{\mathcal {OS}}}_{ww}^2(N), \end{aligned} \end{aligned}$$

(cf. Definition 3.4(*iii*) for the definitions of ${{\mathcal {OS}}}_{w}^2(N)$ and ${{\mathcal {OS}}}_{ww}^2(N))$ and where for some $\sigma _N \ge 0$ and $0< \varepsilon _0 \equiv \varepsilon _0(N) < 1$, ${{\mathcal {F}}}_1$ has the form

3.40 $$\begin{aligned} \begin{aligned}&{{\mathcal {F}}}_1(\theta , w) := F_1(\theta )[w, w]\,, \qquad F_1 \in C^\infty \big ( {\mathbb {T}}^{S_+} \times [0, \varepsilon _0], \, {{\mathcal {B}}}_2(H^{\sigma _N}_\bot ({\mathbb {T}}_1), {\mathbb {R}}^{S_+}) \big ) , \end{aligned} \end{aligned}$$

(cf. (1.39) for the definition ${{\mathcal {B}}}_2(H^{\sigma _N}_\bot ({\mathbb {T}}_1), {\mathbb {R}}^{S_+})$). In the next lemma we compute an expansion of $\Phi _{{\mathcal {Q}}}^* X_{{\mathcal {N}}}$ where $X_{{\mathcal {N}}}$ is the normal form vector field defined in (3.22).

#### Lemma 3.18

For ${\mathcal {Q}} = {\mathcal {Q}}_0 + {\mathcal {Q}}_1$ as in (3.39), the following holds.

(i) The commutator $[X_{{\mathcal {N}}}, {\mathcal {Q}}_0] \in {{\mathcal {OS}}}^2(N - 3)$ has the form $\Upsilon ^{(1)} + {{\mathcal {OS}}}^3(N)$ where
  $$\begin{aligned} \Upsilon ^{(1)}({\mathfrak {x}}) = \Big (0, 0, \big ( [\mathrm{i} \Omega _\bot , \, {{\mathcal {Q}}}^\bot _0(\theta , y)]_{lin} + \omega \cdot \partial _\theta {{\mathcal {Q}}}^\bot _0(\theta , y) \big )[w] \Big )\,. \end{aligned}$$
(ii) The commutator $[X_{{\mathcal {N}}}, {\mathcal {Q}}_1] \in {{\mathcal {OS}}}^2(N - 3)$ has the form $\Upsilon ^{(2)} + {{\mathcal {OS}}}^3(N)$ where
  $$\begin{aligned} \begin{aligned}&\Upsilon ^{(2)}({\mathfrak {x}}) = \begin{pmatrix} \omega \cdot \partial _\theta F_1(\theta )[w, w] - F_1(\theta )[\mathrm{i} \Omega _\bot w, w] - F_1(\theta )[w, \mathrm{i} \Omega _\bot w] \\ 0 \\ \mathrm{i} \Omega _\bot {{\mathcal {Q}}}^\bot _1(\theta )[w, w]- {{\mathcal {Q}}}^\bot _1(\theta )[\mathrm{i} \Omega _\bot w, w] - {{\mathcal {Q}}}^\bot _1(\theta )[w, \mathrm{i} \Omega _\bot w] + \omega \cdot \partial _\theta {{\mathcal {Q}}}^\bot _1(\theta )[w, w] \end{pmatrix} \end{aligned} \end{aligned}$$
(iii) The pullback $\Phi _{{\mathcal {Q}}}^* X_{{\mathcal {N}}}$ is of the form $X_{{\mathcal {N}}} + \Upsilon ^{(1)} + \Upsilon ^{(2)} + {{\mathcal {OS}}}^3(N )$ with $\Upsilon ^{(1)}$ given by item (i) and $\Upsilon ^{(2)}$ given by item (ii).

#### Proof

(*i*): Arguing as in the proof of Lemma 3.9(*i*) (cf. (3.25)), one sees that $[X_{{\mathcal {N}}}, {\mathcal {Q}}_0] ({\mathfrak {x}})$ is of the form $\big (0, 0, [X_{{\mathcal {N}}}, \, {\mathcal {Q}}_0]^\bot ({\mathfrak {x}})\big )$ where
  $$\begin{aligned} {[}X_{{\mathcal {N}}}, {\mathcal {Q}}_0]^\bot ({\mathfrak {x}}) = \Big ( \big [\mathrm{i} \Omega _\bot , {{\mathcal {Q}}}^\bot _0(\theta , y)\big ]_{lin} + (\omega + \varepsilon {\widehat{\omega }}) \cdot \partial _\theta {{\mathcal {Q}}}^\bot _0(\theta , y) + \nabla _y Q(y) \cdot \partial _\theta {{\mathcal {Q}}}^\bot _0(\theta , y)\Big )[w]\, . \end{aligned}$$
  One has
  $$\begin{aligned}&\omega \cdot \partial _\theta {{\mathcal {Q}}}^\bot _0(\theta , y) [w] \in {{\mathcal {OS}}}_{w}^2(N), \qquad \varepsilon {\widehat{\omega }} \cdot \partial _\theta {{\mathcal {Q}}}^\bot _0(\theta , y) [w] \in {{\mathcal {OS}}}^3(N), \\&\qquad \nabla _y Q(y) \cdot \partial _\theta {{\mathcal {Q}}}^\bot _0(\theta , y)[w] \in {{\mathcal {OS}}}^3(N), \end{aligned}$$
  and since $\mathrm{i} \Omega _\bot$ is a Fourier multiplier of order three, it follows that $\big [\mathrm{i} \Omega _\bot , {{\mathcal {Q}}}^\bot _0(\theta , y)\big ]_{lin} w \in {{\mathcal {OS}}}_{ww}^2(N - 3)$. The claimed statement then follows.
(*ii*): Arguing as in the proof of Lemma 3.9(*ii*) (cf. (3.28)), and using that $F_1(\theta )[w, w]$ and ${{\mathcal {Q}}}^\bot _1(\theta )[w, w]$ are quadratic forms with respect to *w*, one sees that $Y := [X_{{\mathcal {N}}}, {\mathcal {Q}}_1]$ is of the form $Y = (Y^{(\theta )}, \, 0, \, Y^\bot )$ where
  $$\begin{aligned} Y^{(\theta )}({\mathfrak {x}})&= ( \omega + \varepsilon {\widehat{\omega }}) \cdot \partial _\theta F_1(\theta )[w, w] \\&\quad - F_1(\theta )[\mathrm{i} \Omega _\bot w, w] - F_1(\theta )[w, \mathrm{i} \Omega _\bot w] + \nabla _y Q(y) \cdot \partial _\theta F_1(\theta )[w, w] \\ Y^\bot ({\mathfrak {x}})&= \mathrm{i} \Omega _\bot {{\mathcal {Q}}}^\bot _1(\theta )[w, w]- {{\mathcal {Q}}}^\bot _1(\theta )[\mathrm{i} \Omega _\bot w, w] \\&\quad - {{\mathcal {Q}}}^\bot _1(\theta )[w, \mathrm{i} \Omega _\bot w] + ( \omega + \varepsilon {\widehat{\omega }}) \cdot \partial _\theta {{\mathcal {Q}}}^\bot _1(\theta )[w, w] \qquad \\&\qquad + \nabla _y Q(y) \cdot \partial _\theta {{\mathcal {Q}}}^\bot _1(\theta )[w, w]\,. \end{aligned}$$
  By (3.40), $\omega \cdot \partial _\theta F_1(\theta )[w, w]$, $F_1(\theta )[\mathrm{i} \Omega _\bot w, w]$, and $F_1(\theta )[w, \mathrm{i} \Omega _\bot w]$ are smooth functions and small of order two, whereas $\varepsilon {\widehat{\omega }} \cdot \partial _\theta F_1(\theta )[w, w]$ and $\nabla _y Q(y) \cdot \partial _\theta F_1(\theta )[w, w]$ are smooth functions and small of order three. (Here we used that by (3.21), $\nabla _y Q(y)$ is small of order one.) Furthermore, by the definition of ${{\mathcal {Q}}}^\bot _1$ one has $\omega \cdot \partial _\theta {{\mathcal {Q}}}^\bot _1(\theta )[w, w] \in {{\mathcal {OS}}}^2_{ww}(N)$, whereas $\varepsilon {\widehat{\omega }} \cdot \partial _\theta {{\mathcal {Q}}}^\bot _1(\theta )[w, w]$ and $\nabla _y Q(y) \cdot \partial _\theta {{\mathcal {Q}}}^\bot _1(\theta )[w, w]$ are in ${{\mathcal {OS}}}^3(N)$. Finally, since $\mathrm{i} \Omega _\bot$ is a Fourier multiplier of order three,
  $$\begin{aligned} \mathrm{i} \Omega _\bot {{\mathcal {Q}}}^\bot _1(\theta )[w, w]- {{\mathcal {Q}}}^\bot _1(\theta )[\mathrm{i} \Omega _\bot w, w] - {{\mathcal {Q}}}^\bot _1(\theta )[w, \mathrm{i} \Omega _\bot w] \in {{\mathcal {OS}}}_{ww}^2(N - 3)\,. \end{aligned}$$
  The claimed statement then follows.
(*iii*): By (1.36), $\Phi _{{\mathcal {Q}}}^* X_{\mathcal {N}}({\mathfrak {x}})$ can be expanded as
  $$\begin{aligned} \Phi _{{\mathcal {Q}}}^* X_{{\mathcal {N}}} = X_{{\mathcal {N}}} + [X_{{\mathcal {N}}}, {\mathcal {Q}}] + Z , \quad Z({\mathfrak {x}}) := \int _0^1 (1 - t) d \Phi _{\mathcal {Q}}(t, {\mathfrak {x}})^{- 1}[[X_{{\mathcal {N}}}, {\mathcal {Q}}], {\mathcal {Q}}](\Phi (t, {\mathfrak {x}}))\, d t\,. \end{aligned}$$
  By items (*i*) and (*ii*), the commutator $[X_{{\mathcal {N}}}, {\mathcal {Q}}]$ is in ${{\mathcal {OS}}}^2(N - 3)$, hence by Lemma 3.1, $[[X_{{\mathcal {N}}}, {\mathcal {Q}}], {\mathcal {Q}}] \in {{\mathcal {OS}}}^3(N - 3)$. By applying Lemma 3.17-(*iii*), one then infers that $Z \in {{\mathcal {OS}}}^3(N - 3)$. The claimed expansion then follows by items (*i*) and (*ii*). $\square$

In Sect. 5, we use Hamiltonian vector fields $X_{\mathcal {F}}$, corresponding to Hamiltonians ${\mathcal {F}}$, which are affine functions with respect to the normal component *w*. More precisely, ${\mathcal {F}}$ is assumed to be of the form

3.41 $$\begin{aligned} {{\mathcal {F}}}({\mathfrak {x}}) := {{\mathcal {F}}}_0(\theta , y) + \big \langle {{\mathcal {F}}}_1(\theta , y)\,,\, w \big \rangle \end{aligned}$$

where

3.42 $$\begin{aligned} \begin{aligned}&{{\mathcal {F}}}_0 \in C^\infty _b\big ( {\mathbb {T}}^{S_+} \times B_{S_+}(\delta ) \times [0, \varepsilon _0] , \, {\mathbb {R}}\big )\,, \\&\quad \qquad {{\mathcal {F}}}_1 \in C^\infty _b\big ( {\mathbb {T}}^{S_+} \times B_{S_+}(\delta )\times [0, \varepsilon _0] , \, H^s_\bot ({\mathbb {T}}_1) \big ), \quad \forall s \ge 0\,. \end{aligned} \end{aligned}$$

The Hamiltonian vector field generated by the Hamiltonian ${{\mathcal {F}}}$ is given by

3.43 $$\begin{aligned} X_{{\mathcal {F}}}({\mathfrak {x}}) = \big ( - \nabla _\theta {{\mathcal {F}}}({\mathfrak {x}}), \, \nabla _y {{\mathcal {F}}}({\mathfrak {x}}), \, \partial _x {{\mathcal {F}}}_1(\theta , y) \big ). \end{aligned}$$

The following lemma can be easily deduced by (3.41)–(3.43).

#### Lemma 3.19

The vector field $X_{{\mathcal {F}}}$ is a smoothing vector field of arbitrary order, i.e., $X_{{\mathcal {F}}} \in {{\mathcal {OS}}}(N)$ for any $N \in {\mathbb {N}}$. Moreover, if in addition ${{\mathcal {F}}}_0$ is small of order *p* and ${{\mathcal {F}}}_1$ is small of order *q*, then $\nabla _\theta {{\mathcal {F}}}$ is small of order $\mathrm{min}\{ p, q + 1\}$, $\nabla _y {{\mathcal {F}}}$ is small of order $\mathrm{min}\{p - 1, q\}$ and $\partial _x {{\mathcal {F}}}_1$ is small of order *q*.

## Reformulation of Theorem 1.1 and Normal Form Theorem

The goal of this section is to describe the normal form coordinates provided by [27, Theorem 1.1], specifically constructed to analyze perturbations of the KdV equations near finite gap solutions and then to express Eq. (1.4) with respect to these coordinates. The main results of this section are Theorem 4.2, which reformulates Theorem 1.1 in these novel coordinates, and Theorem 4.3 (Normal Form Theorem), which is the key ingredient into the proof of Theorem 4.2.

We begin by rephrasing [27, Theorem 1.1] in a form, adapted to our needs. Without further references, we use the notations introduced in Sect. 1.

### Theorem 4.1

Let $S_+ \subseteq {\mathbb {N}}$ be finite and $\Xi \subset {\mathbb {R}}_{> 0}^{S_+}$ be compact. Then for $\delta > 0$ sufficiently small with $\Xi + B_{S_+}(\delta ) \subset {\mathbb {R}}^{S_+}_{> 0}$ there exists a $C^\infty$- smooth family of canonical diffeomorphisms

$$\begin{aligned} \Psi _\mu : {{\mathcal {V}}}(\delta ) \rightarrow \Psi _\mu ({{\mathcal {V}}}(\delta )) \subseteq L^2_0({\mathbb {T}}_1)\,, \, {\mathfrak {x}} \mapsto q, \end{aligned}$$

parametrized by $\mu \in \Xi$, with the property that for any $\mu \in \Xi$, $\Psi _\mu ({\mathfrak {x}})$ satisfies

$$\begin{aligned} \Psi _\mu (\theta , y , 0) = \Psi _{S_+}(\theta , \mu + y), \quad \forall (\theta , y, 0) \in {{\mathcal {V}}}(\delta ) \, , \end{aligned}$$

and is compatible with the scale of Sobolev spaces $H^s_0({\mathbb {T}}_1)$, $s \in {\mathbb {Z}}_{\ge 0}$ (meaning that $\Psi _\mu \big ( {{\mathcal {V}}}(\delta ) \cap {\mathcal {E}}_s \big ) \subseteq H^s_0({\mathbb {T}}_1)$ and $\Psi _\mu : {{\mathcal {V}}}(\delta ) \cap {\mathcal {E}}_s \rightarrow H^s_0({\mathbb {T}}_1)$ is a $C^\infty$-diffeomorphism onto its image), so that the following holds:

(**AE1**): For any $N \in {\mathbb {N}}$, $\mu \in \Xi$, and ${\mathfrak {x}} = (\theta , y , w) \in {\mathcal {V}}(\delta )$, $\Psi ({\mathfrak {x}}) \equiv \Psi _\mu ({\mathfrak {x}})$ has an expansion of the form,
  $$\begin{aligned} \Psi ({\mathfrak {x}}) = \Psi _{S_+}(\theta , \mu + y) + w + \sum _{k = 1}^N a_{- k}({\mathfrak {x}}; \Psi ) \, \partial _x^{- k} w + {{\mathcal {R}}}_{N}({\mathfrak {x}}; \Psi ) \, , \end{aligned}$$
  where ${{\mathcal {R}}}_{N}(\theta , y , 0; \Psi ) = 0$ and where for any $s \in {\mathbb {Z}}_{\ge 0}$ and $1 \le k \le N$,
  $$\begin{aligned} {{\mathcal {V}}}(\delta ) \rightarrow H^s({\mathbb {T}}_1),\, {\mathfrak {x}} \mapsto a_{- k}({\mathfrak {x}}; \Psi ), \qquad {{\mathcal {V}}}^s(\delta ) \rightarrow H^{s + N +1}({\mathbb {T}}_1),\, {\mathfrak {x}} \mapsto {{\mathcal {R}}}_N({\mathfrak {x}}; \Psi ) , \end{aligned}$$
  are $C^\infty$ maps (cf. (1.28) for the definition of ${{\mathcal {V}}}^s(\delta )$).
(**AE2**): For any ${\mathfrak {x}} = (\theta , y, w) \in {{\mathcal {V}}}^1(\delta )$ and $\mu \in \Xi$, the transpose $d \Psi _\mu ({\mathfrak {x}})^\top$ (with respect to the standard inner products) of the differential $d \Psi _\mu ({\mathfrak {x}}) : E_1 \rightarrow H^1_0({\mathbb {T}}_1)$ yields a bounded operator $d \Psi ({\mathfrak {x}} )^\top \equiv d \Psi _\mu ({\mathfrak {x}} )^\top : H^1_0({\mathbb {T}}_1) \rightarrow E_1$. For any ${\widehat{q}} \in H^1_0({\mathbb {T}}_1)$ and any integer $N \ge 1$, $d \Psi ({\mathfrak {x}} )^\top [{\widehat{q}}]$ admits an expansion of the form
  $$\begin{aligned} d \Psi ({\mathfrak {x}})^\top [{\widehat{q}}]= & {} \Big ( \, 0, \, 0, \, \Pi _\bot {\widehat{q}} +\Pi _\bot \sum _{k = 1}^N a_{- k}({\mathfrak {x}}; {d \Psi ^\top }) \partial _x^{- k}{\widehat{q}} \, + \Pi _\bot \sum _{k = 1}^N ( \partial _x^{- k} w) {\mathcal {A}}_{- k}({\mathfrak {x}}; {d \Psi ^\top })[{\widehat{q}}] \, \Big )\\&\ + {{\mathcal {R}}}_{N}({\mathfrak {x}}; {d \Psi ^\top })[{\widehat{q}}] \end{aligned}$$
  where for any $s \in {\mathbb {N}}$ and $1 \le k \le N$,
  $$\begin{aligned} {{\mathcal {V}}}^1(\delta ) \rightarrow H^s({\mathbb {T}}_1)\,, \, {\mathfrak {x}} \mapsto a_{- k}({\mathfrak {x}}; {d \Psi ^\top })\,, \qquad {{\mathcal {V}}}^1(\delta ) \rightarrow {{\mathcal {B}}}(H^1_0({\mathbb {T}}_1), H^s({\mathbb {T}}_1))\,, \, {\mathfrak {x}} \mapsto {\mathcal {A}}_{- k}({\mathfrak {x}}; {d \Psi ^\top })\,, \end{aligned}$$
  and
  $$\begin{aligned} {{\mathcal {V}}}^s(\delta ) \rightarrow {{\mathcal {B}}}(H^s_0({\mathbb {T}}_1), E_{s + N +1}), \, {\mathfrak {x}} \mapsto {{\mathcal {R}}}_N({\mathfrak {x}}; {d \Psi ^\top })\, , \end{aligned}$$
  are $C^\infty$-smooth, bounded maps.
(**AE3**): For any $\mu \in \Xi$, the Hamiltonian ${{\mathcal {H}}}_\mu ^{kdv} := H^{kdv} \circ \Psi _\mu : {{\mathcal {V}}}^1(\delta ) \rightarrow {\mathbb {R}}$ is in normal form up to order three. More precisely, for any ${\mathfrak {x}} = (\theta , y, w) \in {{\mathcal {V}}}^1(\delta ),$ the Taylor expansion of ${{\mathcal {H}}}^{kdv} \equiv {{\mathcal {H}}}_\mu ^{kdv}$ at $(\theta , 0 , 0)$ with respect to *y* and *w* up to order three reads
  4.1 $$\begin{aligned} {{\mathcal {H}}}^{kdv} ({\mathfrak {x}}) = e + \omega \cdot y + \frac{1}{2} \Omega _{S_+} [y] \cdot y + \frac{1}{2}\big \langle D^{- 1}_\bot \Omega _\bot w, w \big \rangle + {{\mathcal {P}}}^{kdv} ({\mathfrak {x}}) \, , \end{aligned}$$
  where $$e := {{\mathcal {H}}}_\mu ^{kdv} (0, 0, 0)= H^{kdv}( \Psi _{S_+}(0, \mu )) $$,
  $$\begin{aligned} \omega = (\omega _n^{kdv}(\mu , 0))_{n \in S_+}\, , \qquad \Omega _{S_+} := (\partial _{I_j} \omega ^{kdv}_k (\mu , 0))_{j, k \in S_+}\, , \end{aligned}$$
  and for any $w = \sum _{n \in S^\bot } w_n e^{\mathrm{i} 2\pi n x}$, $D_\bot ^{- 1} w := \sum _{n \in S^\bot } \frac{1}{2 \pi n} w_n e^{\mathrm{i} 2 \pi n x}$, and (cf. (1.18))
  4.2 $$\begin{aligned} \Omega _\bot w: = \sum _{n \in S^\bot } \Omega _n w_n e^{\mathrm{i} 2\pi n x}\,, \qquad \Omega _n := \omega _n^{kdv}(\mu , 0)\,, \quad \forall n \in S^\bot \, . \end{aligned}$$
  Furthermore, ${{\mathcal {P}}}^{kdv} : {{\mathcal {V}}}^1(\delta ) \rightarrow {\mathbb {R}}$ is $C^\infty$-smooth, satisfies
  $$\begin{aligned} | {{\mathcal {P}}}^{kdv}({\mathfrak {x}}) | \lesssim ( |y| + \Vert w \Vert _1)^3 , \qquad \forall \, {\mathfrak {x}}= (\theta , y, w) \in {\mathcal {V}}^1(\delta )\, , \ \forall \, \mu \in \Xi \, , \end{aligned}$$
  and has the following property: for any integer $N \ge 1$ there exists an integer $\sigma _N \ge N$ (loss of regularity) so that $\nabla {{\mathcal {P}}}^{kdv}({\mathfrak {x}}){=} (\nabla _{\theta } {{\mathcal {P}}}^{kdv}({\mathfrak {x}}), \nabla _{y} {{\mathcal {P}}}^{kdv}({\mathfrak {x}}), \nabla _\bot {{\mathcal {P}}}^{kdv}({\mathfrak {x}}))$ admits an expansion of the form
  $$\begin{aligned} \nabla {{\mathcal {P}}}^{kdv}({\mathfrak {x}}) = \big (\, 0, \, 0, \, \Pi _\bot \sum _{k = 0}^N T_{a_{- k}({\mathfrak {x}}; {{\mathcal {P}}}^{kdv})} \, \partial _x^{- k} w \, \big ) + {{\mathcal {R}}}_N({\mathfrak {x}}; {{\mathcal {P}}}^{kdv}) , \end{aligned}$$
  where there exist integers $s_N > 0$ and $\sigma _N > 0$ so that for any $s \ge s_N$ and any $0 \le k \le N$,
  $$\begin{aligned} \begin{aligned}&{{\mathcal {V}}}^{s + \sigma _N}(\delta ) \rightarrow H^s({\mathbb {T}}_1), \, {\mathfrak {x}} \mapsto a_{- k}({\mathfrak {x}}; {{\mathcal {P}}}^{kdv}) \, , \qquad {{\mathcal {V}}}^{s \vee \sigma _N}(\delta ) \rightarrow {\mathcal {E}}_{s + N + 1}, \, {\mathfrak {x}} \mapsto {{\mathcal {R}}}_N({\mathfrak {x}}; {{\mathcal {P}}}^{kdv}) \end{aligned} \end{aligned}$$
  are $C^\infty$-smooth and satisfy for any $\theta \in {\mathbb {T}}_1^{S_+}$, $\mu \in \Xi$,
  $$\begin{aligned}&a_{- k}(\theta , 0, 0; {{\mathcal {P}}}^{kdv}) = 0, \quad {{\mathcal {R}}}_N(\theta , 0, 0; {{\mathcal {P}}}^{kdv}) = 0, \\&\quad \quad \partial _y {{\mathcal {R}}}_N(\theta , 0, 0; {{\mathcal {P}}}^{kdv}) = 0, \quad d_\bot {{\mathcal {R}}}_N(\theta , 0, 0; {{\mathcal {P}}}^{kdv}) = 0. \end{aligned}$$

Here $T_{a_k({\mathfrak {x}}; {{\mathcal {P}}}^{kdv})}$ denotes the operator of para-multiplication with $a_k({\mathfrak {x}}; {{\mathcal {P}}}^{kdv})$ (cf. Definition 2.1).

### Remark 4.1

Since $\Omega _{- n} = - \Omega _n$ for any $n \in S^\bot$ (cf. (1.12), (1.18)), the Fourier multiplyer $\mathrm{i} \Omega _\bot$ is a real operator. In view of the expansion (4.1) and the identity $\partial _x D^{- 1} = \mathrm{i}$, the component of the Hamiltonian vector field ${{\mathcal {H}}}^{kdv}_\mu$ in the normal direction is given by

$$\begin{aligned} \partial _x \nabla _\bot {{\mathcal {H}}}^{kdv}({\mathfrak {x}}) = \mathrm{i} \Omega _\bot w + \partial _x \nabla _\bot {{\mathcal {P}}}^{kdv}({\mathfrak {x}})\,. \end{aligned}$$

Next, we want to express Eq. (1.4) in the normal form coordinates provided by Theorem 4.1. To this end we write the nonlinear vector field *F*(*u*) in the coordinates $(\theta , y, z)$. Recall that $F(u) = \partial _x \nabla { P}_f(u)$ where ${P}_f(u) := \int _0^1 f(x, u(x))\, d x$ and *f* is given by (1.7).

### Proposition 4.1

Let $N \in {\mathbb {N}}$. Then there exist integers $s_N> 0$, $\sigma _N > 0$ so that for any perturbation $P_f (u) = \int _0^1 f(x, u(x))\, d x$ with *f* $C^{\infty }$-smooth, the following holds. For any $\mu \in \Xi$, the gradient of

4.3 $$\begin{aligned} {{\mathcal {P}}}_{f} \equiv {{\mathcal {P}}}_{f, \mu } := P_f \circ \Psi _\mu : {{\mathcal {V}}}^1(\delta ) \rightarrow {\mathbb {R}}\end{aligned}$$

admits an expansion of the form

$$\begin{aligned} \nabla {{\mathcal {P}}}_f ({\mathfrak {x}}) = \big (0, 0, \, \Pi _\bot \sum _{k = 0}^N T_{a_{ - k}({\mathfrak {x}}; \nabla {\mathcal {P}}_f )} \partial _x^{- k}w \big ) + {{\mathcal {R}}}_N({\mathfrak {x}}; \nabla {\mathcal {P}}_f) \, , \end{aligned}$$

where for any $s \ge s_N$ and for any $0 \le k \le N$, the maps

$$\begin{aligned} {{\mathcal {V}}}^{s + \sigma _N}(\delta ) \rightarrow H^s({\mathbb {T}}_1), \, {\mathfrak {x}} \mapsto a_{- k}({\mathfrak {x}}; \nabla {\mathcal {P}}_f)\, , \qquad {{\mathcal {V}}}^s(\delta ) \rightarrow E_{s + N + 1}, \, {\mathfrak {x}} \mapsto {{\mathcal {R}}}_N({\mathfrak {x}}; \nabla {\mathcal {P}}_f) \end{aligned}$$

are $$C^\infty $$-smooth.

### Proof

One has

4.4 $$\begin{aligned} \begin{aligned}&\nabla {P}_f(u)(x) = \partial _{\zeta } f(x, u(x))\,. \end{aligned} \end{aligned}$$

By the Bony para-linearization formula (cf. [37, Section 5.2.3]) for the composition operator, one gets that

4.5 $$\begin{aligned} \nabla {P}_f(u)(x) = \partial _{\zeta } f(x, u(x))= T_{\partial _{\zeta }^2 f(x, u(x))} u + {{\mathcal {R}}}_{f}(u) \end{aligned}$$

where there exists $s_N > N$ (large) so that for any integer $s \ge s_N$, the map ${\mathcal {R}}_f: H^s({\mathbb {T}}_1) \rightarrow H^{s + N + 1}({\mathbb {T}}_1)$ is $C^\infty$-smooth. Note that ${{\mathcal {R}}}_{f}(u)$ contains the zeroth order term $\partial _{\zeta } f(x, 0)$ of the Taylor expansion of $\partial _\zeta f(x, \zeta )$ at $\zeta =0$. By Theorem 4.1-$\mathbf{(AE2)}$, $d \Psi ({\mathfrak {x}})^\top [{\widehat{q}}]$ has an expansion of the form

4.6 $$\begin{aligned}&\Big ( \, 0, \, 0, \, \Pi _\bot [{\widehat{q}}] + \Pi _\bot \sum _{k = 1}^N a_{- k}({\mathfrak {x}}; d \Psi ^\top ) \partial _x^{- k}{\widehat{q}} \, + \Pi _\bot \sum _{k = 1}^N (\partial _x^{- k} w) {\mathcal {A}}_{- k}({\mathfrak {x}}; d \Psi ^\top )[{\widehat{q}}] \Big ) \nonumber \\&\quad + {{\mathcal {R}}}_{N}({\mathfrak {x}}; d \Psi ^\top )[{\widehat{q}}] \, , \end{aligned}$$

where the maps ${{\mathcal {V}}}(\delta ) \rightarrow H^s({\mathbb {T}}_1), \, {\mathfrak {x}} \mapsto a_k({\mathfrak {x}}; d \Psi ^\top )$,

$$\begin{aligned}&{{\mathcal {V}}}^1(\delta ) \rightarrow {{\mathcal {B}}}(H^1_0({\mathbb {T}}_1), H^s({\mathbb {T}}_1)), \, {\mathfrak {x}} \mapsto {\mathcal {A}}_k({\mathfrak {x}}; d \Psi ^\top ), \\&\quad \qquad {{\mathcal {V}}}^s(\delta ) \rightarrow {{\mathcal {B}}}(H_0^s({\mathbb {T}}_1), E_{s+ N + 1}), \, {\mathfrak {x}} \mapsto {{\mathcal {R}}}_{N}({\mathfrak {x}}; d \Psi ^\top ), \end{aligned}$$

are $C^\infty$-smooth, bounded maps. Using the expansion of $\Psi ({\mathfrak {x}})$ provided by Theorem 4.1-**(AE1)**,

4.7 $$\begin{aligned} \Psi ({\mathfrak {x}}) = \Psi _{S_+}(\theta , \mu + y) + w + \sum _{k = 1}^N a_{- k}({\mathfrak {x}}; \Psi ) \partial _x^{- k} w + {{\mathcal {R}}}_N({\mathfrak {x}}; \Psi ) \end{aligned}$$

together with the para-product formula (2.3) and Lemma 2.3, one obtains

4.8 $$\begin{aligned} \begin{aligned} (\nabla { P}_f)(\Psi ({\mathfrak {x}}))&= \sum _{k = 0}^N T_{a_{ - k}({\mathfrak {x}}; \nabla { P}_f \circ \Psi )} \partial _x^{- k} w + {{\mathcal {R}}}_N({\mathfrak {x}}; \nabla { P}_f \circ \Psi ), \\&\quad \qquad a_{ 0}({\mathfrak {x}}; \nabla { P}_f \circ \Psi ) = \partial _{\zeta }^2 f(x, \Psi ({\mathfrak {x}})), \end{aligned} \end{aligned}$$

where there exist integers $\sigma _N \ge 0$ and $s_N \ge 0$ so that for any $s \ge s_N$ and $0 \le k \le N$, the maps

$$\begin{aligned} {{\mathcal {V}}}^{s + \sigma _N} \rightarrow H^s({\mathbb {T}}_1), \, {\mathfrak {x}} \mapsto a_{- k}({\mathfrak {x}}; \nabla { P}_f \circ \Psi ), \quad {{\mathcal {V}}}^s(\delta ) \rightarrow E_{s + N + 1}, \, {\mathfrak {x}} \mapsto {{\mathcal {R}}}_N({\mathfrak {x}}; \nabla { P}_f \circ \Psi ) , \end{aligned}$$

are $C^\infty$-smooth. The expansion of $\nabla {{\mathcal {P}}}_f({\mathfrak {x}}) = d \Psi ({\mathfrak {x}})^\top (\nabla { P}_f)(\Psi ({\mathfrak {x}}))$ is then computed by using the one of $d\Psi ({\mathfrak {x}})^\top$, provided by Theorem 4.1-**(AE2)**. For any $1 \le k \le N$ , we thus need to compute the expansion of the sum

$$\begin{aligned} \sum _{k=1}^N a_{- k}({\mathfrak {x}}; d \Psi ^\top ) \partial _x^{- k} \nabla {P}_f(\Psi ({\mathfrak {x}})) + ( \partial _x^{- k} w) {\mathcal {A}}_{- k}({\mathfrak {x}}; d \Psi ^\top )[\nabla { P}_f(\Psi ({\mathfrak {x}}))]. \end{aligned}$$

By (4.8) and using the para-product formula (2.3) one obtains

$$\begin{aligned} \begin{aligned}&\Pi _\bot \sum _{k = 1}^N a_{ - k}({\mathfrak {x}}; d \Psi ^\top ) \partial _x^{- k} \nabla P_f(\Psi ({\mathfrak {x}})) + (\partial _x^{-k}w){\mathcal {A}}_{- k}({\mathfrak {x}}; d \Psi ^\top )[\nabla P_f(\Psi ({\mathfrak {x}}))] \\&\quad = \Pi _\bot \sum _{k = 1}^N \Big ( T_{a_{- k}({\mathfrak {x}}; d \Psi ^\top )} \partial _x^{- k} \nabla P_f(\Psi ({\mathfrak {x}})) + T_{ \partial _x^{- k} \nabla P_f(\Psi ({\mathfrak {x}})) } a_{- k}({\mathfrak {x}}; d \Psi ^\top ) \Big ) \\&\qquad + {{\mathcal {R}}}^{(B)}\big (a_{- k}({\mathfrak {x}}; d \Psi ^\top ) ,\, \partial _x^{- k} \nabla P_f(\Psi ({\mathfrak {x}})) \big ) \\&\qquad + \Pi _\bot \sum _{k = 1}^N T_{{\mathcal {A}}_{- k}({\mathfrak {x}}; d \Psi ^\top )[\nabla P_f(\Psi ({\mathfrak {x}}))]} \partial _x^{- k} w + T_{\partial _x^{- k}w} {\mathcal {A}}_{- k}({\mathfrak {x}}; d \Psi ^\top )[\nabla P_f(\Psi ({\mathfrak {x}}))] \\&\qquad + \Pi _\bot \sum _{k = 1}^N {{\mathcal {R}}}^{(B)}\big ({\mathcal {A}}_{- k}({\mathfrak {x}}; d \Psi ^\top )[\nabla P_f(\Psi ({\mathfrak {x}}))] ,\, \partial _x^{- k} w \big ) \\&\quad = \Pi _\bot \sum _{k = 1}^N \Big ( T_{a_{- k}({\mathfrak {x}}; d \Psi ^\top )} \partial _x^{- k} \nabla P_f(\Psi ({\mathfrak {x}})) + T_{{\mathcal {A}}_{- k}({\mathfrak {x}}; d \Psi ^\top )[\nabla P_f(\Psi ({\mathfrak {x}}))]} \partial _x^{- k} w \Big ) + {{\mathcal {R}}}_N^{(1)}({\mathfrak {x}}) \end{aligned} \end{aligned}$$

where

4.9 $$\begin{aligned} \begin{aligned} {{\mathcal {R}}}_{N}^{(1)} ({\mathfrak {x}})&:= \Pi _\bot \sum _{k = 1}^N T_{ \partial _x^{- k} \nabla P_f(\Psi ({\mathfrak {x}})) } a_{- k}({\mathfrak {x}}; d \Psi ^\top ) + T_{\partial _x^{- k}w } {\mathcal {A}}_{- k}({\mathfrak {x}}; d \Psi ^\top )[\nabla P_f(\Psi ({\mathfrak {x}}))] \\&\qquad + \Pi _\bot \sum _{k = 1}^N \Big ( {{\mathcal {R}}}^{(B)}\big (a_{- k}({\mathfrak {x}}; d \Psi ^\top )\,,\, \partial _x^{- k} \nabla P_f(\Psi ({\mathfrak {x}})) \big ) \\&\qquad + {{\mathcal {R}}}^{(B)}\big ({\mathcal {A}}_{- k}({\mathfrak {x}}; d \Psi ^\top )[\nabla P_f(\Psi ({\mathfrak {x}}))]\,,\, \partial _x^{- k} w \big ) \Big )\,. \end{aligned} \end{aligned}$$

By applying Theorem 4.1-$\mathbf{(AE1)}$,$\mathbf{(AE2)}$, and Lemma 2.2, one obtains, after increasing $s_N$ if needed, that for any $s \ge s_N$, the map ${{\mathcal {V}}}^s(\delta ) \rightarrow E_{s + N + 1}$, ${\mathfrak {x}} \mapsto {{\mathcal {R}}}^{(1)}_{N}({\mathfrak {x}})$ is $C^\infty$-smooth. By the expansion given in (4.8) and by applying Lemma 2.5 (composition of para-differential operators), one then gets the following identity for the normal component $(\nabla {{\mathcal {P}}}_f)^\bot$ of $\nabla {{\mathcal {P}}}_f$,

$$\begin{aligned} \begin{aligned} (\nabla {{\mathcal {P}}}_f)^\bot ({\mathfrak {x}})&= \Pi _\bot [\nabla P_f(\Psi ({\mathfrak {x}}))] + \Pi _\bot \sum _{k = 1}^N \Big ( T_{a_{- k}({\mathfrak {x}}; d \Psi ^\top )} \partial _x^{- k} \nabla P_f(\Psi ({\mathfrak {x}})) \\&\quad + T_{{\mathcal {A}}_{- k}({\mathfrak {x}}; d \Psi ^\top )[\nabla P_f (\Psi ({\mathfrak {x}}))]} \partial _x^{- k} w \Big ) + {{\mathcal {R}}}_N^{(1)}({\mathfrak {x}})\\&= \Pi _\bot \sum _{k = 0 }^N T_{a_{ - k}({\mathfrak {x}}; \nabla {{\mathcal {P}}}_f)} \partial _x^{- k} w + {{\mathcal {R}}}_N^{(2)}({\mathfrak {x}})\, , \quad a_{ 0}({\mathfrak {x}}; \nabla {{\mathcal {P}}}_f) = \partial _{\zeta }^2 f(x, w(x)) \, , \end{aligned} \end{aligned}$$

where there exist constants $s_N \ge N$ and $\sigma _N \ge N$ so that for any $s \ge s_N$ and any $0 \le k \le N$, the maps

$$\begin{aligned} {{\mathcal {V}}}^{s + \sigma _N}(\delta ) \rightarrow H^s({\mathbb {T}}_1), \, {\mathfrak {x}} \mapsto a_{- k}({\mathfrak {x}}; \nabla {{\mathcal {P}}}_f), \qquad {{\mathcal {V}}}^s(\delta ) \rightarrow H_\bot ^{s + N + 1}({\mathbb {T}}_1), \, {\mathfrak {x}} \mapsto {{\mathcal {R}}}_N^{(2)}({\mathfrak {x}}) , \end{aligned}$$

are $C^\infty$-smooth. Altogether we obtain

$$\begin{aligned} \nabla {{\mathcal {P}}}_f ({\mathfrak {x}}) = d \Psi ({\mathfrak {x}})^\top (\nabla { P}_f)(\Psi ({\mathfrak {x}})) = \big (0, 0, \, \Pi _\bot \sum _{k = 0}^N T_{a_{ - k}({\mathfrak {x}}; \nabla {\mathcal {P}}_f )} \partial _x^{- k}w \big ) + {{\mathcal {R}}}_N({\mathfrak {x}}; \nabla {\mathcal {P}}_f) \, , \end{aligned}$$

where

$$\begin{aligned} {{\mathcal {R}}}_N({\mathfrak {x}}; \nabla {{\mathcal {P}}}_f) := (0, 0, {{\mathcal {R}}}_N^{(2)}({\mathfrak {x}})) + {{\mathcal {R}}}_{N}({\mathfrak {x}}; d \Psi ^\top )[\nabla P_f(\Psi ({\mathfrak {x}}))]. \end{aligned}$$

One verifies in a straightforward way that ${{\mathcal {R}}}_N({\mathfrak {x}}; \nabla {{\mathcal {P}}}_f)$ has the stated properties. $\square$

Combining Theorem 4.1 and Proposition 4.1 together with Lemma 2.4 yields the following corollary.

### Corollary 4.1

(Expansion of ${\mathcal {H}}_\mu$). For any $\mu \in \Xi$, ${\mathcal {H}} \equiv {\mathcal {H}}_\mu = (H^{kdv} + \varepsilon P_f) \circ \Phi _\mu$ can be written as

4.10 $$\begin{aligned} {\mathcal {H}}({\mathfrak {x}}) = e + {{\mathcal {N}}}({\mathfrak {x}}) + {{\mathcal {P}}}({\mathfrak {x}}) , \qquad {{\mathcal {P}}} ({\mathfrak {x}}): = {{\mathcal {P}}}^{kdv}({\mathfrak {x}}) + \varepsilon {{\mathcal {P}}}_{f}({\mathfrak {x}}), \end{aligned}$$

where *e*, ${{\mathcal {N}}}$, and ${{\mathcal {P}}}^{kdv}$ are given by Theorem  4.1-**(AE3)** and ${{\mathcal {P}}}_{f}$ by Proposition 4.1. More precisely, $e = {{\mathcal {H}}}_\mu ^{kdv} (0, 0, 0)$ and for any ${\mathfrak {x}} = (\theta , y, w) \in {\mathcal {V}}^1(\delta )$,

4.11 $$\begin{aligned} {{\mathcal {N}}}(y, w) = \omega \cdot y + \frac{1}{2} \Omega _{S_+} [y] \cdot y + \frac{1}{2} \big \langle D_\bot ^{- 1} \Omega _\bot w\,,\, w \big \rangle , \end{aligned}$$

with

4.12 $$\begin{aligned} D^{- 1}_\bot w (x) = \sum _{j \in S^\bot } \frac{1}{2 \pi n} w_n e^{\mathrm{i} 2 \pi n x}, \qquad \Omega _\bot w(x) = \sum _{n \in S^\bot } \Omega _n w_n e^{\mathrm{i} 2 \pi n x}\,. \end{aligned}$$

The perturbation ${\mathcal {P}}$ is of the form (cf. Proposition 4.1)

4.13 $$\begin{aligned} {{\mathcal {P}}}({\mathfrak {x}}) = \varepsilon {{\mathcal {P}}}_L({\mathfrak {x}}) + {{\mathcal {P}}}_e({\mathfrak {x}}) \, , \quad \quad {{\mathcal {P}}}_L({\mathfrak {x}}) := {{\mathcal {P}}}_{00}(\theta ) + {{\mathcal {P}}}_{1 0}(\theta ) \cdot y + \big \langle {{\mathcal {P}}}_{0 1}(\theta )\,,\, w \big \rangle \, \qquad \end{aligned}$$

with ${{\mathcal {P}}}_e$, ${{\mathcal {P}}}_{00}(\theta )$, ${{\mathcal {P}}}_{1 0}(\theta )$, and ${{\mathcal {P}}}_{0 1}(\theta )$ having the following properties: there exist $0< \delta < 1$, $0< \varepsilon _0 < 1$, and an integer $\sigma > 0$ so that

4.14 $$\begin{aligned} \begin{aligned}&{{\mathcal {P}}}_{00} \in C^\infty ({\mathbb {T}}^{S_+}, \, {\mathbb {R}}), \quad {{\mathcal {P}}}_{10} \in C^\infty ({\mathbb {T}}^{S_+}, \, {\mathbb {R}}^{S_+}), \quad {{\mathcal {P}}}_{0 1} \in C^\infty ({\mathbb {T}}^{S_+}, \, H^s_\bot ({\mathbb {T}}_1)), \quad \forall s \ge 0\,, \\&{{\mathcal {P}}}_e \in C^\infty ({{\mathcal {V}}}^\sigma (\delta ) \times [0, \varepsilon _0], \, {\mathbb {R}}) \quad \text {small of order three}, \\&X_{{{\mathcal {P}}}_e} = (X_{{{\mathcal {P}}}_e}^{(\theta )}, X_{{{\mathcal {P}}}_e}^{(y)}, X_{{{\mathcal {P}}}_e}^\bot ) = (- \nabla _y {{\mathcal {P}}}_e, \nabla _\theta {{\mathcal {P}}}_e , \, \partial _x \nabla _\bot {{\mathcal {P}}}_e) \quad \text {small of order two}, \\&X_{{{\mathcal {P}}}_e}^\bot = \partial _x \nabla _\bot {{\mathcal {P}}}_e = {{\mathcal {OB}}}^2(1, N) + {{\mathcal {OS}}}^2(N), \quad \forall N \in {\mathbb {N}}\, , \end{aligned} \end{aligned}$$

(cf. Definition 3.1 and Definition 3.3 for the classes of vector fields ${{\mathcal {OB}}}^2(1, N)$ and respectively, ${{\mathcal {OS}}}^2(N)$).

### Remark 4.2

Since the constant *e* in (4.10) does not affect the Hamiltonian vector field $X_{\mathcal {H}}$, by notational convenience, we will suppress it in the sequel. The same convention will be used for any Hamiltonian under consideration.

We now reformulate Theorem 1.1 in the coordinates, provided by Theorem 4.1. By Corollary 4.1, the one parameter family of Hamiltonians ${\mathcal {H}} \equiv {\mathcal {H}}_\mu = (H^{kdv} + \varepsilon P_f) \circ \Phi _\mu$, $\mu \in \Xi$, is given by

4.15 $$\begin{aligned} {{\mathcal {H}}}({\mathfrak {x}}) = {{\mathcal {N}}}({\mathfrak {x}}) + \varepsilon {{\mathcal {P}}}_L({\mathfrak {x}}) + {{\mathcal {P}}}_e({\mathfrak {x}}) \end{aligned}$$

with ${\mathcal {N}}$ defined by (4.11) and ${\mathcal {P}}_L$, ${\mathcal {P}}_e$ by (4.13) (cf. Remark 4.2). Using that $\partial _x D_\bot ^{- 1} \Omega _\bot = \mathrm{i} \Omega _\bot$, the Hamiltonian vector field $X_{{\mathcal {H}}} = \big (- \nabla _y {\mathcal {H}}, \nabla _\theta {\mathcal {H}}, \partial _x\nabla _\bot {\mathcal {H}} \big )$ can be computed as

4.16 $$\begin{aligned} X_{{\mathcal {H}}}({\mathfrak {x}}) = \begin{pmatrix} - \omega - \Omega _{S_+} [y] - \varepsilon {{\mathcal {P}}}_{1 0}(\theta ) - \nabla _y {{\mathcal {P}}}_e({\mathfrak {x}}) \\ \varepsilon \nabla _\theta {{\mathcal {P}}}_L({\mathfrak {x}}) + \nabla _\theta {{\mathcal {P}}}_e({\mathfrak {x}}) \\ \mathrm{i} \Omega _\bot w + \varepsilon \partial _x {{\mathcal {P}}}_{0 1}(\theta ) + \partial _x \nabla _\bot {{\mathcal {P}}}_e({\mathfrak {x}}) \end{pmatrix} \end{aligned}$$

and the corresponding Hamiltonian equations are

4.17 $$\begin{aligned} \begin{aligned} \partial _t \theta&= - \omega - \Omega _{S_+} y - \varepsilon {{\mathcal {P}}}_{1 0}(\theta ) - \nabla _y {{\mathcal {P}}}_e({\mathfrak {x}}), \\ \partial _t y&= \varepsilon \nabla _\theta {{\mathcal {P}}}_L({\mathfrak {x}}) + \nabla _\theta {{\mathcal {P}}}_e({\mathfrak {x}}), \\ \partial _t w&= \mathrm{i} \Omega _\bot w + \varepsilon \partial _x {{\mathcal {P}}}_{0 1}(\theta ) + \partial _x \nabla _\bot {{\mathcal {P}}}_e({\mathfrak {x}}). \end{aligned} \end{aligned}$$

Except for the measure estimate (1.23), Theorem 1.1 is an immediate consequence of the following theorem. (We refer to Sect. 8 for a proof of (1.23).)

### Theorem 4.2

Let $f \in C^{\infty }({\mathbb {T}}_1 \times {\mathbb {R}}, \, {\mathbb {R}})$, $S_+$ be a finite subset of ${\mathbb {N}}$, $\tau$ be a number with $\tau > |S_+|$ (cf. (1.20)), and $\mu = \mu (\omega )$ with $\omega \in \Pi _\gamma$, $0< \gamma < 1$. Then for any integer *s* sufficiently large, there exists $0< \varepsilon _0 \equiv \varepsilon _0(s, \gamma ) < 1$ with the following properties: for any $0 < \varepsilon \le \varepsilon _0$ there exists $T \equiv T_{\varepsilon , s, \gamma } = O(\varepsilon ^{-2})$, so that for any initial data ${\mathfrak {x}}_0 = (\theta _0, y_0, w_0) \in {\mathbb {T}}^{S_+} \times {\mathbb {R}}^{S_+} \times H^s_\bot ({\mathbb {T}}_1)$, satisfying

4.18 $$\begin{aligned} | y_0|\,,\, \Vert w_0 \Vert _s \le \varepsilon \, , \end{aligned}$$

there exists a unique solution $t \mapsto {\mathfrak {x}}(t) = (\theta (t), y(t), w(t))$ of (4.17) with ${\mathfrak {x}}(0) = {\mathfrak {x}}_0$ and

$$\begin{aligned}&\theta \in C^1([- T, T], {\mathbb {T}}^{S_+}), \quad y \in C^1([- T, T], {\mathbb {R}}^{S_+}), \\&\quad \quad w \in C^0([- T, T], H^s_\bot (T_1)) \cap C^1([- T, T], H^{s - 3}_\bot ({\mathbb {T}}_1))\,. \end{aligned}$$

In addition, the solution satisfies $| y(t)|\,,\, \Vert w(t)\Vert _s \lesssim _{s, \gamma } \varepsilon$ for any $t \in [- T, T]$.

Theorem 4.2 is proved in Sect. 7. A key ingredient of its proof is the following result on normal forms.

### Theorem 4.3

(Normal Form Theorem). Let $f \in C^{\infty }({\mathbb {T}}_1 \times {\mathbb {R}}, \, {\mathbb {R}})$, $S_+$ be a finite subset of ${\mathbb {N}}$, $\tau$ be a number with $\tau > |S_+|$ (cf. (1.20)), and $\mu = \mu (\omega )$ with $\omega \in \Pi _\gamma$, $0< \gamma < 1$. Then there exists $\sigma _*>0$ so that for any integer $s \ge \sigma _*$ the following holds: there exist $0< \delta \equiv \delta (s, \gamma ) < 1$, $0 < \varepsilon _0\equiv \varepsilon _0(s, \gamma ) \ll \delta$, and $C_0 \equiv C_0(s, \gamma ) > 1$ with the property that for any $0 < \varepsilon \le \varepsilon _0$ there exists an invertible map ${\mathtt {\Phi }}$ with inverse ${\mathtt {\Phi }}^{-1}$ (cf. Remark 3.4),

4.19 $$\begin{aligned} {\mathtt {\Phi }}^{\pm 1}\in {{\mathcal {C}}}^\infty _b({{\mathcal {V}}}^s(\delta ), {{\mathcal {V}}}^s(C_0 \delta )), \qquad \quad {\mathtt {\Phi }}^{\pm 1}({\mathfrak {x}}) - {{\mathfrak {x}}} \ \ \text {small of order one}\, , \end{aligned}$$

so that the pull back $X = (X^{(\theta )}, X^{(y)}, X^\bot ) := {{\mathtt {\Phi }}}^* X_{{\mathcal {H}}_\mu }$ of the vector field $X_{{\mathcal {H}}_\mu }$ by ${\mathtt {\Phi }}$ has the form

4.20 $$\begin{aligned} \begin{aligned} X^{(\theta )}({\mathfrak {x}}) =&- \omega - \varepsilon {\widehat{\omega }} + {\mathtt {N}}^{(\theta )}(y, w) + {{\mathcal {O}}}_3^{(\theta )}({\mathfrak {x}})\, , \qquad X^{(y)}({\mathfrak {x}}) = {{\mathcal {O}}}_3^{(y)}({\mathfrak {x}})\, , \qquad \\&X^\bot ({\mathfrak {x}}) = \mathrm{i} \Omega _\bot w + {\mathtt {D}}^\bot ({\mathfrak {x}})[w] + \Pi _\bot T_{a({\mathfrak {x}})} \partial _x w + {{\mathcal {R}}}^\bot ({\mathfrak {x}}) \, , \qquad \end{aligned} \end{aligned}$$

where $${\widehat{\omega }} \in {\mathbb {R}}^{S_+}$$ and

4.21 $$\begin{aligned}&\mathtt {N}^{(\theta )} {\in } C^\infty _b \big (B_{S_+}(\delta ) {\times } B^{\sigma _*}_\bot (\delta ) {\times } [0, \varepsilon _0], \, {\mathbb {R}}^{S_+} \big ) \quad \text {small of order one } (\text {and independent of } \theta ), \qquad \qquad \quad \nonumber \\&{{\mathcal {O}}}_3^{(\theta )}, \ {{\mathcal {O}}}_3^{(y)} \in C^\infty _b( {{\mathcal {V}}}^{\sigma _*}(\delta ) \times [0, \varepsilon _0], \, {\mathbb {R}}^{S_+}) \quad \text {small of order three}, \nonumber \\&{\mathtt {D}}^\bot \in C^\infty _b \big ( {{\mathcal {V}}}^{\sigma _*}(\delta ) \times [0, \varepsilon _0], \, {{\mathcal {B}}}(H^{s}_\bot ({\mathbb {T}}_1), H^{s - 1}_\bot ({\mathbb {T}}_1) ) \big ) \quad \text {small of order one,} \nonumber \\&\quad {\mathtt {D}}^\bot \text { Fourier multiplier of the form } {\mathtt {D}}^\bot ({\mathfrak {x}})[w] = \sum _{j \in S^\bot } d_j({\mathfrak {x}}) w_j e^{\mathrm{i} 2 \pi j x}\text { with the properties}\nonumber \\&\quad d_j \in C^\infty _b \big ( {{\mathcal {V}}}^{\sigma _*}(\delta ) \times [0, \varepsilon _0], \, {\mathbb {R}}\big ), \ \ \forall j \in S^\bot , \qquad {\mathtt {D}}^\bot \text { skew-adjoint: } {\mathtt {D}}^\bot ({\mathfrak {x}})^\top = - {\mathtt {D}}^\bot ({\mathfrak {x}}), \nonumber \\&a\in C^\infty _b \big ( {{\mathcal {V}}}^{s + \sigma _*}(\delta ) \times [0, \varepsilon _0], \, H^s({\mathbb {T}}_1) \big ) \quad \text {small of order two}, \qquad \qquad \qquad \nonumber \\&{{\mathcal {R}}}^\bot \in C^\infty _b\big ({{\mathcal {V}}}^s(\delta ) \times [0, \varepsilon _0], \, H^s_\bot ({\mathbb {T}}_1) \big ) \quad \text {small of order three.} \qquad \qquad \qquad \end{aligned}$$

The proof of Theorem 4.3 is given in Sect. 7. The transformation $\mathtt {\Phi }$ is obtained as the composition of several transformations, constructed in Sects. 5–6.

## Smoothing Normal Form Steps

As part of the proof of Theorem 4.3, the aim of this section is to normalize terms in the Taylor expansion of the Hamiltonian ${{\mathcal {H}}}$ (cf. (4.15)), which are affine with respect to the normal coordinate *w* and homogeneous of order at most three with respect to the coordinates *y*, *w* and the parameter $\varepsilon$ (cf. *Overview of the proof of Theorem*  1.1 in Sect. 1). The main result of this section is the following one.

### Proposition 5.1

Let $f \in C^{\infty }({\mathbb {T}}_1 \times {\mathbb {R}}, \, {\mathbb {R}})$, $S_+$ be a finite subset of ${\mathbb {N}}$, $\tau$ be a number with $\tau > |S_+|$ (cf. (1.20)), and $\mu = \mu (\omega )$ with $\omega \in \Pi _\gamma$, $0< \gamma < 1$. Then for any $N \in {\mathbb {N}}$, there exist integers $s_N > 0$, $\sigma _N > 0$ so that for any $s \ge s_N$, there exist $0< \delta \equiv \delta (s, \gamma , N) <1$ and $0 < \varepsilon _0\equiv \varepsilon _0(s, \gamma , N) \ll \delta$ with the following properties: for any $0 < \varepsilon \le \varepsilon _0$ there exists an invertible symplectic transformation $\Phi$ with inverse $\Phi ^{-1}$ so that

5.1 $$\begin{aligned} \Phi ^{\pm 1}\in {{\mathcal {C}}}^\infty _b({{\mathcal {V}}}^s(\delta ) \times [0, \varepsilon _0], {{\mathcal {V}}}^s(2 \delta )) \, , \qquad \Phi ^{\pm 1}({\mathfrak {x}}) - {{\mathfrak {x}}} \quad \text {small of order one}\, , \end{aligned}$$

and so that the Hamiltonian ${{\mathcal {H}}}^{(3)} := {{\mathcal {H}}} \circ \Phi$ (cf. (4.2)) has the form

5.2 $$\begin{aligned} {{\mathcal {H}}}^{(3)}({\mathfrak {x}}) = {{\mathcal {N}}}^{(3)}({\mathfrak {x}}) + {{\mathcal {K}}}({\mathfrak {x}}) \, , \qquad {{\mathcal {N}}}^{(3)}({\mathfrak {x}}) := \omega \cdot y + \varepsilon {\widehat{\omega }} \cdot y + \frac{1}{2} \big \langle D_\bot ^{- 1} \Omega _\bot w\,,\, w \big \rangle + Q(y)\, .\nonumber \\ \end{aligned}$$

Here ${\widehat{\omega }} \equiv {\widehat{\omega }}(\varepsilon ) \in {\mathbb {R}}^{S_+}$ is an affine function of $\varepsilon$, $Q(y) \equiv Q(y, \varepsilon )$ is small of order two, a polynomial of degree three in *y* and an affine function of $\varepsilon$, and the components of the Hamiltonian vector field $X_{{\mathcal {K}}} = (X^{(\theta )}_{{\mathcal {K}}}, X^{(y)}_{{\mathcal {K}}}, X^\bot _{{\mathcal {K}}}) = (- \nabla _y {\mathcal {K}}, \, \nabla _\theta {\mathcal {K}}, \, \partial _x \nabla _\bot {\mathcal {K}})$, corresponding to the Hamiltonian ${{\mathcal {K}}}$, satisfy the following properties: $X^{(\theta )}_{{\mathcal {K}}}({\mathfrak {x}})$ is of the form $\Upsilon _2^{(\theta )}(\theta )[w, w] + \Upsilon _3^{(\theta )}({\mathfrak {x}})$ with

$$\begin{aligned}&\Upsilon _2^{(\theta )} \in C^\infty _b({\mathbb {T}}^{S_+}, \, {\mathcal {B}}_2(H^{\sigma _N}_\bot ({\mathbb {T}}_1), \, {\mathbb {R}}^{S_+})), \\&\qquad \qquad \Upsilon _3^{(\theta )} \in C_b^\infty ({{\mathcal {V}}}^{\sigma _N}(\delta ) \times [0, \varepsilon _0], \, {\mathbb {R}}^{S_+}), \, \text {small of order three,} \end{aligned}$$

and

5.3 $$\begin{aligned} X^{(y)}_{{\mathcal {K}}} \in C^\infty _b( {{\mathcal {V}}}^{\sigma _N}(\delta ) \times [0, \varepsilon _0], \, {\mathbb {R}}^{S_+}), \, \text {small of order three,} \quad X^\bot _{{\mathcal {K}}}({\mathfrak {x}}) = {\Upsilon }^\bot ({\mathfrak {x}}) + {{\mathcal {R}}}^\bot _N({\mathfrak {x}}), \nonumber \\ \end{aligned}$$

where

$$\begin{aligned} {\Upsilon }^\bot = {{\mathcal {OB}}}^2_{w}(1, N) + {{\mathcal {OB}}}^2_{ww}(1, N) + {{\mathcal {OB}}}^3(1, N)\,, \quad {{\mathcal {R}}}^\bot _N = {{\mathcal {OS}}}^2_w(N) + {{\mathcal {OS}}}^2_{ww}(N) + {{\mathcal {OS}}}^3(N)\,. \end{aligned}$$

In the remaining part of this section we prove Proposition 5.1. The transformation $\Phi$ is obtained as the composition $\Phi ^{(1)} \circ \Phi ^{(2)} \circ \Phi ^{(3)}$ of three symplectic transformations $\Phi ^{(j)}$, $1 \le j \le 3$.

**Normalization of** ${{\mathcal {P}}}_L$ **up to** $O(\varepsilon ^2)$.

The aim of this first step is to construct a symplectic transformation $\Phi ^{(1)}$ so that ${{\mathcal {P}}}_L({\mathfrak {x}}) {\mathop {=}\limits ^{(4.13)}} \varepsilon \big ( {{\mathcal {P}}}_{00}(\theta ) + {{\mathcal {P}}}_{10}(\theta ) \cdot y + \langle {{\mathcal {P}}}_{01}(\theta )\,,\,w \rangle \big ),$ when expressed in the new coordinates, is in normal form up to order $\varepsilon ^2$. We construct $\Phi ^{(1)}$ as the time one flow of a Hamiltonian flow corresponding to a Hamiltonian of the form

$$\begin{aligned} \varepsilon {{\mathcal {F}}}^{(1)}({\mathfrak {x}}) = \varepsilon {{\mathcal {F}}}_{00}^{(1)}(\theta ) + \varepsilon {{\mathcal {F}}}_{10}^{(1)}(\theta ) \cdot y + \varepsilon \langle {{\mathcal {F}}}_{01}^{(1)}(\theta )\,,\,w \rangle \end{aligned}$$

where

5.4 $$\begin{aligned} {{\mathcal {F}}}_{00}^{(1)} \in C^\infty \big ({\mathbb {T}}^{S_+}, \, {\mathbb {R}}\big ), \quad {{\mathcal {F}}}_{1 0}^{(1)} \in C^\infty \big ({\mathbb {T}}^{S_+}, \, {\mathbb {R}}^{S_+} \big ), \quad {{\mathcal {F}}}_{01}^{(1)} \in C^\infty \big ({\mathbb {T}}^{S_+}, \, H^n_\bot ({\mathbb {T}}_1) \big ), \ \ \forall \, n \ge 0 ,\nonumber \\ \end{aligned}$$

will be chosen to serve our needs. The Hamiltonian vector field corresponding to the Hamiltonian $\varepsilon {{\mathcal {F}}}^{(1)}({\mathfrak {x}})$,

$$\begin{aligned} X_{\varepsilon {{\mathcal {F}}}^{(1)}} ({\mathfrak {x}}) = \Big ( - \varepsilon {{\mathcal {F}}}_{10}^{(1)}(\theta ), \ \varepsilon \big ( \nabla _\theta {{\mathcal {F}}}_{00}^{(1)}(\theta ) + \nabla _\theta {{\mathcal {F}}}_{10}^{(1)}(\theta )\cdot y + \nabla _\theta \langle {{\mathcal {F}}}_{01}^{(1)}(\theta )\,,\,w \rangle \big )\,, \ \varepsilon \partial _x {{\mathcal {F}}}_{01}^{(1)}(\theta ) \Big ) \, , \end{aligned}$$

is small of order one and by Lemma 3.19 arbitrarily smoothing. It means that $X_{\varepsilon {{\mathcal {F}}}^{(1)}} \in {{\mathcal {OS}}}^1(N)$ for any integer $N \ge 1$ (cf. Definition 3.3). Denote by $\Phi ^{(1)}(\tau , \cdot ) \equiv \Phi _{\varepsilon {{\mathcal {F}}}^{(1)}}(\tau , \cdot )$ the flow of $X_{\varepsilon {{\mathcal {F}}}^{(1)}}$. For any given $N \in {\mathbb {N}},$ there exists an integer $s_N >0$ with the property that for any $s \ge s_N$, there exist $0< \delta \equiv \delta (s, \gamma , N) < 1$ and $0< \varepsilon _0 \equiv \varepsilon _0(s, \gamma , N) < 1$ (small), so that $\Phi ^{(1)}(\tau , \cdot ) \in C^\infty _b({\mathcal {V}}^s(\delta ) \times [0, \varepsilon _0], {\mathcal {V}}^s(2\delta ))$ for any $-1 \le \tau \le 1$. The inverse of the time one flow map $\Phi ^{(1)} := \Phi ^{(1)}(1, \cdot )$ is then given by $(\Phi ^{(1)})^{- 1} = \Phi ^{(1)}(- 1, \cdot )$ (cf. Remark 3.4) and by Lemma 3.16,

5.5 $$\begin{aligned} \Phi ^{(1)}(\tau , \cdot ) ({\mathfrak {x}}) - {{\mathfrak {x}}} \in {{\mathcal {OS}}}^1(N)\, , \qquad \forall \, -1 \le \tau \le 1 \,. \end{aligned}$$

We now compute ${{\mathcal {H}}}^{(1)} := {{\mathcal {H}}} \circ \Phi ^{(1)}$ by separately expanding the terms appearing in (4.13). By (1.33) (Lie expansion), (5.5) (properties of $\Phi ^{(1)}$) and (1.32) (Poisson bracket) one has

$$\begin{aligned} \begin{aligned}&{{\mathcal {N}}} \circ \Phi ^{(1)}= {{\mathcal {N}}} + \varepsilon \{ {{\mathcal {N}}} ,\, {{\mathcal {F}}}^{(1)} \} + \varepsilon ^2 \int _0^1 (1 - \tau )\{ \{{{\mathcal {N}}} \,, \, {{\mathcal {F}}}^{(1)} \}\,,\,{{\mathcal {F}}}^{(1)}\} \circ \Phi ^{(1)}(\tau , \cdot )\, d \tau \,, \\&\{ {{\mathcal {N}}} ,\, {{\mathcal {F}}}^{(1)} \} = \omega \cdot \partial _\theta {{\mathcal {F}}}_{00}^{(1)}(\theta ) + \big (\, \omega \cdot \partial _\theta {{\mathcal {F}}}_{10}^{(1)}(\theta ) + \Omega _{S_+} [\nabla _\theta {{\mathcal {F}}}_{00}^{(1)}(\theta )] \big ) \cdot y \\&\qquad \qquad \qquad + \big \langle \big (\omega \cdot \partial _\theta + \mathrm{i} \Omega _\bot \big ){{\mathcal {F}}}_{0 1}^{(1)}(\theta ),\, w\big \rangle \\&\qquad \qquad \qquad + ( \Omega _{S_+} [y] \cdot \partial _\theta )({{\mathcal {F}}}_{10}^{(1)}(\theta ) \cdot y) + \big \langle (\Omega _{S_+} [y] \cdot \partial _\theta ) {{\mathcal {F}}}_{0 1}^{(1)}(\theta ), w \big \rangle \end{aligned} \end{aligned}$$

and by (1.33) (Lie expansion) and (4.14) (properties of ${{\mathcal {P}}}_e$)

$$\begin{aligned}&\varepsilon {{\mathcal {P}}}_L \circ \Phi ^{(1)} = \varepsilon {{\mathcal {P}}}_L + \varepsilon ^2 \int _0^1 \{ {{\mathcal {P}}}_L, {{\mathcal {F}}}^{(1)} \} \circ \Phi ^{(1)}(\tau , \cdot )\, d \tau \,, \\&\qquad {{\mathcal {P}}}_e \circ \Phi ^{(1)} \quad C^\infty -\text {smooth, small of order three}. \end{aligned}$$

Altogether, one obtains

5.6 $$\begin{aligned} {{\mathcal {H}}}^{(1)}&= {{\mathcal {N}}}+ \varepsilon \big ( \, \omega \cdot \partial _\theta {{\mathcal {F}}}_{00}^{(1)}(\theta ) + {{\mathcal {P}}}_{00}(\theta ) \big ) + \varepsilon \big (\, \omega \cdot \partial _\theta {{\mathcal {F}}}_{10}^{(1)}(\theta ) + {{\mathcal {P}}}_{10}(\theta ) + \Omega _{S_+}[\nabla _\theta {{\mathcal {F}}}_{00}^{(1)}(\theta )] \big ) \cdot y \nonumber \\&+ \varepsilon \big \langle \big (\omega \cdot \partial _\theta + \mathrm{i} \Omega _\bot \big ){{\mathcal {F}}}_{0 1}^{(1)} + {{\mathcal {P}}}_{0 1}\,,\, w\big \rangle + {{\mathcal {P}}}^{(1)} \, , \end{aligned}$$

5.7 $$\begin{aligned} {{\mathcal {P}}}^{(1)}&:= \varepsilon ^2 \int _0^1 (1 - \tau )\{ \{{{\mathcal {N}}} \,, \, {{\mathcal {F}}}^{(1)} \}\,,\,{{\mathcal {F}}}^{(1)}\} \circ \Phi ^{(1)}(\tau , \cdot )\, d \tau + \varepsilon ^2 \int _0^1 \{ {{\mathcal {P}}}_L, {{\mathcal {F}}}^{(1)} \} \circ \Phi ^{(1)}(\tau , \cdot )\, d \tau \nonumber \\&\quad + \varepsilon ( \Omega _{S_+} [y] \cdot \partial _\theta )({{\mathcal {F}}}_{10}^{(1)}(\theta ) \cdot y) + \varepsilon \big \langle (\Omega _{S_+}[y] \cdot \partial _\theta ) {{\mathcal {F}}}_{0 1}^{(1)}(\theta ), w \big \rangle + {{\mathcal {P}}}_e \circ \Phi ^{(1)}. \end{aligned}$$

Since the terms appearing in the second line of (5.7) are small of order three, the Hamiltonian ${{\mathcal {P}}}^{(1)}$ admits an expansion of the form

5.8 $$\begin{aligned} {{\mathcal {P}}}^{(1)} ({\mathfrak {x}}) = \varepsilon ^2 {{\mathcal {P}}}_{00}^{(1)}(\theta ) + {{\mathcal {P}}}^{(1)}_e\, , \end{aligned}$$

where ${{\mathcal {P}}}_{00}^{(1)} \in C^\infty ({\mathbb {T}}^{S_+}, {\mathbb {R}})$ and ${{\mathcal {P}}}^{(1)}_e$ is small of order three. In view of (5.6) and since $\Omega _{S_+} [\nabla _\theta {{\mathcal {F}}}^{(1)}_{00} ]$ has zero average in $\theta$, we consider the following system of homological equations for ${{\mathcal {F}}}_{00}^{(1)}$, ${{\mathcal {F}}}_{1 0}^{(1)}$, ${{\mathcal {F}}}_{0 1}^{(1)}$,

5.9 $$\begin{aligned} {\left\{ \begin{array}{ll} \omega \cdot \partial _\theta {{\mathcal {F}}}_{00}^{(1)} + {{\mathcal {P}}}_{00} = \langle {{\mathcal {P}}}_{00}\rangle _\theta \,, \\ \omega \cdot \partial _\theta {{\mathcal {F}}}_{10}^{(1)} + {{\mathcal {P}}}_{10} + \Omega _{S_+}[ \nabla _\theta {{\mathcal {F}}}^{(1)}_{00}] = \langle {{\mathcal {P}}}_{10} \rangle _\theta \,, \\ \big (\omega \cdot \partial _\theta + \mathrm{i} \Omega _\bot \big ){{\mathcal {F}}}_{0 1}^{(1)} + {{\mathcal {P}}}_{0 1} = 0\, . \end{array}\right. } \end{aligned}$$

Since by assumption $\omega \in \Pi _\gamma$, $0< \gamma < 1$, (cf. (1.19)), we can apply Lemmata B.1, B.2, to conclude that the system (5.9) has a unique solution ${{\mathcal {F}}}_{00}^{(1)}, {{\mathcal {F}}}_{1 0}^{(1)}, {{\mathcal {F}}}_{0 1}^{(1)}$ satisfying (5.4) and $\langle {{\mathcal {F}}}_{00}^{(1)}\rangle _\theta = 0$, $\langle {{\mathcal {F}}}_{1 0}^{(1)}\rangle _\theta = 0$. The Hamiltonian ${{\mathcal {H}}}^{(1)}$, defined in (5.6), then reads

5.10 $$\begin{aligned} {{\mathcal {H}}}^{(1)} = {{\mathcal {N}}} + \varepsilon \widehat{{\mathcal {N}}}_1 + \varepsilon ^2 {{\mathcal {P}}}_{00}^{(1)}(\theta ) + {{\mathcal {P}}}^{(1)}_e\,, \qquad \widehat{{\mathcal {N}}}_1(y) := \langle {{\mathcal {P}}}_{00}\rangle _\theta + \langle {{\mathcal {P}}}_{10} \rangle _\theta \cdot y \,. \end{aligned}$$

Since ${{\mathcal {P}}}_e^{(1)}$ is small of order three, its Hamiltonian vector field $X_{{{\mathcal {P}}}_e^{(1)}}$ is small of order two. For later use we discuss the normal component $X_{{{\mathcal {P}}}^{(1)}_e}^\bot$ of the vector field $X_{{{\mathcal {P}}}^{(1)}_e}$. Since $X_{\varepsilon {{\mathcal {F}}}^{(1)}} \in {{\mathcal {OS}}}^1(N)$, and $X^\bot _{ {{\mathcal {P}}}_e} = {{\mathcal {OB}}}^2(1, N) + {{\mathcal {OS}}}^2(N)$ (cf. (4.14)) it follows from Lemma 3.17 that $X^\bot _{{\mathcal {P}}_e \circ \Phi ^{(1)} } = {{\mathcal {OB}}}^2(1, N) + {{\mathcal {OS}}}^2(N)$. Arguing similarly for all the other terms in the definition of ${{\mathcal {P}}}_e^{(1)}$ (cf. (5.7), (5.8)) one can show that

5.11 $$\begin{aligned} X_{{{\mathcal {P}}}^{(1)}_e}^\bot = \partial _x \nabla _\bot {{\mathcal {P}}}_e^{(1)} = {{\mathcal {OB}}}^2(1, N) + {{\mathcal {OS}}}^2(N). \end{aligned}$$

**Normalization of** $\varepsilon ^2 {{\mathcal {P}}}_{00}^{(1)}(\theta )$. The aim of this second step is to normalize the term $\varepsilon ^2 {{\mathcal {P}}}_{00}^{(1)}(\theta )$ (small of order 2) in (5.10). To this end we construct a symplectic transformation $\Phi ^{(2)}$, given again by the time one flow of a Hamiltonian flow, corresponding to a Hamiltonian of the form $\varepsilon ^2 {{\mathcal {F}}}^{(2)}(\theta )$ with

5.12 $$\begin{aligned} {{\mathcal {F}}}^{(2)} \in C^\infty ({\mathbb {T}}^{S_+}, {\mathbb {R}}) \end{aligned}$$

being a function to be determined. The Hamiltonian vector field corresponding to the Hamiltonian $\varepsilon ^2 {{\mathcal {F}}}^{(2)}(\theta )$,

$$\begin{aligned} X_{\varepsilon ^2 {{\mathcal {F}}}^{(2)}}({\mathfrak {x}}) = \big ( 0, \, \varepsilon ^2 \nabla _\theta {{\mathcal {F}}}^{(2)}(\theta ), \, 0 \big ) \,. \end{aligned}$$

is small of order two and by Lemma 3.19 arbitrarily smoothing. It means that $X_{\varepsilon ^2 {{\mathcal {F}}}^{(2)}} \in {{\mathcal {OS}}}^2(N)$ for any integer $N \ge 1$ (cf. Definition 3.3). Denote by $\Phi ^{(2)}(\tau , \cdot ) \equiv \Phi _{\varepsilon ^2 {{\mathcal {F}}}^{(2)}}(\tau , \cdot )$ the flow of $X_{\varepsilon ^2 {{\mathcal {F}}}^{(2)}}$. For any given $N \in {\mathbb {N}},$ there exists an integer $s_N >0$ with the property that for any $s \ge s_N$, there exist $0< \delta \equiv \delta (s, \gamma , N) < 1$ and $0< \varepsilon _0 \equiv \varepsilon _0(s, \gamma , N) < 1$ (small), so that $\Phi ^{(2)}(\tau , \cdot ) \in C^\infty _b({\mathcal {V}}^s(\delta ) \times [0, \varepsilon _0], {\mathcal {V}}^s(2\delta ))$ for any $-1 \le \tau \le 1$. The inverse of the time one flow map $\Phi ^{(2)} := \Phi ^{(2)}(1, \cdot )$ is then given by $(\Phi ^{(2)})^{- 1} = \Phi ^{(2)}(- 1, \cdot )$ (cf. Remark 3.4) and by Lemma 3.16,

5.13 $$\begin{aligned} \Phi ^{(2)}(\tau , \cdot ) ({\mathfrak {x}}) - {{\mathfrak {x}}} \in {{\mathcal {OS}}}^2(N)\, , \qquad \forall \, -1 \le \tau \le 1 \,. \end{aligned}$$

We now compute ${{\mathcal {H}}}^{(2)} := {{\mathcal {H}}}^{(1)} \circ \Phi ^{(2)}$ by separately expanding the terms in (5.10). By (1.33) (Lie expansion), (5.13) (properties of $\Phi ^{(2)}$) and (1.32) (Poisson bracket) one has

$$\begin{aligned} \begin{aligned}&{{\mathcal {N}}} \circ \Phi ^{(2)} = {{\mathcal {N}}} + \varepsilon ^2 \{ {{\mathcal {N}}}, \, {{\mathcal {F}}}^{(2)} \} + \varepsilon ^4 \int _0^1 (1 - \tau )\{ \{{{\mathcal {N}}} \,, \, {{\mathcal {F}}}^{(2)} \}\,,\,{{\mathcal {F}}}^{(2)}\} \circ \Phi ^{(2)}(\tau , \cdot )\, d \tau \\&\qquad \qquad = {{\mathcal {N}}} + \varepsilon ^2 \omega \cdot \partial _\theta {{\mathcal {F}}}^{(2)}(\theta ) + \varepsilon ^4 \int _0^1 (1 - \tau )\{ \{{{\mathcal {N}}} \,, \, {{\mathcal {F}}}^{(2)} \}\,,\,{{\mathcal {F}}}^{(2)}\} \circ \Phi ^{(2)}(\tau , \cdot )\, d \tau \,, \\&\varepsilon \widehat{{\mathcal {N}}}_1 \circ \Phi ^{(2)} = \varepsilon \widehat{{\mathcal {N}}}_1 + \varepsilon ^3 \int _0^1 \{ \widehat{{\mathcal {N}}}_1, {{\mathcal {F}}}^{(2)} \} \circ \Phi ^{(2)}(\tau , \cdot )\, d \tau \,, \\&\varepsilon ^2 {{\mathcal {P}}}_{00}^{(1)} \circ \Phi ^{(2)} = \varepsilon ^2 {{\mathcal {P}}}_{00}^{(1)}(\theta ) + \varepsilon ^4 \int _0^1 \{ {{\mathcal {P}}}_{00}^{(1)}, {{\mathcal {F}}}^{(2)} \} \circ \Phi ^{(2)}(\tau , \cdot )\, d \tau \\&{{\mathcal {P}}}_e^{(1)} \circ \Phi ^{(2)} \quad C^\infty -\text {smooth, small of order three.} \end{aligned} \end{aligned}$$

Altogether, one obtains

5.14 $$\begin{aligned} {{\mathcal {H}}}^{(2)}= & {} {{\mathcal {H}}}^{(1)} \circ \Phi ^{(2)} = {{\mathcal {N}}}+ \varepsilon \widehat{{\mathcal {N}}}_1 + \varepsilon ^2 \big ( \omega \cdot \partial _\theta {{\mathcal {F}}}^{(2)}(\theta ) + {{\mathcal {P}}}_{00}^{(1)}(\theta ) \big ) + {{\mathcal {P}}}^{(2)}\,, \nonumber \\ {{\mathcal {P}}}^{(2)}:= & {} \varepsilon ^4 \int _0^1 (1 - \tau )\{ \{{{\mathcal {N}}} \,, \, {{\mathcal {F}}}^{(2)} \}\,,\,{{\mathcal {F}}}^{(2)}\} \circ \Phi ^{(2)}(\tau , \cdot )\, d \tau + \varepsilon ^3 \int _0^1 \{ \widehat{{\mathcal {N}}}_1, {{\mathcal {F}}}^{(2)} \} \circ \Phi ^{(2)}(\tau , \cdot )\, d \tau \nonumber \\&+ \varepsilon ^4 \int _0^1 \{ {{\mathcal {P}}}_{00}^{(1)}, {{\mathcal {F}}}^{(2)} \} \circ \Phi ^{(2)}(\tau , \cdot )\, d \tau + {{\mathcal {P}}}_e^{(1)} \circ \Phi ^{(2)}\,. \end{aligned}$$

Since ${{\mathcal {P}}}_e^{(1)}$ is $C^\infty$-smooth and small of order three, so is ${{\mathcal {P}}}^{(2)}$. In view of the formula for ${{\mathcal {H}}}^{(2)}$ in (5.14) we consider the following homological equation for ${{\mathcal {F}}}^{(2)}$,

5.15 $$\begin{aligned} \omega \cdot \partial _\theta {{\mathcal {F}}}^{(2)}(\theta )+ {{\mathcal {P}}}_{00}^{(1)}(\theta )= \langle {{\mathcal {P}}}_{00}^{(1)}\rangle _\theta \,. \end{aligned}$$

Since by assumption $\omega \in \Pi _\gamma$, $0< \gamma < 1$, (cf. (1.19)), we can apply Lemmata B.1, B.2, to conclude that (5.15) has a unique solution ${{\mathcal {F}}}^{(2)} \in C^\infty ({\mathbb {T}}^{S_+}, {\mathbb {R}})$ with $\langle {{\mathcal {F}}}^{(2)} \rangle _\theta = 0$. The Hamiltonian ${{\mathcal {H}}}^{(2)}$ in (5.14) then reads

5.16 $$\begin{aligned} {{\mathcal {H}}}^{(2)} = {{\mathcal {N}}} + \varepsilon \widehat{{\mathcal {N}}}_2 + {{\mathcal {P}}}^{(2)}\,, \quad \widehat{{\mathcal {N}}}_2 := \widehat{{\mathcal {N}}}_1 + \varepsilon \langle {{\mathcal {P}}}_{00}^{(1)} \rangle _\theta {\mathop {=}\limits ^{(5.10)}} \langle {{\mathcal {P}}}_{00}\rangle _\theta + \langle {{\mathcal {P}}}_{10} \rangle _\theta \cdot y + \varepsilon \langle {{\mathcal {P}}}_{00}^{(1)} \rangle _\theta \,.\nonumber \\ \end{aligned}$$

Since ${{\mathcal {P}}}^{(2)}$ is small of order three, its Hamiltonian vector field $X_{{{\mathcal {P}}}^{(2)}}$ is small of order two. For later use, we again discuss the normal component $X_{{{\mathcal {P}}}^{(2)}}^\bot$ of the vector field $X_{{{\mathcal {P}}}^{(2)}}$. Since $X_{\varepsilon ^2 {{\mathcal {F}}}^{(2)}} \in {{\mathcal {OS}}}^2(N)$, and $X^\bot _{ {{\mathcal {P}}}^{(1)}_e} = {{\mathcal {OB}}}^2(1, N) + {{\mathcal {OS}}}^2(N)$ (cf. (5.11)) it follows from Lemma 3.17 that $X^\bot _{{\mathcal {P}}^{(1)}_e \circ \Phi ^{(2)} } = {{\mathcal {OB}}}^2(1, N) + {{\mathcal {OS}}}^2(N)$. Arguing similarly for all the other terms in ${{\mathcal {P}}}^{(2)}$ (cf. (5.14) (5.7), (5.8), (5.10)) one shows that

5.17 $$\begin{aligned} X_{{{\mathcal {P}}}^{(2)}}^\bot = \partial _x \nabla _\bot {{\mathcal {P}}}^{(2)} = {{\mathcal {OB}}}^2(1, N) + {{\mathcal {OS}}}^2(N). \end{aligned}$$

**Normalization of terms affine in** *w*. The aim of this third step is to construct a symplectic coordinate transformation $\Phi ^{(3)}$, normalizing the terms in the Taylor expansion of ${{\mathcal {P}}}^{(2)}$ (cf. (5.16)) with respect to *y*, *w* at $(y, w) = (0, 0)$, which are homogeneous in $y, w, \varepsilon$ of order three, of degree at most one in *w*, and of degree at most two in $\varepsilon$. The Taylor expansion of ${{\mathcal {P}}}^{(2)}$ in *y*, *w*, $\varepsilon$ up to order four reads

$$\begin{aligned} \begin{aligned} {{\mathcal {P}}}^{(2)}({\mathfrak {x}}) =\,&\varepsilon ^3 {{\mathcal {P}}}_{00}^{(2)}(\theta ) + \varepsilon ^2 \big ( {{\mathcal {P}}}_{10}^{(2)}(\theta ) \cdot y + \langle {{\mathcal {P}}}_{0 1}^{(2)}(\theta ), w \rangle \big ) \\&\quad + \varepsilon \big ( {{\mathcal {P}}}_{2 0}^{(2)}(\theta )[y, y] + \langle {{\mathcal {P}}}_{11}^{(2)}(\theta )[y], w \rangle \big ) + \langle {{\mathcal {P}}}_{0 2}^{(2)}(\theta , y)[w], w \rangle \\&\quad + {{\mathcal {P}}}_{3 0}^{(2)}(\theta )[y,y,y] + \langle {{\mathcal {P}}}_{2 1}^{(2)}(\theta )[y, y], w \rangle + {{\mathcal {P}}}_{0 3}^{(2)}(\theta )[w,w,w] + {{\mathcal {O}}}_4({\mathfrak {x}}), \end{aligned} \end{aligned}$$

where for any $n \ge 0$,

5.18 $$\begin{aligned} \begin{aligned}&{{\mathcal {P}}}_{00}^{(2)} \in C^\infty ({\mathbb {T}}^{S_+}, \, {\mathbb {R}}) \,, \qquad \qquad {{\mathcal {P}}}_{10}^{(2)} \in C^\infty ({\mathbb {T}}^{S_+}, \, {\mathbb {R}}^{S_+}), \qquad \ \ {{\mathcal {P}}}_{0 1}^{(2)} \in C^\infty ({\mathbb {T}}^{S_+}, \, H^n_\bot ({\mathbb {T}}_1)), \\&{{\mathcal {P}}}_{2 0}^{(2)} \in C^\infty ({\mathbb {T}}^{S_+}, \, {{\mathcal {B}}}_2({\mathbb {R}}^{S_+})), \quad \ \ {{\mathcal {P}}}_{11}^{(2)} \in C^\infty \big ({\mathbb {T}}^{S_+}, \, {{\mathcal {B}}}({\mathbb {R}}^{S_+}, H^n_\bot ({\mathbb {T}}_1)) \big ), \\&{{\mathcal {P}}}_{3 0}^{(2)} \in C^\infty ({\mathbb {T}}^{S_+}, \, {{\mathcal {B}}}_3({\mathbb {R}}^{S_+}))\, , \quad \, {{\mathcal {P}}}_{2 1}^{(2)} \in C^\infty ({\mathbb {T}}^{S_+}, \, {{\mathcal {B}}}_2({\mathbb {R}}^{S_+}, H^n_\bot ({\mathbb {T}}_1))), \\&\quad \quad {{\mathcal {P}}}_{0 3}^{(2)} \in C^\infty ({\mathbb {T}}^{S_+}, \, {{\mathcal {B}}}_3(H^n_\bot ({\mathbb {T}}_1)), \quad {{\mathcal {P}}}_{0 2}^{(2)} \in C^\infty \big ( {\mathbb {T}}^{S_+} \times {\mathbb {R}}^{S_+} \times {\mathbb {R}}, \, {{\mathcal {B}}}(H^n_\bot ({\mathbb {T}}_1)) \big ),\\&\qquad {{\mathcal {O}}}_4({\mathfrak {x}}) \ \ C^\infty {\text {-smooth, small of order four.}}\\ \end{aligned} \end{aligned}$$

### Remark 5.1

In the above Taylor expansion of ${{\mathcal {P}}}^{(2)}$, we combined the terms which are of the order $(0 \, 2)$ and $(1 \,2)$ in the variables *y*, *w* and for notational convenience, denoted the combined term by $\langle {{\mathcal {P}}}_{0 2}^{(2)}(\theta , y)[w], w \rangle$. The map

$$\begin{aligned} {{\mathcal {P}}}_{0 2}^{(2)}: (\theta , y, \varepsilon ) \mapsto {{\mathcal {P}}}_{0 2}^{(2)}(\theta , y) \equiv {{\mathcal {P}}}_{0 2}^{(2)}(\theta , y, \varepsilon ) \end{aligned}$$

is linear in $y, \varepsilon$.

We split ${{\mathcal {P}}}^{(2)}$ as ${{\mathcal {P}}}^{(2)} = {{\mathcal {P}}}^{(2)}_1 + {{\mathcal {P}}}^{(2)}_2 + {{\mathcal {O}}}_4$ where

5.19 $$\begin{aligned} \begin{aligned} {{\mathcal {P}}}^{(2)}_1&:= \varepsilon ^2 {{\mathcal {P}}}^{(2)}_{10}(\theta ) \cdot y + \varepsilon {{\mathcal {P}}}^{(2)}_{2 0}(\theta )[y, y] + {{\mathcal {P}}}^{(2)}_{3 0}(\theta )[y,y,y] \\&\quad \quad + \varepsilon ^2 \big \langle {{\mathcal {P}}}^{(2)}_{0 1}(\theta ), w \big \rangle + \varepsilon \big \langle {{\mathcal {P}}}^{(2)}_{11}(\theta )[y], w \big \rangle + \big \langle {{\mathcal {P}}}^{(2)}_{2 1}(\theta )[y, y], w \big \rangle \\ {{\mathcal {P}}}^{(2)}_2&:= \varepsilon ^3 {{\mathcal {P}}}^{(2)}_{00}(\theta ) + \langle {{\mathcal {P}}}^{(2)}_{0 2}(\theta , y)[w], w \rangle + {{\mathcal {P}}}^{(2)}_{0 3}(\theta )[w,w,w] \, . \end{aligned} \end{aligned}$$

Note that ${{\mathcal {P}}}^{(2)}_1$ is affine in *w* and that the Hamiltonian vector field corresponding to the term $\varepsilon ^3 {{\mathcal {P}}}^{(2)}_{00}(\theta )$ is small of order three. The transformation $\Phi ^{(3)}$ is then defined as the time one flow of the Hamiltonian vector field $X_{{\mathcal {F}}^{(3)}}$ with a Hamiltonian ${\mathcal {F}}^{(3)}$ of the form

5.20 $$\begin{aligned} \begin{aligned}&{{\mathcal {F}}}^{(3)}({\mathfrak {x}}) := \varepsilon ^2 {{\mathcal {F}}}^{(3)}_{10}(\theta ) \cdot y + \varepsilon {{\mathcal {F}}}^{(3)}_{2 0}(\theta )[y, y] + {{\mathcal {F}}}^{(3)}_{3 0}(\theta )[y,y,y] \\&\qquad + \varepsilon ^2 \big \langle {{\mathcal {F}}}^{(3)}_{0 1}(\theta ), w \big \rangle + \varepsilon \big \langle {{\mathcal {F}}}^{(3)}_{11}(\theta )[y], w \big \rangle + \big \langle {{\mathcal {F}}}^{(3)}_{2 1}(\theta )[y, y], w \big \rangle \end{aligned} \end{aligned}$$

satisfying for any $n \ge 0$,

5.21 $$\begin{aligned} \begin{aligned}&{{\mathcal {F}}}^{(3)}_{10} \in C^\infty ({\mathbb {T}}^{S_+}, {\mathbb {R}}^{S_+}), \qquad \ {{\mathcal {F}}}^{(3)}_{2 0} \in C^\infty ({\mathbb {T}}^{S_+}, {{\mathcal {B}}}_2({\mathbb {R}}^{S_+})),\\&{{\mathcal {F}}}^{(3)}_{3 0} \in C^\infty ({\mathbb {T}}^{S_+}, {{\mathcal {B}}}_3({\mathbb {R}}^{S_+})), \quad {{\mathcal {F}}}^{(3)}_{0 1} \in C^\infty ({\mathbb {T}}^{S_+}, H^n_\bot ({\mathbb {T}}_1)), \ \ \\&{{\mathcal {F}}}^{(3)}_{11} \in C^\infty ({\mathbb {T}}^{S_+}, {{\mathcal {B}}}({\mathbb {R}}^{S_+}, H^n_\bot ({\mathbb {T}}_1))), \quad {{\mathcal {F}}}^{(3)}_{2 1} \in C^\infty ({\mathbb {T}}^{S_+}, {{\mathcal {B}}}_2({\mathbb {R}}^{S_+}, H^n_\bot ({\mathbb {T}}_1))). \end{aligned} \end{aligned}$$

The functions ${\mathcal {F}}^{(3)}_{i j}$ will be chosen according to our needs. By (5.20), (5.21), the Hamiltonian vector field $X_{{{\mathcal {F}}}^{(3)}}$ is small of order two and by Lemma 3.19 arbitrarily smoothing. It means that $X_{{{\mathcal {F}}}^{(3)}} \in {{\mathcal {OS}}}^2(N)$ for any integer $N \ge 1$ (cf. Definition 3.3). Denote by $\Phi ^{(3)}(\tau , \cdot ) \equiv \Phi _{ {{\mathcal {F}}}^{(3)}}(\tau , \cdot )$ the flow of $X_{ {{\mathcal {F}}}^{(3)}}$. For any given $N \in {\mathbb {N}},$ there exists an integer $s_N >0$ with the property that for any $s \ge s_N$, there exist $0< \delta \equiv \delta (s, \gamma , N) < 1$ and $0< \varepsilon _0 \equiv \varepsilon _0(s, \gamma , N) < 1$ (small), so that $\Phi ^{(3)}(\tau , \cdot ) \in C^\infty _b({\mathcal {V}}^s(\delta ) \times [0, \varepsilon _0], {\mathcal {V}}^s(2\delta ))$ for any $-1 \le \tau \le 1$. The inverse of the time one flow map $\Phi ^{(3)} := \Phi ^{(3)}(1, \cdot )$ is then given by $(\Phi ^{(3)})^{- 1} = \Phi ^{(3)}(- 1, \cdot )$ and by Lemma 3.16,

5.22 $$\begin{aligned} \Phi ^{(3)}(\tau , \cdot ) ({\mathfrak {x}}) - {{\mathfrak {x}}} \in {{\mathcal {OS}}}^2(N)\, , \qquad \forall \, -1 \le \tau \le 1 \,. \end{aligned}$$

We now compute ${{\mathcal {H}}}^{(3)} := {{\mathcal {H}}}^{(2)} \circ \Phi ^{(3)}$ by expanding separately the terms in (5.16). By (1.33) (Lie expansion), (5.22) (properties of $\Phi ^{(3)}$), (5.19) (splitting of ${\mathcal {P}}^{(2)}$), (5.20)–(5.21) (properties of ${\mathcal {F}}^{(3)}$), and (1.32) (Poisson bracket)

$$\begin{aligned} {{\mathcal {N}}} \circ \Phi ^{(3)} = {{\mathcal {N}}} + \{ {{\mathcal {N}}}\,,\, {{\mathcal {F}}}^{(3)} \} + \int _0^1 ( 1- \tau ) \{ \{{{\mathcal {N}}}, {{\mathcal {F}}}^{(3)} \}, {{\mathcal {F}}}^{(3)} \} \circ \Phi ^{(3)}(\tau , \cdot )\, d \tau \end{aligned}$$

can be expanded as

5.23 $$\begin{aligned} {{\mathcal {N}}} \circ \Phi ^{(3)}&= {{\mathcal {N}}} + \varepsilon ^2 (\omega \cdot \partial _\theta ) {{\mathcal {F}}}_{1 0}^{(3)}(\theta ) \cdot y + \varepsilon ( \omega \cdot \partial _\theta ) {{\mathcal {F}}}_{2 0}^{(3)}(\theta )[y, y] + (\omega \cdot \partial _\theta ) {{\mathcal {F}}}_{3 0}^{(3)}(\theta )[y,y,y] \nonumber \\&\quad + \varepsilon ^2 \big \langle (\omega \cdot \partial _\theta + \mathrm{i} \Omega _\bot ) {{\mathcal {F}}}_{0 1}^{(3)}(\theta ),w\big \rangle + \varepsilon \big \langle (\omega \cdot \partial _\theta + \mathrm{i} \Omega _\bot ) {{\mathcal {F}}}_{1 1}^{(3)}(\theta )[y],w\big \rangle \nonumber \\&\quad + \big \langle (\omega \cdot \partial _\theta + \mathrm{i} \Omega _\bot ) {{\mathcal {F}}}_{2 1}^{(3)}(\theta )[y, y],w\big \rangle \nonumber \\&\quad +(\Omega _{S_+} [y] \cdot \partial _\theta ) {{\mathcal {F}}}^{(3)} + \int _0^1 ( 1- \tau ) \{ \{{{\mathcal {N}}}, {{\mathcal {F}}}^{(3)} \}, {{\mathcal {F}}}^{(3)} \} \circ \Phi ^{(3)}(\tau , \cdot )\, d \tau \,, \\ \widehat{{\mathcal {N}}}_2 \circ \Phi ^{(3)}&= \widehat{{\mathcal {N}}}_2 + \int _0^1 \{ \widehat{{\mathcal {N}}}_2\,,\, {{\mathcal {F}}}^{(3)} \} \circ \Phi ^{(3)}(\tau , \cdot )\, d \tau , \nonumber \\&\quad {{\mathcal {P}}}^{(2)} \circ \Phi ^{(3)} = {{\mathcal {P}}}_1^{(2)} + {{\mathcal {P}}}_2^{(2)} + \int _0^1 \{ {{\mathcal {P}}}^{(2)}, {{\mathcal {F}}}^{(3)} \} \circ \Phi ^{(3)}(\tau , \cdot )\, d \tau \,.\nonumber \end{aligned}$$

Since ${{\mathcal {P}}}^{(2)}$ (cf. (5.16)), ${{\mathcal {F}}}^{(3)}$ (cf. (5.20)) are small of order three and in view of the definition of ${\mathcal {N}}$, $\widehat{{\mathcal {N}}}_2$ (cf. (5.16)), $\{ \{ {{\mathcal {N}}}, \, {{\mathcal {F}}}^{(3)} \}$, ${{\mathcal {F}}}^{(3)} \}$, $\varepsilon \{ \widehat{{\mathcal {N}}}_2, {{\mathcal {F}}}^{(3)} \}$, and $\{{{\mathcal {P}}}^{(2)}, {{\mathcal {F}}}^{(3)} \}$ are small of order four. Hence the Hamiltonian ${{\mathcal {H}}}^{(3)}$ takes the form

5.24 $$\begin{aligned} {{\mathcal {H}}}^{(3)}&= {{\mathcal {N}}} + \varepsilon \widehat{{\mathcal {N}}}_2 + \varepsilon ^2\Big ( \omega \cdot \partial _\theta {{\mathcal {F}}}_{1 0}^{(3)}(\theta ) + {{\mathcal {P}}}_{1 0}^{(2)}(\theta ) \Big )\cdot y + \varepsilon \Big (\omega \cdot \partial _\theta {{\mathcal {F}}}_{2 0}^{(3)}(\theta ) + {{\mathcal {P}}}_{2 0}^{(2)}(\theta )\Big )[y, y] \nonumber \\&\quad + \Big (\omega \cdot \partial _\theta {{\mathcal {F}}}_{3 0}^{(3)}(\theta ) + {{\mathcal {P}}}_{3 0}^{(2)}(\theta ) \Big )[y,y,y] + \varepsilon ^2 \big \langle (\omega \cdot \partial _\theta + \mathrm{i} \Omega _\bot ) {{\mathcal {F}}}_{0 1}^{(3)}(\theta ) + {{\mathcal {P}}}_{0 1}^{(2)}(\theta ), \, w\big \rangle \nonumber \\&\quad + \varepsilon \big \langle (\omega \cdot \partial _\theta + \mathrm{i} \Omega _\bot ) {{\mathcal {F}}}_{1 1}^{(3)}(\theta )[y] + {{\mathcal {P}}}_{11}^{(2)}(\theta )[y],w\big \rangle \nonumber \\&\quad \quad + \big \langle (\omega \cdot \partial _\theta + \mathrm{i} \Omega _\bot ) {{\mathcal {F}}}_{2 1}^{(3)}(\theta )[y, y] + {{\mathcal {P}}}_{21}^{(2)}(\theta )[y, y],w\big \rangle + {{\mathcal {P}}}_2^{(2)} + {{\mathcal {O}}}_4 \end{aligned}$$

where ${{\mathcal {O}}}_4$ comprises all the terms which are small of order four. In view of (5.24), we consider the following system of homological equations for ${\mathcal {F}}^{(3)}_{i j}$,

5.25 $$\begin{aligned}&\omega \cdot \partial _\theta {{\mathcal {F}}}_{j 0}^{(3)}(\theta ) + {{\mathcal {P}}}_{j 0}^{(2)}(\theta ) = \big \langle {{\mathcal {P}}}_{j 0}^{(2)} \big \rangle _\theta , \quad 1 \le j \le 3, \nonumber \\&(\omega \cdot \partial _\theta + \mathrm{i} \Omega _\bot ) {{\mathcal {F}}}^{(3)}_{0 1}(\theta ) + {{\mathcal {P}}}^{(2)}_{0 1}(\theta ) = 0\,, \quad (\omega \cdot \partial _\theta + \mathrm{i} \Omega _\bot ) {{\mathcal {F}}}^{(3)}_{1 1}(\theta ) + {{\mathcal {P}}}^{(2)}_{11}(\theta ) = 0\,, \nonumber \\&(\omega \cdot \partial _\theta + \mathrm{i} \Omega _\bot ) {{\mathcal {F}}}^{(3)}_{2 1}(\theta ) + {{\mathcal {P}}}^{(2)}_{21}(\theta ) = 0. \end{aligned}$$

Since by assumption $\omega \in \Pi _\gamma$, $0< \gamma < 1$ (cf. (1.19)), we can apply Lemmata B.1, B.2, to conclude that the system (5.25) has a unique solution ${{\mathcal {F}}}_{ij}^{(3)}$, satisfying the properties (5.21). The Hamiltonian ${{\mathcal {H}}}^{(3)}$ in (5.24) then reads

5.26 $$\begin{aligned} \begin{aligned}&{{\mathcal {H}}}^{(3)} = {{\mathcal {N}}}^{(3)}+ {{\mathcal {K}}}, \qquad {{\mathcal {N}}}^{(3)} := \omega \cdot y + \varepsilon {\widehat{\omega }} \cdot y + \frac{1}{2} \big \langle D^{- 1} \Omega _\bot w\,,\, w \big \rangle + Q(y) \,, \qquad {{\mathcal {K}}} := {{\mathcal {P}}}_2^{(2)} + {{\mathcal {O}}}_4 \,. \\&{\widehat{\omega }} := \langle {{\mathcal {P}}}_{10} \rangle _\theta + \varepsilon \langle {{\mathcal {P}}}_{1 0}^{(2)} \rangle _\theta \,, \qquad Q(y) := \frac{1}{2} \Omega _{S_+} y \cdot y + \varepsilon \langle {{\mathcal {P}}}_{2 0}^{(2)} \rangle _\theta [y, y] + \langle {{\mathcal {P}}}_{3 0}^{(2)} \rangle _\theta [y, y, y] \, . \end{aligned} \end{aligned}$$

Here we dropped the irrelevant constant term $\varepsilon \langle {{\mathcal {P}}}_{00} \rangle _\theta + \varepsilon ^2 \langle {{\mathcal {P}}}_{00}^{(1)} \rangle _\theta$ from the Hamiltonan ${{\mathcal {H}}}^{(3)}$ (cf. Remark 4.2). By (5.19), (5.26), the components of the Hamiltonian vector field $X_{{{\mathcal {H}}}^{(3)}} = (X_{{{\mathcal {H}}}^{(3)}}^{(\theta )}, X_{{{\mathcal {H}}}^{(3)}}^{(y)}, X_{{{\mathcal {H}}}^{(3)}}^\bot )$ read

5.27 $$\begin{aligned} \begin{aligned}&X_{{\mathcal {H}}^{(3)}}^{(\theta )}({\mathfrak {x}}) = - \omega - \varepsilon {\widehat{\omega }} - \nabla _y Q(y) - \nabla _y {{\mathcal {P}}}_2^{(2)}({\mathfrak {x}}) - \nabla _y {{\mathcal {O}}}_4({\mathfrak {x}}) \,, \\&X_{{{\mathcal {H}}}^{(3)}}^{(y)}({\mathfrak {x}}) = \nabla _\theta {{\mathcal {P}}}_2^{(2)}({\mathfrak {x}}) + \nabla _\theta {{\mathcal {O}}}_4({\mathfrak {x}})\,, \\&X_{{{\mathcal {H}}}^{(3)}}^\bot ({\mathfrak {x}}) = \mathrm{i} \Omega _\bot w + \partial _x \nabla _\bot {{\mathcal {P}}}_2^{(2)}({\mathfrak {x}}) + \partial _x \nabla _\bot {{\mathcal {O}}}_4({\mathfrak {x}}) \, . \end{aligned} \end{aligned}$$

Since ${{\mathcal {P}}}_2^{(2)}$ is a $C^\infty -$smooth and small of order three and ${{\mathcal {O}}}_4$ is small of order four, $\nabla _\theta {{\mathcal {P}}}_2^{(2)}$ is small of order three and $\nabla _\theta {{\mathcal {O}}}_4$ is small of order four, implying that

5.28 $$\begin{aligned} X_{{{\mathcal {H}}}^{(3)}}^{(y)} \in C^\infty _b \big ( {{\mathcal {V}}}^{\sigma _N}(\delta ) \times [0, \varepsilon _0], \, {\mathbb {R}}^{S_+} \big ) \quad \text {small of order three} \end{aligned}$$

for some $\sigma _N > 0$. Towards $X_{{{\mathcal {H}}}^{(3)}}^{(\theta )}$, note that $\nabla _y {{\mathcal {O}}}_4$ is small of order three and that $\nabla _y {{\mathcal {P}}}_2^{(2)}$ (cf. (5.19)) is small of order two and has the additional property of being at least quadratic with respect to *w*. Therefore

5.29 $$\begin{aligned} \nabla _y {{\mathcal {P}}}_2^{(2)}({\mathfrak {x}})+ \nabla _y {{\mathcal {O}}}_4({\mathfrak {x}}) = \Upsilon _2^{(\theta )}(\theta )[w, w] + \Upsilon _3^{(\theta )}({\mathfrak {x}})\, , \end{aligned}$$

where

$$\begin{aligned}&\Upsilon _2^{(\theta )} \in C^\infty \big ( {\mathbb {T}}^{S_+}, \, {{\mathcal {B}}}_2(H^{\sigma _N}_\bot ({\mathbb {T}}_1), \, {\mathbb {R}}^{S_+})\big ), \\&\quad \qquad \Upsilon _3^{(\theta )} \in C^\infty \big ({{\mathcal {V}}}^{\sigma _N}(\delta ) \times [0, \varepsilon _0], \, {\mathbb {R}}^{S_+} \big ) \ \ \text {small of order three} \end{aligned}$$

for some $\sigma _N > 0$. For later use, we discuss the normal component $X_{{\mathcal {K}}}^\bot$ of the vector field $X_{{\mathcal {K}}}$. Since by (5.19), ${{\mathcal {P}}}^{(2)}_2 = \varepsilon ^3 {{\mathcal {P}}}^{(2)}_{00}(\theta ) + \langle {{\mathcal {P}}}^{(2)}_{0 2}(\theta , y)[w], w \rangle + {{\mathcal {P}}}^{(2)}_{0 3}(\theta )[w,w,w]$ (cf. Remark 5.1) one infers that

5.30 $$\begin{aligned} X_{{\mathcal {K}}}^\bot ({\mathfrak {x}}) = \partial _x \nabla _\bot {{\mathcal {P}}}_2^{(2)}({\mathfrak {x}}) + \partial _x \nabla _\bot {{\mathcal {O}}}_4({\mathfrak {x}}) = 2 \partial _x {{\mathcal {P}}}^{(2)}_{0 2}(\theta , y)[w] + \Upsilon ^\bot _2 (\theta )[w, w] + \Upsilon ^\bot _3({\mathfrak {x}})\nonumber \\ \end{aligned}$$

where $\Upsilon ^\bot _3({\mathfrak {x}})$ is small of order three. Since $X_{{{\mathcal {F}}}^{(3)}} \in {{\mathcal {OS}}}^2(N)$ and $\partial _x \nabla _\bot {{\mathcal {P}}}^{(2)} {=} {{\mathcal {OB}}}^2(1, N) {+} {{\mathcal {OS}}}^2(N)$ (cf. 5.16, 5.17) and in view of the definition of ${\mathcal {O}}_4$ (cf. (5.24)) it then follows from Lemma 3.17 that

5.31 $$\begin{aligned} \begin{aligned}&\qquad \qquad \qquad \qquad \partial _x {{\mathcal {P}}}^{(2)}_{0 2}(\theta , y)[w] = {{\mathcal {OB}}}^2_w(1, N) + {{\mathcal {OS}}}^2_w(1, N)\,, \\&\Upsilon ^\bot _2 (\theta )[w, w] = {{\mathcal {OB}}}^2_{ww}(1, N) + {{\mathcal {OS}}}^2_{ww}(N)\,, \qquad \Upsilon ^\bot _3({\mathfrak {x}}) = {{\mathcal {OB}}}^3(1, N) + {{\mathcal {OS}}}^3( N)\, . \end{aligned}\nonumber \\ \end{aligned}$$

### Proof of Proposition 5.1.

We define $\Phi := \Phi ^{(1)} \circ \Phi ^{(2)} \circ \Phi ^{(3)}$ where $\Phi ^{(1)}, \Phi ^{(2)}, \Phi ^{(3)}$ are the symplectic coordinate transformations, given in the paragraphs above. Using the properties (5.5), (5.13), (5.22) of $\Phi ^{(1)}$, $\Phi ^{(2)}$, and $\Phi ^{(3)}$, respectively one shows that there exists an integer $s_N > 0$ with the property that for any $s \ge s_N$ there exist $0< \delta \equiv \delta (s, \gamma , N) < 1$ and $0< \varepsilon _0 \equiv \varepsilon _0(s, \gamma , N) < 1$ so that (5.1) holds,

$$\begin{aligned} \Phi ^{\pm 1}\in {{\mathcal {C}}}^\infty _b({{\mathcal {V}}}^s(\delta ) \times [0, \varepsilon _0], \, {{\mathcal {V}}}^s(2 \delta )) \, , \qquad \Phi ^{\pm 1}({\mathfrak {x}}) - {{\mathfrak {x}}} \quad \text {small of order one}\, . \end{aligned}$$

Since ${{\mathcal {K}}} = {{\mathcal {P}}}_2^{(2)} + {{\mathcal {O}}}_4$, the remaining statements of Proposition 5.1 then follow by (5.28) - (5.31). $\square$

## Normalization Steps by Para-Differential Calculus

The goal of this section is to normalize terms in the vector field $X_{\mathcal {K}}$, which are linear or quadratic in the variable *w*, where $X_{\mathcal {K}}$ denotes the Hamiltonian vector field of the Hamiltonian ${\mathcal {K}}$ of Proposition 5.1. This is achieved in three steps, described in the following three subsections, by using para-differential calculus.

### Normalization of terms linear or quadratic in *w*

The aim of this subsection is to reduce to constant coefficients the terms in the normal component $X^\bot \equiv X_{{{\mathcal {H}}}_3}^\bot$ of the vector field $X \equiv X_{{{\mathcal {H}}}_3}$, which are linear and quadratic in *w*. Recall that such a reduction is needed since $\Pi _\gamma ^{(3)}$ (cf. (1.20)) allows for a loss of derivatives in space.

By Proposition 5.1, $X^\bot$ is of the form

$$\begin{aligned} X^\bot ({\mathfrak {x}}) = X_{{{\mathcal {H}}}_3}^\bot ({\mathfrak {x}}) {\mathop {=}\limits ^{(5.3)}} \mathrm{i} \Omega _\bot w + X_{{\mathcal {K}}}^\bot ({\mathfrak {x}}). \end{aligned}$$

Since $\Omega _\bot$ is a diagonal Fourier multiplier with constant real coefficients (cf. (1.18), (1.42)), it remains to normalize $X_{{\mathcal {K}}}^\bot ({\mathfrak {x}} )$ in the above sense.

By Proposition 5.1, $X_{{\mathcal {K}}}^\bot ({\mathfrak {x}} )$ admits an expansion of the form

6.1 $$\begin{aligned} \begin{aligned}&X_{{\mathcal {K}}}^\bot ({\mathfrak {x}}) = X^\bot _1(\theta , y)[w] + X^\bot _2(\theta )[w, w] + {{\mathcal {OB}}}^3(1, N) + {{\mathcal {OS}}}^3(1, N)\,, \\&X^\bot _1(\theta , y)[w] := \Upsilon ^\bot _1(\theta , y)[w] + {{\mathcal {R}}}^\bot _{N,1}(\theta , y)[w], \\&\quad \qquad X^\bot _2(\theta )[w, w] := \Upsilon ^\bot _2(\theta , w)[w] + {{\mathcal {R}}}^\bot _{N, 2}(\theta )[w, w]\,, \end{aligned} \end{aligned}$$

where

6.2 $$\begin{aligned} \begin{aligned}&\Upsilon ^\bot _1(\theta , y)[w] = \Pi _\bot \sum _{k = 0}^{N+1} T_{a_{1 - k}(\theta , y)} \partial _x^{1 - k} w \in {{\mathcal {OB}}}^2_w(1, N), \qquad {{\mathcal {R}}}^\bot _{N, 1}(\theta , y)[w] \in {{\mathcal {OS}}}^2_w(N)\,, \\&\Upsilon ^\bot _2(\theta , w)[w] = \Pi _\bot \sum _{k = 0}^{N+1} T_{A_{1 - k}(\theta )[w]} \partial _x^{1 - k} w \in {{\mathcal {OB}}}^2_{ww}(1, N), \quad {{\mathcal {R}}}^\bot _{N, 2}(\theta )[w, w] \in {{\mathcal {OS}}}^2_{ww}(N)\,. \end{aligned} \end{aligned}$$

By Definition 3.4, for any given $$N \in {\mathbb {N}}$$, there are integers $$s_N $$, $$\sigma _N > 0$$ (large) with the property that for any $$s \ge s_N$$ there exist $$0< \delta = \delta (s, \gamma , N) < 1$$ and $$0< \varepsilon _0 \equiv \varepsilon _0(s, \gamma , N) < 1$$ so that for any $$0 \le k \le N+1$$

6.3 $$\begin{aligned} \begin{aligned}&a_{1 - k} \in C^\infty _b \big ( {\mathbb {T}}^{S_+} \times B_{S_+}(\delta ) \times [0, \varepsilon _0] , \, H^s({\mathbb {T}}_1) \big ) \ \ \text {small of order one}, \\&A_{1 - k} \in C^\infty \big ({\mathbb {T}}^{S_+} \times [0, \varepsilon _0], \, {{\mathcal {B}}}(H^{s + \sigma _N}_\bot ({\mathbb {T}}_1), H^s({\mathbb {T}}_1)) \big )\,. \end{aligned} \end{aligned}$$

Note that $X^\bot _1(\theta , y)[w]$ is a vector field small of order 2 and linear in *w*, whereas $X^\bot _2(\theta )[w, w]$ is small of order 2, but quadratic in *w*. Since the vector field $X^\bot _{{{\mathcal {K}}}}$ is Hamiltonian, every term in the expansion (6.1), which is homogeneous in the coordinates *y*, *w*, is a Hamiltonian vector field as well. In particular, $X^\bot _1(\theta , y)[w]$ is such a vector field.

**Preliminary analysis of the vector field** $X^\bot _1(\theta , y)[w]$. Since $X^\bot _1(\theta , y)[w]$ is a Hamiltonian vector field which is linear in *w*, (A.2) in Appendix A implies that the diagonal operator

6.4 $$\begin{aligned} \mathrm{diag}_{j \in S^\bot } [X^\bot _1(\theta , y)]_j^j \end{aligned}$$

is skew-adjoint,

6.5 $$\begin{aligned} {[}X^\bot _1(\theta , y)]_j^j = - \overline{[X^\bot _1(\theta , y)]_j^j}, \qquad j \in S^\bot \,. \end{aligned}$$

We will show that the normal form transformations, constructed in this and the following subsection, preserve this property of $X^\bot _1(\theta , y)[w]$. Since this is the only property of the transformed vector field $X^\bot _1(\theta , y)[w]$ which is needed in the energy estimates in Sect. 7 we can allow for normal form transformations, which are not necessarily symplectic, as long as they preserve (6.5).

Our aim is to construct iteratively a coordinate transformation on ${{\mathcal {V}}}^s(\delta )$ so that when expressed in the new coordinates, the vector field $X^\bot _1(\theta , y)[w] + X^\bot _2(\theta )[w, w]$ is again of the form (6.2) and that the coefficients $a_{1 - k}(\theta , y) + A_{1 - k}(\theta )[w]$, $0 \le k \le N+1$, are independent of *x*. At the $(n+1)$th step, $n \ge 0$, we deal with a vector field $X_n = (X_n^{(\theta )}, X_n^{(y)}, X_n^\bot )$, defined as the pull back of *X* by the composition of the transformations up to the nth step, of the form

6.6 $$\begin{aligned} \begin{aligned} X_n^{(\theta )}({\mathfrak {x}})&= - \omega - \varepsilon {\widehat{\omega }} - \nabla _y Q(y) - \Upsilon _2^{(\theta )}(\theta )[w, w] + {{\mathcal {O}}}_3^{(\theta )}({\mathfrak {x}}) \,, \qquad X_n^{(y)}({\mathfrak {x}}) = {{\mathcal {O}}}_3^{(y)}({\mathfrak {x}})\,, \\ X_n^\bot ({\mathfrak {x}})&=\mathrm{i} \Omega _\bot w + {{\mathcal {D}}}^\bot _{n, 1}(\theta , y)[w] + {{\mathcal {D}}}^\bot _{n, 2}(\theta , w)[w] + X^\bot _{n, 1}(\theta , y)[w] + X^\bot _{n, 2}(\theta , w)[w] \\&\qquad + {{\mathcal {R}}}^{\bot }_{N, 1}(\theta , y)[w] + {{\mathcal {R}}}^{\bot }_{N, 2}(\theta , w)[w] + {{\mathcal {OB}}}^3(1, N) + {{\mathcal {OS}}}^3(N) \end{aligned}\nonumber \\ \end{aligned}$$

where for notational convenience, we write ${{\mathcal {R}}}^{\bot }_{N, j} \equiv {{\mathcal {R}}}^{\bot }_{n, N, j}$ for $j=1,2$, and where

6.7 $$\begin{aligned} \begin{aligned}&{{\mathcal {D}}}^\bot _{n, 1}(\theta , y)[w] \in {{\mathcal {OF}}}^2_{w}(1, N), \qquad \qquad {{\mathcal {D}}}^\bot _{n, 2}(\theta , w)[w] \in {{\mathcal {OF}}}^2_{ww}(1, N), \\&X^\bot _{n, 1}(\theta , y)[w] \in {{\mathcal {OB}}}_{w}^2(1 - n, N), \qquad X^\bot _{n, 2}(\theta , w)[w] \in {{\mathcal {OB}}}_{ww}^2(1 - n, N)\\&{{\mathcal {R}}}^{\bot }_{N,1}(\theta , y)[w] \in {{\mathcal {OS}}}_{w}^2(N), \qquad \qquad \ \ {{\mathcal {R}}}^\bot _{N, 2}(\theta , w)[w] \in {{\mathcal {OS}}}^2_{ww}(N)\,, \\&{{\mathcal {O}}}_3^{(\theta )}, {{\mathcal {O}}}_3^{(y)} \in C^\infty _b \big ( {{\mathcal {V}}}^{\sigma _N}(\delta ) \times [0, \varepsilon _0], \, {\mathbb {R}}^{S_+} \big ) \ \ \text {small of order three} \end{aligned} \end{aligned}$$

for some $\sigma _N > 0$. Moreover

6.8 $$\begin{aligned} \begin{aligned}&{{\mathcal {D}}}^\bot _{n, 1}(\theta , y) = - {{\mathcal {D}}}^\bot _{n, 1}(\theta , y)^\top , \quad [X^\bot _{n, 1}(\theta , y)]_j^j = - \overline{[X^\bot _{n, 1}(\theta , y)]_j^j}, \\&\quad \quad [{{\mathcal {R}}}^\bot _{N, 1}(\theta , y)]_j^j = - \overline{[{{\mathcal {R}}}^\bot _{N,1}(\theta , y)]_j^j}, \quad \forall j \in S^\bot . \end{aligned} \end{aligned}$$

Our goal at the (n+1)th step is to construct a transformation so that when expressed in the new coordinates, the vector field $$X^\bot _{n, 1}(\theta , y)[w] + X^\bot _{n, 2}(\theta , w)[w]$$ is of order $$1 -(n+1) = - n $$. Since $$X^\bot _{n, 1}(\theta , y)[w] \in {{\mathcal {OB}}}^2_{w}(1 - n, N)$$ and $$X^\bot _{n, 2}(\theta , w)[w] \in {{\mathcal {OB}}}_{ww}^2(1 - n, N)$$ we can write

6.9 $$\begin{aligned} \begin{aligned}&X^\bot _{n, 1} (\theta , y)[w] = \Pi _\bot T_{a_{1 - n}(\theta , y)} \partial _x^{1 - n} w + {{\mathcal {OB}}}^2_{w}(- n, N)\,, \\&X^\bot _{n, 1} (\theta , w)[w] = \Pi _\bot T_{A_{1 - n}(\theta )[w]} \partial _x^{1 - n} w + {{\mathcal {OB}}}^2_{ww}(- n, N) \end{aligned} \end{aligned}$$

with the property that there are integers $s_N > 0$, $\sigma _N \ge 0$ so that for any $s \ge s_N$ there exist $0< \delta \equiv \delta (s, \gamma , N) <1$ and $0< \varepsilon _0 \equiv \varepsilon _0(s, \gamma , N) < 1$ so that

6.10 $$\begin{aligned} \begin{aligned}&a_{1 - n} \in C^\infty _b \big ({\mathbb {T}}^{S_+} \times B_{S_+}(\delta ) \times [0, \varepsilon _0], \, H^s({\mathbb {T}}_1) \big ) \ \ \text {small of order one}, \\&A_{1 - n}\in C^\infty \big ({\mathbb {T}}^{S_+} \times [0, \varepsilon _0], \, {{\mathcal {B}}}(H^{s + \sigma _N}_\bot ({\mathbb {T}}_1), \, H^s({\mathbb {T}}_1)) \big )\,. \end{aligned} \end{aligned}$$

Hence we need to normalize the vector field $\Pi _\bot T_{a_{1 - n}(\theta , y) + A_{1 - n}(\theta )[w]} \partial _x^{1 - n} w$. In order to achieve this, we consider a para-differential vector field of the form

6.11 $$\begin{aligned} \begin{aligned}&Y^\bot _n (\theta , y, w) = Y^\bot _{n, 1}(\theta , y)[w] + Y^\bot _{n, 2}(\theta , w)[w]\,, \\&Y^\bot _{n, 1}(\theta ,y)[w] := \Pi _\bot T_{b_n(\theta , y)} \partial _x^{- n - 1} w \in {{\mathcal {OB}}}^2_{w}(- n - 1, N)\,, \\&Y^\bot _{n, 2}(\theta ,w)[w] := \Pi _\bot T_{B_n(\theta )[w]} \partial _x^{- n - 1} w \in {{\mathcal {OB}}}^2_{ww}(- n - 1, N)\,, \end{aligned} \end{aligned}$$

and make the ansatz that $b_n(\theta , y)$, $B_n(\theta )[w]$ are smooth functions (satisfying conditions as in (6.10)) and

$$\begin{aligned} \langle b_n(\theta , y)\rangle _x = 0, \qquad \langle B_n(\theta )[w]\rangle _x= 0\, . \end{aligned}$$

To determine $b_n$ and $B_n$, we compute the pullback $X_{n + 1} := \Phi _{Y_n}^* X_n$ of $X_n$ by the time one flow map $\Phi _{Y_n}$. corresponding to the vector field $Y_n$ . By Lemmata 3.7, 3.10, 3.13 and the induction hypothesis (6.8), one infers that the components of $X_{n + 1} = (X_{n + 1}^{(\theta )}, X_{n + 1}^{(y)}, X_{n + 1}^\bot )$ satisfy

6.12 $$\begin{aligned} \begin{aligned} X_{n + 1}^{(\theta )}({\mathfrak {x}})&= - \omega - \varepsilon {\widehat{\omega }} - \nabla _y Q(y) - \Upsilon _2^{(\theta )}(\theta )[w,w] + {{\mathcal {O}}}_3^{(\theta )}({\mathfrak {x}})\,, \qquad X_{n + 1}^{(y)}({\mathfrak {x}}) = {{\mathcal {O}}}_3^{(y)}({\mathfrak {x}})\,, \\ X_{n + 1}^\bot ({\mathfrak {x}})&=\mathrm{i} \Omega _\bot w + {{\mathcal {D}}}^\bot _{n, 1}(\theta , y)[w] + {{\mathcal {D}}}^\bot _{n, 2}(\theta , w)[w] + \Pi _\bot T_{- 3 \partial _x b_n(\theta , y) + a_{1 - n}(\theta , y)} \partial _x^{1 - n} w \\&\quad + \Pi _\bot T_{- 3 \partial _x B_n(\theta )[w]+ A_{1 - n}(\theta )[w]} \partial _x^{1 - n} w + X^\bot _{n + 1, 1}(\theta , y)[w] + {{\mathcal {R}}}^\bot _{N, 1}(\theta , y)[w] \\&\quad + X^\bot _{n + 1, 2}(\theta , w)[w] + {{\mathcal {R}}}^\bot _{N, 2}(\theta )[w, w] + {{\mathcal {OB}}}^3(1, N) + {{\mathcal {OS}}}^3(N) \end{aligned}\nonumber \\ \end{aligned}$$

where

6.13 $$\begin{aligned} \begin{aligned}&X^\bot _{n + 1, 1}(\theta , y)[w]\in {{\mathcal {OB}}}_{w}^2(- n, N), \qquad X^\bot _{n + 1, 2}(\theta , w)[w] \in {{\mathcal {OB}}}_{ww}^2( - n, N)\\&{{\mathcal {R}}}^\bot _{N, 1}(\theta , y)[w] \in {{\mathcal {OS}}}_{w}^2(N), \qquad \qquad \ \ {{\mathcal {R}}}^\bot _{N, 2}(\theta , w)[w] \in {{\mathcal {OS}}}^2_{ww}(N)\,, \\&{{\mathcal {O}}}_3^{(\theta )}, {{\mathcal {O}}}_3^{(y)} \in C^\infty _b \big ( {{\mathcal {V}}}^\sigma (\delta ) \times [0, \varepsilon _0], \ {\mathbb {R}}^{S_+} \big ) \quad \text {small of order three} \end{aligned} \end{aligned}$$

and the diagonal matrix elements of the operators $X^\bot _{n + 1, 1}(\theta , y)$, ${{\mathcal {R}}}^\bot _{N,1}(\theta , y)$ are purely imaginary, namely

6.14 $$\begin{aligned}{}[ X^\bot _{n + 1, 1}(\theta , y)]_j^j, \ [ {{\mathcal {R}}}^\bot _{N,1}(\theta , y)]_j^j \in \mathrm{i} {\mathbb {R}}\,, \quad \forall j \in S^\bot \,. \end{aligned}$$

We then choose $b_n(\theta , y)$ and $B_n(\theta )[w]$ to be solutions of

6.15 $$\begin{aligned} \begin{aligned}&- 3 \partial _x b_n(\theta , y) + a_{1 - n}(\theta , y) = \langle a_{1 - n}(\theta , y) \rangle _x\, , \\&- 3 \partial _x B_n(\theta )[w] + A_{1 - n}(\theta )[w] = \langle A_{1 - n}(\theta )[w] \rangle _x\, . \end{aligned} \end{aligned}$$

More precisely, we define

6.16 $$\begin{aligned} \begin{aligned}&b_n(\theta , y) := \frac{1}{3} \partial _x^{- 1}\big ( a_{1 - n}(\theta , y) - \langle a_{1 - n}(\theta , y) \rangle _x \big )\,, \\&B_n(\theta )[w] := \frac{1}{3} \partial _x^{- 1}\big ( A_{1 - n}(\theta )[w] - \langle A_{1 - n}(\theta )[w] \rangle _x \big ) \, . \end{aligned} \end{aligned}$$

Since $$\Pi _\bot T_{\langle a_{1 - n}(\theta , y) \rangle _x} \partial _x^{1 - n} w = \langle a_{1 - n}(\theta , y) \rangle _x \partial _x^{1 - n} w$$ and

$$\begin{aligned} \Pi _\bot T_{\langle A_{1 - n}(\theta )[w] \rangle _x} \partial _x^{1 - n} w = \langle A_{1 - n}(\theta )[w] \rangle _x \partial _x^{1 - n} w \end{aligned}$$

one infers from (6.10) that

6.17 $$\begin{aligned} \begin{aligned}&{{\mathcal {D}}}^\bot _{n + 1, 1}(\theta , y)[w] := {{\mathcal {D}}}^\bot _{n, 1}(\theta , y)[w] + \langle a_{1 - n}(\theta , y) \rangle _x \partial _x^{1 - n} w \in {{\mathcal {OF}}}^2_{w}(1, N), \\&{{\mathcal {D}}}^\bot _{n+ 1, 2}(\theta , w)[w] := {{\mathcal {D}}}^\bot _{n, 2}(\theta , w)[w] + \langle A_{1 - n}(\theta )[w] \rangle _x \partial _x^{1 - n} w \in {{\mathcal {OF}}}^2_{ww}(1, N)\,. \end{aligned}\quad \end{aligned}$$

Since $a_{1 - n }(\theta , y)$ is real valued, the Fourier multiplier $\langle a_{1 - n }(\theta , y) \rangle _x \partial ^{1 - n}$ is skew-adjoint if *n* is even. Futhermore, by the induction hypothesis (6.8) and Lemmata A.1, A.2 in Appendix A, one has

$$\begin{aligned} \begin{aligned} \langle a_{1 - n}(\theta , y) \rangle _x = 0 \quad \text {if} \ n \ \text {is odd.} \end{aligned} \end{aligned}$$

Hence the Fourier multiplier ${{\mathcal {D}}}^\bot _{n + 1, 1}(\theta , y)$ is skew-adjoint. Altogether we showed that the vector field $X_{n + 1}^\bot$ is of the form

6.18 $$\begin{aligned} X_{n + 1}^\bot ({\mathfrak {x}})= & {} \mathrm{i} \Omega _\bot w + {{\mathcal {D}}}^\bot _{n + 1, 1}(\theta , y)[w] + {{\mathcal {D}}}^\bot _{n + 1, 2}(\theta , w)[w] + X^\bot _{n + 1, 1}(\theta , y)[w] \nonumber \\&+ X^\bot _{n + 1, 2}(\theta , w)[w] + {{\mathcal {R}}}^\bot _{N, 1}(\theta , y)[w] + {{\mathcal {R}}}^\bot _{N, 2}(\theta , w)[w] + {{\mathcal {OB}}}^3(1, N) + {{\mathcal {OS}}}^3(N) \, .\nonumber \\ \end{aligned}$$

We thus have proved the following

#### Proposition 6.1

For any $N \in {\mathbb {N}}$, there exist $s_N$, $\sigma _N > 0$ with the following property: for any $s \ge s_N$ there exist $0< \delta \equiv \delta (s, \gamma , N) < 1$, $0< \varepsilon _0 \equiv \varepsilon _0(s, \gamma , N) < 1$ so that the following holds: there exists a transformation $\Psi ^{(1)}$ with inverse $(\Psi ^{(1)})^{-1}$ (cf. Remark 3.4),

6.19 $$\begin{aligned} (\Psi ^{(1)})^{\pm 1} \in {{\mathcal {C}}}^\infty _b({{\mathcal {V}}}^s(\delta ) \times [0, \varepsilon _0], \, {{\mathcal {V}}}^s(2 \delta )), \quad \forall \, s \ge s_N, \qquad (\Psi ^{(1)})^{\pm 1} ({\mathfrak {x}}) - {\mathfrak {x}} \ \ \text {small of order two}\, ,\nonumber \\ \end{aligned}$$

so that the transformed vector field $X_4 := (\Psi ^{(1)})^* X_{{{\mathcal {H}}}_3} = (X_4^{(\theta )}, X_4^{(y)}, X_4^\bot )$ has the following properties:

6.20 $$\begin{aligned} X_4^{(\theta )}({\mathfrak {x}})= & {} - \omega - \varepsilon {\widehat{\omega }} - \nabla _y Q(y) - \Upsilon _2^{(\theta )}(\theta )[w, w] + {{\mathcal {O}}}_3^{(\theta )}({\mathfrak {x}})\, , \qquad X_4^{(y)}({\mathfrak {x}}) = {{\mathcal {O}}}_3^{(y)}({\mathfrak {x}}) \, ,\nonumber \\ X^\bot _4({\mathfrak {x}})= & {} \mathrm{i} \Omega _\bot w + {{\mathcal {D}}}^\bot _{4, 1}(\theta , y)[w] + {{\mathcal {D}}}^\bot _{4, 2}(\theta , w)[w] + {{\mathcal {R}}}^\bot _{N, 1}(\theta , y)[w] + {{\mathcal {R}}}^\bot _{N, 2}(\theta , w)[w] \nonumber \\&+ {{\mathcal {OB}}}^3(1, N)+ {{\mathcal {OS}}}^3(N) \end{aligned}$$

where

6.21 $$\begin{aligned} \begin{aligned}&{{\mathcal {D}}}^\bot _{4, 1}(\theta , y)[w] \in {{\mathcal {OF}}}_{w}^2(1, N), \qquad {{\mathcal {D}}}^\bot _{4, 2}(\theta , w)[w] \in {{\mathcal {OF}}}_{ww}^2(1, N)\,, \\&{{\mathcal {R}}}^\bot _{N, 1}(\theta , y)[w] \in {{\mathcal {OS}}}_{w}^2(N), \qquad \quad {{\mathcal {R}}}^\bot _{N, 2}(\theta , w)[w] \in {{\mathcal {OS}}}_{ww}^2(N)\,, \\&{{\mathcal {O}}}_3^{(\theta )}, {{\mathcal {O}}}_3^{(y)} \in C^\infty _b \big ( {{\mathcal {V}}}^{\sigma _N}(\delta ) \times [0, \varepsilon _0], \, {\mathbb {R}}^{S_+} \big ) \quad \text {small of order three.} \end{aligned} \end{aligned}$$

Moreover

6.22 $$\begin{aligned} \quad {{\mathcal {D}}}^\bot _{4, 1}(\theta , y) = - ({{\mathcal {D}}}^\bot _{4, 1}(\theta , y))^\top , \qquad \quad [{{\mathcal {R}}}^\bot _{N, 1}(\theta , y)]_j^j \in \mathrm{i} {\mathbb {R}}, \quad \forall j \in S^\bot \,. \end{aligned}$$

### Normalization of Fourier multiplier quadratic in *w*

The goal of this subsection is to normalize the vector field ${{\mathcal {D}}}^\bot _{4, 2}(\theta , w)[w]$ in (6.20). According to Proposition 6.1 and Definitions (3.2), (3.4),

6.23 $$\begin{aligned} \begin{aligned}&{{\mathcal {D}}}^\bot _{4, 2}(\theta , w)[w] = \Lambda ^\bot _1(\theta )[w] \partial _x w + \widetilde{{\mathcal {D}}}^\bot _{4, 2}(\theta , w)[w], \\&\widetilde{{\mathcal {D}}}^\bot _{4, 2}(\theta , w)[w] := \sum _{k = 1}^{N+1} \Lambda ^\bot _{1 - k}(\theta )[w] \partial _x^{1 - k} w \in {{\mathcal {OF}}}^2_{ww}(0, N), \end{aligned}\nonumber \\ \end{aligned}$$

where for any $0 \le k \le N + 1$, $\Lambda ^\bot _{1 - k} \in C^\infty ({\mathbb {T}}^{S_+}, {{\mathcal {B}}}(H^{\sigma _N}_\bot , {\mathbb {R}}))$ for some $\sigma _N > 0$ (large). Since $\Lambda _1(\theta )[w]$ is real valued, the leading order operator $\Lambda _1(\theta )[w] \partial _x$ is a skew-adjoint Fourier multiplier and hence has the property needed for the energy estimates in Sect. 7. This however is not true for $\widetilde{{\mathcal {D}}}^\bot _{4, 2}(\theta , w)[w]$. The goal of this section is to eliminate it. To this end, we consider a vector field of the form

6.24 $$\begin{aligned} {{\mathcal {M}}} ({\mathfrak {x}}) := \big ( 0, 0, \, {{\mathcal {M}}}^\bot (\theta , w)[w]\big )\, , \quad {{\mathcal {M}}}^\bot (\theta , w)[w] = \sum _{k = 1}^{N+1} \Xi ^\bot _{1 - k}(\theta )[w] \partial _x^{1 - k}w \in {{\mathcal {OF}}}^2_{ww}(0, N),\nonumber \\ \end{aligned}$$

where $\Xi ^\bot _{1 - k}(\theta )$ will be chosen so that the time one flow map $\Phi _{{\mathcal {M}}}$, generated by the vector field $X_{{\mathcal {M}}}$, is a coordinate transformation serving our needs. In more detail, consider the pullback $X_5 := \Phi _{{\mathcal {M}}}^* X_4 = (X_5^{(\theta )}, X_5^{(y)}, X_5^\bot )$ of the vector field $X_4$ of Proposition 6.1 by $\Phi _{{\mathcal {M}}}$. By Lemmata 3.13, 3.15, one has

6.25 $$\begin{aligned} X_5^{(\theta )}({\mathfrak {x}})&= - \omega - \varepsilon {\widehat{\omega }} - \nabla _y Q(y) - \Upsilon ^{(\theta )}_2(\theta )[w, w] + {{\mathcal {O}}}_3^{(\theta )}({\mathfrak {x}}) \, , \qquad X_5^{(y)} = {{\mathcal {O}}}_3^{(y)}({\mathfrak {x}}) \, ,\nonumber \\ X_5^\bot ({\mathfrak {x}})&=\mathrm{i} \Omega _\bot w + {{\mathcal {D}}}_{4,1}^\bot (\theta , y)[w] +\Lambda _1(\theta )[w] \partial _x w + {{\mathcal {R}}}^\bot _{4, 1}(\theta , y)[w] + {{\mathcal {R}}}^\bot _{4,2}(\theta , w)[w] \nonumber \\&\qquad + \omega \cdot \partial _\theta {{\mathcal {M}}}^\bot (\theta , w)[w] - {{\mathcal {M}}}^\bot (\theta ,\mathrm{i} \Omega _\bot w) [w]+ \widetilde{{\mathcal {D}}}_{4, 2}(\theta , w)[w] \nonumber \\&\quad + {{\mathcal {OB}}}^3(1, N) + {{\mathcal {OS}}}^3(N) \end{aligned}$$

where for some integer $\sigma _N > 0$, ${{\mathcal {O}}}_3^{(\theta )}$, ${{\mathcal {O}}}_3^{(y)}$ are in $C^\infty _b \big ({{\mathcal {V}}}^{\sigma _N}(\delta ) \times [0,\varepsilon _0], \, {\mathbb {R}}^{S_+}\big )$ and small of order three. The vector field ${{\mathcal {M}}}^\bot (\theta , w)[w]$ is chosen to be a solution the following homological equation

6.26 $$\begin{aligned} \omega \cdot \partial _\theta {{\mathcal {M}}}^\bot (\theta , w)[w] - {{\mathcal {M}}}^\bot (\theta , \mathrm{i} \Omega _\bot w) [w]+ \widetilde{{\mathcal {D}}}^\bot _{4, 2}(\theta , w)[w] = 0 , \end{aligned}$$

or in view of (6.23), (6.24) equivalently, that for any $1 \le k \le N + 1$, $\Xi ^\bot _{1 - k}(\theta )[w]$ is a solution of

6.27 $$\begin{aligned} \omega \cdot \partial _\theta \, \Xi ^\bot _{1 - k}(\theta )[w] - \Xi ^\bot _{1 - k}(\theta )[ \mathrm{i} \Omega _\bot w] + \Lambda ^\bot _{1 - k}(\theta )[w] = 0\,. \end{aligned}$$

Since $\Lambda ^\bot _{1 - k}$, $\Xi ^\bot _{1 - k} \in C^\infty ({\mathbb {T}}^{S_+} \times [0, \varepsilon _0], \, {{\mathcal {B}}}(H^{\sigma _N}_\bot ({\mathbb {T}}_1), {\mathbb {R}}))$, there exist uniquely determined maps $a_{\Lambda ^\bot _{1 - k}}$, $a_{\Xi ^\bot _{1 - k}}$ in $C^\infty ({\mathbb {T}}^{S_+} \times [0, \varepsilon _0], \, H^{- \sigma _N}_\bot ({\mathbb {T}}_1))$ so that

$$\begin{aligned} \Lambda ^\bot _{1 - k}(\theta )[w] = \big \langle a_{\Lambda ^\bot _{1 - k}}(\theta )\,,\, w \big \rangle \,, \qquad \Xi ^\bot _{1 - k}(\theta )[w] = \big \langle a_{\Xi ^\bot _{1 - k}}(\theta )\,,\, w \big \rangle \, . \end{aligned}$$

Equation (6.27) then reads

6.28 $$\begin{aligned} \big \langle \omega \cdot \partial _\theta \, a_{\Xi ^\bot _{1 - k}}(\theta ), \, w \big \rangle - \big \langle a_{\Xi ^\bot _{1 - k}}(\theta ), \, \mathrm{i} \Omega _\bot w \big \rangle + \big \langle a_{\Lambda ^\bot _{1 - k}}(\theta ), \, w \big \rangle = 0\,. \end{aligned}$$

Since $\mathrm{i} \Omega _\bot$ is skew-adjoint, one has $- \big \langle a_{\Xi ^\bot _{1 - k}}(\theta ), \, \mathrm{i} \Omega _\bot w \big \rangle = \big \langle \mathrm{i} \Omega _\bot a_{\Xi ^\bot _{1 - k}}(\theta ), w \big \rangle$. We choose $a_{\Xi ^\bot _{1 - k}}$ as the solution of

6.29 $$\begin{aligned} \big ( \omega \cdot \partial _\theta + \mathrm{i} \Omega _\bot \big )a_{\Xi ^\bot _{1 - k}}(\theta ) + a_{\Lambda ^\bot _{1 - k}}(\theta ) = 0\,. \end{aligned}$$

This equation can be solved by expanding $a_{\Xi ^\bot _{1 - k}}(\theta )$ and $a_{\Lambda ^\bot _{1 - k}}(\theta )$ in Fourier series with respect to $\theta$ and *x*,

$$\begin{aligned}&a_{\Xi ^\bot _{1 - k}}(\theta ) = \sum _{(\ell , j) \in {\mathbb {Z}}^{S_+} \times S^\bot } {\widehat{a}}_{\Xi ^\bot _{1 - k}}(\ell , j) e^{\mathrm{i} \ell \cdot \theta } e^{\mathrm{i} 2 \pi j x}, \\&\quad \qquad a_{\Lambda ^\bot _{1 - k}}(\theta ) = \sum _{(\ell , j) \in {\mathbb {Z}}^{S_+} \times S^\bot } {\widehat{a}}_{\Lambda ^\bot _{1 - k}}(\ell , j) e^{\mathrm{i} \ell \cdot \theta } e^{\mathrm{i} 2 \pi j x}. \end{aligned}$$

Since by assumption $\omega \in \Pi ^{(1)}_\gamma$, $0< \gamma < 1$, (cf. (1.20)), Eq. (6.29) can be solved. The solution $a_{\Xi ^\bot _{1 - k}} (\theta )$ is given by

6.30 $$\begin{aligned} a_{\Xi ^\bot _{1 - k}} = - (\omega \cdot \partial _\theta + \mathrm{i} \Omega _\bot )^{- 1} a_{\Lambda ^\bot _{1 - k}} = - \sum _{(\ell , j) \in {\mathbb {Z}}^{S_+} \times S^\bot }\dfrac{{\widehat{a}}_{\Lambda ^\bot _{1 - k}}(\ell , j)}{\mathrm{i} (\omega \cdot \ell + \Omega _j)} e^{\mathrm{i} \ell \cdot \theta } e^{\mathrm{i} 2 \pi j x}\,.\nonumber \\ \end{aligned}$$

Since $a_{\Lambda ^\bot _{1 - k}} \in C^\infty ({\mathbb {T}}^{S_+}, H^{- \sigma _N}_\bot ({\mathbb {T}}_1))$, one infers that $a_{\Xi ^\bot _{1 - k}} \in C^\infty ({\mathbb {T}}^{S_+}, H^{- \sigma _N - \tau }_\bot ({\mathbb {T}}_1))$ and therefore (6.24) is verified and Eq. (6.26) is solved. Finally, the vector field $X_5^\bot$ is of the form

6.31 $$\begin{aligned}&X_5^\bot ({\mathfrak {x}}) = \mathrm{i} \Omega _\bot w + {{\mathcal {D}}}_5({\mathfrak {x}})[w] + {{\mathcal {R}}}^\bot _{N, 1}(\theta , y)[w] + {{\mathcal {R}}}^\bot _{N, 2}(\theta , w)[w]\nonumber \\&\quad \quad \quad \quad + {{\mathcal {OB}}}^3(1, N) + {{\mathcal {OS}}}^3(N)\,, \nonumber \\&{{\mathcal {D}}}^\bot _5({\mathfrak {x}})[w] := {{\mathcal {D}}}^\bot _{4, 1}(\theta , y)[w] +\Lambda ^\bot _1(\theta )[w] \partial _x w \in {{\mathcal {OF}}}^2(1, N), \end{aligned}$$

where the remainders ${{\mathcal {R}}}^\bot _{N, 1}(\theta , y)[w]$, ${{\mathcal {R}}}^\bot _{N, 2}(\theta , w)[w]$ are given in Proposition 6.1. Furthermore, ${{\mathcal {D}}}^\bot _5({\mathfrak {x}})$ is skew-adjoint,

6.32 $$\begin{aligned} {{\mathcal {D}}}^\bot _5({\mathfrak {x}})^\top = - {{\mathcal {D}}}^\bot _5({\mathfrak {x}})\,. \end{aligned}$$

We summarize our findings of this subsection as follows.

#### Proposition 6.2

For any $N \in {\mathbb {N}}$, there exists an integer $s_N > 0$ with the property that for any $s \ge s_N$ there exist $0< \delta \equiv \delta (s, \gamma , N) < 1$ and $0< \varepsilon _0 \equiv \varepsilon _0(s, \gamma , N) < 1$ so that the following holds: there exists a transformation $\Psi ^{(2)}$ with inverse $(\Psi ^{(2)})^{-1}$ (cf. Remark 3.4),

6.33 $$\begin{aligned} (\Psi ^{(2)})^{\pm 1} \in {{\mathcal {C}}}^\infty _b({{\mathcal {V}}}^s(\delta ) \times [0, \varepsilon _0], \, {{\mathcal {V}}}^s(2 \delta )), \quad \forall \, s \ge s_N, \qquad (\Psi ^{(2)})^{\pm 1}({\mathfrak {x}}) - {\mathfrak {x}} \quad \text {small of order two},\nonumber \\ \end{aligned}$$

so that the transformed vector field $X_5 := (\Psi ^{(2)})^* X_{4} = (X_5^{(\theta )}, X_5^{(y)}, X_5^\bot )$ has the form

6.34 $$\begin{aligned} X_5^{(\theta )}({\mathfrak {x}})= & {} - \omega - \varepsilon {\widehat{\omega }} - \nabla _y Q(y) - \Upsilon ^{(\theta )}_2(\theta )[w, w] + {{\mathcal {O}}}_3({\mathfrak {x}}) \, , \qquad X_5^{(y)}({\mathfrak {x}}) = {{\mathcal {O}}}_3({\mathfrak {x}}) \, , \nonumber \\ X^\bot _5({\mathfrak {x}})= & {} \mathrm{i} \Omega _\bot w + {{\mathcal {D}}}^\bot _5({\mathfrak {x}})[w] + {{\mathcal {R}}}^\bot _{N, 1}(\theta , y)[w] + {{\mathcal {R}}}^\bot _{N, 2}(\theta , w)[w] + {{\mathcal {OB}}}^3(1, N) + {{\mathcal {OS}}}^3(N)\nonumber \\ \end{aligned}$$

where

6.35 $$\begin{aligned} \begin{aligned}&{{\mathcal {D}}}^\bot _5({\mathfrak {x}})[w] \in {{\mathcal {OF}}}^2(1, N), \qquad {{\mathcal {D}}}^\bot _5({\mathfrak {x}}) = - {{\mathcal {D}}}^\bot _5({\mathfrak {x}})^\top \, \end{aligned} \end{aligned}$$

and the smoothing remainders ${{\mathcal {R}}}^\bot _{N,1}(\theta , y)[w]$, ${{\mathcal {R}}}^\bot _{N, 2}(\theta , w)[w]$ are given by Proposition 6.1.

### Normalization of the smoothing remainders

In this subsection, we normalize the vector field

$$\begin{aligned} \big ( \Upsilon ^{(\theta )}_2(\theta )[w, w], \, 0, \, {{\mathcal {R}}}^\bot _{N, 1}(\theta , y)[w] + {{\mathcal {R}}}^\bot _{N, 2}(\theta )[w, w] \big ) \, , \end{aligned}$$

which is part of the vector field $X_5$ defined in (6.34). Note that all the terms are either linear or quadratic in the variable *w*. We consider a smoothing vector field of the form

$$\begin{aligned} {{\mathcal {S}}}({\mathfrak {x}}) := \big ( \, {\mathcal {S}}^{(\theta )}(\theta )[w, w], \ 0, \ {{\mathcal {S}}}^\bot _1(\theta , y)[w] + {{\mathcal {S}}}^\bot _2(\theta )[w, w] \big ) \end{aligned}$$

where we make the ansatz that for some $\sigma _N > 0$, ${\mathcal {S}}^{(\theta )} \in C^\infty \big ( {\mathbb {T}}^{S_+} \times [0, \varepsilon _0], \, {{\mathcal {B}}}_2(H^{\sigma _N}_\bot ({\mathbb {T}}_1), {\mathbb {R}}^{S_+}) \big )$ and

6.36 $$\begin{aligned} {{\mathcal {S}}}^\bot _1(\theta , y)[w] \in {{\mathcal {OS}}}_{w}^2(N - 1), \qquad {{\mathcal {S}}}^\bot _2(\theta )[w, w] \in {{\mathcal {OS}}}_{ww}^2(N - 5)\,. \end{aligned}$$

We then consider the time one flow map $\Phi _{{\mathcal {S}}}$, associated to the vector field ${{{\mathcal {S}}}}$, and compute the pullback $X_6 := \Phi _{{\mathcal {S}}}^* X_5 = (X_6^{(\theta )}, X_6^{(y)}, X_6^\bot )$ of the vector field $X_5$ by $\Phi _{\mathcal {S}}$. By Lemmata 3.17, 3.18 and in view of Remark (3.3), $X_6$ is of the form

6.37 $$\begin{aligned} \begin{aligned}&X_6^{(\theta )}({\mathfrak {x}}) = - \omega - \varepsilon {\widehat{\omega }} - \nabla _y Q(y) + \omega \cdot \partial _\theta \, {\mathcal {S}}^{(\theta )}(\theta )[w, w] - {\mathcal {S}}^{(\theta )}(\theta )[\mathrm{i} \Omega _\bot w, w]\\&\qquad \qquad \quad - {\mathcal {S}}^{(\theta )}(\theta )[w, \mathrm{i} \Omega _\bot w] - \Upsilon ^{(\theta )}_2(\theta )[w, w] + {{\mathcal {O}}}^{(\theta )}_3({\mathfrak {x}}) , \\&X_6^{(y)}({\mathfrak {x}}) = {{\mathcal {O}}}^{(y)}_3({\mathfrak {x}}) , \\&X_6^\bot ({\mathfrak {x}}) =\mathrm{i} \Omega _\bot w + {{\mathcal {D}}}^\bot _5({\mathfrak {x}})[w] + \Big ( \omega \cdot \partial _\theta {{\mathcal {S}}}^\bot _1(\theta , y) + [\mathrm{i} \Omega _\bot , \, {{\mathcal {S}}}^\bot _1(\theta , y)]_{lin} + {{\mathcal {R}}}^\bot _{N, 1}(\theta , y) \Big )[w] \\&\quad \qquad + \omega \cdot \partial _\theta \, {{\mathcal {S}}}^\bot _2(\theta )[w, w] + \mathrm{i} \Omega _\bot {{\mathcal {S}}}^\bot _2(\theta )[w, w] - {{\mathcal {S}}}^\bot _2(\theta )[\mathrm{i} \Omega _\bot w, w] \\&\quad \qquad - {{\mathcal {S}}}^\bot _2(\theta )[w, \mathrm{i} \Omega _\bot w] + {{\mathcal {R}}}^\bot _{N, 2}(\theta )[ w, w] + {{\mathcal {OB}}}^3(1, N ) + {{\mathcal {OS}}}^3(N - 6) \end{aligned}\nonumber \\ \end{aligned}$$

where ${{\mathcal {O}}}^{(\theta )}_3$, ${{\mathcal {O}}}^{(y)}_3$ denote terms which are small of order three. The components ${\mathcal {S}}^{(\theta )}$ and ${\mathcal {S}}^\bot _1$, ${\mathcal {S}}^\bot _2$ are now chosen as the solutions of the following homological equations,

6.38 $$\begin{aligned} \begin{aligned}&\omega \cdot \partial _\theta \, {\mathcal {S}}^{(\theta )}(\theta )[w , w] - {\mathcal {S}}^{(\theta )}(\theta )[\mathrm{i} \Omega _\bot w, w] - {\mathcal {S}}^{(\theta )}(\theta )[w, \mathrm{i} \Omega _\bot w] \\&\quad - \Upsilon ^{(\theta )}_2(\theta )[w, w] = - {\mathcal {Z}}^{(\theta )}[w, w], \\&\qquad \quad {\mathcal {Z}}^{(\theta )} [w, w] := \sum _{j \in S^\bot } \, w_j w_{- j} \, \langle \Upsilon _2^{(\theta )}(\theta )[e^{\mathrm{i} 2 \pi j x}, e^{- \mathrm{i} 2 \pi j x}] \rangle _\theta \, , \\&\omega \cdot \partial _\theta \, {{\mathcal {S}}}^\bot _1(\theta , y) + [\mathrm{i} \Omega _\bot , {{\mathcal {S}}}^\bot _1(\theta , y)]_{lin} + {{\mathcal {R}}}^\bot _{N, 1}(\theta , y) = {{\mathcal {Z}}}^\bot ( y)\,, \\&\qquad {{\mathcal {Z}}}^\bot ( y) := \mathrm{diag}_{j \in S^\bot } [\widehat{{\mathcal {R}}}^\bot _{N, 1}(0, y)]_j^j\,, \\&\omega \cdot \partial _\theta \, {{\mathcal {S}}}^\bot _2(\theta )[w, w] + \mathrm{i} \Omega _\bot {{\mathcal {S}}}^\bot _2(\theta )[w, w] - {{\mathcal {S}}}^\bot _2(\theta )[\mathrm{i} \Omega _\bot w, w] \\&\quad - {{\mathcal {S}}}^\bot _2(\theta )[w,\mathrm{i} \Omega _\bot w] + {{\mathcal {R}}}^\bot _{N, 2}(\theta )[ w, w]= 0\,. \end{aligned} \end{aligned}$$

Homological equations of this form can be solved by applying the following lemma.

#### Lemma 6.1

Let $N \in {\mathbb {N}}$. (*i*) Let ${\mathcal {M}}^{(\theta )} \in C^\infty ({\mathbb {T}}^{S_+} \times [0, \varepsilon _0], \, {{\mathcal {B}}}_2(H^\sigma _\bot ({\mathbb {T}}_1), \, {\mathbb {R}}^{S_+}))$ for some $\sigma > 0$ and assume that $\omega \in \Pi _\gamma$, $0< \gamma < 1$ (cf. (1.20)). Then there exists ${\mathcal {S}}^{(\theta )} \in C^\infty ({\mathbb {T}}^{S_+} \times [0, \varepsilon _0], \, {{\mathcal {B}}}_2(H^{\sigma + 1}_\bot (T_1), \, {\mathbb {R}}^{S_+}))$ solving

6.39 $$\begin{aligned} \begin{aligned}&\omega \cdot \partial _\theta \, {\mathcal {S}}^{(\theta )}(\theta )[w , w] - {\mathcal {S}}^{(\theta )}(\theta )[\mathrm{i} \Omega _\bot w, w] - {\mathcal {S}}^{(\theta )}(\theta )[w, \mathrm{i} \Omega _\bot w] \\&\quad - {\mathcal {M}}^{(\theta )}(\theta )[w, w] = - {\mathcal {Z}}^{(\theta )}[w, w], \\&{\mathcal {Z}}^{(\theta )}[w, w] := \sum _{j \in S^\bot } \, w_j w_{- j} \, \langle {\mathcal {M}}^{(\theta )}(\theta )[e^{\mathrm{i} 2 \pi j x}, e^{- \mathrm{i} 2 \pi j x}] \rangle _\theta \,. \end{aligned} \end{aligned}$$

(*ii*) Let ${{\mathcal {R}}}^\bot _{N, 1}(\theta , y)[w] \in {{\mathcal {OS}}}_{w}^2(N)$ and $\omega \in \Pi _\gamma$, $0< \gamma < 1$ (cf. (1.20)). Then there exists ${{\mathcal {S}}}^\bot _1(\theta , y)[w] \in {{\mathcal {OS}}}_{w}^2(N - 1)$ which solves the equation

6.40 $$\begin{aligned}&\omega \cdot \partial _\theta \, {{\mathcal {S}}}^\bot _1(\theta , y) + [\mathrm{i} \Omega _\bot , \, {{\mathcal {S}}}^\bot _1(\theta , y)]_{lin} + {{\mathcal {R}}}^\bot _{N, 1}(\theta , y) = {{\mathcal {Z}}}^\bot ( y)\,, \nonumber \\&\quad \qquad {{\mathcal {Z}}}^\bot ( y) := \mathrm{diag}_{j \in S^\bot } [\widehat{{\mathcal {R}}}^\bot _{N, 1}(0, y)]_j^j\,. \end{aligned}$$

(*iii*) Let ${{\mathcal {R}}}^\bot _{N, 2}(\theta )[w, w] \in {{\mathcal {OS}}}_{ww}^2(N)$ and assume that $\omega \in \Pi _\gamma$, $0< \gamma < 1$ (cf. (1.20)). Then there exists ${{\mathcal {S}}}^\bot _2(\theta )[w, w] \in {{\mathcal {OS}}}_{ww}^2(N - 5)$ which solves the equation

6.41 $$\begin{aligned} \begin{aligned}&\omega \cdot \partial _\theta \, {{\mathcal {S}}}^\bot _2(\theta )[w, w] +\mathrm{i} \Omega _\bot {{\mathcal {S}}}^\bot _2(\theta )[w, w] - {{\mathcal {S}}}^\bot _2(\theta )[\mathrm{i} \Omega _\bot w, w] \\&\quad - {{\mathcal {S}}}^\bot _2(\theta )[w, \mathrm{i} \Omega _\bot w]+ {{\mathcal {R}}}^\bot _{N, 2}(\theta )[w, w] = 0\,. \end{aligned} \end{aligned}$$

#### Proof

Since items (*i*), (*ii*) can be proved by arguments similar to the ones used in the proof of item (*iii*), we only prove the latter. By assumption, ${\mathcal {R}}^\bot _{N, 2}(\theta , w)[w] \equiv {{\mathcal {R}}^\bot _{N, 2}}(\theta )[w, w] \in {{\mathcal {OS}}}_{ww}^2(N)$. Hence there exists an integer $s_N> 0$ with the property that for any $s \ge s_N$, there exists $0< \varepsilon _0 \equiv \varepsilon _0(s) < 1$ so that

$$\begin{aligned}&{\mathcal {R}}^\bot _{N, 2} : {\mathbb {T}}^{S_+} \times [0, \varepsilon _0] \rightarrow {{\mathcal {B}}}_{2, s, N} , \, (\theta , \varepsilon ) \mapsto {\mathcal {R}}^\bot _{N, 2}(\theta ) \equiv {\mathcal {R}}^\bot _{N, 2}(\theta , \varepsilon ) \, , \\&\quad \qquad {{\mathcal {B}}}_{2, s, N}: = {\mathcal {B}}_2(H^{s}({\mathbb {T}}_1), H^{s + N + 1}({\mathbb {T}}_1)), \end{aligned}$$

is $C^\infty$-smooth and bounded (cf. (1.39), Defintion 3.4). A a consequence, for any multi-index $\alpha \in {\mathbb {Z}}_{\ge 0}^{S_+}$,

6.42 $$\begin{aligned} \Vert \partial _\theta ^\alpha {\mathcal {R}}^\bot _{N, 2}(\theta ) \Vert _{{{\mathcal {B}}}_{2, s, N}} \lesssim _{\alpha , s} 1\,. \end{aligned}$$

Expanding ${\mathcal {R}}^\bot _{N, 2}(\theta )$ in its Fourier series, ${\mathcal {R}}^\bot _{N, 2}(\theta ) = \sum _{\ell \in {\mathbb {Z}}^{S_+}} \widehat{{\mathcal {R}}^\bot _{N, 2}}(\ell ) e^{\mathrm{i} \ell \cdot \theta }$, the latter estimates imply

6.43 $$\begin{aligned} \Vert \widehat{{\mathcal {R}}^\bot _{N, 2}}(\ell ) \Vert _{{{\mathcal {B}}}_{2, s, N}} \lesssim _{\alpha , s} \langle \ell \rangle ^{- |\alpha |}, \qquad \forall \, \alpha \in {\mathbb {Z}}_{\ge 0}^{S_+}\,, \ \ \forall \, \ell \in {\mathbb {Z}}^{S_+}\,. \end{aligned}$$

Since for any $\ell \in {\mathbb {Z}}^{S_+}$, $\widehat{{\mathcal {R}}^\bot _{N, 2}}(\ell ) \in {{\mathcal {B}}}_{2, s, N}$, one has for any $w, v \in H^s_\bot ({\mathbb {T}}_1)$

6.44 $$\begin{aligned} \widehat{{\mathcal {R}}^\bot _{N, 2}}(\ell )[w, v] = \sum _{j, j' \in S^\bot } w_j v_{j'} \widehat{{\mathcal {R}}^\bot _{N, 2}}(\ell )_{j j'} \,, \qquad \widehat{{\mathcal {R}}^\bot _{N, 2}}(\ell )_{j j'}( x) := \widehat{{\mathcal {R}}^\bot _{N, 2}}(\ell )[e^{\mathrm{i} 2 \pi j x}, e^{\mathrm{i} 2 \pi j' x}]\,.\nonumber \\ \end{aligned}$$

In particular, for $w = e^{\mathrm{i} 2 \pi j x}$, $v = e^{\mathrm{i} 2 \pi j' x}$, one infers from (6.43) that

6.45 $$\begin{aligned} \Vert \widehat{{\mathcal {R}}^\bot _{N, 2}}(\ell )_{j j'} \Vert _{s + N + 1} \lesssim _{\alpha , s} \langle \ell \rangle ^{- |\alpha |}\langle j \rangle ^s \langle j' \rangle ^s\,, \qquad \forall \, \alpha \in {\mathbb {Z}}_{\ge 0}^{S_+}, \ \ell \in {\mathbb {Z}}^{S_+}, \ j , j' \in S^\bot .\nonumber \\ \end{aligned}$$

Expanding also ${\mathcal {S}}^\bot _2(\theta )$ in its Fourier series, ${\mathcal {S}}^\bot _2(\theta ) = \sum _{\ell \in {\mathbb {Z}}^{S_+}} \widehat{{\mathcal {S}}^\bot _2}(\ell ) e^{\mathrm{i} \ell \cdot \theta }$, one has for any $w, v \in H^s_\bot ({\mathbb {T}}_1)$,

6.46 $$\begin{aligned} \widehat{{\mathcal {S}}^\bot _2}(\ell )[w, v] = \sum _{j, j' \in S^\bot } w_j v_{j'} \widehat{{\mathcal {S}}^\bot _2}(\ell )_{j j'} \,, \qquad \widehat{{\mathcal {S}}^\bot _2}(\ell )_{j j'}( x) := \widehat{{\mathcal {S}}^\bot _2}(\ell )[e^{\mathrm{i} 2 \pi j x}, e^{\mathrm{i} 2 \pi j' x}]\,.\nonumber \\ \end{aligned}$$

By expanding $\widehat{{\mathcal {S}}^\bot _2}(\ell )_{j j'}( x)$ and $\widehat{{\mathcal {R}}^\bot _2}(\ell )_{j j'}( x)$ with respect to the variable $x \in {\mathbb {T}}_1$ in Fourier series,

6.47 $$\begin{aligned} \widehat{{\mathcal {S}}^\bot _2}(\ell )_{j j'}( x) = \sum _{n \in S^\bot } \widehat{{\mathcal {S}}^\bot _2}(\ell , n)_{j j'} e^{\mathrm{i} 2 \pi n x}, \quad \widehat{{\mathcal {R}}^\bot _{N, 2}}(\ell )_{j j'}( x) = \sum _{n \in S^\bot } \widehat{{\mathcal {R}}^\bot _{N, 2}}(\ell , n)_{j j'} e^{\mathrm{i} 2 \pi n x}\,,\nonumber \\ \end{aligned}$$

the homological equation (6.41) yields the following equations for the coefficients $\widehat{{\mathcal {S}}^\bot _2}(\ell , n)_{j j'}$ of $\widehat{{\mathcal {S}}^\bot _2}(\ell )$,

6.48 $$\begin{aligned} \mathrm{i} \big ( \omega \cdot \ell + \Omega _n - \Omega _j - \Omega _{j'}\big ) \widehat{{\mathcal {S}}^\bot _2}(\ell , n)_{j j'} + \widehat{{\mathcal {R}}^\bot _{N, 2}}(\ell , n)_{j j'} = 0\,. \end{aligned}$$

Since $\omega \in \Pi _\gamma ^{(3)}$, $0< \gamma < 1$ (cf. (1.20)), the latter equations admit solutions. They are given by

6.49 $$\begin{aligned} \widehat{{\mathcal {S}}^\bot _2}(\ell , n)_{j j'} = - \dfrac{\widehat{{\mathcal {R}}^\bot _{N, 2}}(\ell , n)_{j j'}}{\mathrm{i} \big (\omega \cdot \ell + \Omega _n - \Omega _j - \Omega _{j'}\big )}\, , \qquad \forall \, \ell \in {\mathbb {Z}}^{S_+}, \ n, \, j, \, j' \in S^\bot \, ,\nonumber \\ \end{aligned}$$

and satisfy the estimate $|\widehat{{\mathcal {S}}^\bot _2}(\ell , n)_{j j'}| \le \langle \ell \rangle ^\tau \langle j \rangle ^2 \langle j' \rangle ^2 \langle n \rangle ^2 \gamma ^{- 1} |\widehat{{\mathcal {R}}^\bot _{N, 2}}(\ell , n)_{j j'}|$ (cf. (1.20)). By (6.47), one has $\Vert \widehat{{\mathcal {S}}^\bot _2}(\ell )_{j j'} \Vert _{s + N - 1} = \big ( \sum _{n \in S^\bot } \langle n \rangle ^{2 (s + N - 1)} |\widehat{{\mathcal {S}}^\bot _2}(\ell , n)_{j j'}|^2 \big )^{\frac{1}{2}}$ and hence

6.50 $$\begin{aligned} \begin{aligned} \Vert \widehat{{\mathcal {S}}^\bot _2}(\ell )_{j j'} \Vert _{s + N - 1}&\le \langle \ell \rangle ^\tau \langle j \rangle ^2 \langle j' \rangle ^2 \gamma ^{- 1} \Big ( \sum _{n \in S^\bot } \langle n \rangle ^{2 (s + N - 1)} \langle n \rangle ^4 | \widehat{{\mathcal {R}}^\bot _{N, 2}} (\ell , n)_{j j'}|^2 \Big )^{\frac{1}{2}} \\&= \langle \ell \rangle ^\tau \langle j \rangle ^2 \langle j' \rangle ^2 \gamma ^{- 1} \Vert \widehat{{\mathcal {R}}^\bot _{N, 2}}(\ell )_{j j'} \Vert _{s + N + 1} {\mathop {\lesssim _{\alpha , s}}\limits ^{(6.45)}} \langle \ell \rangle ^{\tau - |\alpha |} \langle j \rangle ^{s + 2} \langle j' \rangle ^{s + 2} \gamma ^{- 1}\,. \end{aligned} \end{aligned}$$

For any $w, v \in H^{s+3}_\bot ({\mathbb {T}}_1)$, one then obtains by the Cauchy–Schwarz inequality,

6.51 $$\begin{aligned} \begin{aligned} \Vert \widehat{{\mathcal {S}}^\bot _2}(\ell )[w, v] \Vert _{s + N - 1}&{\le } \sum _{j, j' \in S^\bot } |w_j | |v_{j'}| \,\Vert \widehat{{\mathcal {S}}^\bot _2}(\ell )_{j j'} \Vert _{s + N - 1}\\&\quad {\mathop {\lesssim _{\alpha , s}}\limits ^{(6.50)}} \langle \ell \rangle ^{\tau - |\alpha |} \gamma ^{- 1} \sum _{j, j' \in S^\bot } \langle j \rangle ^{s + 2} |w_j| \langle j' \rangle ^{s + 2} |v_{j'}| \\&\lesssim _{\alpha , s} \langle \ell \rangle ^{\tau - |\alpha |} \gamma ^{- 1} \Vert w \Vert _{s + 3} \Vert v \Vert _{s + 3} \, . \end{aligned} \end{aligned}$$

Writing *s* for $s + 3$, we thus have proved that there exists $s_N > 0$ (large) so that

$$\begin{aligned} \Vert \widehat{{\mathcal {S}}^\bot _2}(\ell ) \Vert _{{{\mathcal {B}}}_{2, s, N - 4}} \lesssim _{\alpha , s} \langle \ell \rangle ^{\tau - |\alpha |}\gamma ^{- 1}\, , \qquad \forall \, \alpha \in {\mathbb {Z}}_{\ge 0}^{S_+}, \ s \ge s_N\, . \end{aligned}$$

implying that ${{\mathcal {S}}^\bot _2} \in C^\infty ({\mathbb {T}}^{S_+} \times [0, \varepsilon _0], \, {{\mathcal {B}}}_{2, s, (N - 5) + 1})$ for any $s \ge s_N$. Hence ${{\mathcal {S}}^\bot _2}(\theta )[w, w] \in {{\mathcal {OS}}}_{ww}^2(N - 5)$. $\quad \square$

By Lemma 6.1 and in view of (6.37), (6.38), the vector field $X_6 = (X_6^{(\theta )}, X_6^{(y)}, X_6^\bot )$ takes the form

6.52 $$\begin{aligned} X_6^{(\theta )}({\mathfrak {x}})= & {} - \omega - \varepsilon {\widehat{\omega }} - \nabla _y Q(y) - {\mathcal {Z}}^{(\theta )}[w, w] + {{\mathcal {O}}}_3^{(\theta )}({\mathfrak {x}})\, , \qquad \quad \ X_6^{(y)}({\mathfrak {x}}) = {{\mathcal {O}}}_3^{(y)}({\mathfrak {x}}) \, ,\nonumber \\ X_6^\bot ({\mathfrak {x}})= & {} \mathrm{i} \Omega _\bot w + {{\mathcal {D}}}^\bot _6({\mathfrak {x}})[w] + {{\mathcal {OB}}}^3(1, N) + {{\mathcal {OS}}}^3(N - 6)\,, \qquad {{\mathcal {D}}}^\bot _6({\mathfrak {x}}) := {{\mathcal {D}}}^\bot _5({\mathfrak {x}}) + {{\mathcal {Z}}}^\bot (y)\,, \quad \nonumber \\&{{\mathcal {O}}}_3^{(\theta )}, {{\mathcal {O}}}_3^{(y)} \in C^\infty _b([0, \varepsilon _0] \times {{\mathcal {V}}}^{\sigma _N}(\delta ), \, {\mathbb {R}}^{S_+}) \quad \text { terms small of order three} \end{aligned}$$

for some $\sigma _N > 0$. Since by (6.22), $[{{\mathcal {R}}^\bot _{N, 1}}(\theta , y)]_j^j \in \mathrm{i} {\mathbb {R}}$, $j \in S^\bot$, and ${{\mathcal {Z}}}^\bot (y) = \mathrm{diag}_{j \in S^\bot } [\widehat{{\mathcal {R}}^\bot _1}(0, y)]_j^j$, the operator ${{\mathcal {Z}}}^\bot (y)$ is a skew-adjoint Fourier multiplier and hence by (6.35) so is ${{\mathcal {D}}}^\bot _6({\mathfrak {x}})$. We summarize our findings as follows.

#### Proposition 6.3

For any $N \in {\mathbb {Z}}_{\ge 6}$, there exists an integer $s_N > N$ with the property that for any $s \ge s_N$, there exist $0< \delta \equiv \delta (s, \gamma , N) <1$ and $0< \varepsilon _0 \equiv \varepsilon _0(s, \gamma , N) < 1$ so that the following holds. There exists a map $\Psi ^{(3)}$ with inverse $(\Psi ^{(3)})^{-1}$ (cf. Remark 3.4),

6.53 $$\begin{aligned} (\Psi ^{(3)})^{\pm 1} \in {{\mathcal {C}}}^\infty _b({{\mathcal {V}}}^s(\delta ) \times [0, \varepsilon _0], \, {{\mathcal {V}}}^s(2 \delta )), \quad \forall s \ge s_N\,, \quad (\Psi ^{(3)})^{\pm 1}({\mathfrak {x}} ) - {\mathfrak {x}} \quad \text {small of order two},\nonumber \\ \end{aligned}$$

so that the transformed vector field $X_6 := (\Psi ^{(3)})^* X_{5} = (X_6^{(\theta )}, \, X_6^{(y)}, \, X_6^\bot )$ has the form

6.54 $$\begin{aligned} \begin{aligned} X^{(\theta )}_6({\mathfrak {x}}) = - \omega -&\varepsilon {\widehat{\omega }} - \nabla _y Q(y) - {\mathcal {Z}}^{(\theta )}[w, w] + {{\mathcal {O}}}_3^{(\theta )}({\mathfrak {x}}) , \qquad \qquad X^{(y)}_6({\mathfrak {x}}) = {{\mathcal {O}}}_3^{(y)}({\mathfrak {x}}) \, , \\ X^\bot _6({\mathfrak {x}})&= \mathrm{i} \Omega _\bot w + {{\mathcal {D}}}^\bot _6({\mathfrak {x}})[w] + {{\mathcal {OB}}}^3(1, N) + {{\mathcal {OS}}}^3(N - 6) \, , \end{aligned} \end{aligned}$$

where ${{\mathcal {D}}}^\bot _6({\mathfrak {x}})$ is a Fourier multiplier of order one given by (6.52) and satisfies ${{\mathcal {D}}}^\bot _6({\mathfrak {x}}) = - {{\mathcal {D}}}^\bot _6({\mathfrak {x}})^\top$, where

6.55 $$\begin{aligned}&{\mathcal {Z}}^{(\theta )} \in {{\mathcal {B}}}_2(H^{\sigma _N}_\bot , {\mathbb {R}}^{S_+}), \\&{\mathcal {Z}}^{(\theta )}[w, w] = \sum _{j \in S^\bot } w_j w_{- j} \, \langle \Upsilon _2^{(\theta )}(\theta )[e^{\mathrm{i} 2 \pi j x}, e^{- \mathrm{i} 2 \pi j x}] \rangle _\theta , \nonumber \\&\quad \ \ \forall \, w \in H^{\sigma _N}_\bot ({\mathbb {T}}_1), \end{aligned}$$

for some $\sigma _N > 0$, and where ${{\mathcal {O}}}_3^{(\theta )}$, ${{\mathcal {O}}}_3^{(y)}$ comprises terms which are small of order three.

## Proofs of Theorem  4.2 and Theorem 4.3

First we prove Theorem 4.3.

### Proof of Theorem 4.3.

We apply Propositions 5.1, 6.1, 6.2, 6.3. Choose $N = 6$ and define

6.56 $$\begin{aligned} {\mathtt {\Phi }} := \Phi \circ \Psi ^{(1)} \circ \Psi ^{(2)} \circ \Psi ^{(3)}\,. \end{aligned}$$

By (5.1), (6.19), (6.33), (6.53), ${\mathtt {\Phi }}$ satisfies property (4.19). Moreover $X = X_6 = {\mathtt {\Phi }}^* X_{{\mathcal {H}}}$ is given in (6.54) with $N = 6$. Hence by setting

$$\begin{aligned} {\mathtt {D}}^\bot := {{\mathcal {D}}}^\bot _6\, , \qquad \mathtt {N}^{(\theta )}(y, w) := - \nabla _y Q(y) - {\mathcal {Z}}^{(\theta )}[w, w]\, , \end{aligned}$$

one has that ${\mathtt {D}}^\bot$, $\mathtt {N}^{(\theta )}$, ${{\mathcal {O}}}_3^{(\theta )}$, ${{\mathcal {O}}}_3^{(y)}$ satisfy the properties stated in (4.21). Since $N = 6$ , the remainder term ${{\mathcal {OB}}}^3(1, 6) + {{\mathcal {OS}}}^3(0)$ in the expansion of $X^\bot ({\mathfrak {x}}) = X^\bot _6({\mathfrak {x}})$ in (6.54) has the form (cf. Definitions 3.1, 3.3)

$$\begin{aligned} \Pi _\bot \sum _{k = 0}^7 T_{a_{1 - k}({\mathfrak {x}})} \partial _x^{1 - k} w + {{\mathcal {R}}}^\bot _0({\mathfrak {x}}) \end{aligned}$$

with the following property: there are integers $s_*$, $\sigma > 0$ so that for any $s \ge s_*$ there exist $0< \delta \equiv \delta (s, \gamma ) < 1$ and $0< \varepsilon _0 \equiv \varepsilon _0(s, \gamma ) < 1$ so that

7.1 $$\begin{aligned} \begin{aligned}&a_{1 - k} \in C^\infty _b \big ( {{\mathcal {V}}}^{s + \sigma }(\delta ) \times [0, \varepsilon _0], \, H^s({\mathbb {T}}_1) \big ) \quad \text {small of order two}, \qquad \forall \, 0 \le k \le 7 \, ,\\&{{\mathcal {R}}}^\bot _0 \in C^\infty _b \big ( {{\mathcal {V}}}^{s}(\delta ) \times [0, \varepsilon _0], \, H^s_\bot ({\mathbb {T}}_1) \big ) \quad \text {small of order three. } \end{aligned} \end{aligned}$$

We then define

$$\begin{aligned} a ({\mathfrak {x}}) := a_1({\mathfrak {x}}), \qquad {{\mathcal {R}}}^\bot ({\mathfrak {x}}) := \Pi _\bot \sum _{k = 0}^6 T_{a_{- k}({\mathfrak {x}})} \partial _x^{- k} w + {{\mathcal {R}}}^\bot _0({\mathfrak {x}})\, . \end{aligned}$$

One shows that ${{\mathcal {R}}}^\bot \in C^\infty _b( {{\mathcal {V}}}^s(\delta ) \times [0, \varepsilon _0], \, H^s_\bot ({\mathbb {T}}_1))$ for any $s \ge s_*+\sigma$ and that ${{\mathcal {R}}}^\bot$ is small of order three. Indeed, by (7.2) and the estimate (2.2) (paraproduct), it follows that for any ${\mathfrak {x}} \in {{\mathcal {V}}}^{s}(\delta )$,

$$\begin{aligned} \begin{aligned}&\Vert {{\mathcal {R}}}^\bot ({\mathfrak {x}}) \Vert _s \lesssim _{s, \gamma } \mathrm{max}_{0 \le k \le 7} \Vert a_{1 - k}({\mathfrak {x}}) \Vert _1 \Vert w \Vert _s + ( \varepsilon + \Vert y \Vert + \Vert w \Vert _s )^3 \\&\qquad \qquad \lesssim _{s, \gamma } \mathrm{max}_{0 \le k \le 7} \Vert a_{1 - k}({\mathfrak {x}}) \Vert _{s_*} \Vert w \Vert _s + ( \varepsilon + \Vert y \Vert + \Vert w \Vert _s )^3 \\&\qquad \qquad \lesssim _{s, \gamma } ( \varepsilon + \Vert y \Vert + \Vert w \Vert _{s_*+ \sigma } )^3 + ( \varepsilon + \Vert y \Vert + \Vert w \Vert _s )^3 . \end{aligned} \end{aligned}$$

Hence we proved that for any $s \ge s_*+ \sigma$,

$$\begin{aligned} \Vert {{\mathcal {R}}}^\bot ({\mathfrak {x}}) \Vert _s \lesssim _{s, \gamma } ( \varepsilon + \Vert y \Vert + \Vert w \Vert _s )^3. \end{aligned}$$

Theorem 4.3 then follows by choosing $\sigma _*:= s_*+ \sigma$. $\square$

Let us now turn to the proof of Theorem 4.2. It is based on energy estimates for the solutions of the equation $\partial _t {\mathfrak {x}} = X({\mathfrak {x}})$ where *X* is the vector field provided by Theorem 4.3 (cf. (4.20), (4.21))

7.2 $$\begin{aligned} {\left\{ \begin{array}{ll} \partial _t \theta (t) = - \omega - \varepsilon {\widehat{\omega }} + \mathtt {N}^{(\theta )}(y, w) + {{\mathcal {O}}}_3^{(\theta )}({\mathfrak {x}}) \\ \partial _t y (t) = {{\mathcal {O}}}_3^{(y)}({\mathfrak {x}}) \\ \partial _t w (t) = \mathrm{i} \Omega _\bot w + {\mathtt {D}}^\bot ({\mathfrak {x}})[w] + \Pi _\bot T_{a({\mathfrak {x}})} \partial _x w + {{\mathcal {R}}}^\bot ({\mathfrak {x}}). \end{array}\right. } \end{aligned}$$

Choose $\sigma _*> 0$ and for any $s \ge \sigma _*$, $0< \delta \equiv \delta (s, \gamma ) < 1$, $0 < \varepsilon _0 \equiv \varepsilon _0(s, \gamma ) \ll \delta$ as in Theorem 4.3. For any $s \ge \sigma _*$ and $0 < \varepsilon \le \varepsilon _0(s, \gamma )$ we then consider the Cauchy problem of (7.3) with small initial data ${\mathfrak {x}}_0 = (\theta _0, y_0, w_0) \in {\mathbb {T}}^{S_+} \times {\mathbb {R}}^{S_+} \times H^s_\bot ({\mathbb {T}}_1)$,

7.3 $$\begin{aligned} | y_0| , \, \Vert w_0 \Vert _s \le \varepsilon \,. \end{aligned}$$

Increasing $\sigma _*$ and decreasing $\varepsilon _0$, if needed, it follows from Proposition C.1 that for any $s \ge \sigma _*$ and $0 < \varepsilon \le \varepsilon _0$ there exists $T\equiv T_{\varepsilon , s, \gamma }> 0$ so that the Cauchy problem of (7.3) for any initial data ${\mathfrak {x}}_0 = (\theta _0, y_0, w_0)$ satisfying (7.4) has a unique solution $t \mapsto {\mathfrak {x}}(t) = (\theta (t), y(t), w(t))$ with

7.4 $$\begin{aligned}&\theta \in C^1([- T, T], {\mathbb {T}}^{S_+}), \quad y \in C^1([- T, T], {\mathbb {R}}^{S_+}), \nonumber \\&\quad \quad w \in C^0([- T, T], H^s_\bot ({\mathbb {T}}_1)) \cap C^1([- T, T], H^{s - 3}_\bot ({\mathbb {T}}_1)). \end{aligned}$$

In addition, by Proposition C.1 there exists $C_*\equiv C_*(\gamma ) > 1$ so that

7.5 $$\begin{aligned} | y(t) |, \, \Vert w(t) \Vert _s\, , \, |\Theta (t)| \, \le C_*\varepsilon \, , \qquad \forall t \in [- T, T]\,, \end{aligned}$$

where

7.6 $$\begin{aligned} \Theta (t) := \theta (t) - \theta _0 + (\omega + \varepsilon {\widehat{\omega }}) t - \int _0^t \mathtt {N}^{\theta }(y(\tau ), w(\tau ))\, d \tau , \qquad t \in [- T, T]. \end{aligned}$$

We now prove that the time *T* of existence of the solution can be chosen to be of size $\varepsilon ^{-2}$.

### Proposition 7.1

Let $$\sigma _*$$ and $$0< \varepsilon _0 \equiv \varepsilon _0(s, \gamma ) < 1$$, $$s \ge \sigma _*$$ be given as above. Then for any $$s \ge \sigma _*$$ there exists a constant $$C_{**} \equiv C_{**}(s, \gamma ) > 0$$ so that for any $$0 < \varepsilon \le \varepsilon _0$$, the time of existence *T* of the solution $${\mathfrak {x}}(t)$$ can be chosen as $$T_{\varepsilon , s, \gamma } := C_{**} \varepsilon ^{- 2}$$.

To prove the latter proposition, we first need to make some preliminary considerations. Let $s \ge \sigma _*$ and $0 < \varepsilon \le \varepsilon _0$. By (4.21), *a* is small of order two and ${{\mathcal {R}}}^\bot$, ${{\mathcal {O}}}_3^{(\theta )}$, ${{\mathcal {O}}}_3^{(y)}$ are small of order three, and by applying the estimates (7.6), one has

7.7 $$\begin{aligned} \begin{aligned}&|{{\mathcal {O}}}_3^{(\theta )}({\mathfrak {x}}(t))|\,, \, |{{\mathcal {O}}}_3^{(y)}({\mathfrak {x}}(t))| \lesssim _\gamma \varepsilon ^3, \qquad \Vert a({\mathfrak {x}}(t)) \Vert _{\sigma _*} \lesssim _\gamma \varepsilon ^2, \\&\quad \qquad \Vert {{\mathcal {R}}}^\bot ({\mathfrak {x}}(t)) \Vert _s \lesssim _{s, \gamma } \varepsilon ^3, \qquad \forall \, t \in [- T, T]\,. \end{aligned} \end{aligned}$$

First we prove the following lemma.

### Lemma 7.1

Given any $s \ge \sigma _*$, there exists a constant $K_0 \equiv K_0(s, \gamma ) > 0$ (large) so that the solutions (7.5) satisfy

7.8 $$\begin{aligned} |\Theta (t)| \le K_0 T \varepsilon ^3\,,\qquad |y(t)|, \, \Vert w(t) \Vert _s \le \varepsilon + K_0 \varepsilon ^3 T, \qquad \qquad \forall \, t \in [- T, T]\,. \end{aligned}$$

As a consequence, for any $T > 0$ satisfying $T \le \frac{1}{K_0} \varepsilon ^{-2}$, one has

7.9 $$\begin{aligned} |\Theta (t)| \le \varepsilon , \qquad |y(t)|\,,\, \Vert w(t) \Vert _s \le 2\varepsilon , \quad \forall t \in [- T, T]\,. \end{aligned}$$

### Proof of Lemma 7.1.

Let $s \ge \sigma _*$. First we prove the claimed estimates for $\Theta (t)$ and *y*(*t*). By the definition (7.7) of $\Theta$ and (7.3) (Hamiltonian equations), one has

$$\begin{aligned} \Theta (0) = 0, \qquad \quad \partial _t \Theta (t) = {{\mathcal {O}}}_3^{(\theta )}({\mathfrak {x}}(t)), \end{aligned}$$

implying that

$$\begin{aligned} \Theta (t) = \int _0^t {{\mathcal {O}}}_3^{(\theta )}({\mathfrak {x}}(\tau ))\, d \tau \,. \end{aligned}$$

Moreover by (7.3),

$$\begin{aligned} y(t) = y_0 + \int _0^t {{\mathcal {O}}}_3^{(y)}({\mathfrak {x}}(\tau ))\, d \tau \,. \end{aligned}$$

By (7.4) and (7.8), one then concludes that there exists a constant $C_1 \equiv C_1(s, \gamma ) > 0$ so that

7.10 $$\begin{aligned} | \Theta (t)| \le C_1 T \varepsilon ^3, \qquad |y(t)| \le \varepsilon + C_1 T \varepsilon ^3, \qquad \qquad \forall \, t \in [- T, T] \, . \end{aligned}$$

It remains to estimate the $H^s$-norm of *w*(*t*). To this end recall that for any $w \in H^s_\bot ({\mathbb {T}}_1)$,

$$\begin{aligned} \Vert w \Vert _s = \big (\sum _{j \in S^\bot } |j|^{2 s} |w_j|^2 \big )^{\frac{1}{2}} = \Vert \partial _x^s w \Vert \, , \end{aligned}$$

where $\Vert \partial _x^s w \Vert$ denotes the $L^2$-norm of $\partial _x^s w$. Then

7.11 $$\begin{aligned} \begin{aligned} \partial _t \Vert \partial _x^s w(t) \Vert ^2&= \big \langle \partial _x^s \partial _t w(t)\,,\, \partial _x^s w(t) \big \rangle + \big \langle \partial _x^s w(t)\,,\, \partial _x^s \partial _t w(t) \big \rangle \\&{\mathop {=}\limits ^{(7.3)}} \big \langle \partial _x^s\big ( \mathrm{i} \Omega _\bot w + {\mathtt {D}}^\bot ({\mathfrak {x}})[w] + \Pi _\bot T_{a({\mathfrak {x}})} \partial _x w + {{\mathcal {R}}}^\bot ({\mathfrak {x}}) \big )\,,\, \partial _x^s w \big \rangle \\&\quad + \big \langle \partial _x^s w \,,\, \partial _x^s \big (\mathrm{i} \Omega _\bot w + {\mathtt {D}}^\bot ({\mathfrak {x}})[w] + \Pi _\bot T_{a({\mathfrak {x}})} \partial _x w + {{\mathcal {R}}}^\bot ({\mathfrak {x}}) \big )\big \rangle \,. \end{aligned} \end{aligned}$$

Since $\Omega _\bot$ and ${\mathtt {D}}^\bot ({\mathfrak {x}})$ are both Fourier multipliers, the linear commutators with the Fourier multiplier $\partial _x^s$ vanish,

$$\begin{aligned} {[}\partial _x^s, \Omega _\bot ]_{lin}= 0 \, , \qquad [\partial _x^s, {\mathtt {D}}^\bot ({\mathfrak {x}})]_{lin} = 0\, . \end{aligned}$$

Using in addition that ${\mathtt {D}}^\bot ({\mathfrak {x}})$ is skew-adjoint (cf. (4.21)) and hence $(\mathrm{i} \Omega _\bot + {\mathtt {D}}^\bot ({\mathfrak {x}}))^\top = - \mathrm{i} \Omega _\bot - {\mathtt {D}}^\bot ({\mathfrak {x}})$, one infers

7.12 $$\begin{aligned} \begin{aligned}&\big \langle \partial _x^s \big ( \mathrm{i} \Omega _\bot w + {\mathtt {D}}^\bot ({\mathfrak {x}})[w] \big )\,,\, \partial _x^s w \big \rangle + \big \langle \partial _x^s w \,,\, \partial _x^s \big (\mathrm{i} \Omega _\bot w + {\mathtt {D}}^\bot ({\mathfrak {x}})[w] \big )\big \rangle \\&\qquad = \big \langle \big (\mathrm{i} \Omega _\bot + {\mathtt {D}}^\bot ({\mathfrak {x}}) \big ) \partial _x^s w\,,\, \partial _x^s w \big \rangle + \big \langle \partial _x^s w \,,\, \big (\mathrm{i} \Omega _\bot + {\mathtt {D}}^\bot ({\mathfrak {x}}) \big ) \partial _x^s w \big \rangle \\&\qquad = \big \langle \big (\mathrm{i} \Omega _\bot + {\mathtt {D}}^\bot ({\mathfrak {x}}) \big ) \partial _x^s w\,,\, \partial _x^s w \big \rangle + \big \langle \big (\mathrm{i} \Omega _\bot + {\mathtt {D}}^\bot ({\mathfrak {x}}) \big )^\top \partial _x^s w\,,\, \partial _x^s w \big \rangle = 0\,. \end{aligned} \end{aligned}$$

Moreover

7.13 $$\begin{aligned}&\big \langle \partial _x^s T_{a({\mathfrak {x}})} \partial _x w\,,\, \partial _x^s w \big \rangle + \big \langle \partial _x^s w \,,\, \partial _x^s T_{a({\mathfrak {x}})} \partial _x w\big \rangle \nonumber \\&\quad = \big \langle T_{a({\mathfrak {x}})} \partial _x \partial _x^s w\,,\, \partial _x^s w \big \rangle + \big \langle \partial _x^s w \,,\, T_{a({\mathfrak {x}})} \partial _x \partial _x^sw\big \rangle + \big \langle [\partial _x^s, T_{a({\mathfrak {x}})} \partial _x] w\,,\, \partial _x^s w \big \rangle + \big \langle \partial _x^s w \,,\, [\partial _x^s, T_{a({\mathfrak {x}})} \partial _x] w\big \rangle \nonumber \\&\quad = \big \langle \big ( T_{a({\mathfrak {x}})} \partial _x + (T_{a({\mathfrak {x}})} \partial _x)^\top \big ) \partial _x^s w\,,\, \partial _x^s w \big \rangle + \big \langle [\partial _x^s, T_{a({\mathfrak {x}})} \partial _x] w\,,\, \partial _x^s w \big \rangle + \big \langle \partial _x^s w \,,\, [\partial _x^s, T_{a({\mathfrak {x}})} \partial _x] w\big \rangle \,.\nonumber \\ \end{aligned}$$

By increasing $\sigma _*$ if needed one gets by Corollary 2.2 (with $N=1$, $m=1$)

$$\begin{aligned} \Vert \Pi _\bot T_{a({\mathfrak {x}})} \partial _x + \Pi _\bot (T_{a({\mathfrak {x}})} \partial _x)^\top \Vert _{{{\mathcal {B}}}(L^2_\bot )} \lesssim \Vert a({\mathfrak {x}}) \Vert _{\sigma _*} {\mathop {\lesssim _\gamma }\limits ^{(7.8)}} \varepsilon ^2 \end{aligned}$$

and hence by the Cauchy–Schwarz inequality,

7.14 $$\begin{aligned} \begin{aligned} |\big \langle \big ( T_{a({\mathfrak {x}})} \partial _x + (T_{a({\mathfrak {x}})} \partial _x)^\top \big ) \partial _x^s w\,,\, \partial _x^s w \big \rangle | \lesssim _\gamma \varepsilon ^2 \Vert \partial _x^s w \Vert \lesssim _\gamma \varepsilon ^2 \Vert w \Vert _s^2 \,. \end{aligned} \end{aligned}$$

Moreover, arguing as in [9, Lemma A.1], one has

$$\begin{aligned} \Vert [\partial _x^s, T_{a({\mathfrak {x}})} \partial _x] w\Vert _{L^2} \lesssim _{s} \Vert a({\mathfrak {x}}) \Vert _2 \Vert w \Vert _s {\mathop {\lesssim _{s, \gamma }}\limits ^{\sigma _*\ge 2, (7.8)}} \varepsilon ^2 \Vert w \Vert _s\,. \end{aligned}$$

The latter estimate, together with the Cauchy–Schwarz inequality, imply that

7.15 $$\begin{aligned} \begin{aligned} |\big \langle [\partial _x^s, T_{a({\mathfrak {x}})} \partial _x] w\,,\, \partial _x^s w \big \rangle + \big \langle \partial _x^s w \,,\, [\partial _x^s, T_{a({\mathfrak {x}})} \partial _x] w\big \rangle | \ \lesssim _{s, \gamma } \varepsilon ^2 \Vert w \Vert _s^2\,. \end{aligned} \end{aligned}$$

Finally, by using the Cauchy–Schwarz inequality once more and the estimate (7.8) for ${{\mathcal {R}}}^\bot$, one gets

7.16 $$\begin{aligned} \begin{aligned}&|\big \langle \partial _x^s {{\mathcal {R}}}^\bot ({\mathfrak {x}})\,,\, \partial _x^s w \big \rangle + \big \langle \partial _x^s w\,,\,\partial _x^s {{\mathcal {R}}}^\bot ({\mathfrak {x}}) \big \rangle | \lesssim \Vert {{\mathcal {R}}}^\bot ({\mathfrak {x}}) \Vert _s \Vert w \Vert _s \lesssim _{s, \gamma } \varepsilon ^3 \Vert w \Vert _s\,. \end{aligned} \end{aligned}$$

Thus, collecting (7.12)–(7.17), and since by (7.6), $\Vert w(t) \Vert _s \le C_*\varepsilon$ for any $t \in [- T, T]$, one gets

$$\begin{aligned} |\partial _t \, \Vert \partial _x^s w(t) \Vert ^2| \lesssim _{s, \gamma } \varepsilon ^4\, , \qquad \forall \, t \in [- T, T]. \end{aligned}$$

We then conclude that there exists a constant $C_2 \equiv C_2(s, \gamma ) > 0$ so that

7.17 $$\begin{aligned} \Vert w(t) \Vert _s \le (\Vert w_0 \Vert _s^2 + C_2 T \varepsilon ^4 )^{1/2} \le \varepsilon (1 + C_2 T \varepsilon ^2)^{1/2} \le \varepsilon + C_2 T \varepsilon ^3 , \quad \forall t \in [- T, T] \, .\qquad \end{aligned}$$

The claimed statement then follows with $K_0(s, \gamma ) := \max \{ C_1(s, \gamma ), C_2(s, \gamma ) \}$. $\quad \square$

### Proof of Proposition 7.1.

For any given $s \ge \sigma _*$, $0 < \varepsilon \le \varepsilon _0$, and initial data satisfying (7.4), consider the solution $t \mapsto {\mathfrak {x}}(t)$ in (7.5) of (7.3). It satisfies the estimates (7.9)–(7.10) of Lemma 7.1. Let

$$\begin{aligned} {\check{T}} := \mathrm{sup}\{ \, 0< T < \frac{1}{K_0} \varepsilon ^{-2} : \, 2 |\Theta (t)|, \, |y(t)|, \, \Vert w(t) \Vert _s \, \le 2 \varepsilon , \ \forall t \in [- T, T] \} \, , \end{aligned}$$

where $K_0 \equiv K_0(s, \gamma )$ is given by Lemma 7.1, and define

$$\begin{aligned} M(T) := \max _{ \, |t| \le T}\{2 |\Theta ( t)|, |y( t)|, \Vert w( t) \Vert _s \}, \quad T \in [0, {\check{T}})\,. \end{aligned}$$

Assume that ${\check{T}} \le \frac{1}{2} \frac{1}{K_0} \varepsilon ^{- 2}$. By the definition of ${\check{T}}$ and Proposition C.1 it then follows that $\sup _{T < {\check{T}}} M(T) = 2 \varepsilon$. On the other hand, from Lemma 7.1 one infers that

$$\begin{aligned} \begin{aligned} M( {\check{T}}) \le \varepsilon + K_0 \varepsilon ^3 {\check{T}} \le \varepsilon (1 + 1/2 ) \le \frac{3}{2} \varepsilon \, . \end{aligned} \end{aligned}$$

Hence we obtained a contradiction and thus conclude that ${\check{T}} = O(\varepsilon ^{- 2})$. $\square$

### Proof of Theorem 4.2.

Let $t \mapsto {\mathfrak {x}}(t) = (\theta (t), y(t), w(t))$ be a curve satisfying (7.4)–(7.6). By Theorem 4.3 (Normal Form Theorem), ${\mathfrak {x}}(t) = (\theta (t), y(t), w(t))$ is a solution of (7.3) if and only if

$$\begin{aligned} {\mathfrak {x}}'(t) = (\theta '(t), y'(t), w'(t)) := {\mathtt {\Phi }}({\mathfrak {x}}(t)) \end{aligned}$$

is a solution of (4.17) with initial data ${\mathfrak {x}}'_0 = {\mathtt {\Phi }}({\mathfrak {x}}_0)$.

By (4.19) (properties of the transformation $\mathtt {\Phi }$), for any ${\mathfrak {x}}$ in ${\mathcal {V}}^s(\delta )$ with ${\mathfrak {x}}' := {\mathtt {\Phi }}({\mathfrak {x}}) \in {\mathcal {V}}^s(\delta )$ one has ${\mathfrak {x}} = {\mathtt {\Phi }}^{- 1}({\mathfrak {x}}')$ and

$$\begin{aligned} | y'|, \Vert w' \Vert _s \le C(s, \gamma )\big ( \varepsilon + | y | + \Vert w\Vert _s \big )\, , \qquad |y|, \Vert w \Vert _s \le C(s, \gamma ) \big ( \varepsilon + | y' | + \Vert w'\Vert _s \big ) \end{aligned}$$

for some constant $C(s, \gamma ) > 0$. Hence, if ${\mathfrak {x}}(t)$ satisfies (7.4)–(7.6), then ${\mathfrak {x}}_0' \in {\mathbb {T}}^{S_+} \times {\mathbb {R}}^{S_+} \times H^s_\bot ({\mathbb {T}}_1)$ with $| y_0' |$, $\Vert w_0' \Vert _s \le C(s, \gamma ) \varepsilon$ and

$$\begin{aligned}&\theta ' \in C^1([- T, T], {\mathbb {T}}^{S_+}), \quad y' \in C^1([- T, T], {\mathbb {R}}^{S_+}), \\&\quad \quad w' \in C^0([- T, T], H^s_\bot ({\mathbb {T}}_1)) \cap C^1([- T, T], H^{s - 3}_\bot ({\mathbb {T}}_1)) \end{aligned}$$

with

$$\begin{aligned} | y'(t) |, \, \Vert w'(t) \Vert _s\, \le 2 C(s, \gamma ) \varepsilon \, , \quad \forall t \in [- T, T]\,. \end{aligned}$$

By Proposition 7.1, *T* can be chosen as $T_{\varepsilon , s, \gamma } = O(\varepsilon ^{- 2})$. This proves Theorem 4.2 . $\quad \square$

## Measure Estimates

In this section we prove the measure estimate (1.23) of the set $\Pi _\gamma$ defined in (1.19), (1.20). More precisely we show the following

### Proposition 8.1

There exists $\mathtt {a} \in (0, 1)$ so that for any $0 \le j \le 3$ and any $0< \gamma < 1$, $| \Pi {\setminus } \Pi _\gamma ^{(j)} | \lesssim \gamma ^{\mathtt {a}}$.

We will concentrate on the proof of the claimed measure estimate of $\Pi _\gamma ^{(3)}$. The ones of $\Pi _\gamma ^{(0)}$, $\Pi _\gamma ^{(1)}$, and $\Pi _\gamma ^{(2)}$ can be obtained in a similar way and are in fact a bit easier to prove. Recall that

7.18 $$\begin{aligned} \Pi _\gamma ^{(3)}= & {} \Big \{ \omega \in \Pi : |\omega \cdot \ell + \Omega _{j_1} (\omega ) + \Omega _{j_2}(\omega ) + \Omega _{j_3}(\omega )| \ge \frac{\gamma }{\langle \ell \rangle ^\tau \langle j_1 \rangle ^2 \langle j_2 \rangle ^2 \langle j_3 \rangle ^2}, \nonumber \\&\qquad \forall (\ell , j_1, j_2, j_3) \in {\mathbb {Z}}^{S_+} \times S^\bot \times S^\bot \times S^\bot \quad \text {with}\quad j_k + j_m \ne 0, \quad \forall k, m \in \{ 1,2,3\}\Big \}\,\nonumber \\ \end{aligned}$$

where for any $j \in S^\bot$, $\Omega _j(\omega ) := \omega ^{kdv}_j(\mu (\omega ), 0)$. One has

$$\begin{aligned} \Pi {\setminus } \Pi _\gamma ^{(3)} \subset \bigcup _{\begin{array}{c} \ell \in {\mathbb {Z}}^{S_+}, j_1, j_2, j_3 \in S^\bot \\ j_k + j_m \ne 0, \forall k, m \in \{ 1,2,3\} \end{array}} R_{\ell j_1 j_2 j_3}(\gamma )\,, \end{aligned}$$

where

$$\begin{aligned} R_{\ell j_1 j_2 j_3}(\gamma ) = \Big \{ \omega \in \Pi : |\omega \cdot \ell + \Omega _{j_1}(\omega ) + \Omega _{j_2}(\omega ) + \Omega _{j_3}(\omega )| < \frac{\gamma }{\langle \ell \rangle ^\tau \langle j_1 \rangle ^2 \langle j_2 \rangle ^2 \langle j_3 \rangle ^2} \Big \}\,. \end{aligned}$$

First we need to establish the following regularity properties and asymptotics for the normal frequencies $\Omega _j(\omega )$, $j \in S^\bot$.

### Lemma 8.1

The map

$$\begin{aligned} \Omega ^*: \Pi \rightarrow \ell ^\infty (S^\bot , {\mathbb {R}}), \, \omega \mapsto (\Omega ^*_j(\omega ))_{j \in S^\bot }\, , \qquad \Omega ^*_j(\omega ) := j \big ( \Omega _j(\omega ) - (2\pi j)^3 \big )\, , \end{aligned}$$

is real analytic. Furthermore, uniformly on a complex neighborhood of $\Pi$ in ${\mathbb {C}}^{S_+}$,

8.1 $$\begin{aligned} \Omega _j(\omega ) = (2 \pi j)^3 + O(j^{- 1}) \, \quad \text {as } j \rightarrow \pm \infty \, . \end{aligned}$$

### Proof

Since by [26, Theorem 1.2 (i)], $\Xi \rightarrow \ell ^\infty (S^\bot _+, {\mathbb {R}}), \, I \mapsto (\omega ^{kdv}_j(I, 0))_{j \in S_+^\bot }$ is real analytic and since by [26, Theorem 1.2 (iii)]

$$\begin{aligned} \Xi \rightarrow \ell ^\infty (S^\bot _+, {\mathbb {R}}), \, I \mapsto \big (j (\omega ^{kdv}_j(I, 0) - (2\pi j)^3) \big )_{j \in S_+^\bot } \end{aligned}$$

is locally bounded in a complex neighborhood of $\Pi$ in ${\mathbb {C}}^{S_+}$, it follows from [28, Theorem A.3] that the latter map is real analytic. Furthermore, by [28, Theorem 15.4], the action to frequency map

$$\begin{aligned} \Xi \rightarrow \Pi , \, I = (I_j)_{j \in S_+} \mapsto (\omega _j^{kdv}(I, 0))_{j \in S_+} \end{aligned}$$

is real analytic and by the definition of $\Xi$ and $\Pi$, it is a diffeomorphism. Hence its inverse $\mu : \Pi \rightarrow \Xi , \omega \mapsto \mu (\omega )$ is also a real analytic diffeomorphism. Since for any $\omega \in \Pi$ and $j \in S^\bot$, $\Omega _j(\omega ) = \omega ^{kdv}_j(\mu (\omega ), 0)$ and $\Omega _j(\omega ) = - \Omega _{- j}(\omega )$ we altogether have proved that the composition

$$\begin{aligned} \Omega ^* : \Pi \rightarrow \ell ^\infty (S^+, {\mathbb {R}}), \, \omega \mapsto (j (\omega ^{kdv}_j(\mu (\omega ), 0) - (2\pi j)^3)_{j \in S^\bot } \end{aligned}$$

is real analytic. Since $\Pi \subset {\mathbb {R}}^{S_+}$ is compact, $\Omega ^*$ is actually bounded on a complex neighborhood of $\Pi$ in ${\mathbb {C}}^{S_+}$ and hence the claimed asymptotics hold. $\square$

### Lemma 8.2

There exist constants $C_0 > 0$ and $C_1 > 0$ so that for any $j_1, j_2, j_3 \in S^\bot$ and any $\ell \in {\mathbb {Z}}^{S_+}$ with $|\ell | \ge C_1$

$$\begin{aligned} |R_{\ell j_1 j_2 j_3}(\gamma )| \le C_0 \frac{\gamma }{\langle \ell \rangle ^\tau \langle j_1 \rangle ^2 \langle j_2 \rangle ^2 \langle j_3 \rangle ^2}\,. \end{aligned}$$

### Proof

Let $\ell \in {\mathbb {Z}}^{S_+} {\setminus } \{ 0 \}$. Choose $v \in {\mathbb {R}}^{S_+}$ with $v \cdot \ell = 0$ and introduce $s \mapsto \omega (s) := s \frac{\ell }{|\ell |} + v$. Then $\ell \cdot \omega (s) = s |\ell |$ and hence for any $j_1, j_2, j_3 \in S^\bot$ and any $s \in {\mathbb {R}}$ with $\omega (s) \in \Pi ,$

$$\begin{aligned}&\varphi (s) := \ell \cdot \omega (s) + \Omega _{j_1}(\omega (s)) + \Omega _{j_2}(\omega (s)) + \Omega _{j_3}(\omega (s))\\&\quad = s |\ell | + \Omega _{j_1}(\omega (s)) + \Omega _{j_2}(\omega (s)) + \Omega _{j_3}(\omega (s)). \end{aligned}$$

By Lemma 8.1 and Cauchy’s theorem there exists $C >0$, independent of $j_1, j_2, j_3 \in S^\bot$, so that

$$\begin{aligned} \big | \frac{d}{ds} \big ( \Omega _{j_1}(\omega (s)) + \Omega _{j_2}(\omega (s)) + \Omega _{j_3}(\omega (s)) \big ) \big | \le C\, . \end{aligned}$$

It then follows that $|\varphi '(s)| \ge 1$ for any $|\ell | \ge C_1:= C+1$. This implies the claimed estimate. $\square$

### Lemma 8.3

There exist constants $C_0 > 0$, $C_2 > 0$ so that for $j_1, j_2, j_3 \in S^\bot$ with $\min \{ |j_1|, |j_2|, |j_3| \} \ge C_2$ one has

8.2 $$\begin{aligned} R_{0 j_1 j_2 j_3} (\gamma ) = \emptyset \, , \qquad |R_{\ell j_1 j_2 j_3}(\gamma )| \le C_0 \frac{\gamma }{\langle \ell \rangle ^\tau \langle j_1 \rangle ^2 \langle j_2 \rangle ^2 \langle j_3 \rangle ^2}\, , \quad \forall \, \ell \in {\mathbb {Z}}^{S_+}{\setminus }\{0\} .\quad \end{aligned}$$

### Proof

First we consider the case $\ell = 0$. By the asymptotics (8.2) it follows that for any $j_1, j_2, j_3 \in S^\bot$,

$$\begin{aligned} |\Omega _{j_1} + \Omega _{j_2} + \Omega _{j_3}| \ge 8\pi ^3 |j_1^3 + j_2^3 + j_3^3| - \frac{C}{\min \{|j_1|, |j_2|, |j_3| \}} \end{aligned}$$

for some constant $C > 0$. By the case $n=3$ of Fermat’s Last Theorem (cf. [21])

$$\begin{aligned} |j_1^3 + j_2^3 + j_3^3| \ge 1\, . \end{aligned}$$

Requesting that $\mathrm{min}\{|j_1|, |j_2|, |j_3| \} \ge C_2:= 2C$, one gets $|\Omega _{j_1} + \Omega _{j_2} + \Omega _{j_3}| \ge 4\pi ^3$ and hence $R_{0 j_1 j_2 j_3}(\gamma ) = \emptyset$ for any such $j_1, j_2 , j_3$ in $S^\bot$.

Now let us consider the case $\ell \in {\mathbb {Z}}^{S_+} {\setminus } \{ 0 \}$. For any given $j_1, j_2, j_3 \in S^\bot$, define $s \mapsto \varphi (s)$ as in the proof of Lemma 8.2,

$$\begin{aligned} \varphi (s) := |\ell | s + \Omega _{j_1}(\omega (s)) + \Omega _{j_2}(\omega (s)) + \Omega _{j_3}(\omega (s))\,. \end{aligned}$$

By Lemma 8.1 there exists $C >0$, independent of $j_1, j_2, j_3 \in S^\bot$, so that

$$\begin{aligned} \big | \frac{d}{ds} j_k \Omega _{j_k}(\omega (s)) \big | \le C\, , \qquad \forall \, 1 \le k \le 3\, . \end{aligned}$$

By increasing $C_2$ if needed, it follows that for $j_1, j_2, j_3 \in S^\bot$ satisfying $\min \{ |j_1|, |j_2|, |j_3|\} {\ge } C_2$,

$$\begin{aligned} |\varphi '(s)| \ge |\ell | - \frac{3C}{\min \{ |j_1|, |j_2|, |j_3|\}} \ge \frac{1}{2} \, . \end{aligned}$$

This implies the claimed measure estimate (8.3). $\quad \square$

### Lemma 8.4

There exists a constant $C_3 \ge \max \{ C_2, C_1 \}$, where $C_2$ is the constant of Lemma 8.3 and $C_1$ the constant of Lemma 8.2, so that

$$\begin{aligned} R_{\ell j_1 j_2 j_3} (\gamma ) = \emptyset \, \qquad \forall \ell \in {\mathbb {Z}}^{S_+} \text { with } |\ell | < C_1\, \text { and } \, \, \forall \, j_1, j_2, j_3 \in S^\bot \text { satisfying } (*) \end{aligned}$$

where

$$\begin{aligned} (*) \qquad j_k + j_m \ne 0, \quad \forall \, k, m \in \{ 1,2,3 \}\, , \qquad \min \{ |j_1|, |j_2|, |j_3| \} < C_2 \, , \quad \max \{ |j_1|, |j_2|, |j_3| \} \ge C_3 \, . \end{aligned}$$

### Proof

Let $\ell \in {\mathbb {Z}}^{S_+}$ with $|\ell | \le C_1$ and $j_1, j_2, j_3 \in S^\bot$ with $\min \{ |j_1|, |j_2|, |j_3| \} \le C_2$ and $j_k + j_m \ne 0$ for any $k, m \in \{ 1,2,3 \}$. First consider the case where $|j_2|, |j_3| < C_2$. By Lemma 8.1 one then has for $|j_1| \ge C_3$ with $C_3 > 0$ chosen large enough,

$$\begin{aligned} |\omega \cdot \ell + \Omega _{j_1} + \Omega _{j_2} + \Omega _{j_3}| \, \ge \, 8\pi ^3 ( |j_1|^3 - |j_2|^3 - |j_3|^3) - C - |\omega | C_1 \, \ge \, C_3^3 - 2 C_2^3 - C - |\omega | C_1 \ge 1 \, , \end{aligned}$$

implying that $$R_{\ell j_1 j_2 j_3}(\gamma ) = \emptyset $$.

Let us now turn to the case where $|j_1| , |j_2| \ge C_3$ and $|j_3| \le C_2$. If $j_1$ and $j_2$ have the same sign, then one concludes again that

$$\begin{aligned} |\omega \cdot \ell + \Omega _{j_1} + \Omega _{j_2} + \Omega _{j_3}| \ge 8 \pi ^3(|j_1|^3 + |j_2|^3 - |j_3|^3) - C - |\omega | C_1 \ge 2 C_3^3 - C_2^3 - C - |\omega | C_1 \ge 1 \end{aligned}$$

by increasing $C_3$ if needed. Hence again $R_{\ell j_1 j_2 j_3}(\gamma ) = \emptyset$. Now assume that $j_1$ and $j_2$ do not have the same sign. Since by assumption, $j_1 + j_2 \ne 0$, one has $|j_1| - |j_2| \ne 0$ and it then follows that

$$\begin{aligned} \begin{aligned} |\omega \cdot \ell + \Omega _{j_1} + \Omega _{j_2} + \Omega _{j_3} |&\ge ||j_1|^3 - |j_2|^3| - |j_3|^3 - C - C_1 |\omega | \\&\ge |(|j_1| - |j_2|)|(|j_1|^2 + |j_1||j_2| + |j_2|^2) - C_2^3 - C - C_1 |\omega | \\&\ge 3 C_3^2 - C_2^3 - C - C_1 |\omega | \ge 1 \end{aligned} \end{aligned}$$

by increasing $C_3$ once more if needed. We conclude that also in this case $R_{\ell j_1 j_2 j_3}(\gamma ) =\emptyset$. $\square$

### Proof of Proposition 8.1

As already mentioned, we concentrate on the proof of the claimed estimate for $| \Pi {\setminus } \Pi _\gamma ^{(3)} |$. In view of Lemmas 8.2–8.4, it remains to estimate the measure of the finite union

$$\begin{aligned} \bigcup _{\begin{array}{c} |\ell | \le C_1 \\ |j_1|, |j_2|, |j_3| \le C_3 \end{array}} R_{\ell j_1 j_2 j_3}(\gamma ) \end{aligned}$$

where $C_1 > 0$ is given by Lemma 8.2 and $C_3 > 0$ by Lemma 8.4. By Lemma 8.1, for any $\ell \in {\mathbb {Z}}^{S_+}$, $j_1, j_2, j_3 \in S^\bot$ with $|\ell | \le C_1$ and $|j_1|, |j_2|, |j_3| \le C_3$, the function

$$\begin{aligned} \omega \mapsto \omega \cdot \ell + \Omega _{j_1}(\omega ) + \Omega _{j_2}(\omega ) + \Omega _{j_3}(\omega ) \end{aligned}$$

is real analytic and by [28, Proposition 15.5], does not vanish identically. Hence by the Weierstrass Preparation Theorem (cf. [8, Lemma 9.7], [10, Proposition 3.1]), for any given $C > 0$ there exists $\mathtt {a} \in (0, 1)$ so that

$$\begin{aligned} \big | \bigcup _{\begin{array}{c} |\ell | \le C_1 \\ |j_1|, |j_2|, |j_3| \le C_3 \end{array}} \big \{ \omega \in \Pi : |\omega \cdot \ell + \Omega _{j_1}(\omega ) + \Omega _{j_2}(\omega ) + \Omega _{j_3}(\omega )| \le C \gamma \big \} \big | \lesssim \gamma ^{\mathtt {a}} \end{aligned}$$

and the claimed estimate for $| \Pi {\setminus } \Pi _\gamma ^{(3)} |$ follows. $\quad \square$

### Remark 8.1

Note that there exist (many) non-trivial solutions of the diophantine equation

8.3 $$\begin{aligned} j_1^3 + j_2^3 + j_3^3 + j_4^3 = 0 \end{aligned}$$

where $(j_1, j_2, j_3, j_4) \in {\mathbb {Z}}^4$ is said to be a trivial solution if there exist $1 \le \alpha < \beta \le 4$ so that $j_\alpha = - j_\beta$. The following example was suggested by Michela Procesi,

$$\begin{aligned} {(}10)^3 + 9^3 + (-1)^3 + (-12)^3 = 0 \, . \end{aligned}$$

We therefore expect that Lemma 8.3 does not extend to the sets $R_{\ell j_1 j_2 j_3 j_4} (\gamma )$, defined as

$$\begin{aligned} R_{\ell j_1 j_2 j_3 j_4}(\gamma ) : = \Big \{ \omega \in \Pi : \big | \omega \cdot \ell + \sum _{k=1}^4\Omega _{j_k}(\omega ) \big | < \frac{\gamma }{\langle \ell \rangle ^\tau \langle j_1 \rangle ^2 \langle j_2 \rangle ^2 \langle j_3 \rangle ^2 \langle j_4 \rangle ^2} \Big \} \end{aligned}$$

and hence that an estimate for $|\Pi {\setminus } \Pi ^{(4)}_\gamma |$ of the type as in Proposition 8.1 for $|\Pi {\setminus } \Pi ^{(3)}_\gamma |$ does not hold. Here $\Pi _\gamma ^{(4)}$ is defined as

$$\begin{aligned} \begin{aligned} \Pi _\gamma ^{(4)}&:= \big \{ \omega \in \Pi \ : \ |\omega \cdot \ell + \sum _{k = 1}^4 \Omega _{j_k}(\omega ) | \ge \frac{\gamma }{\langle \ell \rangle ^\tau \langle j_1 \rangle ^2 \langle j_2 \rangle ^2 \langle j_3 \rangle ^2 \langle j_4 \rangle ^2} \\&\qquad \forall (\ell , j_1, j_2, j_3, j_4) \in {\mathbb {Z}}^{S_+} \times (S^\bot )^4 \ \text {with} \ j_k + j_m \ne 0 \ \ \forall k, m \in \{1,2,3, 4\} \big \} \, . \end{aligned} \end{aligned}$$

## Linear Vector Fields on $H^s_\bot ({\mathbb {T}}_1)$

In this appendix we discuss properties of linear vector fields on $H^s_\bot ({\mathbb {T}}_1)$, used throughout the main body of the paper. Let *X* be an unbounded linear vector field on $H^s_\bot ({\mathbb {T}}_1)$, $s \in {\mathbb {N}}$, with domain $H^{s + 1}_\bot ({\mathbb {T}}_1)$,

$$\begin{aligned} X : H^{s}_\bot ({\mathbb {T}}_1) \rightarrow H^{s-1}_\bot ({\mathbb {T}}_1)\, , \end{aligned}$$

which admits an expansion of order $N \in {\mathbb {N}}$,

8.4 $$\begin{aligned} X [w] = \sum _{k = 0}^{N+1} \lambda _{1 - k} \partial _x^{1 - k} w + {{\mathcal {R}}}_N [w]\, , \qquad \lambda _{1 - k} \in {\mathbb {R}}, \quad \forall \, 0 \le k \le N + 1\, , \end{aligned}$$

where the remainder ${{\mathcal {R}}}_N$ is $(N +1)$-regularizing, ${{\mathcal {R}}}_N \in {{\mathcal {B}}}(H^s_\bot ({\mathbb {T}}_1), H^{s + N +1}_\bot ({\mathbb {T}}_1))$. If in addition, *X* is a Hamiltonian linear vector field on $H^s_\bot ({\mathbb {T}}_1)$,

$$\begin{aligned} X[w] = \partial _x \nabla H[w]\, , \qquad H(w) := \frac{1}{2}\int _0^1 A[w] \cdot w d x \, , \quad \forall w \in H^s_\bot ({\mathbb {T}}_1), \end{aligned}$$

where $A: H^s_\bot ({\mathbb {T}}_1) \rightarrow H^s_\bot ({\mathbb {T}}_1)$ is a symmetric, bounded linear operator, then the diagonal matrix elements $X_j^j$ of *X* satisfy

A.1 $$\begin{aligned} X_j^j = \int _0^1 \partial _x A [e^{\mathrm{i} 2\pi j x}] \cdot e^{-\mathrm{i} 2\pi j x} d x \, \in \,\mathrm{i} {\mathbb {R}}, \qquad \forall j \in S^\bot . \end{aligned}$$

### Lemma A.1

Let *X* be a vector field as in (A.1) and assume that its diagonal matrix elements satisfy $X_j^j \in \mathrm{i} {\mathbb {R}}$ for any $j \in S^\bot$. Then $\lambda _{1 - k} = 0$ for any $0 \le k \le N + 1$ with $1 - k$ even and $({{\mathcal {R}}}_N)_j^j \in \mathrm{i} {\mathbb {R}}$ for any $j \in S^\bot$.

### Proof

It follows from the assumptions that for any $j \in S^\bot$,

$$\begin{aligned} X_j^j = - {\overline{X}}_j^j \, , \quad X_j^j = \sum _{k = 0}^{N+1} \lambda _{1 - k} (\mathrm{i} 2 \pi j)^{1 - k} + ({{\mathcal {R}}}_N)_j^j \quad \text {with} \quad \lambda _{1-k} \in {\mathbb {R}}\, , \quad ({{\mathcal {R}}}_N)_j^j = O(j^{- N - 1})\, . \end{aligned}$$

One thus concludes that

$$\begin{aligned} \sum _{k = 0}^{N+1} \lambda _{1 - k} (\mathrm{i} 2\pi j)^{1 - k} + O(j^{- N -1}) = - \sum _{k = 0}^{N+1} \lambda _{1 - k}(- 1)^{1 - k} (\mathrm{i} 2\pi j)^{1 - k} + O(j^{- N -1}) \end{aligned}$$

and hence $\lambda _{1 - k} = 0$ for any $0 \le k \le N +1$ with $1 - k$ even. This implies that

$$\begin{aligned} {(}{{\mathcal {R}}}_N)_j^j = X^j_j - \sum _{k = 0}^{N+1} \lambda _{1 - k} (\mathrm{i} 2\pi j)^{1 - k} \in \mathrm{i} {\mathbb {R}}\, , \qquad \forall \, j \in S^\bot . \end{aligned}$$

$$\square $$

Consider a vector field $X : H^{s}_\bot ({\mathbb {T}}_1) \rightarrow H^{s-1}_\bot ({\mathbb {T}}_1)$, admitting an expansion of order *N* of the form

A.2 $$\begin{aligned} X[w]= \Pi _\bot \sum _{k = 0}^{N +1} T_{a_{1 - k}} \partial _x^{1 - k} w + {{\mathcal {R}}}_N[w], \quad a_{1 - k}\in H^s({\mathbb {T}}_1)\,, \quad \forall \, 0 \le k \le N + 1\, ,\qquad \end{aligned}$$

where the remainder ${{\mathcal {R}}}_N$ is $(N+1)$-regularizing, ${{\mathcal {R}}}_N \in {{\mathcal {B}}}(H^s_\bot ({\mathbb {T}}_1), H^{s + N + 1}_\bot ({\mathbb {T}}_1))$.

### Lemma A.2

Let *X* be a vector field as in (A.3) and assume that $X_j^j \in \mathrm{i} {\mathbb {R}}$ for any $j \in S^\bot$. Then $\langle a_{1 - k } \rangle _x = 0$ for any $0 \le k \le N + 1$ with $1-k$ even and $({{\mathcal {R}}}_N)_j^j \in \mathrm{i} {\mathbb {R}}$ for any $j \in S^\bot$.

### Proof

For any $j \in S^\bot$, a direct calculation shows that

$$\begin{aligned} X_j^j = \sum _{k = 0}^{N+1}\lambda _{1 - k} (\mathrm{i} 2 \pi j)^{1 - k} + ({{\mathcal {R}}}_N)_j^j, \qquad \lambda _{1 - k} := \langle a_{1 - k} \rangle _x \in {\mathbb {R}}, \quad \forall 0 \le k \le N + 1\,. \end{aligned}$$

Since by assumption $X_j^j$ is purely imaginary, the claimed results then follow from Lemma A.1. $\square$

## Standard Results on Homological Equations

In this appendix we record two standard results on homological equations, used in our normal form procedure. Without further reference, we use the notations introduced in the paragraph *Notations and terminology* in Sect. 1.

### Lemma B.1

Let $\gamma \in (0, 1)$, $\tau > 0$, and $\omega \in {\mathbb {R}}^{S_+}$. Assume that

$$\begin{aligned} |\omega \cdot \ell | \ge \frac{\gamma }{|\ell |^\tau }, \qquad \forall \ell \in {\mathbb {Z}}^{S_+} {\setminus } \{ 0 \} , \end{aligned}$$

and that ${{\mathcal {P}}} \in C^\infty ({\mathbb {T}}^{S_+}, B)$ where *B* is a Banach space with norm $\Vert \cdot \Vert _B$. Then there exists a unique solution ${{\mathcal {F}}} \in C^\infty ({\mathbb {T}}^{S_+}, B)$ with zero average of

$$\begin{aligned} \omega \cdot \partial _\theta \, {{\mathcal {F}}}(\theta ) + {{\mathcal {P}}}(\theta ) = \langle {{\mathcal {P}}} \rangle _\theta \,, \qquad \langle {{\mathcal {P}}} \rangle _\theta := \int _{{\mathbb {T}}^{S_+}} {\mathcal {F}}(\theta ) d \theta = 0 \, . \end{aligned}$$

It is denoted by ${{\mathcal {F}}}(\theta ) = - (\omega \cdot \partial _\theta )^{- 1}\big ( {{\mathcal {P}}}(\theta ) - \langle {{\mathcal {P}}} \rangle _\theta \big )$.

### Lemma B.2

Let $\Omega _\bot : L^2_\bot ({\mathbb {T}}_1) \rightarrow L^2_\bot ({\mathbb {T}}_1)$ be a (possibly unbounded) Fourier multiplier of diagonal form, $\Omega _\bot [w] := \sum _{n \in S^\bot } \Omega _n w_n e^{\mathrm{i} 2\pi n x}$, and let $0< \gamma < 1$, $\tau > 0$, and $\omega \in {\mathbb {R}}^{S_+}$. Assume that

$$\begin{aligned} |\omega \cdot \ell + \Omega _n| \ge \frac{\gamma }{\langle \ell \rangle ^\tau }, \qquad \forall \, (\ell , n) \in {\mathbb {Z}}^{S_+} \times S^\bot , \end{aligned}$$

and that ${{\mathcal {P}}} \in C^\infty ({\mathbb {T}}^{S_+}, H^s_\bot ({\mathbb {T}}_1))$ for any $s \ge 0$. Then there exists a unique solution ${{\mathcal {F}}} \in C^\infty ({\mathbb {T}}^{S_+}, H^0_\bot ({\mathbb {T}}_1))$ of the equation

$$\begin{aligned} \big ( \omega \cdot \partial _\theta + \mathrm{i} \Omega _\bot \big ) {{\mathcal {F}}}(\theta ) + {{\mathcal {P}}}(\theta ) = 0\,. \end{aligned}$$

Furthermore, ${{\mathcal {F}}} \in C^\infty ({\mathbb {T}}^{S_+}, H^s_\bot ({\mathbb {T}}_1))$ for any $s \ge 0$.

## A Local Existence Result for $\partial _t {\mathfrak {x}} \hbox {=} X({\mathfrak {x}})$

The goal of this appendix is to state a local existence result for the equation $\partial _t {\mathfrak {x}} = X({\mathfrak {x}})$ where *X* is the vector field, introduced in Theorem 4.3 (cf. (4.20), (4.21)),

A.3 $$\begin{aligned} {\left\{ \begin{array}{ll} \partial _t \theta = - \omega - \varepsilon {\widehat{\omega }} + \mathtt {N}^{(\theta )}(y, w) + {{\mathcal {O}}}_3^{(\theta )}({\mathfrak {x}}) \\ \partial _t y = {{\mathcal {O}}}_3^{(y)}({\mathfrak {x}}) \\ \partial _t w= \mathrm{i} \Omega _\bot w + {\mathtt {D}}^\bot ({\mathfrak {x}})[w] + \Pi _\bot T_{a({\mathfrak {x}})} \partial _x w + {{\mathcal {R}}}^\bot ({\mathfrak {x}}) \end{array}\right. } \end{aligned}$$

where we assume that the assumptions of Theorem 4.3 are satisfied. In particular, $\omega \in \Pi _\gamma$, $0< \gamma < 1$. This local existence result is used in Sect. 7. It reads as follows.

### Proposition C.1

There exists $\sigma _*> 0$ (large) so that for any integer $s \ge \sigma _*$, there exist $0< \varepsilon _0 \equiv \varepsilon _0(s, \gamma ) < 1$ (small) and $C_* = C_*(s, \gamma ) > 1$ (large) with the following property: for any $0 < \varepsilon \le \varepsilon _0$, there exists $T= T_{\varepsilon , s, \gamma } > 0$ so that for any initial data ${\mathfrak {x}}_0 = (\theta _0, y_0, w_0) \in {\mathbb {T}}^{S_+} \times {\mathbb {R}}^{S_+} \times H^s_\bot ({\mathbb {T}}_1)$ with

C.1 $$\begin{aligned} | y_0| \le \varepsilon \, , \quad \Vert w_0 \Vert _s \le \varepsilon \, , \end{aligned}$$

there exists a unique solution ${\mathfrak {x}}(t) = (\theta (t), y(t), w(t))$, $t \in [-T, T]$, of (C.1) with ${\mathfrak {x}}(0) = {\mathfrak {x}}_0$ satisfying

C.2 $$\begin{aligned}&\theta \in C^1([- T, T], {\mathbb {T}}^{S_+}), \quad y \in C^1([- T, T], {\mathbb {R}}^{S_+}), \nonumber \\&\quad \quad w \in C^0([- T, T], H^s_\bot ({\mathbb {T}}_1)) \cap C^1([- T, T], H^{s - 3}_\bot ({\mathbb {T}}_1)). \end{aligned}$$

Furthermore,

C.3 $$\begin{aligned} | y(t) |, \, \Vert w (t) \Vert _s\, , \, |\Theta (t)| \, \le C_* \varepsilon \qquad \forall t \in [- T, T]\,, \end{aligned}$$

where

C.4 $$\begin{aligned} \begin{aligned}&\Theta (t) := \theta (t) - \theta _0 + (\omega + \varepsilon {\widehat{\omega }}) t - \int _0^t \mathtt {N}^{(\theta )}(y(\tau ), w(\tau ))\, d \tau . \end{aligned} \end{aligned}$$

The rest of this appendix is devoted to the proof of Proposition C.1, which is based on an iterative scheme. For any given ${\mathfrak {x}}_0$ satisfying (C.2), define inductively a sequence ${\mathfrak {x}}^{(n)}(t) = (\theta ^{(n)}(t), y^{(n)}(t), w^{(n)}(t))$, $n \ge 0,$ as follows:

C.5 $$\begin{aligned} {\mathfrak {x}}^{(0)}(t) = ( \theta ^{(0)}(t), y^{(0)}(t), w^{(0)}(t)) : = {\mathfrak {x}}_0 = (\theta _0, y_0, w_0) \end{aligned}$$

whereas for $n \ge 1$, ${\mathfrak {x}}^{(n)}(t) = (\theta ^{(n )}(t), y^{(n )}(t), w^{(n )}(t))$ is defined to be the solution (cf. Lemma C.1 below) of

C.6 $$\begin{aligned} \begin{aligned} {\left\{ \begin{array}{ll} \partial _t \theta ^{(n )} = - \omega - \varepsilon {\widehat{\omega }} + \mathtt {N}^{(\theta )}(y^{(n )}, w^{(n )}) + {{\mathcal {O}}}_3^{(\theta )}({{\mathfrak {x}}}^{(n-1)}), \\ \partial _t y^{(n )} = {{\mathcal {O}}}_3^{(y)}({{\mathfrak {x}}}^{(n -1)}), \\ \partial _t w^{(n)} = \mathrm{i} \Omega _\bot w^{(n )} + {\mathtt {D}}^\bot ({{\mathfrak {x}}}^{(n-1)})[w^{(n )}] + \Pi _\bot T_{a({{\mathfrak {x}}}^{(n-1)})} \partial _x w^{(n )} + {{\mathcal {R}}}^\bot ({{\mathfrak {x}}}^{(n-1)}), \end{array}\right. } \end{aligned} \end{aligned}$$

with initial data ${\mathfrak {x}}^{(n)}(0) = {\mathfrak {x}}_0$. The following lemma holds.

### Lemma C.1

There exists $\sigma _*> 0$ (large) so that for any integer $s \ge \sigma _*$, there exist $\varepsilon _0 \equiv \varepsilon _0(s, \gamma ) > 0$ (small) and $C_*\equiv C_*(s, \gamma ) > 1$ (large) with the following property: for any $0 < \varepsilon \le \varepsilon _0(s, \gamma )$, there exists $T= T_{\varepsilon , s, \gamma } > 0$ so that for any initial data ${\mathfrak {x}}_0 = (\theta _0, y_0, w_0) \in {\mathbb {T}}^{S_+} \times {\mathbb {R}}^{S_+} \times H^s_\bot ({\mathbb {T}}_1)$ satisfying (C.2) and for any integer $n \ge 0$, the system (C.7) admits a unique solution, satisfying $\theta ^{(n)} \in C^1([- T, T], \, {\mathbb {T}}^{S_+}),$ $y^{(n)} \in C^1([- T, T], \, {\mathbb {R}}^{S_+})$, and

C.7 $$\begin{aligned} w^{(n)} \in C^0([- T, T], \, H^s_\bot ({\mathbb {T}}_1)) \cap C^1([- T, T], \, H^{s - 3}_\bot ({\mathbb {T}}_1)). \end{aligned}$$

Furthermore,

C.8 $$\begin{aligned} | y^{(n)}(t) |, \ \Vert w^{(n)} (t) \Vert _s\, , \ |\Theta ^{(n)}(t)| \, \le C_*\varepsilon \, , \qquad \forall t \in [- T, T]\,, \end{aligned}$$

where $$ \Theta ^{(0)}(t) := 0$$ and

C.9 $$\begin{aligned} \Theta ^{(n)} (t) := \theta ^{(n)} (t) - \theta _0 + (\omega + \varepsilon {\widehat{\omega }}) t - \int _0^t \mathtt {N}^{(\theta )}(y^{(n - 1)}(\tau ), w^{(n - 1)}(\tau ))\, d \tau , \quad n \ge 1\,.\nonumber \\ \end{aligned}$$

### Proof

We prove the claimed results by induction on *n*. For $n = 0$, by the definition (C.6) of ${\mathfrak {x}}^{(0)}(t)$, the claimed statement holds with $T = 1$ and with $\sigma _*$, $\varepsilon _0$ given as in Theorem 4.3. Now assume that the claimed statement holds at the step $n\ge 0$ of the induction and let us prove it at the step $n + 1$. We first need to make some preliminary considerations. Let $s \ge \sigma _*$ and $0 < \varepsilon \le \varepsilon _0$. Since by (4.21), *a* is small of order two and ${{\mathcal {R}}}^\bot$, ${{\mathcal {O}}}_3^{(\theta )}$, ${{\mathcal {O}}}_3^{(y)}$ are small of order three, it follows from Theorem 4.3 and the estimates (C.9), which hold by the induction hypothesis, that there exists a constant $C_s \equiv C_s( \gamma ) > 0$, independent of *n*, so that for any $t \in [- T, T]$

C.10 $$\begin{aligned} \begin{aligned}&\quad |{{\mathcal {O}}}_3^{(\theta )}({\mathfrak {x}}^{(n)}(t))|\,, \ |{{\mathcal {O}}}_3^{(y)}({\mathfrak {x}}^{(n)}(t))| \le C_s \varepsilon ^3, \qquad \Vert a({\mathfrak {x}}^{(n)}(t)) \Vert _{\sigma _*} \le C_s \varepsilon ^2, \\&\quad \qquad \Vert {{\mathcal {R}}}^\bot ({\mathfrak {x}}^{(n)}(t)) \Vert _s \le C_s \varepsilon ^3 \, \end{aligned} \end{aligned}$$

By the second equation in (C.7), one has

$$\begin{aligned} y^{(n + 1)}(t) = y_0 + \int _0^t {{\mathcal {O}}}_3^{(y)}({{\mathfrak {x}}}^{(n)}(\tau ))\, d \tau , \end{aligned}$$

implying that

C.11 $$\begin{aligned} y^{(n + 1)} \in C^1([- T, T], \, {\mathbb {R}}^{S_+}), \quad |y^{(n + 1)}(t)| \le \varepsilon + T C_s \varepsilon ^3 \le \, C_* \varepsilon , \quad \forall \, t \in [-T, T] \, ,\nonumber \\ \end{aligned}$$

where we have chosen $T>0$ so that $T C_s \varepsilon ^2 \le 1$ small enough. By (C.11) it then also follows that

C.12 $$\begin{aligned} T \Vert a({{\mathfrak {x}}}^{(n)}) \Vert _{\sigma _*} \le T C_s \varepsilon ^2 \le 1\,. \end{aligned}$$

To solve the equation for $w^{(n + 1)}$ in (C.7), we apply Lemma D.2 in Appendix D with ${{\mathcal {D}}}(t) = \mathrm{i} \Omega _\bot + {\mathtt {D}}^\bot ({{\mathfrak {x}}}^{(n)}(t))$, $a = a({{\mathfrak {x}}}^{(n)}(t))$, and $f = {{\mathcal {R}}}^\bot ({{\mathfrak {x}}}^{(n)}(t))$ to conclude that there exists a unique solution $w^{(n + 1)}$ of

$$\begin{aligned} {\left\{ \begin{array}{ll} \partial _t w^{(n + 1)} = \mathrm{i} \Omega _\bot w^{(n + 1)} + {\mathtt {D}}^\bot ({{\mathfrak {x}}}^{(n)})[w^{(n + 1)}] + \Pi _\bot T_{a({{\mathfrak {x}}}^{(n)})} \partial _x w^{(n + 1)} + {{\mathcal {R}}}^\bot ({{\mathfrak {x}}}^{(n)}) \\ w^{(n + 1)}(0) = w_0 \end{array}\right. } \end{aligned}$$

in $C^0([- T, T], \, H^s_\bot ({\mathbb {T}}_1)) \cap C^1([- T, T], \, H^{s - 3}_\bot ({\mathbb {T}}_1))$ and that $w^{(n+1)}$ satisfies

C.13 $$\begin{aligned} \Vert w^{(n + 1)}(t) \Vert _s , \ \Vert \partial _t w^{(n + 1)}(t) \Vert _{s - 3} \lesssim _{s, \gamma } \varepsilon + T \Vert {{\mathcal {R}}}^\bot ({{\mathfrak {x}}}^{(n)}) \Vert _s {\mathop {\lesssim _{s, \gamma } }\limits ^{ (C.11)}} \varepsilon + T C_s \varepsilon ^3 \le C_* \varepsilon \nonumber \\ \end{aligned}$$

since $T C_s\varepsilon ^2 \le 1$. We then define

C.14 $$\begin{aligned} \Theta ^{(n + 1)} (t) := \theta ^{(n + 1)} (t) - \theta _0 + (\omega + \varepsilon {\widehat{\omega }}) t - \int _0^t \mathtt {N}^{(\theta )}(y^{(n + 1)}(\tau ), w^{(n + 1)}(\tau ))\, d \tau . \quad t \in [- T, T].\nonumber \\ \end{aligned}$$

By the first equation in (C.7), one gets $\Theta ^{(n + 1)}(t) = \int _0^t {{\mathcal {O}}}_3^{(\theta )}({{\mathfrak {x}}}^{(n)}(\tau ))\, d \tau$ and hence, using again (C.11),

C.15 $$\begin{aligned} \theta ^{(n + 1)} \in C^1 ([- T, T], {\mathbb {T}}^{S_+}), \qquad |\Theta ^{(n + 1)}(t)| \le C_* \varepsilon , \quad \forall t \in [- T, T]\,. \end{aligned}$$

This concludes the proof of the lemma. $\square$

In order to prove the convergence of the sequence $({\mathfrak {x}}_n(t))_{n \ge 0}$, constructed in Lemma C.1, we prove

### Lemma C.2

Under the assumptions of Lemma C.1, for any $n \ge 1$,

$$\begin{aligned} \Vert {{\mathfrak {x}}}^{(n )}(\cdot ) - {{\mathfrak {x}}}^{(n - 1)}(\cdot ) \Vert _{{{\mathcal {C}}}^0_t E_{s - 1}}\,, \ \Vert \partial _t ({{\mathfrak {x}}}^{(n )}(\cdot ) - {{\mathfrak {x}}}^{(n - 1)}(\cdot )) \Vert _{{{\mathcal {C}}}^0_t E_{s - 4}} \le \, 2^{- n}\,. \end{aligned}$$

### Proof

By (C.7), $$\widehat{{\mathfrak {x}}}^{(n)}(t) = ({\widehat{\theta }}^{(n)}(t), {\widehat{y}}^{(n)}(t), {\widehat{w}}^{(n)}(t)) := {{\mathfrak {x}}}^{(n)}(t) - {{\mathfrak {x}}}^{(n -1)}(t) $$ satisfies

C.16 $$\begin{aligned} {\left\{ \begin{array}{ll} \partial _t {\widehat{\theta }}^{(n)} = f^{(\theta , n)}\, , \\ \partial _t {\widehat{y}}^{(n)} = f^{(y, n)}\, , \\ \partial _t {\widehat{w}}^{(n)} = \mathrm{i} \Omega _\bot {\widehat{w}}^{(n)} + {\mathtt {D}}^\bot ({{\mathfrak {x}}}^{(n)}) {\widehat{w}}^{(n)} + \Pi _\bot T_{a({{\mathfrak {x}}}^{(n)})} \partial _x {\widehat{w}}^{(n)}+ f^{(\bot , n)}, \end{array}\right. } \end{aligned}$$

with $$\widehat{{\mathfrak {x}}}^{(n)}(0) = (0, 0, 0)$$, where

C.17 $$\begin{aligned} \begin{aligned}&f^{(\theta , n)} := \mathtt {N}^{(\theta )}(y^{(n)}, w^{(n)}) - \mathtt {N}^{(\theta )}(y^{(n-1)}, w^{(n-1)}) + {{\mathcal {O}}}_3^{(\theta )}({{\mathfrak {x}}}^{(n)}) - {{\mathcal {O}}}_3^{(\theta )}({{\mathfrak {x}}}^{(n - 1)}) \,, \\&f^{(y, n)} := {{\mathcal {O}}}_3^{(y)}({{\mathfrak {x}}}^{(n)}) - {{\mathcal {O}}}_3^{(y)}({{\mathfrak {x}}}^{(n - 1)})\,, \\&f^{(\bot , n)} := \Big ( {\mathtt {D}}^\bot ({{\mathfrak {x}}}^{(n)}) - {\mathtt {D}}^\bot ({{\mathfrak {x}}}^{(n - 1)}) \Big )[w^{(n - 1)}] + \Pi _\bot T_{a({{\mathfrak {x}}}^{(n)}) - a({{\mathfrak {x}}}^{(n - 1)})} \partial _x w^{(n-1)}\\&\quad + {{\mathcal {R}}}^\bot ({{\mathfrak {x}}}^{(n)}) - {{\mathcal {R}}}^\bot ({{\mathfrak {x}}}^{(n - 1)}) \, . \end{aligned} \end{aligned}$$

By the properties stated in (4.21) and by the mean value theorem, for some $\sigma > 0$ large enough and $s \ge \sigma$, one can show that

C.18 $$\begin{aligned} \begin{aligned}&f^{(\theta , n)}, f^{(y, n)} \in C^0([- T, T], {\mathbb {R}}^{S_+}), \qquad |f^{(\theta , n)}| \lesssim \Vert {{\mathfrak {x}}}^{(n)} - {{\mathfrak {x}}}^{(n - 1)} \Vert _{{C}^0_t E_\sigma }, \\&\quad \quad |f^{(y, n)}| \lesssim \varepsilon ^2 \Vert {{\mathfrak {x}}}^{(n)} - {{\mathfrak {x}}}^{(n-1)} \Vert _{{C}^0_t E_\sigma } \,, \\&f^{(\bot , n)} \in C^0([- T, T], H^{s - 1}_\bot ({\mathbb {T}}_1)), \qquad \Vert f^{(\bot , n)} \Vert _{C^0_t H^{s - 1}_x} \lesssim _s \varepsilon \Vert {{\mathfrak {x}}}^{(n)} - {{\mathfrak {x}}}^{(n - 1)} \Vert _{{C}^0_t E_{ s - 1}}\,. \end{aligned} \end{aligned}$$

Hence we immediately conclude that for any $t \in [- T, T]$,

C.19 $$\begin{aligned} |{\widehat{\theta }}^{(n)} (t)| \lesssim T \Vert {{\mathfrak {x}}}^{(n)} - {{\mathfrak {x}}}^{(n - 1)} \Vert _{{C}^0_t E_\sigma }, \quad |{\widehat{y}}^{(n)}(t)| \lesssim T \varepsilon ^2 \Vert {{\mathfrak {x}}}^{(n)} - {{\mathfrak {x}}}^{(n-1)} \Vert _{{C}^0_t E_\sigma }.\qquad \end{aligned}$$

Furthermore, by applying Lemma D.2, with ${{\mathcal {D}}}(t) := \mathrm{i} \Omega _\bot + {\mathtt {D}}^\bot ({{\mathfrak {x}}}^{(n)})$, $a = a({{\mathfrak {x}}}^{(n)})$, $f = f^{(\bot , n)}$, and by the estimate (C.19) for $f^{(\bot , n)}$, one also deduces that

C.20 $$\begin{aligned} \Vert {\widehat{w}}^{(n)}(t) \Vert _{s - 1} \lesssim _s \varepsilon T \Vert {{\mathfrak {x}}}^{(n)} - {{\mathfrak {x}}}^{(n-1)} \Vert _{{{\mathcal {C}}}^0_t E_{s - 1}}, \quad \forall t \in [- T, T]\,. \end{aligned}$$

Therefore, collecting (C.20), (C.21), using the induction hypothesis, and by taking *T* small enough, one gets $\Vert {{\mathfrak {x}}}^{(n + 1)} - {{\mathfrak {x}}}^{(n)} \Vert _{{{\mathcal {C}}}^0_t E_{s - 1}} \le 2^{- (n + 1)}$ which is one of the two claimed estimates at the step $n + 1$. The estimate for $\partial _t ({{\mathfrak {x}}}^{(n+1)} - {{\mathfrak {x}}}^{(n)})$ can be proved in a similar fashion. $\square$

By Lemma C.2 and by a standard telescoping argument, one obtains

$$\begin{aligned} \theta ^{(n)} {\rightarrow } \theta , \quad y^{(n)} {\rightarrow } y\,,\quad \partial _t \theta ^{(n)} \rightarrow \partial _t \theta \,,\quad \partial _t y^{(n)} \rightarrow \partial _t y \quad \text {uniformly for} \quad - T \le t \le T \, . \end{aligned}$$

By the estimates (C.8) and by passing to the limit as $n \rightarrow + \infty$, one then obtains the bounds (C.3) for $\Theta (t)$ and *y*(*t*).

Furthermore,

$$\begin{aligned} w^{(n)} \rightarrow w \quad \text {in} \quad C^0([- T, T], \, H^{s - 1}_\bot ) \cap C^1([- T, T], \, H^{s - 4}_\bot ({\mathbb {T}}_1))\,. \end{aligned}$$

and $(\theta (t), y(t), w(t))$ is a smooth solution of (C.1). Furthermore, arguing as at the end of the proof of Lemma D.1, one shows that

$$\begin{aligned} w \in C^0([- T, T], \, H^s_\bot ({\mathbb {T}}_1)) \end{aligned}$$

and in turn, using the equation, that $\partial _t w \in C^0([- T, T], H^{s - 3}_\bot ({\mathbb {T}}_1))$. One also shows that *w*(*t*) satisfies the claimed bound (C.3) by using the bounds on $w^{(n)}$ in (C.9). To prove the uniqueness, take two smooth solutions ${{\mathfrak {x}}}_1, {{\mathfrak {x}}}_2$ satisfying the same initial condition ${{\mathfrak {x}}}_1(0) = {{\mathfrak {x}}}_0 = {{\mathfrak {x}}}_2(0)$. Then write the equation for the difference ${{\mathfrak {x}}}_1 - {{\mathfrak {x}}}_2$ and argue as in the proof of Lemma C.2 to conclude that

$$\begin{aligned} \Vert {{\mathfrak {x}}}_1(t) - {{\mathfrak {x}}}_2(t) \Vert _{E_{\sigma }} \lesssim \int _0^t \Vert {{\mathfrak {x}}}_1(\tau ) - {{\mathfrak {x}}}_2(\tau ) \Vert _{E_{\sigma }}\, d \tau , \quad \forall t \in [- T, T] \end{aligned}$$

for some $\sigma > 0$ (large). By the Gronwall Lemma, ${{\mathfrak {x}}}_1 = {{\mathfrak {x}}}_2$. This concludes the proof of Proposition C.1.

## On a Class of Linear Para-Differential Equations

In this appendix we discuss a well-posedness result for a linear para-differential equation of the form

C.21 $$\begin{aligned} \partial _t w = {{\mathcal {D}}}(t) [w] + \Pi _\bot T_a \partial _x w + f\,, \qquad x \in {\mathbb {T}}_1, \ t \in [-T, T] , \end{aligned}$$

in the Sobolev space $H^s_\bot ({\mathbb {T}}_1)$ for some integer $s \ge \sigma$ with $\sigma > 0$ sufficiently large. Here the linear operator ${{\mathcal {D}}}(t)$ is a time-dependent Fourier multiplier of order $m \ge 1$, ${{\mathcal {D}}}(t) w( x) = \sum _{n \in S^\bot } d_n(t)w_n e^{\mathrm{i} 2\pi n x}$ with

D.1 $$\begin{aligned} {{\mathcal {D}}} \in C^0\big ([- T, T], \, {{\mathcal {B}}}(H^{s }_\bot ({\mathbb {T}}_1), H^{s-m}_\bot ({\mathbb {T}}_1)) \big ), \quad {{\mathcal {D}}}(t) = - {{\mathcal {D}}}(t)^\top , \quad \forall t \in [- T, T]\, ,\nonumber \\ \end{aligned}$$

and the coefficient *a*(*t*, *x*) of the operator $T_a$ of para-multiplication by *a* and the forcing term *f*(*t*, *x*) satisfy

D.2 $$\begin{aligned} a \in C^0\big ( [- T, T], H^\sigma _\bot ({\mathbb {T}}_1) \big ), \qquad f \in C^0 \big ( [- T, T], H^s_\bot ({\mathbb {T}}_1) \big ) . \end{aligned}$$

The main result of this appendix is Lemma D.2 which is used in the proof of Proposition C.1.

First we consider the initial value problem for Eq. (D.1) with vanishing forcing term,

D.3 $$\begin{aligned} \partial _t w = {{\mathcal {D}}}(t) [w] + \Pi _\bot T_a \partial _x w\,, \qquad w(\tau , \cdot ) = w_0(\cdot ) \, , \end{aligned}$$

where the initial time $\tau$ is in $[- T, T]$.

### Lemma D.1

There exists $\sigma \ge m$ (large) with the following property: Assume that for some $0 < T \le 1$ and any $s \ge \sigma$, (D.2)–(D.3) hold and $\Vert a \Vert _{{{\mathcal {C}}}^0_t H^\sigma _x} \le 1$. Then for any $w_0 \in H^s_\bot ({\mathbb {T}}_1)$, there exists a unique solution *w* of (D.4) in $C^0([- T, T], H^s_\bot ({\mathbb {T}}_1)) \cap C^1([- T, T], H^{s - m}_\bot ({\mathbb {T}}_1))$. For any $t \in [- T, T]$, it satisfies the estimate

D.4 $$\begin{aligned} \begin{aligned}&\Vert w(t) \Vert _s\,,\, \Vert \partial _t w(t) \Vert _{s - m} \lesssim _s \Vert w_0 \Vert _s\,. \end{aligned} \end{aligned}$$

### Proof

The lemma is proved by constructing a sequence of approximating solutions. To this end we introduce for any integer $N \ge 1$ the finite dimensional subspace $H_N$ of $L^2_\bot ({\mathbb {T}}_1)$,

D.5 $$\begin{aligned} H_N := \big \{ u \in L^2_\bot ({\mathbb {T}}_1) : \, u(x) = \sum _{j \in S^\bot _N} u_n e^{\mathrm{i} 2 \pi n x}\big \}\, , \quad S^\bot _N:= S^\bot \cap [-N, N]\, ,\qquad \end{aligned}$$

and denote by $\Pi _N$ the corresponding $L^2-$orthogonal projector $\Pi _N : L^2_\bot ({\mathbb {T}}_1) \rightarrow H_N$. We consider the truncated equation

D.6 $$\begin{aligned} \partial _t w = \Pi _N \big ( {{\mathcal {D}}}(t) [w] + \Pi _\bot T_a \partial _x w \big ), \qquad w(\tau , \cdot ) = \Pi _N w_0\, , \end{aligned}$$

where $w(t, x) = \sum _{n \in S^\bot _N} w_n(t) e^{\mathrm{i} 2 \pi n x} \in H_N$. The equation in (D.7) is a linear non-autonomous ODE on the finite dimensional space $H_N$ and hence it admits a unique solution $w^{(N)} \in C^1([- T, T], H_N)$. We will show that the sequence $(w^{(N)})_{N \ge 1}$ admits a limit, which is the solution of (D.4) with the claimed properties. To this end, in a first step, we prove estimates for the Sobolev norm $\Vert w^{(N)}(t) \Vert _{s}$.

Bound of $\Vert w^{(N)}(t) \Vert _{s}$. Note that $\Vert w^{(N)}(t) \Vert _{s} = \Vert \partial _x^s w^{(N)}(t) \Vert$. Since ${{\mathcal {D}}}(t)$ is a Fourier multiplier, the commutator $[\partial _x^s, \, {{\mathcal {D}}}(t)]$ vanishes and since for any $v \in L^2_\bot ({\mathbb {T}}_1)$,

$$\begin{aligned} \big \langle \Pi _\bot u,\, v \big \rangle = \big \langle u,\, v \big \rangle \, , \ \ \forall \, u \in L^2({\mathbb {T}}_1) \, , \qquad \big \langle \Pi _N v , \, g \big \rangle = \big \langle v,\, g \big \rangle \, , \ \ \forall \, g \in H_N, \end{aligned}$$

one concludes that

D.7 $$\begin{aligned} \partial _t \Vert \partial _x^s w^{(N)} \Vert&= \big \langle \partial _x^s \big ( {{\mathcal {D}}}(t) [w^{(N)}] + \Pi _N \Pi _\bot T_a \partial _x w^{(N)} \big ) , \, \partial _x^s w^{(N)} \big \rangle \nonumber \\&\quad + \big \langle \partial _x^s w^{(N)} , \, \partial _x^s \big ( {{\mathcal {D}}}(t) [w^{(N)}] + \Pi _N \Pi _\bot T_a \partial _x w^{(N)} \big )\big \rangle \nonumber \\&= \big \langle {{\mathcal {D}}}(t) \partial _x^s w^{(N)} , \, \partial _x^s w^{(N)} \big \rangle + \big \langle \partial _x^s w^{(N)}, \, {{\mathcal {D}}}(t) \partial _x^s w^{(N)} \big \rangle \end{aligned}$$

D.8 $$\begin{aligned}&\quad + \big \langle \partial _x^s T_a \partial _x w^{(N)} ,\, \partial _x^s w^{(N)} \big \rangle + \big \langle \partial _x^s w^{(N)} ,\, \partial _x^s T_a \partial _x w^{(N)} \big \rangle \,. \end{aligned}$$

*Analysis of the terms in* (D.8). Since by assumption ${{\mathcal {D}}}(t)^\top = - {{\mathcal {D}}}(t)$, one has

D.9 $$\begin{aligned} \begin{aligned}&\big \langle {{\mathcal {D}}}(t) \partial _x^s w^{(N)}, \, \partial _x^s w^{(N)} \big \rangle + \big \langle \partial _x^s w^{(N)}, \, {{\mathcal {D}}}(t) \partial _x^s w^{(N)} \big \rangle \\&\quad = \big \langle \big ( {{\mathcal {D}}}(t) + {{\mathcal {D}}}(t)^\top \big ) \partial _x^s w^{(N)}, \, \partial _x^s w^{(N)} \big \rangle = 0\,. \end{aligned} \end{aligned}$$

*Analysis of the terms in* (D.9). One computes

D.10 $$\begin{aligned} \begin{aligned}&\big \langle \partial _x^s T_{a} \partial _x w^{(N)}\,,\, \partial _x^s w^{(N)} \big \rangle + \big \langle \partial _x^s w^{(N)} \,,\, \partial _x^s T_{a} \partial _x w^{(N)}\big \rangle \\&\quad = \big \langle T_{a} \partial _x \partial _x^s w^{(N)}\,,\, \partial _x^s w^{(N)} \big \rangle + \big \langle \partial _x^s w^{(N)} \,,\, T_{a} \partial _x \partial _x^s w^{(N)}\big \rangle \\&\qquad + \big \langle [\partial _x^s, T_{a} \partial _x] w^{(N)}\,,\, \partial _x^s w^{(N)} \big \rangle + \big \langle \partial _x^s w^{(N)} \,,\, [\partial _x^s, T_{a} \partial _x] w^{(N)}\big \rangle \\&\quad = \big \langle \big ( T_{a} \partial _x + (T_{a} \partial _x)^\top \big ) \partial _x^s w^{(N)}\,,\, \partial _x^s w^{(N)} \big \rangle \\&\qquad + \big \langle [\partial _x^s, T_{a} \partial _x] w^{(N)}\,,\, \partial _x^s w^{(N)} \big \rangle + \big \langle \partial _x^s w^{(N)} \,,\, [\partial _x^s, T_{a} \partial _x] w^{(N)}\big \rangle \,. \end{aligned} \end{aligned}$$

By Corollary 2.2 (with $N=1$, $m=1$) there exists an integer $\sigma \ge 1$ so that

$$\begin{aligned} \Vert T_{a} \partial _x + (T_{a} \partial _x)^\top \Vert _{{{\mathcal {B}}}(L^2)} \lesssim \Vert a \Vert _{\sigma } \end{aligned}$$

and hence by the Cauchy–Schwarz inequality,

D.11 $$\begin{aligned} \begin{aligned} |\big \langle \big ( T_{a} \partial _x + (T_{a} \partial _x)^\top \big ) \partial _x^s w^{(N)}\,,\, \partial _x^s w^{(N)} \big \rangle | \lesssim \Vert a \Vert _\sigma \, \Vert \partial _x^s w^{(N)} \Vert ^2 \,. \end{aligned} \end{aligned}$$

Moreover, arguing as in [9, Lemma A.1], one has

$$\begin{aligned} \Vert [\partial _x^s, \, T_{a} \partial _x] w^{(N)}\Vert \lesssim _s \Vert a \Vert _2 \Vert w^{(N)} \Vert _s {\mathop {\lesssim _s}\limits ^{\sigma \ge 2}} \Vert a \Vert _\sigma \, \Vert \partial _x^s w^{(N)} \Vert \,. \end{aligned}$$

The latter estimate, together with the Cauchy–Schwarz inequality, imply that

D.12 $$\begin{aligned} \begin{aligned} |\big \langle [\partial _x^s, \, T_{a} \partial _x] w^{(N)}, \, \partial _x^s w^{(N)} \big \rangle + \big \langle \partial _x^s w^{(N)} \,,\, [\partial _x^s, \, T_{a} \partial _x] w^{(N)}\big \rangle | \lesssim _s \Vert a \Vert _\sigma \, \Vert \partial _x^s w^{(N)} \Vert ^2\,.\qquad \quad \end{aligned} \end{aligned}$$

Using (D.12)–(D.13), one then infers from (D.11)

D.13 $$\begin{aligned} |\big \langle \partial _x^s T_{a} \partial _x w^{(N)}, \, \partial _x^s w^{(N)} \big \rangle + \big \langle \partial _x^s w^{(N)} ,\, \partial _x^s T_{a} \partial _x w^{(N)}\big \rangle | \lesssim _s \Vert a \Vert _\sigma \Vert \partial _x^s w^{(N)} \Vert ^2 \, . \end{aligned}$$

Combining (D.8), (D.9), (D.10), (D.14), yields the estimate

D.14 $$\begin{aligned} |\, \partial _t \Vert \partial _x^s w^{(N)} \Vert ^2| \lesssim _s \Vert a \Vert _\sigma \Vert \partial _x^s w^{(N)} \Vert ^2 , \end{aligned}$$

which implies that

D.15 $$\begin{aligned} \begin{aligned} \Vert \partial _x^s w^{(N)} (t)\Vert _{L^2}^2&\le \Vert w_0 \Vert _{s}^2 + C(s) \Big | \int _\tau ^t \Vert a(t' ) \Vert _\sigma \Vert \partial _x^s w^{(N)}(t') \Vert ^2\, d t' \Big | \\&{\le } \Vert w_0 \Vert _s^2 + C(s) \Vert a \Vert _{{{\mathcal {C}}}^0_t H^\sigma _x} \Big | \int _\tau ^t \Vert \partial _x^s w^{(N)}(t') \Vert ^2\, d t' \Big | \end{aligned} \end{aligned}$$

for some constant $C(s) > 0$. The Gronwall Lemma (recall that $- T \le t, \tau \le T$) then implies that

$$\begin{aligned} \Vert w^{(N)}(t) \Vert _s^2= \Vert \partial _x^s w^{(N)} \Vert ^2 \le \mathrm{exp} \big (C(s) \Vert a \Vert _{{{\mathcal {C}}}^0_t H^\sigma _x} T \big ) \Vert w_0 \Vert _s^2, \quad \forall t \in [- T, T]\,. \end{aligned}$$

Since by assumption $0 < T \le 1$ and $\Vert a \Vert _{{{\mathcal {C}}}^0_t H^\sigma _x} \le 1$, it then follows that

D.16 $$\begin{aligned} \Vert w^{(N)}(t) \Vert _s^2= \Vert \partial _x^s w^{(N)} \Vert ^2 \le \mathrm{exp} (C(s)) \Vert w_0 \Vert _s^2, \quad \forall t \in [- T, T]\,. \end{aligned}$$

Convergence. Now we pass to the limit $N \rightarrow + \infty$. By (D.17) the sequence of functions $w^{(N)}$ is bounded in $C^0([- T, T], \, H^s_\bot ({\mathbb {T}}_1)) \subseteq L^\infty ([- T, T], \, H^s_\bot ({\mathbb {T}}_1))$ and, up to subsequences,

D.17 $$\begin{aligned} w^{(N)} {\mathop {\rightharpoonup }\limits ^{w^*}} w \ \ \text {in } \ L^\infty ([- T, T], H^s_\bot ({\mathbb {T}}_1)) \, , \quad \Vert w \Vert _{L^\infty _{ t} H^s_x} \le \liminf _{N \rightarrow + \infty } \Vert w^{(N)} \Vert _{L^\infty _{ t} H^s_x}\,.\qquad \end{aligned}$$

*Claim:* $( w^{(N)})_{N \ge 1}$ converges to *w* in $C^0([- T, T], \, H^{s}_\bot ({\mathbb {T}}_1)) \cap C^1([- T, T], \, H^{ s - m}_\bot ({\mathbb {T}}_1))$, and *w* solves (D.4).

We first prove that $w^{(N)}$ is a Cauchy sequence in $C^0([- T, T], L^2_\bot ({\mathbb {T}}_1) )$. Indeed, by (D.7), the difference $h^{(N)} := w^{(N + 1)} - w^{(N)}$ solves

$$\begin{aligned}&\partial _t h^{(N)} = {{\mathcal {D}}}(t) h^{(N)} + \Pi _{N+ 1} ( \Pi _\bot T_a \partial _x h^{(N)} ) +(\Pi _{N + 1} - \Pi _N) \Pi _\bot T_a \partial _x w^{(N)}\,, \\&\quad \qquad h^{(N)}(\tau ) = (\Pi _{N + 1} - \Pi _N) w_0 \, , \end{aligned}$$

and therefore

D.18 $$\begin{aligned} \partial _t \Vert h^{(N)}(t)\Vert ^2&= \big \langle \partial _t h^{(N)}, \,h^{(N)} \big \rangle + \big \langle h^{(N)}, \,\partial _t h^{(N)} \big \rangle \nonumber \\&= \big \langle {{\mathcal {D}}}(t) h^{(N)}, \, h^{(N)} \big \rangle + \big \langle h^{(N)}, \, {{\mathcal {D}}}(t) h^{(N)} \big \rangle + \big \langle T_a \partial _x h^{(N)} ,\, h^{(N)} \big \rangle + \big \langle h^{(N)},\, T_a \partial _x h^{(N)} ) \big \rangle \nonumber \\&\quad + \big \langle (\Pi _{N + 1} - \Pi _N)\Pi _\bot T_a \partial _x w^{(N)}, \, h^{(N)} \big \rangle + \big \langle h^{(N)}, \, (\Pi _{N + 1} - \Pi _N) \Pi _\bot T_a \partial _x w^{(N)} \big \rangle \,. \end{aligned}$$

Arguing as in (D.10), (D.11)–(D.14), one gets

D.19 $$\begin{aligned} \begin{aligned}&\big \langle {{\mathcal {D}}}(t) h^{(N)}, \, h^{(N)} \big \rangle + \big \langle h^{(N)}, \, {{\mathcal {D}}}(t) h^{(N)} \big \rangle = 0\,, \\&| \big \langle T_a \partial _x h^{(N)} ,\, h^{(N)} \big \rangle + \big \langle h^{(N)} , \, T_a \partial _x h^{(N)} )| \lesssim \Vert a \Vert _\sigma \Vert h^{(N)} \Vert ^2\,. \end{aligned} \end{aligned}$$

Moreover

D.20 $$\begin{aligned}&| \big \langle (\Pi _{N + 1} - \Pi _N)\Pi _\bot T_a \partial _x w^{(N)}, h^{(N)} \big \rangle + \big \langle h^{(N)}, (\Pi _{N + 1} - \Pi _N) \Pi _\bot T_a \partial _x w^{(N)} \big \rangle | \nonumber \\&\lesssim \Vert (\Pi _{N + 1} - \Pi _N) \Pi _\bot T_a \partial _x w^{(N)} \Vert \Vert h^{(N)}\Vert \lesssim \Vert h^{(N)}\Vert ^2 + \Vert (\Pi _{N + 1} - \Pi _N) \Pi _\bot T_a \partial _x w^{(N)} \Vert ^2 \nonumber \\&\lesssim \Vert h^{(N)}\Vert ^2 + \big ( N^{-2} \Vert T_a \partial _x w^{(N)} \Vert _2 \big )^2 {\mathop {\lesssim }\limits ^{(2.2),(D.17)}} \Vert h^{(N)}\Vert ^2 + \big ( N^{-2} \Vert w_0 \Vert _3 \big )^2 \nonumber \\&\quad {\mathop {\lesssim }\limits ^{\sigma \ge 3}} \Vert h^{(N)}\Vert ^2 + \big ( N^{-2} \Vert w_0 \Vert _{\sigma } \big )^2\,. \end{aligned}$$

Hence (D.19)–(D.21) imply that

$$\begin{aligned} \partial _t \Vert h^{(N)}(t)\Vert ^2 \lesssim \Vert h^{(N)}(t)\Vert ^2 + N^{-4}\Vert w_0\Vert _{\sigma }^2 \end{aligned}$$

and, since $\Vert h^{(N)}(\tau )\Vert \le N^{- 2} \Vert w_0\Vert _{2}$, we deduce from the Gronwall Lemma that

$$\begin{aligned} \Vert w^{(N + 1)} - w^{(N)}\Vert _{{{\mathcal {C}}}^0_{ t} L^2_x} \lesssim N^{-2} \Vert w_0\Vert _\sigma \, \mathrm{exp}(C T)^{\frac{1}{2}} \end{aligned}$$

for some constant $C > 0$. The above inequality, together with a standard telescoping argument implies that $w^{(N)}$ is a Cauchy sequence in $C^0([- T, T], \, L^2_\bot ({\mathbb {T}}_1))$. Hence $w^{(N)} \rightarrow {\tilde{w}} \in C^0([- T, T], \, L^2_\bot ({\mathbb {T}}_1))$. By (D.18) we have

$$\begin{aligned} {\tilde{w}} = w \in C^0([- T, T], L^2_\bot ({\mathbb {T}}_1)) \cap L^\infty ([- T, T], \, H^s_\bot ({\mathbb {T}}_1)).\end{aligned}$$

Next, for any ${\bar{s}} \in [0, s)$ one has by the interpolation inequality

$$\begin{aligned} \Vert w^{(N)} - w \Vert _{L^\infty _{t} H^{{\bar{s}}}_x } \le \Vert w^{(N)} - w \Vert _{L^\infty _{ t} L^2_x }^{1 - \lambda } \, \Vert w^{(N)} - w \Vert _{L^\infty _{ t} H^{ s}_x }^\lambda \, , \qquad \lambda := {\bar{s}} / s, \end{aligned}$$

and, since $w^{(N)}$ is bounded in $L^\infty ([- T, T], H^s_\bot ({\mathbb {T}}_1))$ (see (D.17)), $w \in L^\infty ([- T, T], H^s_\bot ({\mathbb {T}}_1))$, and $w^{(N)} \rightarrow w \in C^0([- T, T], \, L^2_\bot ({\mathbb {T}}_1))$, we deduce that $w^{(N)} \rightarrow w$ in $C^0([- T, T], \, H^{{\bar{s}}}_\bot ({\mathbb {T}}_1))$. Moreover we deduce

$$\begin{aligned} \partial _t w^{(N)}&= \Pi _N \big ( {{\mathcal {D}}}(t) [w^{(N)}] + \Pi _\bot T_a \partial _x w^{(N)} \big ) \rightarrow \\&\quad {{\mathcal {D}}}(t) [w] + \Pi _\bot T_a \partial _x w \quad \text {in}\ \ C^0([- T, T], \, H^{{\bar{s}} - m}_\bot ({\mathbb {T}}_1) ) \, , \quad \forall {\bar{s}} \in [0, s) \, . \end{aligned}$$

As a consequence $w \in C^1([- T, T], \, H^{{\bar{s}} - m}_\bot ({\mathbb {T}}_1) )$ and $\partial _t w = {{\mathcal {D}}}(t) [w] + \Pi _\bot T_a \partial _x w$ solves (D.4).

Finally, arguing as in [42], Proposition 5.1.D, it follows that the function $t \rightarrow \Vert w(t)\Vert _{s}^2$ is Lipschitz. Furthermore, one can show that if $t_n \rightarrow t$ then $w(t_n ) \rightharpoonup w(t)$ weakly in $H^s_\bot ({\mathbb {T}}_1)$, because $w(t_n) \rightarrow w(t)$ in $H^{{\bar{s}}}_\bot ({\mathbb {T}}_1)$ for any ${\bar{s}} \in [0, s)$. As a consequence the sequence $w(t_n) \rightarrow w(t)$ strongly in $H^s_\bot ({\mathbb {T}}_1)$. This proves that $w \in C^0([- T, T], H^s_\bot ({\mathbb {T}}_1))$ and therefore $\partial _t w \in C^0([- T, T], H^{s - m}_\bot ({\mathbb {T}}_1))$.

Uniqueness. If $w_1, w_2 \in C^0([- T, T], H^{s}_\bot ({\mathbb {T}}_1)) \cap C^1([- T, T], H^{s - m}_\bot ({\mathbb {T}}_1))$, $s \ge \sigma$, are solutions of (D.4) with $w_1(\tau ) = w_2(\tau ) \in H^s_\bot ({\mathbb {T}}_1)$, then $h: = w_1 - w_2$ solves

$$\begin{aligned} \partial _t h = {{\mathcal {D}}}(t) h + \Pi _\bot T_a \partial _x h\,, \qquad h(\tau ) = 0\,. \end{aligned}$$

Arguing as in the proofs of the previous energy estimates, we deduce the energy inequality $\partial _t \Vert h (t)\Vert ^2 \le C \Vert h(t)\Vert ^2$. Since $h(\tau )= 0$, the Gronwall Lemma implies that $\Vert h(t)\Vert ^2 = 0$, for any $t \in [-T, T]$. This shows the uniqueness.

The estimate for $\Vert w \Vert _{s }$ in (D.5) then follows by (D.17)–(D.18) and the one of $\Vert \partial _t w \Vert _{s - m}$ in (D.5) by using the equation. $\quad \square$

In the next lemma we consider the inhomogeneous equation (D.1).

### Lemma D.2

Let $\sigma \ge m$ and *m* be given as in Lemma D.1 and assume that for some $0 < T \le 1$ and $s \ge \sigma$, (D.2)–(D.3) hold and $\Vert a \Vert _{{{\mathcal {C}}}^0_t H^\sigma _x} \le 1$. Then for any $w_0 \in H^s_\bot ({\mathbb {T}}_1)$, there exists a unique solution $t \mapsto w(t)$ of (D.1) in $C^0([- T, T], H^s_\bot ({\mathbb {T}}_1)) \cap C^1([- T, T], H^{s - m}_\bot ({\mathbb {T}}_1))$, with $w(0) = w_0$. For any $t \in [- T, T]$ it satisfies,

D.21 $$\begin{aligned} \begin{aligned}&\Vert w(t) \Vert _s \lesssim _s \Vert w_0 \Vert _s + \int _0^t \Vert f(\tau ) \Vert _s \,d \tau \lesssim _s \Vert w_0 \Vert _s + T \Vert f \Vert _{{{\mathcal {C}}}^0_t H^s_x}\,, \\&\Vert \partial _t w(t) \Vert _{s - m} \lesssim _s \Vert w_0 \Vert _s + T \Vert f \Vert _{{{\mathcal {C}}}^0_t H^s_x}\,, \quad \forall t \in [- T, T]. \end{aligned} \end{aligned}$$

### Proof

For any $t, \tau \in [-T, T]$, denote by $\Phi (\tau , t)$ the flow map of the para-differential equation (D.4),

$$\begin{aligned} \partial _t w = {{\mathcal {D}}}(t) [w] + \Pi _\bot T_a \partial _x w\,, \qquad w(\tau , \cdot ) = w_0(\cdot ) \, . \end{aligned}$$

By Lemma D.1, $\Phi (\tau , t)$ is a bounded linear operator $H^s_\bot ({\mathbb {T}}_1) \rightarrow H^s_\bot ({\mathbb {T}}_1)$ for any $s \ge \sigma$. The estimate (D.5) implies that

$$\begin{aligned} \Vert \Phi (\tau , t) w_0 \Vert _s \lesssim _s \Vert w_0 \Vert _s , \qquad \Vert \partial _t \Phi (\tau , t) w_0 \Vert _{s - m} \lesssim _s \Vert w_0 \Vert _s\,. \end{aligned}$$

The unique solution of the Eq. (D.1) in $C^0([- T, T], H^s_\bot ({\mathbb {T}}_1)) \cap C^1([- T, T], H^{s - m}_\bot ({\mathbb {T}}_1))$ with initial data $w(0) = w_0$ is then given by the Duhamel formula $w(t) = \Phi (0, t) w_0 + \int _0^t \Phi (\tau , t) f(\tau )\, d \tau$ and the claimed estimates easily follow. $\square$
